# Targeting the Energy-Coupling Factor Transporters: A Novel Antibiotic Drug Target in *Streptococcus pneumoniae*

**DOI:** 10.1021/acs.jmedchem.5c03638

**Published:** 2026-06-12

**Authors:** Eleonora Diamanti, Amelieke J.H. Cremers, Yue Li, Ioulia Exapicheidou, Carole Baumann, Paddy Gibson, Atanaz Shams, Louise Martin, Rouven Becker, Inda Setyawati, Lucie Zeimetz, Jörg Haupenthal, Matthias Witschel, Dirk J. Slotboom, Jennifer Herrmann, Katharina Rox, Mostafa M. Hamed, Jan-Willem Veening, Anna K. H. Hirsch

**Affiliations:** 1 Helmholtz Institute for Pharmaceutical Research (HIPS)-Helmholtz Centre for Infection Research (HZI), Campus Building E 8.1, Saarbrücken, Saarland D-66123, Germany; 2 Department of Pharmacy, Saarland University, Campus E8.1, Saarbrücken, Saarland 66123, Germany; 3 Department of Fundamental Microbiology, Faculty of Biology and Medicine, 30588 University of Lausanne, Lausanne CH-1015, Switzerland; 4 Groningen Biomolecular Sciences and Biotechnology Institute, University of Groningen, Nijenborgh 4, Groningen 9747AG, The Netherlands; 5 Helmholtz Institute for Pharmaceutical Research (HIPS) -Helmholtz Centre for Infection Research (HZI) Campus Building E 8.1, Saarbrücken, Saarland D-66123, Germany; 6 Saarland University, PharmaScienceHub (PSH), Campus E2.1, Saarbrücken, Saarland 66123, Germany; 7 BASF-SE Carl-Bosch-Strasse 38, Ludwigshafen 67056, Germany; 8 Department of Chemical Biology, Helmholtz Centre for Infection Research (HZI), Inhoffenstraße 7, Braunschweig 38124, Germany; 9 German Center for Infection Research (DZIF), Partner Site Hannover-Braunschweig Inhoffenstraße 7, Braunschweig 38124, Germany

## Abstract

Energy-Coupling Factor (ECF) transporters are a hitherto underexplored target involved in the uptake of several micronutrients in bacteria and are absent in humans. Here, in *Streptococcus pneumoniae* , we demonstrate that the genes encoding ECF transporters are highly conserved and their expression is crucial for causing bacterial infection in both murine (*in vivo*) and human (*ex vivo*) infection models. Next, we demonstrated that the antimicrobial activity of our inhibitors against *S. pneumoniae* is related to the level of the ECF transporter expressed by the bacterium, confirming the target engagement of this chemical class in *S. pneumoniae* . The pharmacokinetic studies conducted revealed high peroral bioavailability for **4**, which was also assessed in a murine neutropenic lung-infection model with *S. pneumoniae* , and observed a one log_10_ reduction in bacterial load compared to vehicle. This work sets the stage for an innovative approach to combat *S. pneumonia* -derived infections by targeting the ECF transporters with the novel chemotype reported.

## Graphical abstract

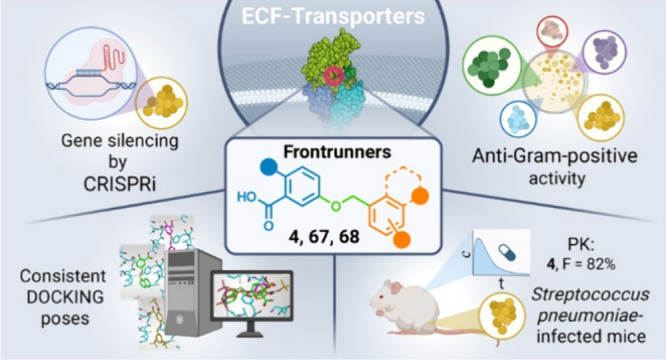

## Introduction

Drug-resistant bacteria are a global health threat that has escalated over the past few years.^1^ New therapies to cure bacterial infections and to combat the antimicrobial-resistance crisis are urgently needed.^2^ Despite this alarming situation, the World Health Organization (WHO) recently stated that the antibiotics pipeline is drying out and that most new antibiotics that reach the market are traditional antibiotics with a known mode of action.^3^ Based on the criteria defined by the WHO, an agent is defined as “innovative” only when it meets the three criteria: it is a new class (new scaffold or pharmacophore), it addresses a new target (new binding site), and it has a new mode of action.^4^ The intention of this “hard to find but long-term approach” is to minimize the risk of cross-resistance to currently applied antibiotics.^5^

Here, we present this combination by introducing a novel chemical scaffold engaging a hitherto underexplored drug target named the energy-coupling factor (ECF) transporter.^6^ The ECF transporters are a recently disclosed class of transmembrane proteins involved in the uptake of important vitamins or other nutrients (such as Ni^2+^ or Co^2+^ ions), mainly in Gram-positive species.^7^ Their selective presence in prokaryotic species and not in humans, together with their contribution to the survival and growth of the bacterium, make them an attractive antibacterial target.^8^

The selective uptake of solutes by ECF transporters is ruled by an unprecedented architecture and a unique mechanism of action. They are endowed with two specific features: a substrate-specific component (S-component, integral membrane protein) and an energy-coupling module (Figure 1).^9^

**Figure 1:**
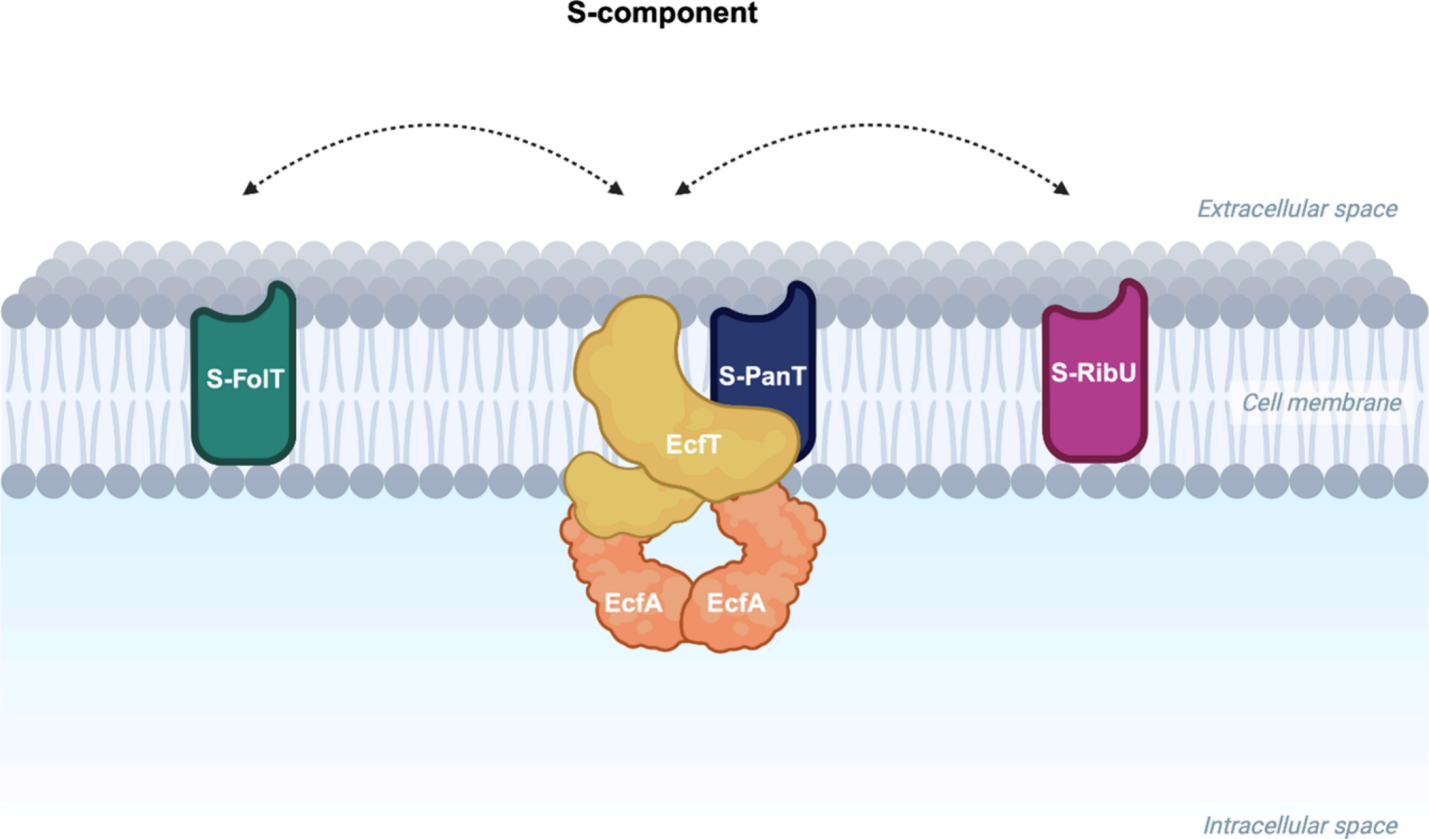
Schematic representation of the composition of an energy-coupling factor (ECF) transporter comprising two transmembrane proteins (EcfT and S-component) and two ATP-hydrolyzing domains (EcfA and A′). Brown: the EcfT has an L shape with each coupling helix making tight contacts with a single ATPase subunit ensuring joint movement. Different colors: S-components bind the transported substrate(s) of ECF transporters. Orange: ATPase subunits. ATP hydrolysis is essential for substrate transport.

The energy-coupling module is a ternary complex: (*i*) EcfT or T component, (*ii*) EcfA, and (*iii*) EcfA′ or A component. The T component spans the membrane and is exposed to the extracellular compartment, while the A module lies on the cytosolic side and consists of two similar or identical ATPases (A and A′). The ATPases are the common element within the superfamily of the ABC transporters.^10^ The uptake of substrate by an ECF transporter is a dynamic cycle, and only recently, cryogenic electron microscopy (cryo-EM) has shed light on the chemo-mechanical coupling in the transport cycle of ECF transporters, triggered not by ATP hydrolysis, but by Mg-ATP binding.^11^ This study showed a unique powering transport where the Mg-ATP binding corresponds to a significant motion in the entire EcfT, with the long coupling helices switching from a concave to a convex shape and the withdrawal of the S-component toward the lipid bilayer. Afterward, the hydrolysis of ATP and release of ADP and Pi dictate both the return of the coupling helices to their original concave shape and the S-component orientation toward the extracellular environment, ready to host another vitamin. As the movement of the coupling helices is crucial for vitamin transport, we aimed to specifically block this conformational transition. To achieve this, we launched a structure-based virtual screening (SBVS) campaign using the X-ray crystal structure of the folate-specific transporter ECF-FolT2 from *Lactobacillus delbrueckii* (PDB ID: 5JSZ^12^) that enabled us to identify the first ECF inhibitors.^13,14^

Aside from that, we have described another ECF inhibitor chemotype,^15^ we identified compounds acting as substrate-mimicking inhibitors for the S-component ThiT from *Lactococcus lactis*,^16^ and ultimately, we applied a protein-templated approach, called dynamic combinatorial chemistry, to speed up our medicinal-chemistry optimization.^17^ While each approach separately led to compounds with considerable potency, the utility of these agents is hindered by low chemical and metabolic stability that needs further optimization. Thus, the field still lacks suitable ECF small-molecule inhibitors to foster drug discovery and development.

Recently, we have disclosed that ligand **1** may bind at the protein–protein interaction interface between the S-component and ECF module using coarse-grained molecular-dynamics simulations on *L. delbrueckii* ECF-FolT2 and ECF-PanT.^13^

Preliminary structure–activity relationship (SAR) studies showed that the salicylic acid motif, as in the initial hit **1**, is crucial for the interaction. Removal of the carboxylic group (compound **2**) led to a loss of inhibitory activity; while a small growth of **1** with a carbamate-branched chain, led to a 6-fold increase in inhibitory potency (**3**, IC_50_ = 49.2 ± 3.4 μM). This boost in potency is rationally explained as, in addition to the hydrogen bond with Ser173 provided by the NH (or OH in the hit **1**), an additional hydrophobic interaction with Leu 172 is furnished by the *tert*-butyl group. Addition of a bromine in position 4 of the naphthalene ring led to compound **4** showing also a pronounced boost in activity (Figure 2).^18,19^ Compound **4** emerged as one of our earliest optimized hits that progressed into early *in vivo* evaluation in a *Galleria mellonella* infection model.^19^

**Figure 2:**
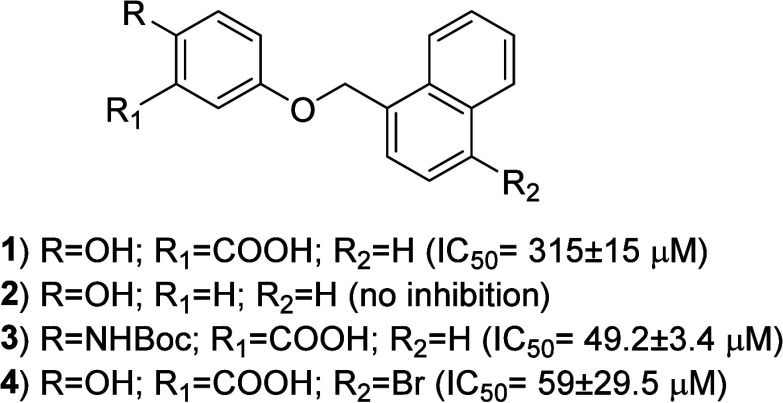
Chemical structures of reported energy-coupling factor (ECF) transporter inhibitors **1**–**4** and respective on-target activities using the whole-cell uptake assay in *Lactobacillus casei*.^13,14,18,19^

In the present study, we expanded the initial SAR study (Figure 2) around compound **1** to elucidate the key molecular features crucial for interaction. Figure 3 summarizes the steps pursued in this manuscript, starting from the VS hit, the subsequent SAR optimization, and finally the biological evaluation that led to the selection of three frontrunner compounds (**4**, **67**, and **68**). Additionally, here for the first time, we demonstrated that the ECF transporter is conserved and essential for the growth of the opportunistic human pathogen *Streptococcus pneumoniae* . We also reported that the antimicrobial activity of the synthesized inhibitors against *S. pneumoniae* was dependent on the expression of the ECF transporter.

**Figure 3:**
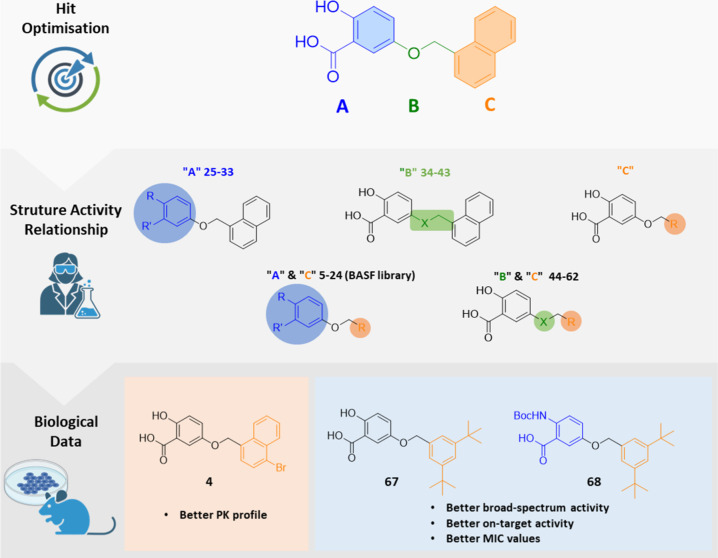
Schematic overview of the workflow performed in this study. PK: pharmacokinetics; MIC: minimal inhibitory concentration.

## Results and Discussion

### SAR Investigation

The structure of hit **1** consists of a salicylic acid ring (blue, A) bridged to a naphthyl ring (orange, C) by an ether linker (green, B) as depicted at the top of Figure 3. We pursued a focused SAR study on each part of the molecule both by testing a subset of molecules from the BASF library (Table S1, compounds **5**–**24**) and by the synthesis of compounds **25**–**70**.

All the compounds (**5**–**70**) were initially screened for their ability to inhibit the transport of vitamins operated by ECF transporters, and to do that efficiently, we implemented a new workflow for the biological evaluation. Until now, we were mainly relying on a robust, albeit low-throughput activity assay for which we purified the ECF transporter ECF-FolT2, reconstituted the protein into liposomes, and measured ATP-dependent uptake of radiolabeled folate into the lumen of the proteoliposomes (proteoliposome assay, see Section 4.0 of the Supporting Information). The low-throughput nature of this assay hampered our ability to screen larger numbers of compounds in a short time.^12^ To overcome this limitation, we recently established a novel whole-cell assay,^14^ and here, we relied on both complementary assays. Importantly, both assays gave comparable results, highlighting their interchangeability. Thus, we first screened all compounds at a concentration of 200 μM in a whole-cell uptake assay using *Lactobacillus casei* as an organism that constitutively expresses the ECF transporters. Afterward, only the compounds showing more than 90% inhibition at the concentration of 200 μM were selected for IC_50_ determination. Subsequently, we only validated inhibitors that showed promising IC_50_ values (<20 μM) in the proteoliposome uptake assay. This stepwise screening workflow provided the opportunity to quickly screen multiple compounds while minimizing false positives by relying on the two complementary assays. A sequence-conservation study between *L. delbrueckii* and *L. casei* using FASTA files that have been retrieved from the UniProtKB database has been reported by us.^14^ The alignment of the four domains of the ECF transporter from both strains (FolT, EcfT, EcfA1, and EcfA2) revealed some differences in the amino acid sequence of the ECF transporter between *L. delbrueckii* and *L. casei*, but with very high sequence identity.

The exploration around part A (**25**–**33**, Table S3) of molecule **1** supports the notion of the key hydrogen-bond interactions already revealed previously.^13^ Replacing the hydroxyl group in **1** by an amine (**25**) resulted in a marginal increase in activity. Meanwhile, omitting it (**26**) or changing it to a methyl (**27**) led to a further decrease in activity. On the other hand, compounds **28** and **29**, in which the spacer progressively increased from one to three methylene units, induced a complete loss of potency. By analogy, esterification of the carboxylic acid (**30**), methyl etherification of the hydroxyl group (**32**), or reduction to a primary alcohol (**33**) led to inactive compounds. The synthesis is outlined in Scheme 1.

**Scheme 1:**
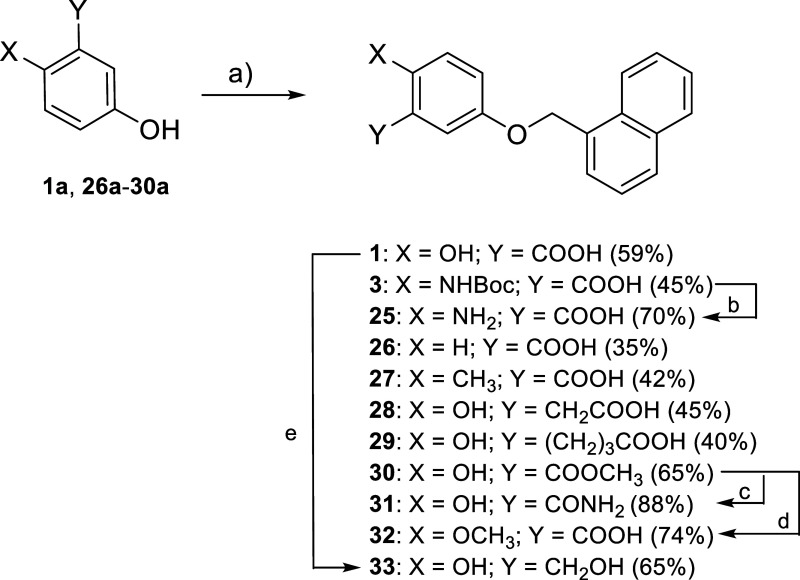
Synthesis of Derivatives **1**, **3**, and **25**–**33** ^a^

a Reagents and conditions: a) 1-(bromomethyl)­naphthalene, NaH 60% at 0 °C, DMF for 2 h, then rt overnight b) HCl (4M) in dioxane, DCM, 2 h, rt; c) NH_4_OH, dioxane, rt, 2 d.; d) 1. NaH 60%, CH_3_I, DMF, rt, 5 h (**32a**, 98%) 2. NaOH 10%, MeOH, 2 h, reflux; e) NaBH_4_, BF_3_.Et_2_O, THF, reflux, on.

Compound **1** ^13^ was obtained via an etherification reaction at position 5 of the 2,5-dihydroxybenzoic acid in the presence of 2.5 eq. of NaH.^20^ We accessed compounds **25**–**30** via an analogous route with the only exception of compound **30**, where we used K_2_CO_3_ as a base. Treatment of the methyl ester **30** with NH_4_OH afforded the corresponding amide **31** in 88% yield, while use of CH_3_I as methylating agent μM in a bacterial uptake assay usingyielded the methylated intermediate **32a**, which after basic saponification led to compound **32**. We used the combination of boron trifluoride etherate and sodium borohydride (NaBH_4_/BF_3_.Et_2_O) to reduce the carboxylic acid of **1** to the primary alcohol **33**. Cleavage of the *tert*-butyloxycarbonyl group of **3** to yield **25** took place under standard acidic conditions.

Modifications of the linker B (**34**–**43**) has been done keeping the 2-hydroxy benzoic acid “region A” and the naphthalenyl ring “region C” constant (Scheme 2 and 3, Table 1).

**Scheme 2:**
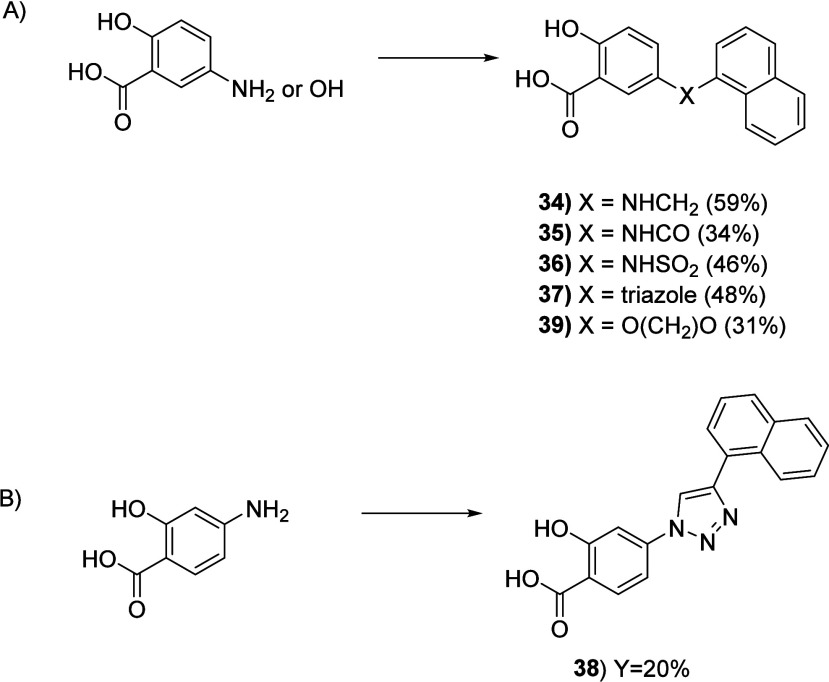
Synthesis of Derivatives **34**–**39** ^a^

a Reagents and conditions: 5-amino-2-hydroxybenzoic acid, naphthaldehyde, STAB, DMF, rt, on (**34**); 5-amino-2-hydroxybenzoic acid, 1-naphthoyl chloride, DMF, rt, 2 h (**35**); 5-amino-2-hydroxybenzoic acid, naphthalene-1-sulfonyl chloride, DMF, rt, 2 h (**36**); 5-amino-2-hydroxybenzoic acid, *t*-BuNO_2_, Me_3_SiN_3_, MeCN, rt, 2 h, followed by 1-ethynylnaphthalene, sodium ascorbate, CuSO_4_·5H_2_O, (H_2_O/*t*-BuOH 1:1), rt, 24 h (**37**); 4-amino-2-hydroxybenzoic acid, *t*-BuNO_2_, Me_3_SiN_3_, MeCN, rt, 2 h, followed by 1-ethynylnaphthalene, sodium ascorbate, CuSO_4_·5H_2_O, (H_2_O/*t*-BuOH 1:1), rt, 24 h (**38**); 2,5-dihydroxybenzoic acid, 1-(2-bromoethoxy)­naphthalene, NaH 60%, DMF, rt, on (**39**).

**Scheme 3:**
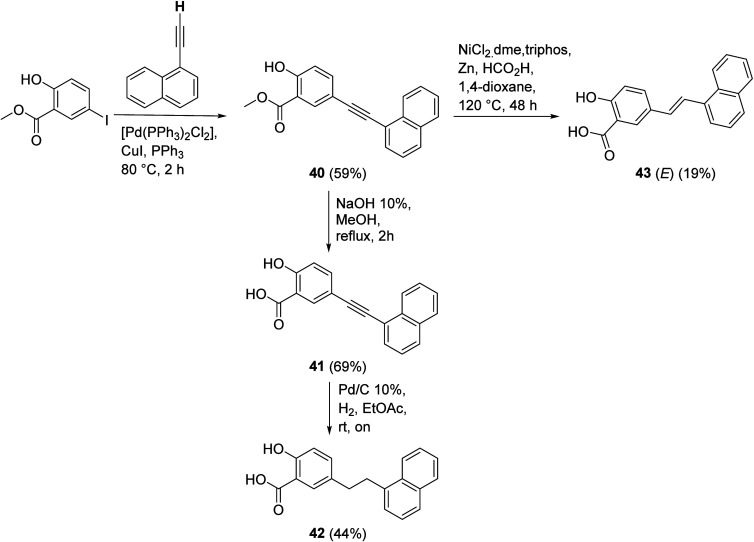
Synthesis of Derivatives **40**–**43**

**Table 1:**
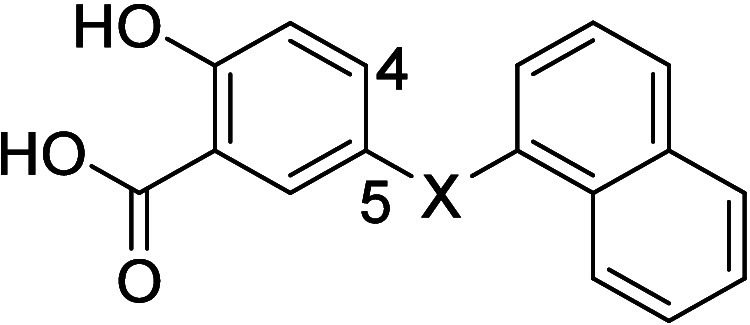
Inhibitory Potency and IC_50_ Determination of **34**–**39** and **41**–**43** in the *Lactobacillus casei* Whole-Cell Uptake Assay

compounds	X	% Inh. @ 200 μM ± S.E.M.^a^	IC_50_ (μM) ± S.E.M.^a^
**34**	NHCH_2_	36.6 ± 0.3	
**35**	NHCO	n.i.	
**36**	NHSO_2_	n.i.	
**37**	triazole	53.4 ± 9.1	
**38** ^b^	triazole	74.2 ± 3.0	
**39**	O(CH_2_)O	37.8 ± 4.6	
**41**	C≡C	100 ± 0.7	58 ± 10.8
**42**	(CH_2_)_2_	96.1 ± 1.6	41.7 ± 18.8
**43**	C=C (*E*)	85.7 ± 0.1	

a SEM derived as mean values of at least two replicates. n.i. = no inhibition when inhibition <10%.

b Triazole is connected in position 4.

Compound **34** was synthesized by using 5-amino-2-hydroxybenzoic acid as a warhead in the presence of naphthaldehyde and STAB as a reducing agent. The reaction of the 5-amino-2-hydroxybenzoic acid with 1-naphthoyl chloride and naphthalene-1-sulfonyl afforded the carboxamide **35** and sulfonamide **36**, respectively. Treatment of the aniline group of our warhead with *tert*-BuNO_2_ and Me_3_SiN_3_ led to the corresponding azide that was converted to the triazole derivative **37**. Similarly, the corresponding regioisomer at position 4 (**38**) was prepared (Scheme 2B). A Williamson ether synthesis yielded elongated polyether linker **39**. In parallel, we explored more rigid linkers (Scheme 3).

A Sonogashira cross-coupling reaction afforded the common building block **40** in 59% yield, which was then used for further derivatizations. Basic hydrolysis of the methyl ester afforded the planar alkyne **41**, which was then completely hydrogenated using Pd/C to the more flexible alkane **42**. Next, we applied a semireductive divergent hydrogenation on compound **40** that would allow us to selectively get access to the *E* and *Z*-olefin isomers. The tunable *E*/*Z* chemoselectivity was achieved by employing different nickel catalysts (NiBr_2_ for *Z*- and NiCl_2_·dme for *E*-isomer).^21^ After several attempts, however, we only managed to obtain the *E*-alkene and not the *Z*-isomer, likely due to steric hindrance. Treatment of compound **40** with NiCl_2_·dme, triphos as ligand, zinc, and HCO_2_H at 120 °C for 48 h led to full isomerization to the *E*-alkene **43**.

The bioisosteric replacement of the OCH_2_ linker to NHCH_2_ as in **34** or the elongation to a polyether linker (**39**) did not affect the inhibitory potency, while the introduction of more polar linkers like carboxamide (**35**) or sulfonamide (**36**) caused a drop in activity. Conversely, the activity is regained in the presence of triazoles **37** and **38**. Replacement of the heteroatom with a rigid alkyne (**41**, IC_50_ = 58 ± 10.8 μM) or fully saturated (**42**, IC_50_ = 41.7 ± 18.8 μM) chains boosted the activity.

Next, all the attempts to replace the naphthalenyl moiety with more soluble and drug-like aromatic rings (**44**–**56**) did not produce any boost in activity (Scheme 4, Table S4). This subset of derivatives included simplification of the naphthalenyl ring (**44**–**46**), two phenyl rings bridged by various atoms and spacers (**47**–**51**) or fused aromatic bicyclic compounds (**52**–**56**). We rationalize this lack of activity by suggesting that the limited permeability of more polar compounds to cross the phospholipid membrane could hamper the activity. The synthesis followed a reductive amination (Scheme 4 and Scheme S1).

**Scheme 4:**
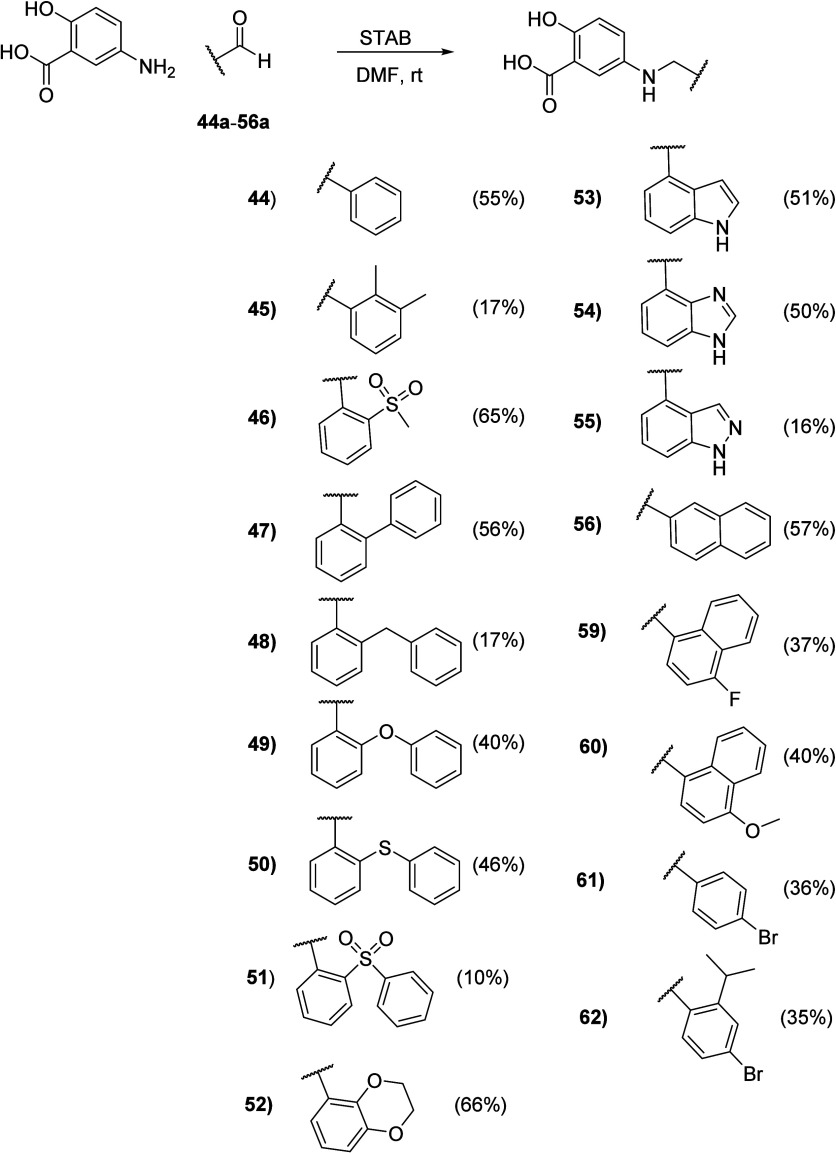
Synthesis of Derivatives **44**–**56** and **59**–**62**

To improve the potency of our series, we thought to further expand the SAR around our early optimized hit compound **4** by a series of derivatives **58**–**65** (Table 2). Initially, we made the bioisostere **58** (Scheme S2), which, as expected, showed a comparable potency to **4**. Among the synthesized compounds, we found that replacing the 4-bromo on the naphthalenyl ring with a fluorine (**59**) or a methoxy (**60**) group did not improve the potency. Interestingly, we found that keeping the bromine constant in position 4 but rebuilding the naphthalene ring with a less rigid and lipophilic group led to compound **62** with a percentage of inhibition comparable to its naphthalenyl analogue (**62**, % inh. @ 200 μM = 80.2 ± 6.6 vs **58**, % inh. @ 200 μM = 89.0 ± 2.7). Rigidifying the linker with a triazole group (**63**–**65**) further improved the activity (**61** vs **63**), with the 4-triazole (**65**) being more potent than the 5-triazole (**64**). The synthesis is depicted in Scheme S3.

**Table 2:**
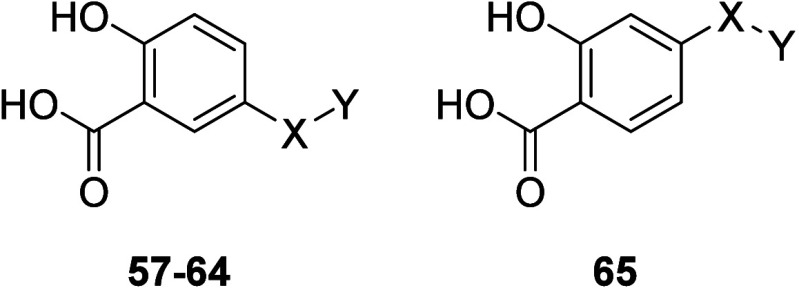
Inhibitory Potency and IC_50_ Determination of **58**–**65** in the *Lactobacillus casei* Whole-Cell Uptake Assay

cmpd	X	Y	% Inh. at 200 μM ± S.E.M.^a^	IC_50_ (μM) ± S.E.M.^a^
**4**	–OCH_2_	4-bromo-1-naphthyl	91.0 ± 3.7	59 ± 29.5
**58**	NHCH_2_	4-bromo-1-naphthyl	89 ± 2.7	
**59**	NHCH_2_	4-fluoro-1-naphthyl	30.2 ± 20.8	
**60**	NHCH_2_	4-methoxy-1-naphthyl	43 ± 9.4	
**61**	NHCH_2_	4-bromo-1-phenyl	23.7 ± 2.7	
**62**	NHCH_2_	4-bromo-2-isopropyl-phenyl	80.2 ± 6.6	
**63**	triazole	4-bromo-1-phenyl	45.4 ± 5.3	
**64**	triazole	3,5-dichlorophenyl	100 ± 0.6	85 ± 4.8
**65**	triazole	3,5-dichlorophenyl	100 ± 0.7	47.4 ± 10.1

a SEM derived as mean values of at least two replicates.

Finally, we merged the best features identified in our SAR study in compounds **67** and **68** via a Williamson ether synthesis (Scheme 5). As we observed on compound **3** that a Boc group is beneficial for the activity, we made a hybrid molecule **68** bearing an NHBoc and a 3,5-bis *tert*-butyl group in regions A and C, respectively. Consequently, cleavage of the Boc group in **68** liberated the free aniline **68a**, which, after reaction with mesyl chloride and acetyl chloride, yielded compounds **69** and **70**, respectively.

**Scheme 5:**
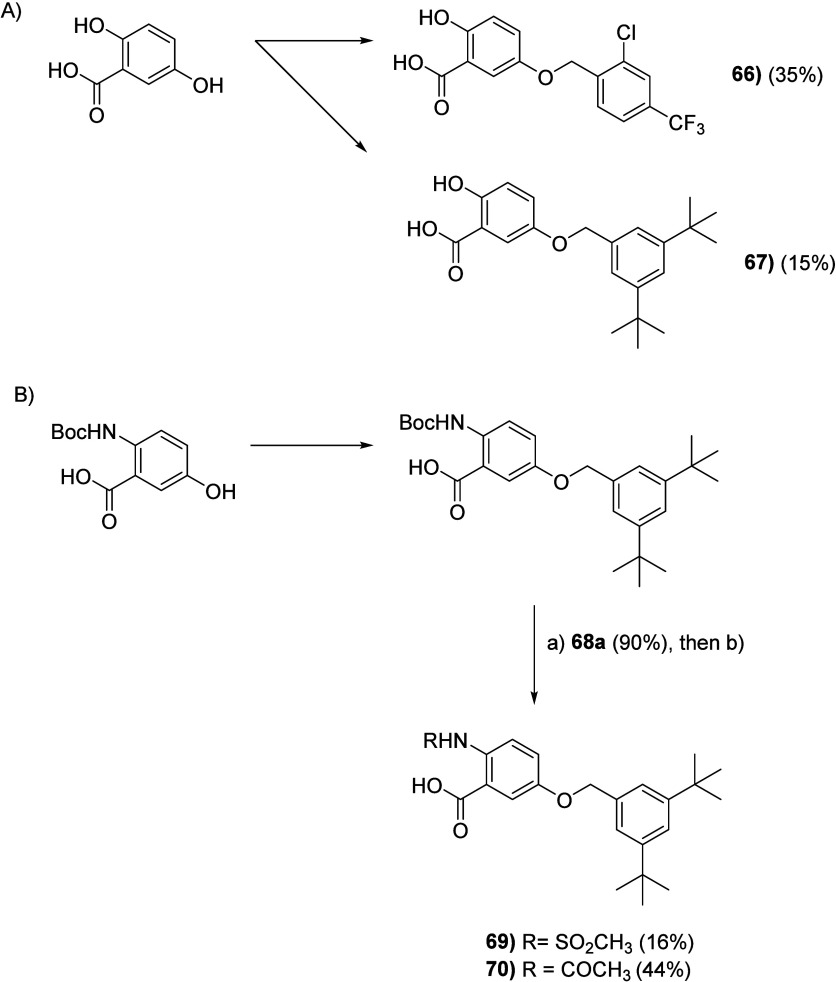
Synthesis of **66**–**70**.

a Reagents and conditions: (A) 1-(bromomethyl)-2-chloro-4-(trifluoromethyl)­benzene (for **66**), 1-(bromomethyl)-3,5-di-*tert*-butylbenzene (for **67**), NaH, DMF, rt, on; (B) 1-(bromomethyl)-3,5-di-*tert*-butylbenzene, NaH 60%, DMF, rt, on; (a) TFA in DCM to get **68a**; (b) mesyl chloride (for **69**), acetyl chloride (for **70**), pyridine, DCM, rt, 24 h.

Encouragingly, compound **67** exhibited an IC_50_ of 16.1 ± 3.8 μM. Based on the observation that a sterically bulky carbamate moiety is well-tolerated as demonstrated in compound **3,** the hybrid molecule **68** was designed to combine features of **67** and **3**. Compound **68** displayed an IC_50_ value of 5.7 ± 4.8 μM, making it the most potent compound of this series (Table 3).

**Table 3:**
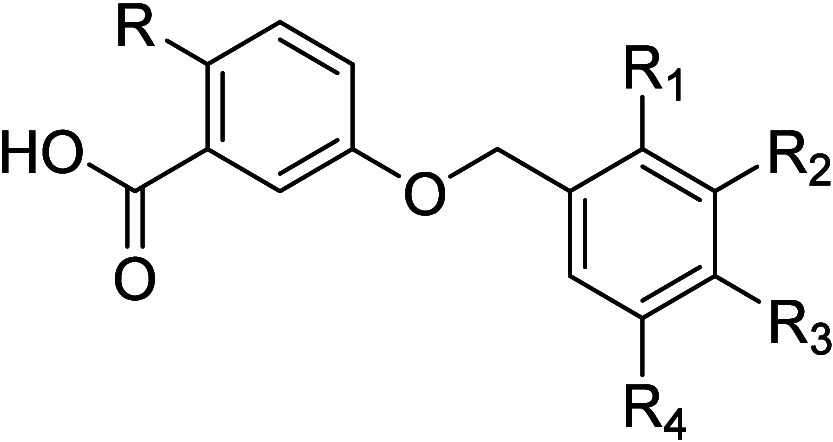
Inhibitory Potency and IC_50_ Determination of **66**–**70** in the *Lactobacillus casei* Whole-Cell Assay

compounds	R	R_1_	R_2_	R_3_	R_4_	% Inh. at 200 μM ± S.E.M.^a^	IC_50_ (μM) ± S.E.M.^a^
**66**	OH	Cl	H	CF_3_	H	70.2 ± 6.5	80 ± 19.4
**67**	OH	H	C(CH_3_)_3_	H	C(CH_3_)_3_	100 ± 0.6	16.1 ± 3.8
**68**	NHBoc	H	C(CH_3_)_3_	H	C(CH_3_)_3_	99.4 ± 1	5.7 ± 4.8
**69**	NHSO_2_CH_3_	H	C(CH_3_)_3_	H	C(CH_3_)_3_	13.3 ± 6.7	n.d.
**70**	NHCOCH_3_	H	C(CH_3_)_3_	H	C(CH_3_)_3_	97.1 ± 8.4	29.4 ± 12.0

a SEM derived as mean values of at least two replicates.

### Docking Studies

To gain insights into the binding mode of the new derivatives, we built on our previous findings from MD simulations, which identified pocket P9 of ECF-FolT2—located at the interface between EcfT and the S-component as the preferred binding site of compound **1** (Figure 4A).^13^ We subsequently docked compounds **67** and **68** into the same pocket derived from the MD simulations. As shown in Figure 4B and 4C, both compounds retained the key hydrogen bond with Lys102 while additionally establishing a hydrogen bond with Ser173. Notably, the bulkier *tert*-butyl substituents extend into the hydrophobic groove of the pocket, thereby enhancing hydrophobic interactions. The overlay of all three compounds (Figure 4D) shows a similar binding orientation of the core scaffold, while also demonstrating how substituents can modulate the hydrogen-bonding network and expand hydrophobic interactions.

**Figure 4:**
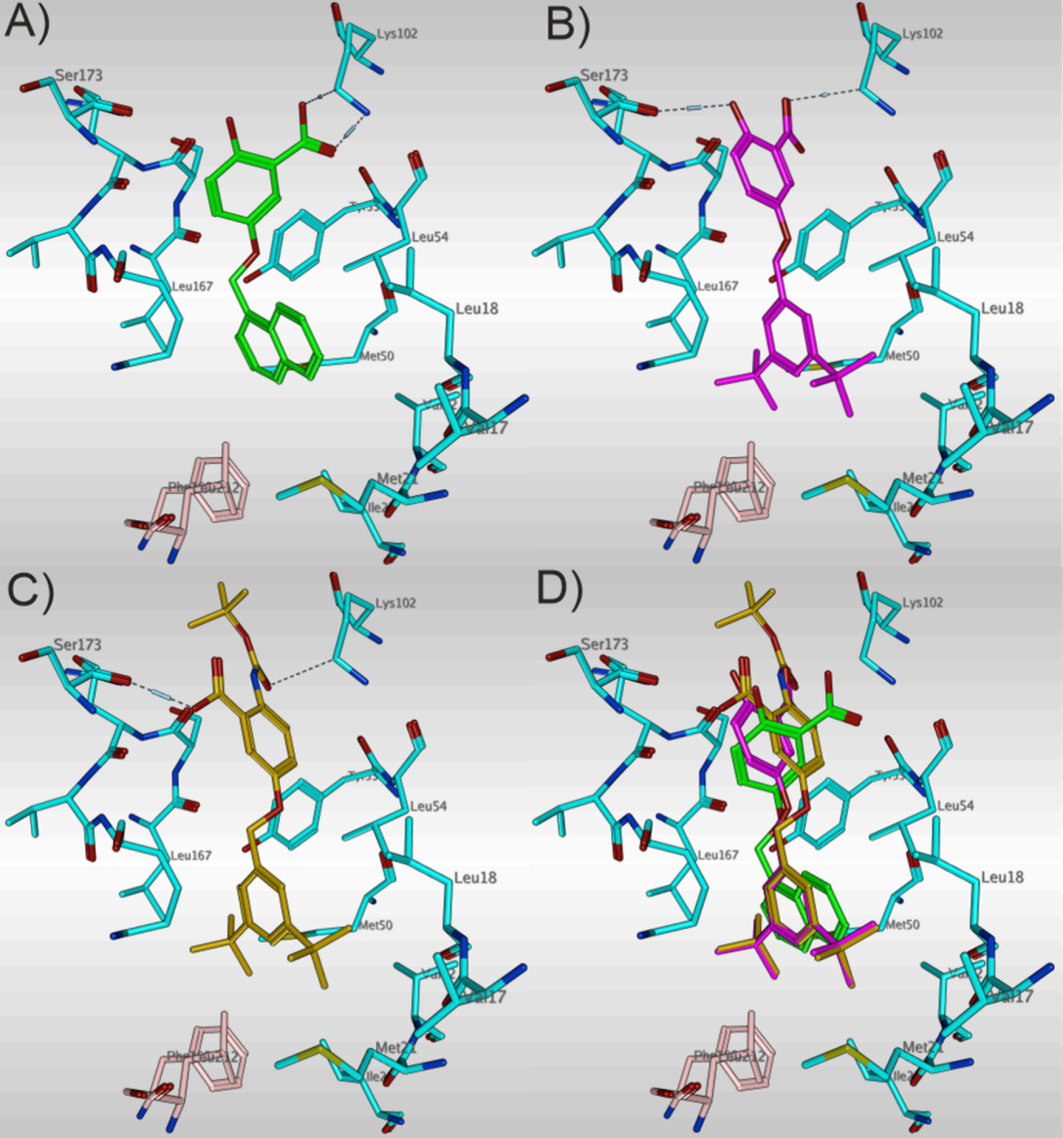
Docking pose of compounds **67** and **68** into P9 pocket of ECF-FolT2 (PDB ID: 5JSZ). (A) Pose of compound **1** (green) obtained from molecular dynamics simulations.^13^ (B) Docked pose of compound **67** (magenta). (C) Docked pose of compound **68** (gold). (D) Overlay of all three compounds, showing their similar orientation and the *tert*-butyl substituents extending into the hydrophobic groove. Protein residues of the S-component are shown in cyan and those of EcfT in pink; hydrogen bonds are indicated by dashed lines.

### ECF Transporter Complex Is Conserved in *Streptococcus pneumoniae*

The three genes encoding the ECF transporter complex in *S. pneumoniae* (*cbiO1*, *cbiO2*, and *cbiQ*) are universally conserved across 27 pneumococcal blood isolates that span the global pneumococcal pangenome, with a minimum nucleotide sequence identity of 94% (average 99.8% for *cbiO1*, 99.6% for *cbiO2*, 99.8% for *cbiQ*). Thus, at the amino acid sequence, the identity is even higher.

### Importance of ECF Transporter for Survival

Experimental knockdown of any gene in the ECF operon (*cbiO1O2Q*) by CRISPR interference led to decreased fitness of these pneumococcal strains. Elements of the ECF operon were universally important for survival: *in vitro*, in an *in vivo* murine infection model, as well as in human specimens that are involved in invasive pneumococcal infections during *ex vivo* culture (Table 4). Table 4 displays the log2 fold changes in *S. pneumoniae* cell counts (adjusted *p*-value or fraction of donors in which depletion was statistically significant) when a gene of the ECF operon is knocked down, compared to wild-type expression after challenge under different conditions. A fold change of 0 indicates that expression of the *cbi* gene is not relevant for survival of the pneumococcus, whereas depletion (a negative fold change) indicates that the bacterium needs expression of the gene for surviving that challenge condition. The size of the observed negative fold changes and the associated p-values both indicate that the *cbi* genes were highly important for survival of *S. pneumoniae* in all studied infection models. Data were selected from genome-wide CRISPR interference screens in *S. pneumoniae* .

**Table 4:** Importance of ECF Transporter for Pneumococcal Survival by CRISPRi-seq

		gene knockdown		
		*cbiO1*	*cbiO2*	*cbiQ*
challenge condition (# donors)	laboratory medium	–9.6 (5 × 10^–13^)	–6.7 (7 × 10^–23^)	–9.4 (8 × 10^–16^)
	mouse pneumonia model	–8.3 (4 × 10^–102^)	–5.5 (2 × 10^–58^)	–8.1 (4 × 10^–138^)
	mouse sepsis model	–8.1 (2 × 10^–4^)	–2.6 (ns)	–8.1 (1 × 10^–5^)
	human serum (1)	–3.7 (1 × 10^–20^)	–3.0 (2 × 10^–23^)	–3.4 (5 × 10^–13^)
	human cerebrospinal fluid (4)	–4.8 (3/4)	–3.2 (2/4)	–5.2 (4/4)
	human lung pleural fluid (3)	–4.1 (3/3)	–1.5 (1/3)	–4.1 (3/3)

### Drug Activity in Relation to Availability of Target in *Streptococcus pneumoniae*

We tested how the availability of the presumed target (ECF transporter) affected the activity of the compounds in killing *S. pneumoniae* . To this end, bacterial time-kill assays were performed using a D39 V pneumococcal strain in which the original copy of the *cbiO1O2Q* operon was removed from the bacterial genome. Instead, in an ectopic locus, a complementary *cbiO1O2Q* operon was placed, which is under control of an IPTG-titratable promoter, such that the expression of the ECF transporter can be controlled. Without antimicrobial treatment, both under- and overexpression of the *cbiO1O2Q* operon led to a slight growth defect compared to wild-type growth (Figure 5). Under treatment using the most potent compounds from this study (**67** and **68**), some expression of the *cbiO1O2Q* operon was essential to sustaining bacterial growth. When overexpression of the *cbiO1O2Q* operon was initiated under treatment with compounds **67** and **68**, this could partially recover growth.

**Figure 5:**
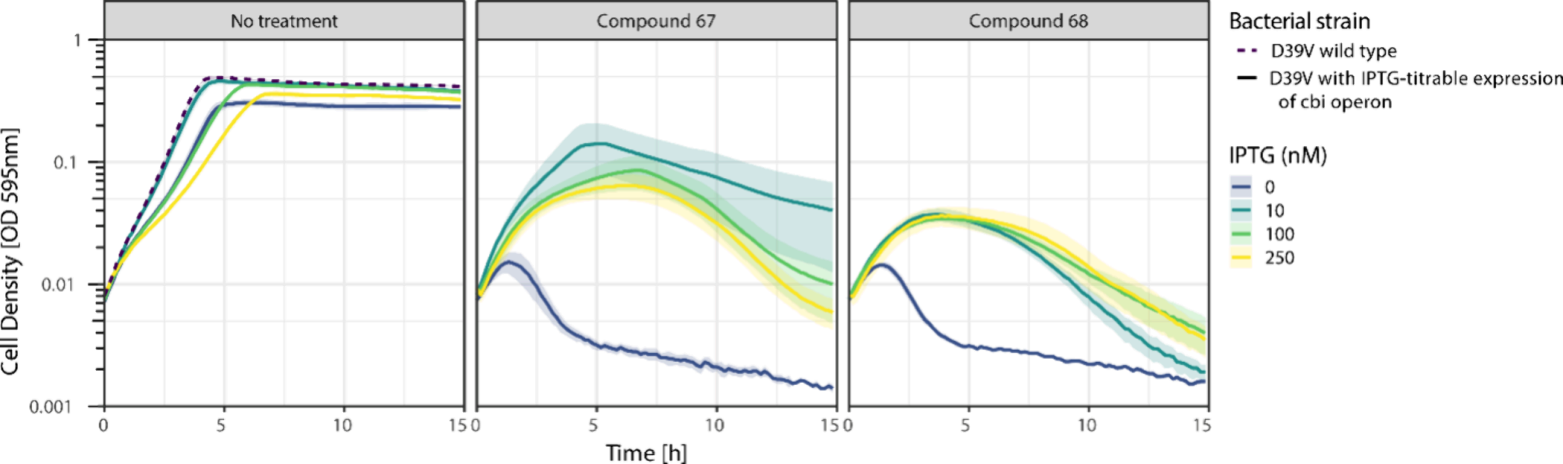
Bacterial growth curves demonstrating the activity of compound **67** and **68** in relation to the expression of presumed target: the energy-coupling factor (ECF) transporter in *Streptococcus pneumoniae* . Each line represents four replicate measurements and their colors indicate the relative availability of the presumed target: the *cbiO1O2Q*-encoded ECF transporter. The treatment dose of compounds **67** and **68** was at 1× their MIC. IPTG: Isopropyl β-d-1-thiogalactopyranoside. Expression of the ECF transporter was zero when the pneumococcal cells were exposed to the compound, and only then, induction using IPTG was initiated.

These observations support the hypothesis that compounds **67** and **68** directly bind to the pneumococcal *cbiO1O2Q*-encoded ECF transporter. Since cells were depleted for CbiO in this experiment for a few hours, it has been assumed that CbiO protein levels are low but not zero; therefore, we interpret these results that these depleted cells are hypersensitive to the compounds. Upon overproduction of CbiO (with IPTG), more targets become available, making the compounds relatively less active on bacterial growth. However, we also cannot fully exclude the possibility that compounds **67** and **68** have additional targets in cells. While being exposed to an ECF transporter inhibitor, no resistant pneumococcal variants were identified in the mutation screens. This lack of resistance development is a great asset for future antibiotics.

To advance our characterization, we focused on the most potent inhibitors identified (compounds **67** and **68**) and included our early optimized hit **4** ^19^ to enable a direct comparison.

### Proteoliposome Assay

As a next step, we tested compounds **4**, **67**, and **68** in the proteoliposome transport-activity assay using ECF-FolT2 and ECF-PanT from *L. delbrueckii*.^12^ The bacterium *L. delbrueckii* has eight different S-components that share the same ECF module.^8^ Compounds **4**, **67**, and **68** showed an IC_50_ slightly higher, but still in the low-micromolar range, in particular compound **4** has an IC_50_ of 69 μM, while **67** and **68** showed an IC_50_ of 55 ± 10 μM and 37.2 ± 5.5 μM, respectively (Supporting Information Section 4.2).

### Antibacterial Activity and HepG2 Toxicity of Selected Frontrunner Molecules

We moved one step forward and investigated the cytotoxic effects on HepG2 (human liver cancer cells) of compounds **4**, **67**, and **68**. While **67** has an IC_50_ = 75.0 ± 22.1 μM, compounds **4** and **68** did not show any considerable toxicity (HepG2: IC_50_ > 100 μM, Section 4.3, Supporting Information). These results prompted us to measure the effect of the compounds on the human pathogens *Enterococcus* spp. and *S. pneumoniae* . These bacteria are auxotrophic for pantothenate and biotin and depend on ECF transporters for their uptake. Therefore, we predicted that inhibition of the ECF transporter would reduce viability. By studying compounds **4**, **67**, and **68**, which are structurally different will allow us to determine whether activity on the target is also correlated with activity in the cell. Consistently, compounds **67** and **68** inhibited the growth of various *S. pneumoniae* strains and *Enterococcus* spp. (Table 5) with a minimum inhibitory concentration (MIC) in the micromolar range. By tendency, the improvement with respect to on-target activity of **67** and **68** compared to **4** translates into higher antibacterial potency and no loss of potency in drug-resistant species. Notably, only compounds **67** and **68** exhibit a broader spectrum of activity.

**Table 5:** Minimum Inhibitory Concentrations (MICs) of ECF Transporters Inhibitors for *Streptococcus pneumoniae* *,* *Enterococcus faecium* and *E. faecalis* Estimated by Broth Microdilution

	MIC [μg/mL]		
strain	4	67	68
*S. pneumoniae* DSM-20566	3–12 ^19^	3–6	1–2
*S. pneumoniae* DSM-11865^a^	128	1.5–3	3–6
*S. pneumoniae* D39 V	16	1	0.7
*E. faecalis* DSM-20478	>128	11	4
*E. faecium* DSM-20477	>128	3–6	4
*E. faecium* DSM-17050^b^	24–48	11	4

a PRSP: penicillin-resistant *S. pneumoniae* .

b VRE: vancomycin-resistant *Enterococcus*.

### Time-Kill Evaluation on *Streptococcus pneumoniae* DSM-20566

Encouraged by the observed antibacterial effects, we performed a time-kill analysis to establish the rate at which the *S. pneumoniae* DSM-20566 is killed by an ECF transporter inhibitor. To do so, we chose as a representative ECF transporter inhibitor, compound **67**. When treated with 8× MIC, no colonies were recovered (LoD: 10^2^ CFU/mL), and treatment at 2×/4× MIC resulted in an initial static effect (0–2 h), followed by a fast decline (cidal) in CFU/mL with colony numbers dropping below the LoD as early as 6 h after treatment (Figure 6).

**Figure 6:**
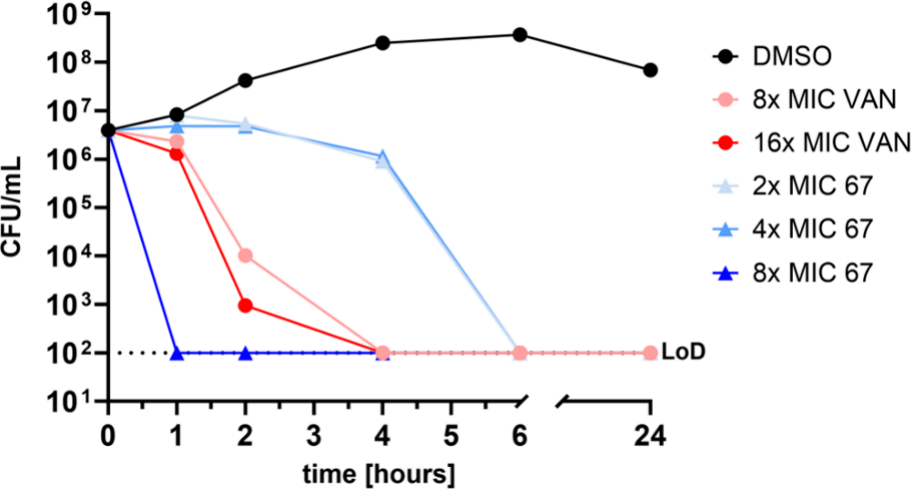
Time-kill curve (TKC) of *Streptococcus pneumoniae* DSMg20566 treated with **67** at 2×, 4×, and 8× MIC. DMSO served as solvent/growth control (1%, *v/v*) VAN at 8×, 16× MIC was used as reference antibiotic. LoD: limit of detection (10^2^ CFU/mL).

### *In Vivo* Pharmacokinetic Profiling

Next, we wanted to explore the *in vivo* pharmacokinetic (PK) profile of this chemical class exemplified by compounds **4**, **67**, and **68**. The outcome of this work is also important to guide future medicinal chemistry optimization and to ensure that subsequent rounds of improvement in potency will also translate into a lead candidate with optimum PK behavior. The *in vitro* ADME profiling is depicted in Section 6.0 of the Supporting Information. First, we embarked on an *in vivo* PK study using the intravenous (IV) route at 10 mg/kg for **4** to understand the exposure at 100% bioavailability and derive PK parameters, such as half-life, clearance, and volume of distribution. We derived high initial *C*_0_ concentrations of around 16.9 μg/mL and an exposure of 5.9 μg/mL × h (Table 6, Figure 7a). Moreover, we observed a moderate-to-low volume of distribution of around 2.4 L/kg suggesting good distribution into tissue with a moderate-to-low plasma clearance of 28 mL/min/kg and an acceptable half-life of around 1 h (Table 6). Next, we ran a cassette PK study with **67** and **68** at 1 mg/kg IV. Both compounds exhibited a lower half-life of only around 0.7–0.75 h. Moreover, **67** had a high plasma clearance with 92.3 mL/min/kg, whereas for **68**, a moderate and slightly higher clearance compared to **4** was observed. Additionally, exposure was much lower compared to **4** (when dose-adjusting exposure) (Table 7). Although we had run several studies with **67** and **68**, we decided on **4** for further *in vivo* animal studies as it had a similar MIC profile as **67**, a similar inhibition rate at 200 μM and only a slightly higher IC_50_ (when taking standard deviation into account), but more favorable PK properties, and we then conducted an *in vivo* PK study using the peroral (PO) route at 10 mg/kg (Figure 7b). We observed a good exposure as well as a high *C*_max_ of around 3.6 μg/mL and a *T*_max_ of 0.5 h. Additionally, **4** exhibited a very good peroral bioavailability of around 82% (Table 6). To prepare for an *in vivo* animal infection model, plasma concentrations as a surrogate for lung concentrations were not yet sufficient, as we planned to ensure total plasma concentrations above the MIC value determined *in vitro* to increase the chances of observing efficacy. Therefore, we conducted a PK study at a 10-fold higher dose at 100 mg/kg PO. We detected a substantially higher *C*_max_ of around 16 μg/mL as well as a dose-linear increase in exposure (Table 6, Figure 7c).

**Table 6:** PK Properties of **4** Using Different PK Designs

	10 mg/kg IV	10 mg/kg PO	100 mg/kg PO
*t*_1/2_ [h]	1.00 ± 0.1		
*C*_0_ [μg/mL]	16.9 ± 4.1		
*C*_max_ [μg/mL]		3.6 ± 0.6	16.7 ± 12.8
*T*_max_ [h]		0.5 ± 0.0	1.0 ± 0.0
AUC [μg/mL × h]	5.9 ± 0.3	4.9 ± 1.0	47.2 ± 29.4
MRT [h]	0.5 ± 0.0	2.1 ± 0.4	3.5 ± 1.1
Vz_obs/F [L/kg]	2.44 ± 0.4	6.14 ± 4.3	8.0 ± 6.5
Cl_obs/F [mL/min/kg]	28.0 ± 1.5	33.1 ± 5.7	38.0 ± 20.3

**Figure 7:**
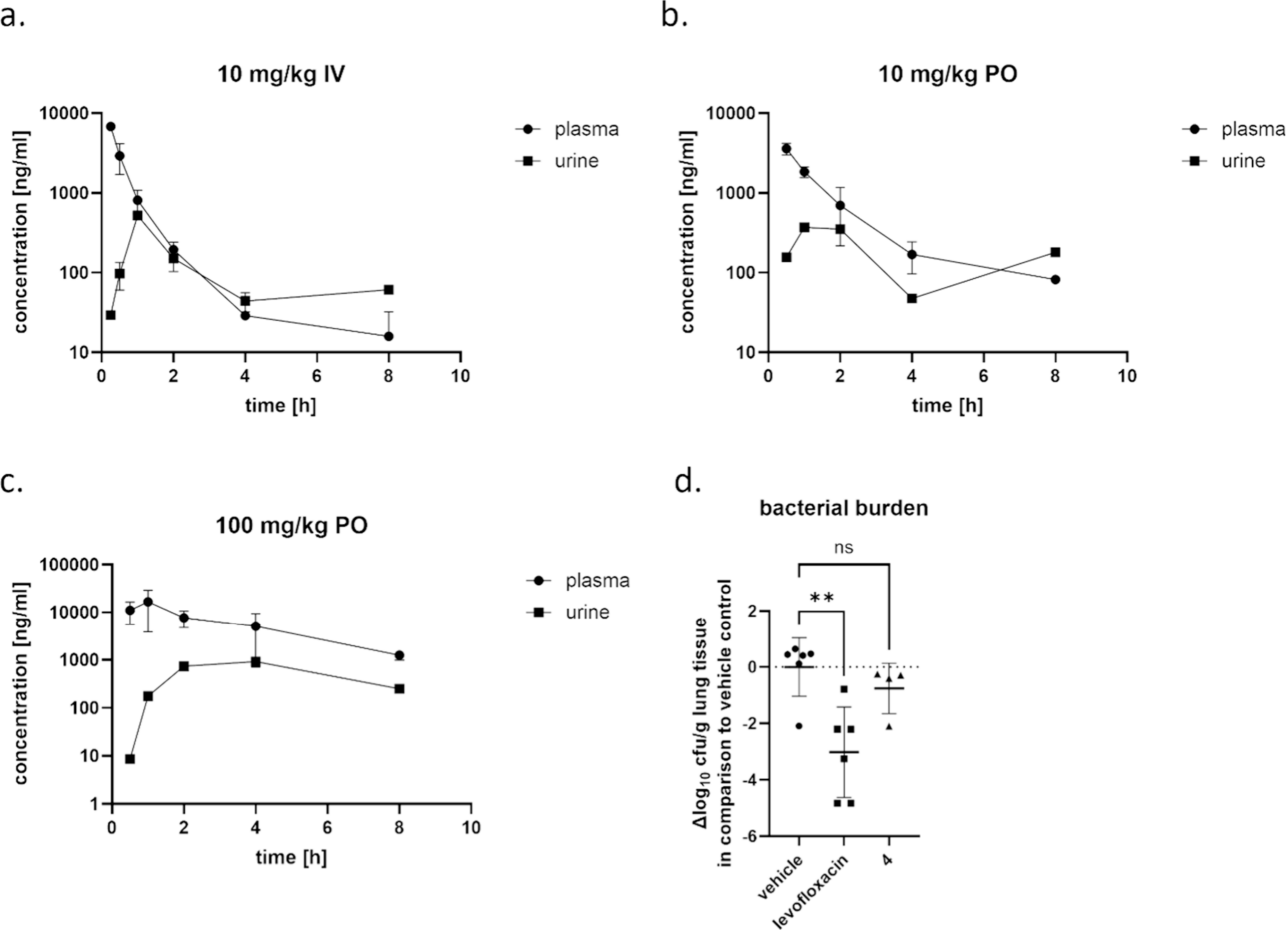
*In vivo* profiling compound **4**. Pharmacokinetic (PK) profiles in plasma (circles) and urine (squares) of compound **4** after administration of 10 mg/kg IV (a), 10 mg/kg PO (b), and 100 mg/kg PO (c) show that **4** is renally excreted and has good peroral bioavailability and a sustained plasma profile after 100 mg/kg PO. In a neutropenic lung-infection model with *S. pneumoniae* compound **4** shows a one log_10_-unit bacterial burden reduction compared to the vehicle-treated group, whereas levofloxacin (positive control) shows a bacterial burden reduction of around 3 log_10_-units (d). Statistical testing was performed using a Kruskal–Wallis Test. **: *p* < 0.01; ns: not significant.

**Table 7:** Comparison of Pharmacokinetic Properties of **4** (Dose-Adjusted Calculation), **67**, and **68** after IV Dosing

	**4** (dose adjusted to 1 mg/kg IV)	**67** 1 mg/kg IV	**68** 1 mg/kg IV
*t*_1/2_ [h]	1.0	0.79	0.75
C_0_ [μg/mL]	0.17	0.18	0.32
AUC [μg/mL × h]	0.59	0.18	0.28
Vz_obs [L/kg]	2.44	6.34	2.35
Cl_obs [mL/min/kg]	28.0	92.3	36.3

However, we also observed that levels dropped below 4 μg/mL shortly after 4 hours post-administration. Therefore, we decided that for the *in vivo* efficacy trial, a three times daily administration at 100 mg/kg PO was necessary to achieve sufficient compound concentrations. Next, we conducted a neutropenic lung infection model with *S. pneumoniae* .^23^ To avoid that compound concentrations drop below 4 μg/mL for a longer period, we ended the experiment already at 18 h. We administered **4** at 100 mg/kg PO at time points *t* = 2, 6, and 10 h postinfection. Similarly, we administered vehicle only or levofloxacin at 100 mg/kg IP at 2 h postinfection. We observed a bacterial-load reduction by 3 log_10_ units in cfu/g tissue for the positive control group, *i.e*., levofloxacin, compared to vehicle, and a moderate reduction of around 1 log_10_ unit in cfu/g tissue for **4** (Figure 7d). This first result shows that targeting the ECF transporter holds great potential. Future studies need to focus on enhancing the efficacy of the compounds used. These first findings are promising and provide a strong foundation for further optimization to achieve more significant improvements.

## Conclusions

The present study describes a systematic SAR investigation of a recently disclosed class of ECF-transporter inhibitors featuring the salicylic acid scaffold. We present various synthetic approaches to obtain diversified analogues in high yield and with good feasibility. We successfully managed to identify the key structural determinants to achieve inhibition and simplify the naphthalene ring of the initial hit **1**. Furthermore, for the first time, we demonstrated by CRISPR interference that the ECF operon (*cbiO1O2Q*) is conserved and universally important for survival of **S. pneumoniae* in vitro* and in an *in vivo* murine infection model. The antimicrobial activity of our best compounds in the series **67** and **68** is dependent on bacterial expression of the ECF transporter, giving a first indication of target engagement of the ECF transporter in *S. pneumoniae* .

The PK studies with **4**, **67**, and **68** revealed that **4** had the best PK properties, and we therefore selected it for *in vivo* proof-of-concept studies. In a neutropenic lung infection model, **4** reduced the bacterial burden and exhibited excellent peroral bioavailability. These results provide convincing evidence that the ECF transporters are a novel druggable target in the fight against antimicrobial resistance and that the disclosed compounds can act as tool compounds to unravel the biological role of ECF transporters. Additionally, our study reveals that these compounds harbor good initial PK properties and the potential for peroral bioavailability, providing a first translation of the efficacy of our ECF inhibitor. Through our in-depth investigations, we confirmed that the bis-*tert*-butyl substituent boosts the activity, but the naphthalenyl ring is better in terms of bioavailability; therefore, this work sets the stage for future optimization with the goal to balance PK and potency.

## Experimental Section

### Bacterial Strains

*S. pneumoniae* is a human pathogen. In *S. pneumoniae* , three genes encode for the energy-coupling factor (ECF) ABC-transporter complex (*cbiO1*, *cbiO2*, and *cbiQ*). The conservedness of these three gene sequences was tested in 27 selected pneumococcal draft genomes from the PBCN study in the Netherlands^22^ that represent the global pangenome, by nucleotide BLAST alignment. Pneumococcal strain D39 V wild-type (VL1) was used to determine the minimum inhibitory concentration (MIC) of an ECF transporter inhibitor. To test whether the *cbiO1O2Q* system was involved in pneumococcal killing by an ECF inhibitor, we deleted the original operon from the D39 V genome and complemented it by an ectopic copy to put its expression under control of an IPTG-inducible promoter (VL5121:D39 V;*Prs1*::LacI;*cbiQO1O2*::ery,ZIP::Plac-*cbiQO1O2*). The importance of the *cbiO1O2Q* system for pneumococcal survival was tested using CRISPR interference sequencing (CRISPRi-seq) libraries in strain D39 V (IPTG-inducible^23^ and tetracycline inducible^24^) and a clinical blood isolate PBCN0272 (unpublished data, Veening et al.). These libraries enable an estimation of the fitness effect of silencing *cbiO1*, *cbiO2*, and *cbiQ* under relevant conditions.

### Target Conservedness

To assess the presence and homology of the sequence elements *cbiO1*, *cbiO2*, and *cbiQ* of pneumococcal strain D39 V across the pneumococcal population, their nucleotide sequences were aligned to draft genomes of 27 pneumococcal strains isolated from human blood cultures (listed with serotypes and ENA accession IDs in section 5.0 of the Supporting Information).

### CRISPRi-seq Screen

Genome-wide fitness data were generated previously using the D39 V CRISPRi-seq library in the following challenge conditions: C+Y laboratory medium, a mouse pneumonia model,^24^ and a mouse sepsis model via intraperitoneal injection of bacteria (unpublished data, Veening et al.).^25^ In addition, genome-wide fitness was screened by culturing the PBCN0272 CRISPRi-seq library in human serum, cerebrospinal fluid, and pleural fluid (unpublished data, Veening et al.).^25^ Significant depletion of strains in which we silenced a gene of the *cbiO1O2Q* system indicated a fitness defect for those strains and, as such, the importance of the ECF transporter for survival in the tested *in vitro*, *in vivo*, and *ex vivo* conditions. Methods for genome-wide fitness quantification have been detailed in a previous publication.^25^

### Minimum Inhibitory Concentration (MIC) and Expression of the ECF System

Pneumococcal growth curves were observed in triplicate in 300 μL of fresh Tryptic Soy broth (TSB) in a sterile Nunc MicroWell 96-Well Microplate (Thermo Fisher; Cat no 269787) at 37 °C. Cell density was measured by optical density at 595 nm every 10 min over 20 h in a Tecan Spark microplate reader. The MIC was defined as the lowest concentration of the ECF inhibitor at which OD_595_ < 0.1 (∼0.5 log reduction) after 5 h of growth. Growth curves were also generated under controlled expression of the *cbiO1O2Q* operon at an ECF inhibitor concentration of double the MIC.

### Time-Kill Kinetics in *Streptococcus pneumoniae*

To study the time-kill kinetics of **67** in *S. pneumoniae* DSM-20566, an overnight bacterial culture was prepared from single colonies grown on blood agar in sterile, fresh TSB. The culture was then placed in an incubator at 37 °C with 5% CO_2_. The overnight bacterial culture was diluted 1:100 in fresh sterile medium (TSB). After 3–4 h of incubation, OD_600_ was determined, after which the initial inoculum was adjusted to 5 × 10^6^ colony-forming units (CFU/mL). Compound **67** was dissolved in DMSO (Sigma), and the concentrations were adjusted to achieve 1% (*v/v*) DMSO for all samples in the assay. Time-kill kinetics were determined at 2×, 4×, and 8× the MIC, and at 8× and 16× the MIC of vancomycin (VAN) as a reference antibiotic. As growth control, *S. pneumoniae* was treated with 1% (*v/v*) DMSO. The treated bacterial culture was incubated at 37 °C with 5% CO_2_. At designated time points (0, 1, 2, 4, 6, and 24 h), samples were taken, and CFU/mL was determined by plating the culture on nonselective blood agar in serial dilutions. The plates were then incubated in a static incubator at 37 °C with 5% CO_2_ for 24 h. After incubation, the colonies were counted, the CFU/mL determined, and the results plotted as a time-kill curve (TKC).

### Mutation Screen

*S. pneumoniae* D39 V was inoculated on tryptic soy agar that contained the ECF inhibitor at 1× and 2× MIC and incubated at 37 °C for 5 days and 5% CO_2_. If potentially resistant pneumococcal colonies emerged, these were further cultivated in TSB that contained the same concentration of the ECF inhibitor and Illumina whole-genome sequenced to identify genetic mutations as compared to the wild-type strain.

### Animals

The animal studies were conducted in accordance with the recommendations of the European Community (Directive 2010/63/EU, January 1, 2013). All animal procedures were performed in strict accordance with the German regulations of the Society for Laboratory Animal Science (GV-SOLAS) and the European Health Law of the Federation of Laboratory Animal Science Associations (FELASA).

The ethical approval for this study was obtained from the ethical board of the Niedersächsisches Landesamt für Verbraucherschutz and Lebensmittelsicherheit, Oldenburg, Germany. The related approval numbers have been shared confidentially with the journal. Due to legal and institutional restrictions, these numbers cannot be disclosed publicly. Any inquiries or concerns regarding this approval may be directed to the corresponding author or the institutional ombuds group at Helmholtz Centre for Infection Research, Braunschweig, Germany.

### Pharmacokinetic (PK) Studies

For PK experiments, outbred male CD-1 mice (Charles River, Netherlands), 4 weeks old, were used. Compounds **4**, **67**, and **68** were dissolved in 20% DMSO, 60% PEG400, and 20% sodium citrate solution (25 mg/mL). Compound **4** was administered at 10 mg/kg IV, 10 mg/kg PO, and 100 mg/kg PO. Per the PK study, *n* = 3 animals were used. Compounds **67** and **68** were administered at 1 mg/kg IV as a cassette format with *n* = 2 animals per study. For the PK study with 10 mg/kg IV, the following time points were used: *t* = 0.25, 0.5, 1, 2, 4, 8, and 24 h. For the 10 mg/kg PO and 100 mg/kg PO PK studies, the following time points were used: *t* = 0.5, 1, 2, 4, 8, and 24 h. At time points *t* = 0.25–8 h, samples were collected using microsampling, whereas time points at *t* = 24 h were terminal. For the PK study with **67** and **68**, the following time points were used: *t* = 0.25, 0.5, 1, 3, and 5 h. At time points *t* = 0.25–3 h, samples were collected using microsampling, whereas the time point at *t* = 5 h was terminal. Whole blood was collected into Eppendorf tubes coated with 0.5 M EDTA and immediately spun down at 13,000 rpm for 10 min at 4 °C. The plasma was transferred into a new Eppendorf tube and then stored at −80 °C until analysis. Moreover, spontaneous urine was also collected. All PK plasma samples were analyzed via HPLC–MS/MS using an Agilent 1290 Infinity II HPLC system and coupled to an AB Sciex QTrap6500plus mass spectrometer. First, a calibration curve was prepared by spiking different concentrations of **4** or **67** and **68** into the respective matrix (mouse plasma (pooled, from CD-1 mice) for plasma samples and urine for urine samples). Caffeine was used as an internal standard. In addition, quality control samples (QCs) were prepared for **4** or **67** and **68** with the respective matrix. The following extraction procedure was used: 7.5 μL of a plasma sample (calibration samples, QCs, or PK samples) was extracted with 37.5 μL of a 1:1 mixture of methanol and acetonitrile containing 12.5 ng/mL of caffeine as an internal standard for 5 min at 2000 rpm on an Eppendorf MixMate vortex mixer. Fifteen μL of a urine sample (calibration samples, QCs or PK samples) was extracted with 15 μL water containing 0.1% formic acid and 22.5 μL of a 1:1 mixture of methanol and acetonitrile containing 12.5 ng/mL of caffeine as an internal standard for 5 min at 2000 rpm on an Eppendorf MixMate vortex mixer. Then, samples (plasma and urine) were spun down at 13,000 rpm for 10 min at 12 °C. Supernatants were transferred to standard HPLC-glass vials. HPLC conditions were as follows: column: Agilent Zorbax Eclipse Plus C18, 50 × 2.1 mm, 1.8 μm; temperature: 30 °C; injection volume: 5 μL; flow rate: 700 μL/min; solvent A: water + 0.1% formic acid; solvent B: acetonitrile + 0.1% formic acid; gradient: 99% A at 0 min and until 0.1 min, 99%–15% A from 0.1 to 2.4 min, 15%–0% A from 2.51 min until 4 min. Mass spectrometric conditions were as follows: scan type: MRM, negative and positive mode; Q1 and Q3 masses for caffeine and **4** can be found in Table S7. Peak areas of each sample and of the corresponding internal standard were analyzed using MultiQuant 3.0 software (AB Sciex). Peak areas of the respective sample were normalized to the internal standard peak area. The MS/MS pairs used for quantification are marked with a “Q” in the table; the other MS/MS pairs for the respective compound were used for qualification. Peaks of PK samples were quantified using the calibration curve. The accuracy of the calibration curve was determined using QCs independently prepared on different days. PK parameters were determined using a noncompartmental analysis with PKSolver.^26^

### Neutropenic Lung Infection Model

The neutropenic lung infection model was performed as described previously.^27^ In brief, *n* = 6 female, outbred, CD-1 mice were used per group and infected, after being rendered neutropenic, with an inoculum of 2.4 × 10^9^ CFU/mL of *S. pneumoniae* ATCC 700905 using an Aeroneb lab nebulizer. All groups were euthanized 18 h postinfection. In detail, the following groups were used: a vehicle-treated group, a positive control group receiving levofloxacin at 100 mg/kg IP at *t* = 2 h postinfection, and one treatment group receiving **4** at 100 mg/kg PO at *t* = 2, 6, and 10 h. 18 h postinfection, lungs were removed and plated in serial dilutions on blood agar plates to determine CFU counts. Plates were incubated for at least 24 h before being counted and analyzed. Samples were analyzed using GraphPad Prism version 10.4.1 using a Kruskal–Wallis test for statistical testing (statistical power α < 0.05, β < 0.2).

### Synthesis of Compounds

All compounds are >95% pure by HPLC analysis and are described in the Supporting Information.

## Supplementary Material





## Notes

Experimental procedures and compound characterization data (NMR spectra and HPLC purity), molecular docking procedure, biological data, and protocols (PDF)
Molecular formula strings (CSV)

## ##

Department of Biochemistry, Faculty of Mathematics and Natural Sciences, IPB University, 16680 Bogor, Indonesia

## Abbreviations Used

AMR: antimicrobial resistance
CFU: colony-forming units
ECF: energy-coupling factor
ECF-FolT2: folate-specific energy-coupling factor transporter
ECF-PanT: pantothenate-specific energy-coupling factor transporter
LOD: limit of detection
PRSP: penicillin-resistant *Streptococcus pneumoniae*
PK: pharmacokinetic
SBVS: structure-based virtual screening
SI: selectivity index
TKC: time-kill curve
