# Supplementary material for: Targeting the Energy-Coupling Factor Transporters: A Novel Antibiotic Drug Target in Streptococcus pneumoniae
Source: J Med Chem. 2026 Jun 12;69(12):14054–68. doi: 10.1021/acs.jmedchem.5c03638 (PMC13312444; doi:10.1021/acs.jmedchem.5c03638)
Supplement: Supplementary file 1 [file jm5c03638_si_001.pdf]

Supporting Information

Targeting the Energy-Coupling Factor (ECF) transporters: a novel antibiotic drug target in *Streptococcus pneumoniae*

Eleonora Diamanti,[1,2] Amelieke J.H. Cremers,[3] Yue Li,[4] Ioulia Exapicheidou,[1,2] Carole Baumann,[5,6] Paddy Gibson, [3] Atanaz Shams,[1,2] Louise Martin,[3] Rouven Becker,[1] Inda Setyawati,[4] Lucie Zeimetz,[1,2] Jörg Haupenthal,[1,2] Matthias Witschel,[7] Dirk J. Slotboom,[4] Jennifer Herrmann,[5,6] Katharina Rox,[8,9] Mostafa M. Hamed,[1,2] Jan-Willem Veening,[3] Anna K. H. Hirsch[*1,2,6]

[1]Helmholtz Institute for Pharmaceutical Research (HIPS)-Helmholtz Centre for Infection Research (HZI), Campus Building E 8.1, D-66123, Saarbrücken, Saarland, Germany.

[2]Saarland University, Department of Pharmacy, Campus E8.1, 66123, Saarbrücken, Saarland, Germany.

[3]Department of Fundamental Microbiology, Faculty of Biology and Medicine, University of Lausanne, CH-1015 Lausanne, Switzerland.

[4]Groningen Biomolecular Sciences and Biotechnology Institute, University of Groningen, Nijenborgh 4, 9747AG Groningen, The Netherlands.

[5]Helmholtz Institute for Pharmaceutical Research (HIPS) -Helmholtz Centre for Infection Research (HZI), Campus Building E 8.1, D-66123, Saarbrücken, Saarland, Germany.

[6]Saarland University, PharmaScienceHub (PSH), Campus E2.1, 66123, Saarbrücken, Saarland,Germany.

[7]BASF-SE Carl-Bosch-Strasse 38, 67056 Ludwigshafen, Germany.

[8]Department of Chemical Biology, Helmholtz Centre for Infection Research (HZI), Inhoffenstraße 7, 38124 Braunschweig, Germany.

[9]German Center for Infection Research (DZIF), Partner Site Hannover-Braunschweig, Inhoffenstraße 7, 38124 Braunschweig, Germany.

*Corresponding author
Prof. Anna K. H. Hirsch
e-mail: anna.hirsch@helmholtz-hips.de

Table of Contents

1.0 General information ... 5
1.1 Chemicals, Materials and Methods ... 5
1.2 Abbreviations ... 6
1.3 Synthetic Schemes S1–S4 ... 6
2.0 Synthesis and characterization of compounds 25-70. ... 7
2.1 General procedures ... 7
2.2 Table S1. Chemical structures and purity of salicylic acid derivatives provided by BASF (5–24) .. 25
3.0 Molecular docking ... 27
4.0 Biological results ... 28
4.1 ECF-T folate uptake assay ... 28
Tables S2–S4. Inhibitory potency and IC_{50} determination of ECF compounds in the *Lactobacillus casei* whole-cell assay. ... 28
4.2 Radiolabeled folate and pantothenate transport assay ... 30
4.3 Cytotoxicity assay ... 34
4.4 Evaluation of MIC activities ... 34
5.0 ECF conservedness strain list ... 35
5.1 MIC determination ... 35
5.2 Untreated culture of *S. pneumoniae* ... 36
6.0 *In vitro* ADME assays ... 37
7.0 Mass spectrometric conditions of the pharmacokinetic studies ... 40
8.0 Spectra of representative compounds ... 40
Compound 27 ... 40
Compound 28 ... 44
Compound 29 ... 47
Compound 31 ... 50
Compound 33 ... 54
Compound 34 ... 57
Compound 35 ... 62
Compound 36 ... 66
Compound 37 ... 69

Compound 38 72
Compound 41 75
Compound 42 78
Compound 46 81
Compound 49 84
Compound 52 87
Compound 63 90
Compound 64 93
Compound 65 96
Compound 66 99
Compound 67 103
Compound 68 106
Compound 70 109
References 112

1.0 General information

1.1 Chemicals, Materials and Methods

NMR experiments were run on a Bruker Avance Neo 500 MHz spectrometer. Spectra were acquired at 300 K, using deuterated solvents. Chemical shifts for ^{1}H and ^{13}C spectra were recorded in parts per million (ppm) using the residual non-deuterated solvent as the internal standard. Coupling constants (*J*) are given in Hertz (Hz). Data are reported as follows: chemical shift, multiplicity (s = singlet, d =doublet, t = triplet, m = multiplet, br = broad and combinations of these) coupling constants and integration. Flash chromatography was performed using the automated flash chromatography system CombiFlash Rf+ (Teledyne Isco, Lincoln, NE, USA) equipped with RediSepRf silica columns (Axel Semrau, Sprockhövel Germany). TLC was performed with aluminum-backed silica TLC plates (Macherey-Nagel MN ALUGRAM Sheets SIL G/UV 254 20 x 20cm 818133) with a suitable solvent system and was visualized using UV fluorescence (254 & 366 nm). All reactions were carried out in oven-dried glassware under an atmosphere of argon. Anhydrous DMF was purchased from Aldrich and used directly.

Liquid chromatography-mass spectrometry was performed on a LC-MS system, consisting of a Dionex UltiMate 3000 pump, autosampler, column compartment and MWD or DAD detector (Thermo Fisher Scientific, Dreieich, Germany) and ESI quadrupole MS (MSQ Plus or ISQ EC, Thermo Fisher Scientific, Dreieich, Germany). Columns used: 1) Hypersil Gold column, 100 x 2.1 mm, 3 µm. At a flow rate of 700 µL/min, the gradient of H_2O (0.1% FA) and ACN (0.1% FA) starting from 5% ACN and then increased to 100% over 7 min. 2) Hypersil Gold column, 100 x 1.9 mm, 2.1 µm. At a flow rate of 600 µL/min, the gradient of H_2O (0.1% FA) and ACN (0.1% FA) starting from 5% ACN and then increased to 100% over 5.5 min. The mass spectrum was measured in positive and negative mode in a range from 100–600 *m/z*. The UV spectrum was recorded at 254 nm. High-resolution mass spectra (HR-MS) were recorded with a ThermoScientific system where a Dionex Ultimate 3000 RSLC was coupled to a Q Exactive Focus mass spectrometer with an electrospray ion (ESI) source. An Acquity UPLC® BEH C8, 150 x 2.1 mm, 1.7 µm column equipped with a VanGuard Pre-Column BEH C8, 5 x 2.1 mm, 1.7 µm (Waters, Germany) was used for separation. At a flow rate of 250 µL/min, the gradient of (A) H_2O + 0.1% FA and (B) ACN + 0.1% FA was held at 10% B for 1 min and then increased to 95% B over 4 min. It was held there for 1.2 min before the gradient was decreased to 10% B over 0.3 min where it was held for 1 min. The mass spectrum was measured in positive mode in a range from 120–1000 m/z. UV spectrum was recorded at 254 nm.

Preparative RP-HPLC was performed using an UltiMate 3000 Semi-Preparative System (Thermo Fisher Scientific) with nucleodur® C18 Gravity (250 mm × 16 mm, 5 µm). Separation was done using gradient 5–100% CH_3CN + 0.05% HCOOH in water + 0.05% HCOOH in 53 min at a flow rate of 10 mL/min and end with a 5 min step at 100% CH_3CN. The sample was dissolved in DMSO and manually injected to the HPLC system.

1.2 Abbreviations

Ammonium hydroxide (NH_4OH), boron trifluoride diethyl etherate ($BF_3 \cdot Et_2O$), chloroform ($CHCl_3$), dichloromethane (DCM), dimethylformamide (DMF), dimethylsulfoxide (DMSO), ethyl acetate (EtOAc), formic acid (HCO_2H), hydrochloric acid (HCl), methyl iodide (CH_3I), methanol (CH_3OH), sodium borohydride ($NaBH_4$), potassium carbonate (K_2CO_3), sodium sulfate (Na_2SO_4), sodium hydride 60% dispersion in mineral oil (NaH 60%), sodium hydroxide (NaOH), tetrahydrofuran (THF). Other abbreviations used are: aqueous (aq.), hours (h), minutes (min), room temperature (rt), on (overnight), saturated (sat.).

1.3 Synthetic Schemes S1–S4

Scheme S1. Synthesis of intermediates 48a, 50a and 52a.

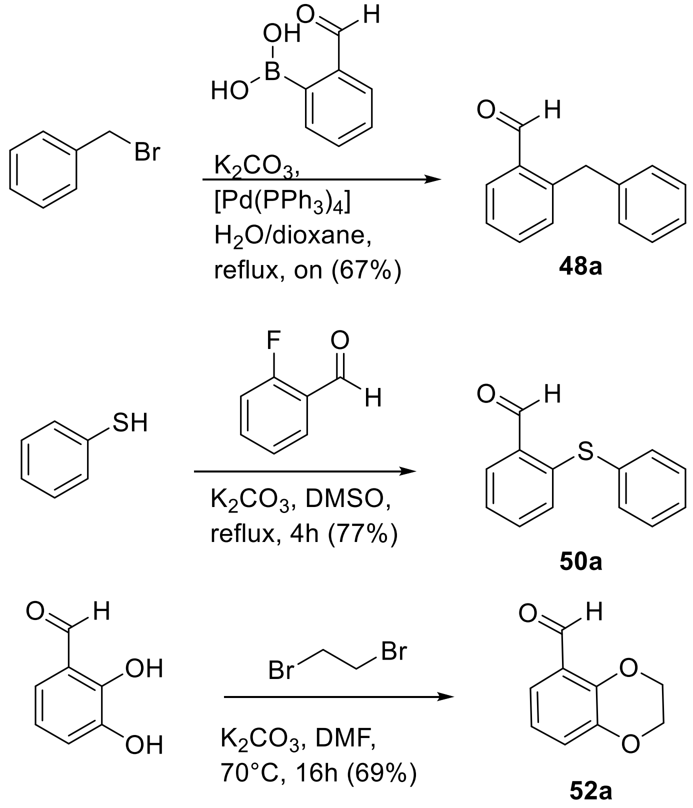

Scheme S2. Synthesis of 58

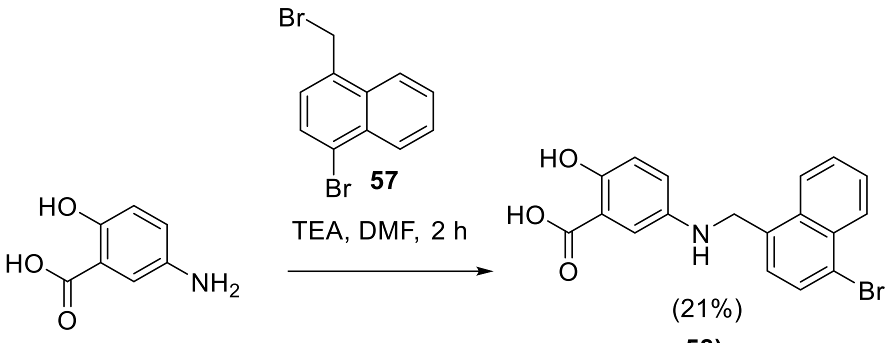

Scheme S3. Synthesis of 63-65[a]

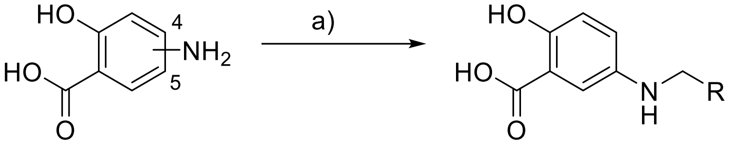

63) R= 5-triazol; R= 4-bromophenyl (38%)
64) R= 5-triazol; R= 3,5-dichlorophenyl (50%)
65) R= 4-triazol; R=3,5-dichlorophenyl (19%)

[a]Reagent and conditions: a) 5-amino-2-hydroxybenzoic acid or 4-amino-2-hydroxybenzoic acid, *t*-$BuNO_2$, Me_3SiN_3, MeCN, rt, 2 h, followed by 1-bromo-4-ethynylbenzene or 1,3-dichloro-5-ethynylbenzene, sodium ascorbate, $CuSO_4.5H_2O$, (H_2O:*t*-ButOH 1:1), rt, 24 h.

2.0 Synthesis and characterization of compounds 25-70.

2.1 General procedures

General procedure 1 (GP1): ether synthesis

To a stirred solution of 5-hydroxy-2-substituted benzoic acid (1.0 eq) in DMF (0.3 M), was added at 0 °C portionwise NaH (60%, 2.5 eq) previously washed with diethyl ether (3x5 mL). The reaction mixture was stirred at rt for 2 h, and then bromomethyl compounds (1.0 eq) were slowly added at 0 °C. The reaction mixture was stirred at rt on until TLC and LC-MS analysis showed the complete consumption of the starting materials. The resulting suspension was quenched at 0 °C with HCl (6 N) and extracted with EtOAc (3x10 mL). The combined organic layers were dried over Na_2SO_4, filtered, concentrated *in vacuo* and purified by flash chromatography.

General procedure 2 (GP2): reductive amination

An oven-dried round-bottomed flask was charged with 5-amino-2-hydroxybenzoic acid (1.0 eq.), aryl aldehyde (1.5 eq.) and $NaBH(OAc)_3$ (2.0 eq.) in dry DMF (0.3 M). The solution was stirred under N_2 on until the starting material was consumed as indicated by TLC and LC-MS analysis. The crude material was taken up with H_2O, and the aqueous phase was acidified with HCl (1 M) solution to a pH of 4–5 and extracted with EtOAc (3 x 20 mL). The combined organic layers were dried over Na_2SO_4, filtered, concentrated *in vacuo* and purified by flash chromatography.

General procedure 3 (GP3): coupling with chloride

Into an oven dried round-bottomed flask, aryl chloride (1.0 eq.) was added at 0 °C to a solution of 5-amino-2-hydroxybenzoic acid (1.0 eq.) in dry DMF (0.3 M). The solution is left to reach rt and stirred for 2 h. The reaction was poured into crushed ice and the aqueous phase was extracted with DCM (3 x 15 mL). The combined organic layers were dried over Na_2SO_4, filtered, concentrated *in vacuo* and purified by flash chromatography.

General procedure 4 (GP4): azide synthesis

To the amine derivative (1.0 eq.) in MeCN (0.2 M), was added dropwise *t*-$BuNO_2$ (4.0 eq.) and Me_3SiN_3 (3.0 eq.). The resulting solution was stirred at room temperature for 2 h. Water was added to the reaction mixture, and the mixture was extracted with CH_2Cl_2 (2 × 30 mL). The combined organic phases were washed with 20 mL of saturated aqueous NaCl solution. The resulting organic phase was dried over $MgSO_4$, and the filtrate was concentrated *in vacuo*. The product was used directly without further purification.

General procedure 4 (GP5): triazole synthesis

To the azide derivative (1.0 eq.) and alkyne derivative (1.0 eq.) was added 3 mL (H_2O:*t*-BuOH 1:1). Sodium ascorbate (0.1 eq.) of freshly prepared 1 M solution in water was added, followed by the addition of copper (II) sulfate pentahydrate (0.01 eq.). The resulting reaction was vigorously stirred at room temperature under Argon for 24 h. Excess solvent was evaporated under reduced pressure, and to the remaining residue, water (20 mL) was added, followed by extraction with ethyl acetate. The organic solvent was then dried over $MgSO_4$, filtered and evaporated under reduced pressure. Purification was done by semi-preparative HPLC.

2-Amino-5-(naphthalen-1-ylmethoxy)benzoic acid (25)

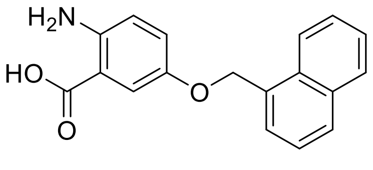

To a solution of **3**[1] (0.03 g, 0.07 mmol) in DCM (0.76 mL), HCl 4M in dioxane (48 eq.) was added, and the solution is stirred at rt for 2 h. The solution was concentrated *in vacuo* to give **25** as a yellowish powder (0.015 g, 70%). **^{1}H NMR** (500 MHz, DMSO-d_6) δ 8.37 (br, 2H), 8.09 (d, J = 8.1, 1H), 7.96 (dd, J = 8.9, 7.4, 1H), 7.92 (d, J = 8.2, 1H), 7.63 (d, J =

6.8, 1H), 7.60 – 7.53 (m, 1H), 7.52 – 7.46 (m, 1H), 7.38 (d, *J* = 2.9, 1H), 7.07 (dd, *J* = 8.9, 3.0, 1H), 6.73 (d, *J* = 8.9, 1H), 5.42 (s, 2H). **^{13}C NMR** (126 MHz DMSO-*d*$_6$) 169.2, 148.0, 146.5, 133.2, 132.9, 131.1, 128.5, 128.4, 126.6, 126.3, 125.9, 125.3, 123.9, 123.6, 117.8, 114.9, 109.4, 68.6. **HR-MS** (ESI) calcd for $C_{18}H_{16}NO_3$ [*M*+H]$^+$: 294.1125, found: 294.1119.

3- (Naphthalen-1-ylmethoxy)benzoic acid (26)

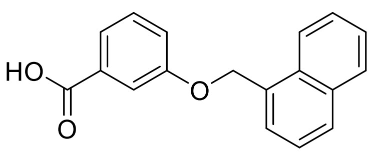

According to GP1, using 3-hydroxybenzoic acid (0.100 g, 0.724 mmol), NaH (0.072 g, 1.81 mmol) and naphthylbromide (0.160 g, 0.724 mmol) in DMF (2.4 mL) to give, after purification by column chromatography (hexane/EtOAc 3:7) **26** as white powder (0.025 g, 35%). **^{1}H NMR** (500 MHz, DMSO-*d*$_6$) *δ* 13.01 (s, 1H), 8.11 (d, *J* = 7.9, 1H), 7.98 (m, 1H), 7.94 (d, *J* = 8.2, 1H), 7.69 (d, *J* = 6.9, 1H), 7.61 (d, *J* = 1.7, 1H), 7.60 – 7.54 (m, 3H), 7.52 (m, 1H), 7.44 (t, *J* = 7.9, 1H), 7.35 (dd, *J* = 8.2, 2.0, 1H), 5.62 (s, 2H). **^{13}C NMR** (126 MHz DMSO-*d*$_6$) 167.3, 158.4, 133.3, 132.2, 132.2, 131.3, 129.8, 128.7, 128.5, 126.7, 126.5, 126.0, 125.4, 123.9, 121.9, 119.7, 115.0, 68.0. **HR-MS** (ESI) calcd for $C_{18}H_{13}O_3$ [*M*–H]$^-$: 277.0870, found: 277.0867.

2-Methyl-5-((naphthalen-1-yloxy)methyl)benzoic acid (27)

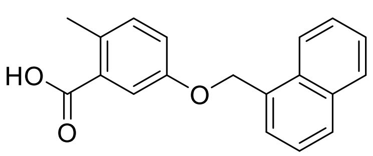

According to GP1, using 5-hydroxy-2-methylbenzoic acid (0.200 g, 1.31 mmol), NaH (0.13 g, 3.28 mmol) and naphthylbromide (0.29 g, 1.31 mmol) in DMF (4.38 mL) to give, after purification by column chromatography (hexane/EtOAC 3:7) **27** as a yellowish powder (0.16 g, 42%). **^{1}H NMR** (500 MHz, DMSO-*d*$_6$) *δ* 12.87 (s, 1H), 8.09 (d, *J* = 7.9, 1H), 7.99 – 7.96 (m, 1H), 7.93 (d, *J* = 8.2, 1H), 7.67 (d, *J* = 6.9, 1H), 7.61 – 7.54 (m, 2H), 7.53 – 7.50 (m, 1H, 7.49 (d, *J*=2.7, 1H), 7.23 (d, *J* = 8.4, 1H), 7.19 (dd, *J* = 8.4, 2.7, 1H), 5.56 (s, 2H), 2.44 (s, 3H). **^{13}C NMR** (126 MHz DMSO-*d*$_6$) 168.4, 156.2, 133.2, 132.6, 132.4, 131.3, 131.1, 131.0, 128.7, 128.4, 126.6, 126.4, 126.0, 125.3, 123.9, 118.5, 116.0, 68.0, 20.34. **HR-MS** (ESI) calcd for $C_{19}H_{15}O_3$ [*M*–H]$^-$: 291.1027, found: 291.1026.

2-(2-Hydroxy-5-(naphthalen-1-ylmethoxy)phenyl)acetic acid (28)

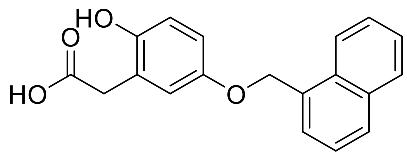

According to GP1, 2,5-dihydroxyphenylacetic acid (0.20 g, 1.19 mmol), NaH (0.117 g, 2.97 mmol) and naphthylbromide (0.263 g, 1.19 mmol) in DMF (3.95 mL) to give, after purification by column chromatography (hexane/EtOAc 1:9) **28** as yellow powder (0.16 g, 45%). **^{1}H NMR** (500 MHz, MeOD-*d*$_4$) *δ* 8.10 (d, *J* = 8.1, 1H), 7.88 (dd, *J* = 22.4, 8.1, 2H), 7.60 (d, *J*=6.9, 1H), 7.56 – 7.49 (m, 2H), 7.46 (t, *J*=7.6, 1H), 6.91 (d, *J*=2.4, 1H), 6.83 (dd, *J*=8.6, 2.5, 1H), 6.74 (d, *J*=8.7, 1H), 5.43 (s, 2H), 3.59 (s, 2H). **^{13}C NMR** (126 MHz, DMSO-*d*$_6$) *δ* = 172.6, 151.0, 149.6, 133.2, 132.9, 131.2, 128.5, 128.4, 126.5, 126.4, 125.9, 125.4, 123.9,

122.7, 117.8, 115.1, 113.7, 68.3, 35.5. **HR-MS** (ESI) calcd for $C_{19}H_{16}O_4$ [*M*-H]$^-$: 307.0976, found: 307.0970.

4-(2-Hydroxy-5-(naphthalen-1-ylmethoxy)phenyl)butanoic acid (29)

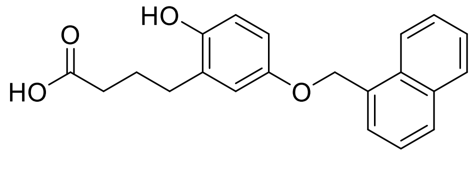

According to GP1, 4-(2,5-dihydroxyphenyl)butanoic acid (0.100 g, 0.51 mmol), NaH (0.83g, 1.25 mmol) and naphthylbromide (0.51 g, 1.3 mmol) in DMF (1.7 mL) were used to give, after purification by column chromatography (hexane/EtOAc 1:9) **29** as white powder (0.40 g, 40%). **^{1}H NMR** (500 MHz, DMSO-*d*$_6$) δ 8.51 (d, *J* = 12.3, 2H), 8.03 – 7.91 (m, 2H), 7.62 – 7.53 (m, 3H), 7.50 (t, *J*=7.6, 1H), 6.55 (d, *J* = 8.5, 1H), 6.43 (d, *J*=2.9, 1H), 6.39 (dd, *J*=8.4, 2.9, 1H), 5.54 (s, 2H), 2.44 (t, *J* = 7.5, 2H), 2.34 (t, *J* = 7.5, 2H), 1.82 – 1.71 (m, 2H). **^{13}C NMR** (126 MHz, DMSO-*d*$_6$) δ 172.8, 149.6, 147.5, 133.2, 131.7, 131.1, 128.9, 128.5, 128.1, 127.1, 126.6, 126.0, 125.4, 123.6, 116.4, 115.4, 113.1, 63.7, 33.2, 29.0, 24.7. **HR-MS** (ESI) calcd for $C_{21}H_{20}O_4$ [*M*-H]$^-$: 335.1288, found: 335.1282.

Methyl 2-hydroxy-5-((naphthalen-1-yloxy)methyl)benzoate (30)

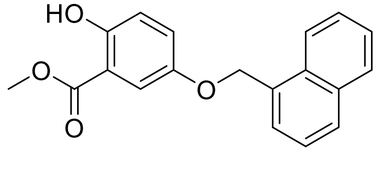

To a refluxed suspension of K_2CO_3 (3.29 g, 23.8 mmol) in MeOH (18.4 mL) and $CHCl_3$ (34 mL), was added a mixture of methyl 2,5-dihydroxybenzoate (1.0 g, 5.95 mmol) and naphthyl bromide (1.3 g, 5.95 mmol) in a mixture of MeOH/$CHCl_3$ (1:1, 1 M) dropwise over 30 min. The resulting mixture was stirred under reflux on. After cooling down and filtration, the suspension was quenched with H_2O and extracted with EtOAc (3x10 mL). The combined organic layers were dried over Na_2SO_4, filtered, concentrated *in vacuo* and purified by flash chromatography (hexane/EtOAc 9:1) to give **30** as white powder (1.2 g, 65%). **^{1}H NMR** (500 MHz, DMSO-*d*$_6$) δ 10.13 (s, 1H), 8.09 (d, *J* = 8.1, 1H), 7.97 (dd, *J* = 8.7, 7.1, 2H), 7.66 (d, *J* = 6.9, 1H), 7.61 – 7.54 (m, 2H), 7.54 – 7.48 (m, 1H), 7.45 (d, *J* = 3.2, 1H), 7.30 (dd, *J* = 9.0, 3.2, 1H), 6.96 (d, *J* = 9.0, 1H), 5.51 (s, 2H), 3.89 (s, 3H). **^{13}C NMR** (126 MHz DMSO-*d*$_6$) 168.9, 154.5, 150.8, 133.2, 132.4, 131.1, 128.7, 128.5, 126.7, 126.4, 125.9, 125.3, 124.2, 123.9, 118.5, 113.8, 112.8, 68.5, 52.53. **HR-MS** (ESI) calcd for $C_{19}H_{17}O_4$ [*M*+H]$^+$: 309.1121, found: 309.1118.

2-Hydroxy-5-(naphthalen-1-ylmethoxy)benzamide (31)

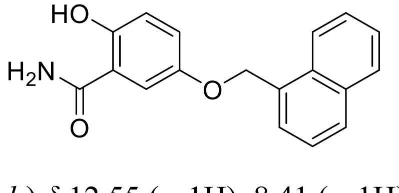

To a stirred solution of **30** (0.100 g, 0.324 mmol) in dioxane (3.2 mL, 0.1 M), NH_4OH (2 mL) was added, and the solution was stirred at rt for 2 d. The mixture was concentrated *in vacuo* to give **31** as a white powder (0.08 g, 88%). **^{1}H NMR** (500 MHz, DMSO-*d*$_6$) δ 12.55 (s, 1H), 8.41 (s, 1H), 8.09 (d, *J* = 8.2, 1H), 7.98 (d, *J* = 7.7, 1H), 7.94 (d, *J* = 8.2, 1H), 7.91 (s, 1H), 7.68 (d, *J* = 6.9, 1H), 7.65 (d, *J* = 3.0, 1H), 7.62 – 7.55 (m, 2H), 7.52 (dd, *J* = 15.2,

7.4, 1H), 7.18 (dd, J = 8.9, 2.9, 1H), 6.84 (d, J = 8.9, 1H), 5.49 (s, 2H). **^{13}C NMR** (126 MHz, DMSO-d_6) 171.8, 155.4, 150.4, 133.3, 132.6, 131.1, 128.7, 128.5, 126.6, 126.5, 126.0, 125.4, 123.9, 122.2, 118.2, 114.2, 112.8, 68.5. **HR-MS** (ESI) calcd for $C_{18}H_{16}NO_3$ [M+H]$^+$: 294.1125, found: 294.1119.

Methyl 2-methoxy-5-(naphthalen-1-ylmethoxy)benzoate (32a)

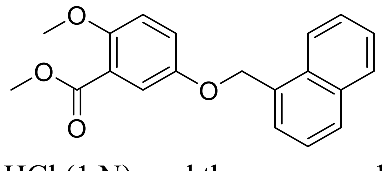

To a solution of **30** (0.150 g, 0.486 mmol) in DMF (2.43 mL, 0.2M), was slowly added at 0 °C NaH 60% (0.020 g, 0.486 mmol) followed by CH_3I (0.030 mL, 0.486 mmol), and the reaction mixture was stirred at rt for 5 h. The suspension was quenched with HCl (1 N), and the aqueous phase extracted with EtOAc (3x15 mL). The combined organic layers were dried over Na_2SO_4, filtered, concentrated *in vacuo* and purified by flash chromatography (hexane/EtOAc 95:5) to give **32a** as white powder (0.15 g, 98%). **^{1}H NMR** (500 MHz, DMSO-d_6) δ 8.10 (d, J = 8.0, 1H), 7.98 (d, J = 7.5, 1H), 7.93 (d, J = 8.2, 1H), 7.66 (d, J = 6.9, 1H), 7.61 – 7.54 (m, 2H), 7.51 (t, J = 7.6, 1H), 7.35 (d, J = 3.1, 1H), 7.28 (dd, J = 9.0, 3.1, 1H), 7.10 (d, J = 9.1, 1H), 5.53 (s, 2H), 3.78 (s, 3H), 3.77 (s, 3H). **^{13}C NMR** (126 MHz, DMSO-d_6) 166.0, 152.4, 151.6, 133.3, 132.4, 131.1, 128.7, 128.4, 126.6, 126.4, 125.9, 125.3, 123.9, 120.7, 119.9, 116.5, 114.1, 68.4, 56.3, 52.0. **HR-MS** (ESI) calcd for $C_{20}H_{19}O_4$ [M+H]$^+$: 323.1278, found: 323.1270.

2-Methoxy-5-(naphthalen-1-ylmethoxy)benzoic acid (32)

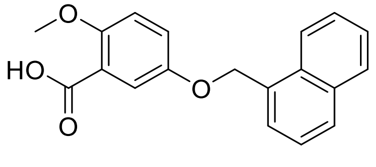

To a solution of **32a** (0.075 g, 0.03 mmol) in MeOH (1 mL, 0.2 M), NaOH 10% aq. (1 mL) was added, and the solution was stirred under reflux for 2 h. The solution was acidified with HCl (1N) and extracted with EtOAc (3x15 mL). The combined organic layers were dried over Na_2SO_4, filtered, concentrated *in vacuo* to give **32** as white powder (0.52 g, 74%). **^{1}H NMR** (500 MHz, DMSO-d_6) δ 12.66 (s, 1H), 8.14 – 8.07 (m, 1H), 8.00 – 7.96 (m, 1H), 7.93 (d, J = 8.2, 1H), 7.66 (d, J = 6.9, 1H), 7.61 – 7.54 (m, 2H), 7.53 – 7.48 (m, 1H), 7.33 (d, J = 3.2, 1H), 7.24 (dd, J = 9.0, 3.2, 1H), 7.07 (d, J = 9.1, 1H), 5.52 (s, 2H), 3.77 (s, 3H). **^{13}C NMR** (126 MHz, DMSO-d_6) 167.0, 152.4, 151.6, 133.2, 132.5, 131.1, 128.6, 128.4, 126.6, 126.4, 125.9, 125.38, 123.9, 122.0, 119.4, 116.5, 114.0, 68.4, 56.3. **HR-MS** (ESI) calcd for $C_{19}H_{17}O_4$ [M+H]$^+$: 309.1121, found: 309.1113.

2-(Hydroxymethyl)-4-(naphthalen-1-ylmethoxy)phenol (33)

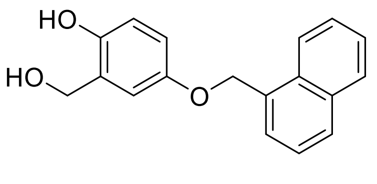

A solution of $BF_3{\cdot}Et_2O$ (0.04 mmol) in THF (1 mL) was added slowly to a solution of $NaBH_4$ (0.03 g, 0.76 mmol) and **1** (0.15 g, 0.51 mmol) in THF (5.1 mL). The mixture was heated to reflux on until TLC and LC-MS analysis showed complete consumption of

the substrate. The reaction mixture was cooled to 0 °C and quenched by addition of H_2O. After stirring for 30 min at 10 °C, the THF was removed *in vacuo*, DCM was added, and the stirring was continued for another 1 h. The organic layer was separated, washed with a saturated aqueous NaCl solution, dried over Na_2SO_4, filtered and concentrated *in vacuo* and purified by column chromatography (hexane/EtOAc 6:4) to afford **33** as a white powder (0.09 g, 65%). **^{1}H NMR** (500 MHz, DMSO-*d*$_6$) δ 8.90 (s, 1H), 8.08 (d, *J* = 7.8, 1H), 8.00 – 7.95 (m, 1H), 7.92 (d, *J* = 8.2, 1H), 7.64 (d, *J* = 6.8, 1H), 7.57 (m, 2H), 7.53 – 7.48 (m, 1H), 7.04 (d, *J* = 3.0, 1H), 6.79 (dd, *J* = 8.6, 3.1, 1H), 6.69 (d, *J* = 8.6, 1H), 5.43 (s, 2H), 4.98 (t, *J* = 5.6, 1H), 4.46 (d, *J* = 5.6, 2H). **^{13}C NMR** (126 MHz, DMSO-*d*$_6$) 151.3, 148.1, 133.2, 133.1, 131.2, 129.7, 128.5, 128.5, 126.5, 126.3, 125.9, 125.3, 123.9, 115.0, 114.0, 113.2, 68.3, 58.2. **HR-MS** (ESI) calcd for $C_{18}H_{15}O_2$ [*M*–OH-H]$^-$: 263.1077, found: 263.1060.

2-Hydroxy-5-((naphthalen-1-ylmethyl)amino)benzoic acid (34)

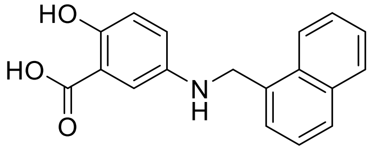

According to GP2, 5-amino-2-hydroxybenzoic acid (0.200 g, 1.3 mmol), naphthaldehyde (0.224 g, 1.44 mmol) and NaBH(OAc)$_3$ (0.36 g, 1.7 mmol) in DMF (4.3 mL) were used to give, after purification by column chromatography (hexane/EtOAc 3:7) **34** as a white powder (0.22 g, 59%). **^{1}H NMR** (500 MHz, DMSO-*d*$_6$) δ 8.15 (d, *J* = 8.2, 1H), 7.98 – 7.93 (m, 1H), 7.83 (d, *J* = 8.1, 1H), 7.59 – 7.53 (m, 2H), 7.52 (d, *J* = 6.9, 1H), 7.48 – 7.42 (m, 1H), 7.02 (d, *J* = 2.9, 1H), 6.94 (dd, *J* = 8.9, 3.0, 1H), 6.75 (d, *J* = 8.8, 1H), 4.65 (s, 2H). **^{13}C NMR** (126 MHz, DMSO-*d*$_6$) 172.1, 152.7, 141.6, 135.0, 133.4, 131.2, 128.5, 127.3, 126.1, 125.7, 125.5, 125.1, 123.7, 121.8, 117.5, 112.5, 111.0, 45.4. **HR-MS** (ESI) calcd for $C_{18}H_{14}NO_3$ [*M*-H]$^-$: 292.0979, found: 292.0977.

5-(1-Naphthamido)-2-hydroxybenzoic acid (35)

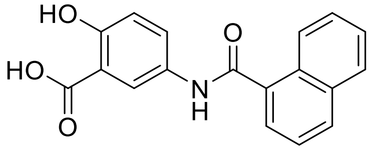

According to GP3, 1-naphthoyl chloride (0.22 mL, 1.43 mmol) and 5-amino-2-hydroxybenzoic acid (0.20 g, 1.30 mmol) in dry DMF (4.8 mL) were used to give, after prep. HPLC purification, **35** as white powder (0.15 g, 34%). **^{1}H NMR** (500 MHz, DMSO-*d*$_6$) δ 10.53 (s, 1H), 8.38 (d, *J* = 2.7, 1H), 8.23 – 8.17 (m, 1H), 8.08 (d, *J* = 8.3, 1H), 8.04 – 7.99 (m, 1H), 7.85 (dd, *J* = 8.9, 2.7, 1H), 7.76 (dd, *J* = 7.0, 0.9, 1H), 7.64 – 7.55 (m, 3H), 6.99 (d, *J* = 8.9, 1H). **^{13}C NMR** (126 MHz, DMSO-*d*$_6$) 171.8, 167.0, 157.4, 134.6, 133.2, 131.0, 130.1, 129.7, 128.3, 128.1, 127.0, 126.4, 125.5, 125.2, 125.0, 121.2, 117.2, 112.5. **HR-MS** (ESI) calcd for $C_{18}H_{14}NO_4$ [*M*+H]$^+$: 308.0917, found: 308.0903.

2-Hydroxy-5-(naphthalene-1-sulfonamido)benzoic acid (36)

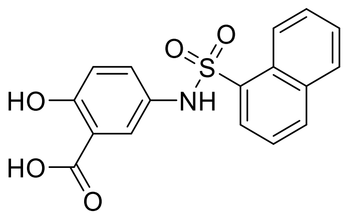

According to GP3, naphthalene-1-sulfonyl chloride (0.30 g, 1.32 mmol) and 5-amino-2-hydroxybenzoic acid (0.20 g, 1.32 mmol) in dry DMF (4.3 mL) were used to give, after flash chromatography (DCM/MeOH 9:1), **36** as brownish powder (0.21 g, 46%). **^{1}H NMR** (500 MHz, DMSO-*d*$_6$) δ 10.38 (s, 1H), 8.69 (d, *J* = 8.7, 1H), 8.20 (d, *J* = 8.3, 1H), 8.07 (dd, *J* = 7.4, 1.1, 2H), 7.73 (ddt, *J* = 16.8, 11.3, 5.6, 1H), 7.67 (ddd, *J* = 8.0, 6.9, 1.1, 1H), 7.58 (dd, *J* = 8.1, 7.5, 1H), 7.37 (d, *J* = 2.8, 1H), 7.09 (dd, *J* = 8.9, 2.8, 1H), 6.75 (d, *J* = 8.9, 1H). **^{13}C NMR** (126 MHz, DMSO-*d*$_6$) 171.1, 158.1, 134.4, 134.2, 133.7, 129.8, 129.2, 129.1, 128.5, 128.1, 127.5, 127.0, 124.4, 124.3, 122.6, 117.8, 113.0. **HR-MS** (ESI) calcd for $C_{17}H_{12}NO_5S$ [*M*-H]$^-$: 342.0442, found: 342.0439.

5-Azido-2-hydroxybenzoic acid (37a)

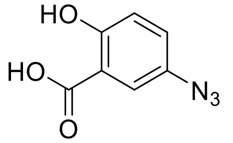

According to GP4, to a stirring solution of 5-amino-2-hydroxybenzoic acid (500 mg, 3.26 mmol) in MeCN (16.3 mL, 0.2 M) was added dropwise *t*-BuNO$_2$ (1.35 g, 13.05 mmol) and Me_3SiN_3 (1.128 g, 9.79 mmol) to afford **37a**, which was used without further purification (crude yield 85%).

2-Hydroxy-5-(4-(naphthalen-1-yl)-1*H*-1,2,3-triazol-1-yl)benzoic acid (37)

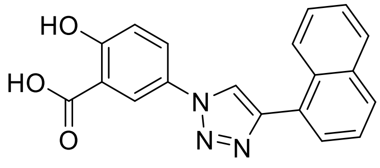

According to GP5, **37a** (0.05 g, 0.28 mmol) and 1-ethynylnaphthalene (0.042 g, 0.28 mmol) in (H_2O:*t*-ButOH 1:1, 3 mL), sodium ascorbate (5.52 mg, 0.028 mmol) and copper (II) sulfate pentahydrate (0.7 mg, 0.0028 mmol) were used to give, after preparative HPLC purification, compound **38** as white powder (0.044 g, 48%). **^{1}H NMR** (500 MHz, DMSO-*d*$_6$) δ 9.27 (s, 1H), 8.60 (dd, *J* = 6.7, 2.8, 1H), 8.39 (d, *J* = 2.8, 1H), 8.17 (dd, *J* = 8.9, 2.8, 1H), 8.02 (dd, *J* = 8.7, 5.8, 2H), 7.88 (dd, *J* = 7.1, 1.0, 1H), 7.66 – 7.58 (m, 3H), 7.23 (d, *J* = 8.9, 1H). **^{13}C NMR** (126 MHz, DMSO-*d*$_6$) δ 171.0, 160.9, 146.6, 133.6, 130.3, 128.8, 128.6, 128.5, 127.7, 127.5, 127.1, 126.8, 126.2, 125.6, 125.5, 122.4, 121.9, 118.6, 114.0. **HR-MS** (ESI) calcd for $C_{19}H_{14}N_3O_3$ [*M*+H]$^+$: 332.1029, found: 332.1016.

4-Azido-2-hydroxybenzoic acid (38a)

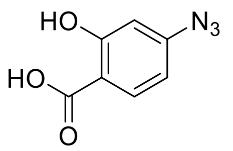

According to GP4, 4-amino-2-hydroxybenzoic acid (500 mg, 3.26 mmol) in MeCN (16.3 mL, 0.2 M) was added dropwise *t*-BuNO$_2$ (1.346 g, 13.05 mmol) and Me_3SiN_3 (1.128 g, 9.79 mmol) was used to give **38a**, which was used without further purification (crude yield 85%).

2-Hydroxy-4-(4-(naphthalen-1-yl)-1*H*-1,2,3-triazol-1-yl)benzoic acid (38)

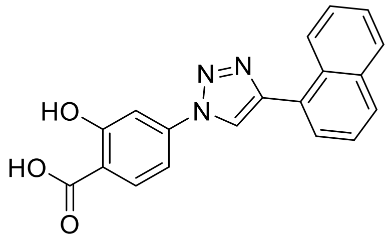

According to GP5, **38a** (0.05 g, 0.28 mmol) and 1-ethynylnaphthalene (0.042 g, 0.28 mmol) in (H_2O:*t*-ButOH 1:1, 3 mL), sodium ascorbate (5.52 mg, 0.028 mmol) and copper (II) sulfate pentahydrate (0.7 mg, 0.0028 mmol) were used to give, after preparative HPLC purification, compound **38** as beige powder (0.019 g, 20%). **^{1}H NMR** (500 MHz, Acetone) δ 9.12 (s, 1H), 8.68 – 8.61 (m, 1H), 8.16 (d, J = 8.6, 1H), 8.01 (d, J = 8.8, 2H), 7.92 (d, J = 7.0, 1H), 7.73 (dd, J = 8.6, 2.0, 1H), 7.69 (d, J = 1.9, 1H), 7.65 – 7.56 (m, 3H). **^{13}C NMR** (126 MHz, Acetone) δ 171.9, 163.9, 148.5, 143.3, 135.0, 133.3, 131.9, 130.0, 129.4, 128.7, 128.2, 127.5, 127.0, 126.6, 126.3, 122.5, 113.1, 111.5, 108.8. **HR-MS** (ESI) calcd for $C_{19}H_{12}N_3O_3$ [*M*-H]-: 330.0884, found: 330.0871.

2-Hydroxy-5-(2-(naphthalen-1-yloxy)ethoxy)benzoic acid (39)

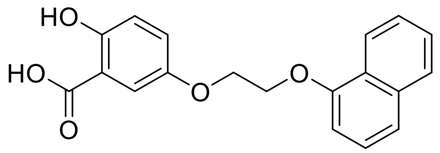

According to GP1, 2,5-dihydroxybenzoic acid (0.12 g, 0.8 mmol, 1 eq), 1-(2-bromoethoxy)naphthalene (0.20 g, 0.8 mmol, 1 eq) and NaH (0.08 g, 2 mmol), in DMF (2.6 mL) were used to give after flash chromatography (hexane/EtOAc 1:9) **39** as white powder (0.08 g, 31%). **^{1}H NMR** (500 MHz, DMSO-d_6) 8.12 (d, J = 8.3, 1H), 7.86 (d, J = 8.1, 1H), 7.56 – 7.37 (m, 5H), 7.25 (d, J = 9.0, 1H), 7.03 (d, J = 7.5, 1H), 6.92 (d, J = 9.0, 1H), 4.46 (d, J = 6.9, 4H). **^{13}C NMR** (126 MHz DMSO-d_6) 171.6, 156.6, 153.8, 150.8, 134.0, 127.4, 126.5, 126.2, 125.3, 124.8, 124.2, 121.6, 120.2, 118.2, 113.8, 112.8, 105.4, 67.2, 67.0. **HR-MS** (ESI) calcd for $C_{19}H_{17}O_5$ [M+H]$^+$: 325.1070, found: 325.1060.

Methyl 2-hydroxy-5-(naphthalen-1-ylethynyl)benzoate (40)

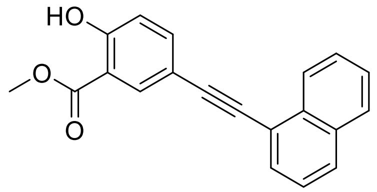

To an oven-dried Schlenk flask, 1-ethynylnaphthalene (0.50 g, 3.28 mmol, 1.0 eq.) and dry TEA (0.45 mL, 3.28 mmol, 1.0 eq) were added to a solution of methyl 2-hydroxy-5-iodobenzoate (0.91g, 3.28 mol, 1.0 eq.), CuI (4 mg, 0.6%), [$PdCl_2(PPh_3)_2$] (4.6 mg, 0.2%) and PPh_3 (7 mg, 0.9%) in MeCN (0.2 M). The mixture was refluxed for 2 h and a white precipitate formed (**40**, 0.58 g, 59%). **^{1}H NMR** (500 MHz, DMSO-d_6) δ 10.77 (s, 1H), 8.36 (d, J = 8.4, 1H), 8.04 (d, J = 2.2, 1H), 8.00 (t, J = 7.8, 2H), 7.84 – 7.78 (m, 2H), 7.68 (ddd, J = 8.2, 6.9, 1.2, 1H), 7.62 (ddd, J = 8.0, 6.9, 1.1, 1H), 7.55 (dd, J = 8.2, 7.2, 1H), 7.09 (d, J = 8.6, 1H), 3.92 (s, 3H). **^{13}C NMR** (126 MHz, DMSO-d_6) 168.0, 159.8, 138.0, 133.4, 132.8, 132.4, 130.3, 129.0, 128.5, 127.3, 126.8, 125.6, 125.5, 119.8, 118.3, 114.4, 113.3, 93.3, 86.1, 52.6.

2-Hydroxy-5-(naphthalen-1-ylethynyl)benzoic acid (41)

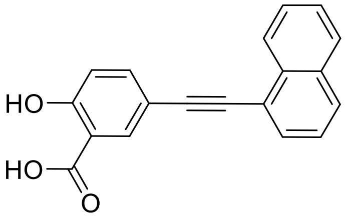

To a solution of **40** (0.05 g, 0.16 mmol) in MeOH (8 mL, 0.02M) was added NaOH 10 % (6 mL). The reaction mixture was stirred at 65 °C for 5 h. Upon completion, the solvent was evaporated, and the aqueous solution was acidified and diluted with EtOAc (3 x 15 mL). The crude product was purified by flash chromatography (hexane/EtOAc 4:6) to give **41** (0.032 g, 69%) as white powder. **^{1}H NMR** (500 MHz, DMSO-*d$_6$*) *δ* 8.37 (d, *J* = 8.3, 1H), 8.07 (d, *J* = 2.2, 1H), 8.00 (t, *J* = 8.0, 2H), 7.85 – 7.79 (m, 2H), 7.72 – 7.65 (m, 1H), 7.64 – 7.60 (m, 1H), 7.55 (dd, *J* = 8.2, 7.2, 1H), 7.07 (d, *J* = 8.6, 1H). **^{13}C NMR** (126 MHz, DMSO-*d$_6$*) 171.1, 161.1, 138.3, 133.5, 132.9, 132.4, 130.3, 128.9, 128.5, 127.4, 126.8, 125.7, 125.6, 119.9, 118.1, 113.9, 113.1, 93.5, 86.0. **HR-MS** (ESI) calcd for $C_{19}H_{11}O_3$ [*M*-H]$^-$: 287.0714, found: 287.0702.

2-Hydroxy-5-(2-(naphthalen-1-yl)ethyl)benzoic acid (42)

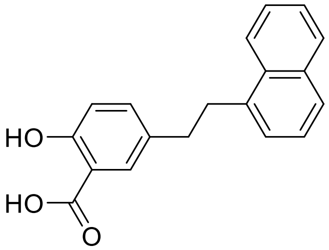

To a stirred solution of **41** (0.10 g, 0.35 mmol, 1.0 eq) in EtOH (3.5 mL), 10% Pd/C (0.04 g, 0.035 mmol, 0.1 eq) was added. The reaction mixture was stirred at rt on until the starting material was consumed. The resulting suspension was passed through a bed of Celite and washed with copious amounts of EtOH. The solvent was then removed under reduced pressure to give the product **42** as white powder (0.04 g, 44%). **^{1}H NMR** (500 MHz, DMSO-*d$_6$*) *δ* 8.16 (d, *J* = 8.5, 1H), 7.95 – 7.91 (m, 1H), 7.78 (d, *J* = 8.1, 1H), 7.66 (d, *J* = 2.3, 1H), 7.61 – 7.55 (m, 1H), 7.53 (ddd, *J* = 7.9, 6.8, 1.2, 1H), 7.47 – 7.42 (m, 1H), 7.42 – 7.39 (m, 1H), 7.37 – 7.33 (m, 1H), 3.31 (dd, *J* = 9.5, 7.1, 8H), 2.93 (dd, *J* = 9.3, 6.9, 2H). **^{13}C NMR** (126 MHz, DMSO-*d$_6$*) 171.9, 159.5, 137.4, 135.9, 133.4, 132.2, 131.3, 129.5, 128.6, 126.5, 126.2, 126.0, 125.5, 123.7, 117.0, 112.5, 35.4, 34.3. **HR-MS** (ESI) calcd for $C_{19}H_{15}O_3$ [*M*-H]$^-$: 291.1026, found: 291.1021.

(*E*)-2-Hydroxy-5-(2-(naphthalen-1-yl)vinyl)benzoic acid (43)[4]

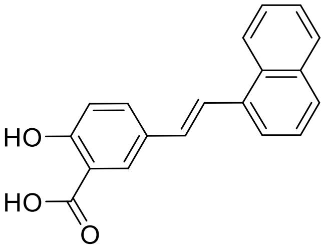

To an oven dried 10 mL screw-top vial equipped with a magnetic stirrer bar were added **40** (0.31 g, 1.01 mmol), zinc (0.53 g, 8.12 mmol, 8.0 eq), $NiCl_2$·dme (0.044 g, 0.20 mmol, 0.2 eq.), triphos (0.124 g, 0.2 mmol, 0.2 eq.) and HCO_2H (0.42 mL, 8.12 mmol, 8.0 eq.) in 1,4-dioxane (0.2 M, 2 mL). The vial was then sealed and transferred to a heating block at 120 °C for 48 h. After being allowed to cool, the crude reaction mixture was filtered through a celite plug; the filter cake was then washed with CH_2Cl_2, and the reaction mixture concentrated *in vacuo* to give after purification (cyclohexane/EtOAc 9:1) the desired (*E*)-alkene **43** as yellowish solid (0.18 g, 61%). **^{1}H NMR** (500 MHz, DMSO-*d$_6$*) *δ* 8.42 (d, *J* = 8.1, 1H), 8.06 (d, *J* = 9.1, 2H), 7.96 (d, *J* = 5.5, 1H), 7.94 (s, 1H), 7.87 (d, *J* = 7.8, 2H), 7.56 (td, *J* = 15.2, 6.6 Hz, 3H), 7.29 (d, *J* = 16.1, 1H), 7.03 (d, *J* = 8.3, 1H). **^{13}C NMR** (126 MHz, DMSO-*d$_6$*) *δ* 171.8, 160.8, 137.3, 134.4, 133.4, 133.3, 130.7, 130.3,

129.1, 128.7, 128.4, 127.6, 126.1, 125.9, 125.8, 123.9, 123.3, 122.9, 117.6. **HR-MS** (ESI) calcd for $C_{19}H_{13}O_3^-$ [*M*–H]$^-$: 289.0870, found: 289.0860.

5-(Benzylamino)-2-hydroxybenzoic acid (44)

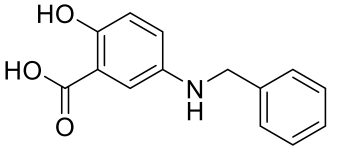

According to GP2, 5-amino-2-hydroxybenzoic acid (0.250 g, 1.63 mmol), benzaldehyde (0.191 g, 1.8 mmol) and $NaBH(OAc)_3$ (0.45 g, 2.12 mmol) in DMF (5.4 mL) were used to give, after purification by column chromatography (hexane/EtOAc 6:4), **44** as a white powder (0.22 g, 55%). **^{1}H NMR** (500 MHz, DMSO-d_6) δ 7.35 (d, *J* = 7.1, 2H), 7.31 (dd, *J* = 10.3, 4.9, 2H), 7.22 (t, *J* = 7.2, 1H), 6.95 (d, *J* = 2.9, 1H), 6.87 (dd, *J* = 8.8, 3.0, 1H), 6.71 (d, *J* = 8.8, 1H), 4.21 (s, 2H).**^{13}C NMR** (126 MHz DMSO-d_6) 172.0, 152.7, 141.2, 140.1, 128.2, 127.2, 126.6, 121.8, 117.3, 112.59, 11.3, 47.2. **HR-MS** (ESI) calcd for $C_{14}H_{14}NO_3$ [*M*+H]$^+$: 244.0968, found: 244.0963.

5-(((2,3-Dimethylphenyl)amino)methyl)-2-hydroxybenzoic acid (45)

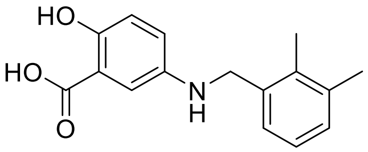

According to GP2, 5-amino-2-hydroxybenzoic acid (0.25 g, 1.63 mmol), 2,3-dimethyl-benzaldehyde (0.24 g, 1.8 mmol) and $NaBH(OAc)_3$ (0.45 g, 2.12 mmol) in DMF (5.4 mL) were used to give, after purification by column chromatography (hexane/EtOAc 6:4), **45** as a light brown powder (0.22 g, 17%). **^{1}H NMR** (500 MHz, DMSO-d_6) δ 7.12 (d, *J* = 7.3, 1H), 7.06 (d, *J* = 7.0, 1H), 7.01 (t, *J* = 7.4, 1H), 6.96 (d, *J* = 2.8, 1H), 6.88 (dd, *J* = 8.8, 2.9, 1H), 6.73 (d, *J* = 8.8, 1H), 4.14 (s, 2H), 2.25 (s, 3H), 2.20 (s, 3 H). **^{13}C NMR** (126 MHz, DMSO-d_6) 172.1, 152.7, 141.6, 137.2, 136.2, 134.5, 128.4, 125.7, 125.0, 121.7, 117.3, 112.5, 110.8, 46.2, 20.0, 14.3. **HR-MS** (ESI) calcd for $C_{16}H_{18}NO_3$ [*M*+H]$^+$: 272.1281, found: 272.1275.

2-Hydroxy-5-((2-(methylsulfonyl)benzyl)amino)benzoic acid (46)

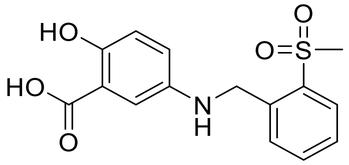

According to GP2, 5-amino-2-hydroxybenzoic acid (0.30 g, 1.96 mmol), 2-(methylsulfonyl)benzaldehyde (0.36 g, 2.94 mmol) and $NaBH(OAc)_3$ (0.83 g, 3.92 mmol) in DMF (6.5 mL) were used to give, after purification by column chromatography (hexane/EtOAc 6:4), **46** as a light-yellow powder (0.41 g, 65%). **^{1}H NMR** (500 MHz, DMSO-d_6) δ 7.96 (dd, *J* = 15.8, 8.0, 1H), 7.67 (ddd, *J* = 13.8, 10.3, 3.8, 2H), 7.58 – 7.48 (m, 1H), 6.98 (d, *J* = 2.9, 1H), 6.88 (dd, *J* = 8.9, 3.0, 1H), 6.74 (d, *J* = 8.8, 1H), 4.67 (s, 2H), 3.30 (s, 3H). **^{13}C NMR** (126 MHz, DMSO-d_6) 172.0, 153.0, 140.9, 139.5, 138.6, 133.8, 129.4, 128.9, 127.7, 121.7, 117.6, 112.7, 111.6, 44.1, 44.0. **HR-MS** (ESI) calcd for $C_{15}H_{16}NO_5S$ [*M*+H]$^+$: 322.0744, found: 322.0731.

5-(([1,1'-biPhenyl]-2-ylmethyl)amino)-2-hydroxybenzoic acid (47)

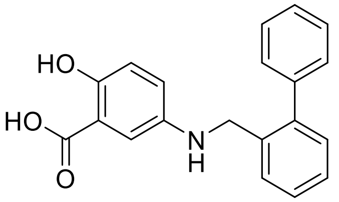

According to GP2, 5-amino-2-hydroxybenzoic acid (0.30 g, 1.96 mmol), [1,1'-biphenyl]-2-carbaldehyde (0.53 g, 2.94 mmol) and $NaBH(OAc)_3$ (0.83 g, 3.92 mmol) in DMF (6.5 mL) were used to give, after purification by column chromatography (hexane/EtOAc 6:4), **47** as light-brown powder (0.35 g, 56%). **^{1}H NMR** (500 MHz, DMSO-d_6) δ 7.50 (dd, J = 7.3, 1.5, 1H), 7.46 – 7.37 (m, 5H), 7.32 (ddd, J = 14.0, 7.1, 1.7, 2H), 7.26 – 7.20 (m, 1H), 6.79 – 6.73 (m, 2H), 6.70 – 6.65 (m, 1H), 4.05 (s, 2H). **^{13}C NMR** (126 MHz, DMSO-d_6) 172.0, 152.7, 141.20, 141.18, 140.4, 136.7, 129.6, 128.9, 128.2, 128.1, 127.4, 127.19, 126.8, 121.7, 117.3, 112.4, 110.8, 45.2. **HR-MS** (ESI) calcd for $C_{20}H_{18}NO_3$ [M+H]$^+$: 320.1281, found: 320.1275.

2-Benzylbenzaldehyde (48a)

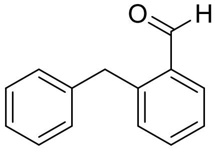

In a two-necked flask, bromomethyl benzene (0.25 g, 1.46 mmol, 1.0 eq.), K_2CO_3 (2.5 eq.) and (2-formylphenyl)boronic acid (0.24 g, 1.61 mmol, 1.1 eq.) were dissolved in a dioxane:H_2O mixture (5:1), and the mixture was degassed. Afterwards, [Pd(PPh_3)$_4$] (0.04 g, 0.036 mmol, 0.025 eq.) was added and the mixture was refluxed overnight. After cooling, the reaction was quenched with HCl 1M and washed with EtOAc (x 3). The crude material was purified by column chromatography (EtOAc/n-Hexane = 5:95) to give **48a** (0.18 mg, 67%) as a pale oil. **^{1}H NMR** (300 MHz, Acetone) δ 10.32 (s, 1H), 7.89 (dd, J = 7.6, 1.5, 1H), 7.59 (td, J = 7.5, 1.5, 1H), 7.46 (td, J = 7.6, 1.2, 1H), 7.31 – 7.23 (m, 2H), 7.22 – 7.09 (m, 3H), 4.50 (s, 2H). **^{13}C NMR** (75 MHz, Acetone) δ 206.2, 192.9, 143.9, 141.8, 135.0, 134.6, 132.4, 129.5, 129.3, 127.8, 126.9, 38.1. **LC-MS**, rt = 8.70 min.

5-(((2-Benzylphenyl)amino)methyl)-2-hydroxybenzoic acid (48)

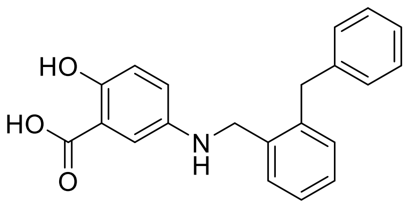

According to GP2, 5-amino-2-hydroxybenzoic acid (0.25 g, 1.63 mmol), **48a** (0.24 g, 1.8 mmol) and $NaBH(OAc)_3$ (0.45 g, 2.12 mmol) in DMF (5.4 mL) were used to give, after purification by column chromatography (hexane/EtOAc 6:4), **48** as a light-brown powder (0.22 g, 17%). **^{1}H NMR** (500 MHz, DMSO-d_6) δ 7.36 – 7.24 (m, J = 7.6, 3H), 7.22 – 7.13 (m, 6H), 6.87 (d, J = 2.8, 1H), 6.74 (dd, J = 8.8, 2.8, 1H), 6.68 (d, J = 8.8, 1H), 4.12 (s, 2H), 4.07 (s, 2H). **^{13}C NMR** (126 MHz, DMSO-d_6) 172.1, 152.7, 141.4, 140.3, 138.6, 137.5, 130.2, 128.6, 128.4, 127.5, 126.8, 126.3, 126.0, 121.5, 117.3, 111.1, 45.0, 37.7. **HR-MS** (ESI) calcd for $C_{21}H_{20}NO_3$ [M+H]$^+$: 334.1438, found: 334.1431.

2-Hydroxy-5-((2-phenoxybenzyl)amino)benzoic acid (49)

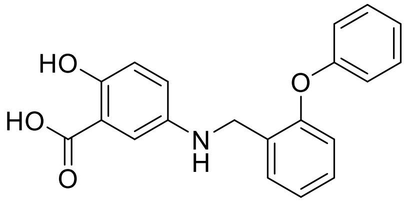

According to GP2, 5-amino-2-hydroxybenzoic acid (0.25 g, 1.63 mmol), 2-phenoxybenzaldehyde (0.48 g, 2.4 mmol) and $NaBH(OAc)_3$ (0.69 g, 3.26 mmol) in DMF (5.4 mL) were used to give, after purification by column chromatography (hexane/EtOAc 6:4), **49** as a light yellow powder (0.22 g, 40%). **^{1}H NMR** (500 MHz, DMSO-d_6) δ 7.44 – 7.35 (m, 3H), 7.25 (td, J=8.0, 1.4, 1H), 7.12 (q, J = 7.2, 2H), 6.98 (d, J = 7.9, 2H), 6.92 – 6.83 (m, 3H), 6.72 (d, J = 8.7, 1H), 4.20 (s, 2H). **^{13}C NMR** (126 MHz, DMSO-d_6) 172.0, 157.2, 153.8, 152.8, 141.1, 130.9, 130.1, 128.8, 128.3, 124.0, 123.0, 121.9, 119.2, 117.6, 117.5, 112.5, 111.0, 41.7. **HR-MS** (ESI) calcd for $C_{20}H_{18}NO_4$ $[M{+}H]^+$: 336.1230, found: 336.1216.

2-(Phenylthio)benzaldehyde (50a)

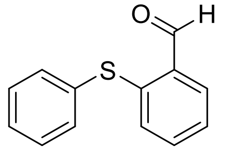

2-Fluoro benzaldehyde (0.6 g, 4.75 mmol, 1.0 eq), benzothiol (0.5 mL, 4.75 mmol, 1.0 eq) and K_2CO_3 (1.4 g, 9.96 mmol, 2.1 eq) were dissolved in DMSO (5 mL), and the mixture was heated to 100 °C for 4 h. After completion, the mixture was cooled and poured into H_2O. The aqueous layer was extracted with EtOAc (x 3) to afford **50a** as yellowish solid (0.78 g, 77%). **^{1}H NMR** (500 MHz, DMSO-d_6) δ 7.96 (dd, J = 7.6, 1.5, 1H), 7.53 (td, J = 7.7, 1.6, 1H), 7.50 – 7.45 (m, 5H), 7.42 (t, J = 7.4, 1H), 6.96 (d, J = 7.9, 1H). **^{13}C NMR** (126 MHz, DMSO-d_6) δ 192.1, 140.6, 134.4, 133.8, 133.5, 132.8, 131.9, 130.1, 129.1, 128.6, 126.1. **LC-MS**, rt = 4.36 min.

2-Hydroxy-5-((2-(phenylthio)benzyl)amino)benzoic acid (50)

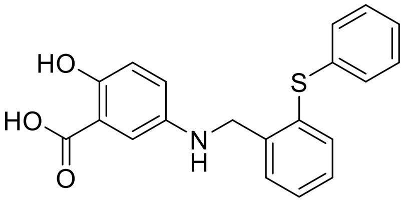

According to GP2, 5-amino-2-hydroxybenzoic acid (0.25 g, 1.63 mmol), **50a** (0.52 g, 2.4 mmol) and $NaBH(OAc)_3$ (0.69 g, 3.26 mmol) in DMF (5.4 mL) were used to give, after purification by column chromatography (hexane/EtOAc 6:4) **50** as a white powder (0.26 g, 46%). **^{1}H NMR** (500 MHz, DMSO-d_6) δ 7.44 (d, J = 7.4, 1H), 7.39 – 7.30 (m, 4H), 7.30 – 7.21 (m, 4H), 6.83 (d, J = 2.9, 1H), 6.78 (dd, J = 8.8, 2.9, 1H), 6.70 (d, J = 8.8, 1H), 4.26 (s, 2H). **^{13}C NMR** (126 MHz, DMSO-d_6) 172.0, 152.8, 141.0, 140.9, 135.4, 133.3, 132.1, 129.5, 129.3, 128.3, 128.0, 127.8, 126.8, 121.7, 117.4, 112.5, 110.9, 45.4. **HR-MS** (ESI) calcd for $C_{20}H_{18}NO_3S$ $[M{+}H]^+$: 352,1002, found: 352.0986.

2-Hydroxy-5-((2-(phenylsulfonyl)benzyl)amino)benzoic acid (51)

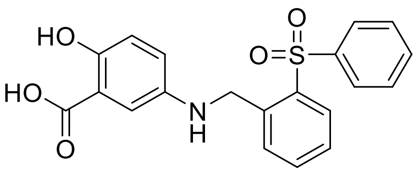

According to GP2, using 5-amino-2-hydroxybenzoic acid (0.10 g, 0.65 mmol), 2-(phenylsulfonyl)benzaldehyde (0.17 g, 0.98 mmol) and $NaBH(Ac)_3$ (0.27 g, 1.3 mmol) in DMF (2 mL) were used to give, after purification by column chromatography (DCM/MeOH 9:1), **51** as light brown solid (0.025 g, 10%). **^{1}H NMR** (500 MHz, DMSO-d_6) δ 8.18 (dd, J = 7.8, 0.9, 1H), 7.90 (d, J = 7.4,

2H), 7.74 (t, J = 7.4, 1H), 7.70 – 7.63 (m, 3H), 7.60 – 7.52 (m, 2H), 6.64 (d, J = 2.9, 1H), 6.58 (d, J = 8.8, 1H), 6.37 (dd, J = 8.8, 3.0, 1H), 4.36 (s, 2H). **^{13}C NMR** (126 MHz, DMSO-d_6) 171.86, 152.79, 140.55, 140.35, 139.37, 137.62, 134.27, 133.83, 129.75, 129.32, 128.31, 127.70, 127.22, 120.88, 117.45, 112.52, 111.22, 43.77. **HR-MS** (ESI) calcd for $C_{20}H_{16}NO_5S$ [M-H]$^-$: 382.0755, found: 382.0738.

2,3-*di*Hydrobenzo[*b*][1,4]dioxine-5-carbaldehyde (52a)

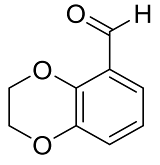

To a solution of 2,3-dihydroxybenzaldehyde (0.40 g, 2.89 mmol) and K_2CO_3 (1.60 g, 11.6 mmol) in DMF (16 mL), was added 1,2-dibromoethane (1 mL, 11.6 mmol). The resulting mixture was stirred at 70 °C for 16 h. Diluted HCl was added, and the mixture was extracted with EtOAc (3 x 20 mL). The combined organic layers were dried over Na_2SO_4, filtered, concentrated *in vacuo* and purified by flash chromatography (hexane/EtOAc 1:1) yielding **52a** as brown solid (0.33 g, 69%). **^{1}H NMR** (300 MHz, DMSO-d_6) δ 10.29 (d, J = 0.5, 1H), 7.26 (dt, J = 6.0, 3.0, 1H), 7.17 (dd, J = 8.0, 1.6, 1H), 7.00 – 6.90 (m, 1H), 4.45 – 4.26 (m, 4H). **^{13}C NMR** (126 MHz, DMSO-d_6) 188.5, 146.5, 144.1, 124.6, 123.0, 120.8, 119.8, 64.6, 63.7. **LC-MS**: 165 (M+H), rt = 2.66 min.

5-(((2,3-*di*Hydrobenzo[b][1,4]dioxin-5-yl)methyl)amino)-2-hydroxybenzoic acid (52)

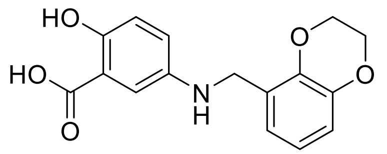

According to GP2, 5-amino-2-hydroxybenzoic acid (0.20 g, 1.30 mmol), **52a** (0.32 g, 1.95 mmol) and NaBH(OAc)$_3$ (0.55 g, 2.6 mmol) in DMF (4.3 mL) were used to give, after purification by column chromatography (hexane/EtOAc 6:4), **52** as white powder (0.26 g, 66%). **^{1}H NMR** (500 MHz, DMSO-d_6) δ 6.95 (d, J = 2.9, 1H), 6.84 (ddd, J = 9.1, 7.7, 2.7, 2H), 6.73 (m, 3H), 4.33 – 4.27 (m, 2H), 4.26 – 4.20 (m, 2H), 4.13 (s, 2H). **^{13}C NMR** (126 MHz, DMSO-d_6)172.1, 152.8, 143.1, 141.3, 141.2, 128.0, 121.8, 120.3, 120.1, 117.4, 115.5, 112.6, 111.0, 64.2, 63.8, 41.3. **HR-MS** (ESI) calcd for $C_{16}H_{16}NO_5$ [M+H]$^+$: 302.1023, found: 302.1035.

5-(((1*H*-Indol-4-yl)methyl)amino)-2-hydroxybenzoic acid (53)

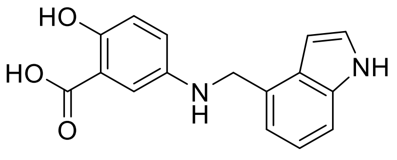

According to GP2, 5-amino-2-hydroxybenzoic acid (0.15 g, 0.98 mmol), 1*H*-indole-4-carbaldehyde (0.21 g, 1.47 mmol) and NaBH(OAc)$_3$ (0.41 g, 1.96 mmol) in DMF (3.25 mL) were used to give, after purification by column chromatography (DCM/MeOH 85:15), **53** as white powder (0.14 g, 51%). **^{1}H NMR** (500 MHz, DMSO-d_6) δ 11.10 (s, 1H), 7.33 (t, J = 2.7, 1H), 7.27 (d, J = 7.8, 1H), 7.00 (m, 3H), 6.88 (dd, J = 8.8, 2.6, 1H), 6.69 (d, J = 8.8, 1H), 6.60 (s, 1H), 4.44 (s, 2H). **^{13}C NMR** (126 MHz, DMSO-d_6) 172.2, 152.7, 141.6, 135.9, 130.9, 126.3, 124.9, 121.4, 120.8, 117.1, 112.9, 111.3, 110.1, 99.5, 45.8. **HR-MS** (ESI) calcd for $C_{16}H_{13}N_2O_3$ [M-H]$^-$: 281.0932, found: 281.0930.

5-(((1*H*-Benzo[d]imidazol-4-yl)methyl)amino)-2-hydroxybenzoic acid (54)

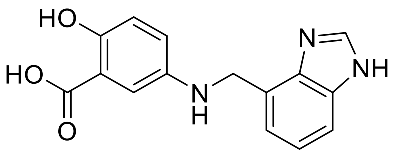

According to GP2, 5-amino-2-hydroxybenzoic acid (0.15 g, 0.98 mmol), 1*H*-benzo[*d*]imidazole-4-carbaldehyde (0.21 g, 1.47 mmol) and NaBH(OAc)$_3$ (0.41 g, 1.96 mmol) in DMF (3.25 mL) were used to give, after purification by column chromatography (DCM/MeOH 85:15) **54** as white powder (0.14 g, 50%). **^{1}H NMR** (500 MHz, DMSO-d_6) δ 8.27 (s, 1H), 7.47 (dd, J = 7.8, 0.7, 1H), 7.24 – 7.09 (m, 2H), 7.02 (d, J = 2.9, 1H), 6.89 (dt, J = 14.5, 7.2, 1H), 6.71 (dd, J = 11.6, 7.1, 1H), 4.54 (s, 2H). **^{13}C NMR** (126 MHz, DMSO-d_6) 172.1, 152.9, 141.6, 141.3, 137.6, 136.5, 127.1, 121.8, 121.76, 120.2, 117.3, 113.6, 112.8, 111.5, 43.7. **HR-MS** (ESI) calcd for $C_{15}H_{14}N_3O_3$ [M+H]$^+$: 284.1029, found: 284.1022.

5-(((1*H*-Indazol-4-yl)methyl)amino)-2-hydroxybenzoic acid (55)

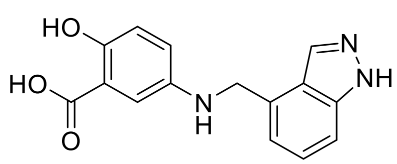

According to GP2, 5-amino-2-hydroxybenzoic acid (0.30 g, 1.96 mmol), 1*H*-indazole-4-carbaldehyde (0.43 g, 2.94 mmol) and NaBH(OAc)$_3$ (0.83 g, 3.92 mmol) in DMF (6.5 mL) were used to give, after purification by preparative HPLC (H_2O 0.1% FA/CH_3CN), **55** as white powder (0.09 g, 16%). **^{1}H NMR** (500 MHz, DMSO-d_6) δ 8.26 (d, J = 0.9, 1H), 7.39 (d, J = 8.3, 1H), 7.26 (dd, J = 8.3, 7.0, 1H), 7.07 (d, J = 6.9, 1H), 6.99 (d, J = 2.9, 1H), 6.90 (dd, J = 8.9, 3.0, 1H), 6.70 (d, J = 8.8, 1H), 4.54 (s, 2H). **^{13}C NMR** (126 MHz, DMSO-d_6) 172.1, 152.7, 141.4, 140.1, 133.0, 132.3, 125.9, 121.6, 118.2, 117.4, 112.5, 111.3, 108.7, 45.6. **HR-MS** (ESI) calcd for $C_{15}H_{14}N_3O_3$ [M+H]$^+$: 284.1029, found: 284.1024.

2-Hydroxy-5-((naphthalen-2-ylmethyl)amino)benzoic acid (56)

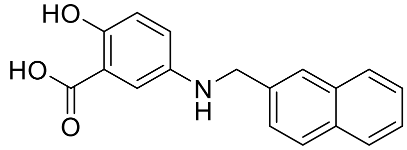

According to GP2, 5-amino-2-hydroxybenzoic acid (0.30 g, 1.96 mmol), 2-naphthaldehyde (0.46 g, 2.94 mmol) and NaBH(OAc)$_3$ (0.83 g, 3.92 mmol) in DMF (6.5 mL) were used to give, after purification by column chromatography (hexane/EtOAc 6:4) **56** as off-white powder (0.33 g, 57%). **^{1}H NMR** (500 MHz, DMSO-d_6) δ 7.92 – 7.79 (m, 4H), 7.52 (dt, = 7.8, 3.9, 1H), 7.50 – 7.40 (m, 2H), 7.00 (d, J = 2.9, 1H), 6.92 (dd, J = 8.9, 3.0, 1H), 6.72 (d, J = 8.8, 1H), 4.39 (s, 2H). **^{13}C NMR** (126 MHz, DMSO-d_6) 172.0, 152.8, 141.3, 137.8, 133.0, 132.2, 127.9, 127.6, 127.5, 126.1, 125.9, 125.3, 121.9, 117.4, 112.5, 111.5, 47.5. **HR-MS** (ESI) calcd for $C_{18}H_{14}NO_3$ [M-H]$^-$: 292.0979, found: 292.0974.

5-(((4-Bromonaphthalen-1-yl)methyl)amino)-2-hydroxybenzoic acid (58)

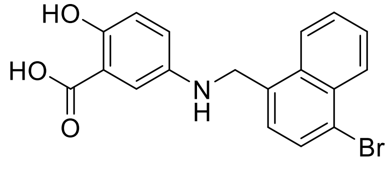

1-Bromo-4-(bromomethyl)naphthalene (0.17 g, 0.57 mmol, 1.1 eq.) was slowly added at 0 °C to a solution of 5-amino-2-hydroxybenzoic acid (0.08 g, 0.52 mmol, 1.0 eq.) and Et_3N (0.08 mL, 0.57 mmol, 1.1 eq.) in anhydrous DMF (2.8, 0.2 M). After stirring the reaction mixture for 2 h, NH_4Cl was added, and the

aqueous phase washed with EtOAc (3 x 40 mL). The combined organic phases were then washed with saturated aqueous NaCl solution (10 mL), dried over $MgSO_4$, filtered, and the solvents were evaporated under reduced pressure. The obtained crude material was further purified by column chromatography (EtOAc/*n*-Hexane = 50:50) to get **58** (28 mg, 21%) as off-brown solid. **^{1}H NMR** (300 MHz, DMSO-d_6) δ 8.21 (ddd, J = 7.8, 5.0, 1.6, 2H), 7.84 (d, J = 7.7, 1H), 7.70 (tdd, J = 13.2, 6.9, 1.5, 2H), 7.43 (d, J = 7.7, 1H), 6.99 (d, J = 2.9, 1H), 6.88 (dd, J = 8.8, 3.0, 1H), 6.71 (d, J = 8.8, 1H), 4.65 (s, 2H). **^{13}C NMR** (75 MHz, DMSO-d_6) δ 172.0, 152.8, 141.4, 135.8, 132.4, 131.2, 129.6, 127.6, 127.6, 127.1, 127.0, 125.7, 124.5, 121.6, 120.9, 117.5, 112.6, 111.1, 45.0. **HR-MS** (ESI) calcd for $C_{18}H_{15}BrNO_3$ [M+H]$^+$: 372.0229, found: 372.0224.

5-(((4-Fluoronaphthalen-1-yl)methyl)amino)-2-hydroxybenzoic acid (59)

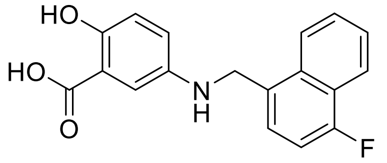

According to GP2, 5-amino-2-hydroxybenzoic acid (0.10 g, 0.65 mmol), 4-fluoro-1-naphthaldehyde (0.17 g, 0.97 mmol) and $NaBH(OAc)_3$ (0.27 g, 1.3 mmol) in DMF (2 mL) were used to give, after purification by column chromatography (EtOAc/n-Hexane = 60:40), **59** (0.068 g, 37%) as an off-white solid. **^{1}H NMR** (500 MHz, DMSO-d_6) δ 8.20 (d, J = 8.0, 1H), 8.09 (dd, J = 6.7, 2.8, 1H), 7.71 – 7.63 (m, 2H), 7.49 (dd, J = 7.8, 5.6, 1H), 7.28 (dd, J = 10.7, 7.9, 1H), 7.01 (d, J = 2.9, 1H), 6.93 (dd, J = 8.9, 3.0, 1H), 6.75 (d, J = 8.8, 1H), 4.62 (s, 2H). **^{13}C NMR** (126 MHz, DMSO-d_6) δ 172.1, 158.3, 156.3, 152.8, 141.5, 132.4, 131.4, 127.3, 126.5, 125.1, 124.2, 123.1, 121.8, 120.4, 117.5, 112.5, 111.1, 109.1, 45.0. **HR-MS** (ESI) calcd for $C_{18}H_{13}FNO_3$ [M-H]$^-$: 310.0885, found: 310.0874.

2-Hydroxy-5-(((4-methoxynaphthalen-1-yl)methyl)amino)benzoic acid (60)

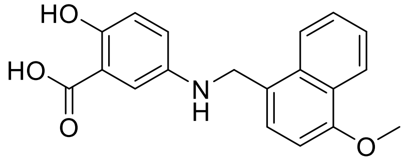

According to GP2, 5-amino-2-hydroxybenzoic acid (0.11 g, 0.75 mmol, 1.0 eq.), 4-methoxy-1-naphthaldehyde (0.21 g, 1.12 mmol) and $NaBH(OAc)_3$ (0.32 g, 1.5 mmol) in DMF (2.5 mL) were used to give, after purification by column chromatography (MeOH/DCM = 10:90), **60** as a brown solid (96 mg, 40%). **^{1}H NMR** (500 MHz, DMSO-d_6) δ 8.20 (d, J = 8.1, 1H), 8.08 (d, J = 8.3, 1H), 7.60 – 7.49 (m, 2H), 7.42 (d, J = 7.9, 1H), 7.03 (d, J = 2.9, 1H), 6.95 – 6.87 (m, 2H), 6.73 (d, J = 8.8, 1H), 4.54 (s, 2H), 3.95 (s, 3H). **^{13}C NMR** (126 MHz, DMSO-d_6) δ 172.2, 163.1, 154.2, 152.8, 141.6, 132.1, 126.6, 125.7, 125.2, 125.1, 123.8, 122.0, 121.6, 117.4, 112.9, 111.1, 103.7, 55.5, 45.3. **HR-MS** (ESI) calcd for $C_{19}H_{16}NO_4^-$ [M-H]$^-$: 322.1085, found: 322.1069.

5-((4-Bromobenzyl)amino)-2-hydroxybenzoic acid (61)

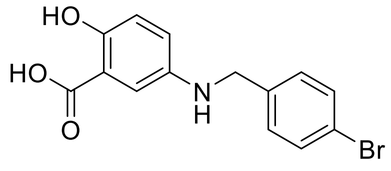

According to GP2, 5-amino-2-hydroxybenzoic acid (0.10 g, 0.65 mmol), 4-bromobenzaldehyde (0.18 g, 0.97 mmol) and $NaBH(OAc)_3$ (0.27 g, 1.3 mmol) in DMF (2 mL) were used to give, after purification by column chromatography (DCM/MeOH, 95:5), **61** (0.076 g, 36%) as a yellow solid. **^{1}H NMR** (300 MHz, DMSO-*d6*) δ 7.53 – 7.47 (m, 2H), 7.30 (d, *J* = 8.4, 2H), 6.92 (d, *J* = 2.9, 1H), 6.81 (dd, *J* = 8.8, 2.9, 1H), 6.68 (d, *J* = 8.8, 1H), 4.18 (s, 2H). **^{13}C NMR** (75 MHz, DMSO-*d6*) δ 172.0, 152.9, 140.9, 139.8, 131.1, 129.4, 121.5, 119.5, 117.3, 113.0, 111.6, 46.5. **HR-MS** (ESI) calcd for $C_{14}H_{13}BrNO_3^+$ [*M*+H]$^+$: 322.0073, found: 322.0059.

5-((4-Bromo-2-isopropylbenzyl)amino)-2-hydroxybenzoic acid (62)

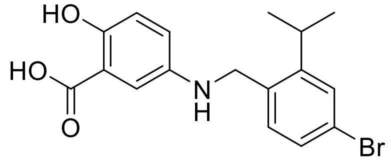

According to GP2, 5-amino-2-hydroxybenzoic acid (0.22 g, 1.42 mmol), 4-bromo-2-isopropylbenzaldehyde (0.58 g, 2.13 mmol) and $NaBH(OAc)_3$ (0.59 g, 2.8 mmol) in DMF (5 mL) were used to give, after purification by column chromatography (Hexan/EtOAc, 7:3), **62** as brown solid (0.035 g, 35%). **^{1}H NMR** (500 MHz, DMSO-*d6*) *δ* 7.44 (d, *J* = 1.9, 1H), 7.32 (dd, *J* = 8.2, 1.9, 1H), 7.24 (d, *J* = 8.3, 1H), 6.91 (d, *J* = 2.8, 1H), 6.87 (dd, *J* = 8.8, 2.9, 1H), 6.74 (d, *J* = 8.8, 1H), 4.18 (s, 2H), 3.21 (dq, *J* = 13.5, 6.7, 1H), 1.22 (s, 3H), 1.20 (s, 3H). **^{13}C NMR** (126 MHz, DMSO-*d6*) *δ* 172.0, 152.8, 149.4, 141.2, 135.5, 130.2, 128.4, 128.0, 121.8, 120.5, 117.5, 112.4, 110.8, 44.4, 27.9, 23.4. **HR-MS** (ESI) calcd for $C_{17}H_{19}BrNO_3$ [*M*+H]$^+$: 364.0543, found: 364.0527.

5-(4-(4-Bromophenyl)-1*H*-1,2,3-triazol-1-yl)-2-hydroxybenzoic acid (63)

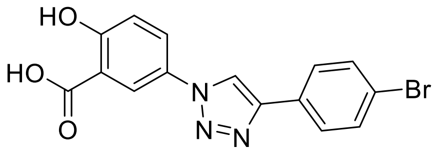

According to GP5, **37a** (0.05 g, 0.28 mmol) and 1-bromo-4-ethynylbenzene (0.051 g, 0.28 mmol) in 3 mL (H_2O:*t*-ButOH 1:1), sodium ascorbate (5.52 mg, 0.028 mmol) and copper (II) sulfate pentahydrate (0.7 mg, 0.0028 mmol) were used to give, after preparative HPLC purification, compound **64** as beige powder (0.038 g, 38%). **^{1}H NMR** (500 MHz, DMSO-*d6*) *δ* 9.35 (s, 1H), 8.28 (d, *J* = 2.7, 1H), 8.06 (dd, *J* = 8.9, 2.8, 1H), 7.90 (d, *J* = 8.5, 2H), 7.70 (d, *J* = 8.5, 2H), 7.21 (d, *J* = 8.9, 1H). **^{13}C NMR** (126 MHz, DMSO-*d6*) *δ* 170.8, 160.9, 146.2, 132.0, 129.6, 128.5, 127.3, 127.3, 121.6, 121.2, 120.1, 118.7, 114.4. **HR-MS** (ESI) calcd for $C_{15}H_9BrN_3O_3$ [*M*-H]$^-$: 357.9833, found: 357.9819.

5-(4-(3,5-*di*Chlorophenyl)-1*H*-1,2,3-triazol-1-yl)-2-hydroxybenzoic acid (64)

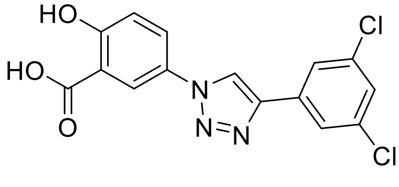

According to GP5, **37a** (0.05 g, 0.28 mmol) and 1,3-dichloro-5-ethynylbenzene (0.048 g, 0.28 mmol) in 3 mL (H_2O:*t*-BuOH 1:1), sodium ascorbate (5.52 mg, 0.028 mmol) and copper (II) sulfate pentahydrate (0.7 mg, 0.0028 mmol) were used to give,

after preparative HPLC purification, compound **64** as white powder (0.049 g, 50%). **^{1}H NMR** (500 MHz, DMSO-*d_6*) δ 9.48 (s, 1H), 8.26 (d, *J* = 2.8, 1H), 8.05 (dd, *J* = 8.9, 2.8, 1H), 7.97 (d, *J* = 1.9, 2H), 7.62 (t, *J* = 1.9, 1H), 7.22 (d, *J* = 8.9, 1H). **^{13}C NMR** (126 MHz, DMSO-*d_6*) δ 170.8, 161.0, 144.7, 134.9, 133.7, 128.3, 127.5, 127.2, 123.6, 121.5, 121.2, 118.8, 114.1. **HR-MS** (ESI) calcd for $C_{15}H_8Cl_2N_3O_3^-$ [*M*-H]$^-$: 347.9948, found: 347.9934.

4-(4-(3,5-*di*Chlorophenyl)-1*H*-1,2,3-triazol-1-yl)-2-hydroxybenzoic acid (65)

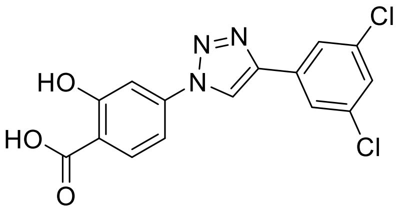

According to GP5, **38a** (0.05 g, 0.28 mmol) and 1,3-dichloro-5-ethynylbenzene (0.048 g, 0.28 mmol) in 3 mL (H_2O:*t*-ButOH 1:1) were used to give after, preparative HPLC purification, compound **66** as beige powder (0.019 g, 19%). **^{1}H NMR** (500 MHz, Acetone) δ 9.28 (s, 1H), 8.11 (d, *J* = 8.6, 1H), 7.96 (d, *J* = 1.9, 2H), 7.59 (dd, *J* = 8.6, 2.1, 1H), 7.55 (d, *J* = 2.0, 1H), 7.47 (t, *J* = 1.8, 1H). **^{13}C NMR** (126 MHz, Acetone) δ 171.9, 163.8, 146.3, 142.8, 136.2, 134.8, 133.3, 128.6, 124.8, 121.1, 113.4, 111.2, 108.6. **HR-MS** (ESI) calcd for $C_{15}H_8Cl_2N_3O_3$ [*M*-H]$^-$: 347.9948, found: 347.9933.

5-((2-Chloro-4-(trifluoromethyl)benzyl)oxy)-2-hydroxybenzoic acid (66)

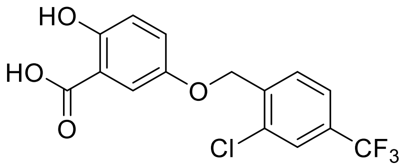

According to GP1, 2,5-dihydroxybenzoic acid (0.09 g, 0.58 mmol, 1 eq), 1-(bromomethyl)-2-chloro-4-(trifluoromethyl)benzene (0.16 g, 0.58 mmol, 1 eq) and NaH (0.004 g, 0.11 mmol) in DMF (2.0 mL) were used to give, after flash chromatography (hexane/EtOAc 1:9), **66** as white powder (0.07 g, 35%). **^{1}H NMR** (500 MHz, DMSO-*d_6*) δ 7.94 (s, 1H), 7.81 (dd, *J*=23.1, 8.1, 2H), 7.37 (d, *J* = 3.2, 1H), 7.27 (dd, *J* = 9.0, 3.2, 1H), 6.93 (d, *J* = 9.0, 1H), 5.21 (s, 2H). **^{13}C NMR** (126 MHz, DMSO-*d_6*) δ 171.4, 155.8, 150.1, 139.3, 133.0, 130.3, 126.2 (q, *J* = 3.6) 124.4, 124.3 (q, *J* = 3.7), 124.1, 118.3, 114.1, 112.8, 67.0. **^{19}F NMR** (470 MHz, DMSO-*d_6*) δ -61.13. **HR-MS** (ESI) calcd for $C_{15}H_9ClF_3O_4$ [*M*-H]$^-$: 345.0147, found: 345.0138.

5-((3,5-di-*tert*-Butylbenzyl)oxy)-2-hydroxybenzoic acid (67)

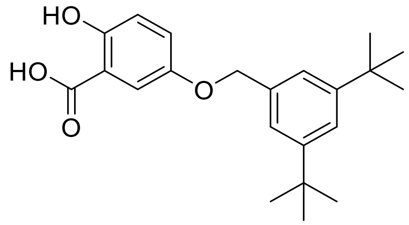

According to GP1, 2,5-dihydroxybenzoic acid (0.20 g, 1.3 mmol), 1-(bromomethyl)-3,5-di-*tert*-butylbenzene (0.36 g, 1.3 mmol) and NaH (0.13 g, 3.24 mmol) in DMF (2.0 mL), were used to give after flash chromatography (hexane/EtOAc 1:9) **67** as white powder (0.07 g, 15%). **^{1}H NMR** (500 MHz, DMSO-*d_6*) δ 7.37 (d, *J* = 3.2, 1H), 7.35 (t, *J* = 1.8, 1H), 7.27 (d, *J* = 1.8, 2H), 7.22 (dd, *J* = 9.0, 3.2, 1H), 6.90 (d, *J* = 9.0, 1H), 5.01 (s, 2H), 1.28 (s, 18H). **^{13}C NMR** (126 MHz, DMSO-*d_6*) δ 171.6, 155.5, 150.7, 150.3, 136.0, 124.3, 122.1, 121.4, 118.1, 113.9, 112.7, 70.7, 34.5, 31.2. **HR-MS** (ESI) calcd for $C_{22}H_{27}O_4$ [*M*-H]$^-$: 355.1915, found: 355.1908.

2-((*tert*-Butoxycarbonyl)amino)-5-((3,5-di-*tert*-butylbenzyl)oxy)benzoic acid (68)

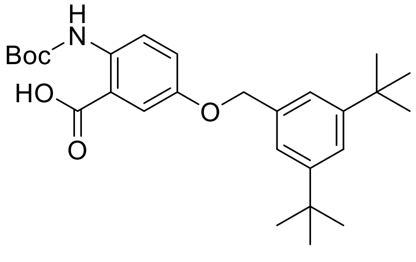

According to GP1, 2-*tert*-butoxycarbonylamino-5-hydroxy-benzoic acid (0.05 g, 0.2 mmol), and 1-(bromomethyl)-3,5-di-*tert*-butylbenzene (0.6 g, 0.21 mmol), were used to give, after purification by column chromatography (hexane/EtOAc 9:1), **68** as light-yellow powder (0.03 g, 33%). **^{1}H NMR** (500 MHz, DMSO-d_6) δ 10.18 (s, 1H), 8.17 (d, J=9.1, 1H), 7.55 (d, J=2.9, 1H), 7.35 (s, 1H), 7.32 – 7.23 (m, 3H), 5.06 (s, 2H), 1.47 (s, 9H), 1.28 (s, 18H). **^{13}C NMR** (126 MHz, DMSO-d_6) δ 169.2, 152.6, 152.2, 150.4, 135.9, 135.0, 122.1, 121.7, 121.4, 119.9, 116.4, 115.9, 79.8, 70.4, 34.5, 31.3, 28.0. **HR-MS** (ESI) calcd for $C_{27}H_{36}NO_5$ [M-H]$^-$: 454.2599, found: 454.2592.

2-Amino-5-((3,5-di-*tert*-butylbenzyl)oxy)benzoic acid (68a)

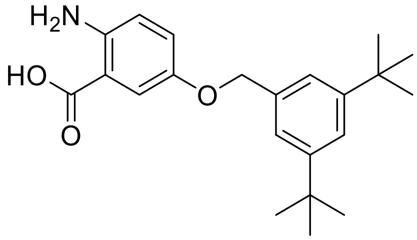

To a stirred solution of **68** (0.3 g, 0.63 mmol, 1 eq) in dry DCM (1.2 mL) TFA (0.5 mL) was added. The reaction mixture was stirred at room temperature on. The excess reagent and solvent were removed under reduced pressure. The crude material was neutralized with sat. $NaHCO_3$ solution to pH=5 and extracted with DCM (3×10 mL) and saturated aqueous NaCl solution (2×10 mL). The combined organic layers were dried over Na_2SO_4, filtered and concentrated *in vacuo* to obtain **68a** as a light yellow solid (0.2 g, 0.63 mmol, 90%). **^{1}H NMR** (500 MHz, DMSO-d_6) δ 8.1-8.7 (m, 2H), 7.34 (t, J = 1.8, 1H), 7.30 (d, J = 3.1, 1H), 7.25 (d, J = 1.8, 2H), 7.02 (dd, J = 3.1, 9.0, 1H), 6.70 (d, J = 9.0, 1H), 4.93 (s, 2H), 1.28 (s, 18H). **^{13}C NMR** (126 MHz, DMSO-d_6) δ 169.3, 150.3, 148.1, 146.5, 136.5, 123.8, 122.1, 121.3, 117.8, 114.6, 109.3, 70.7, 34.5, 31.3. LC−MS calcd for $C_{22}H_{28}NO_3$ [M−H]$^-$: 354.2, found 354.2.

5-((3,5-di-*tert*-Butylbenzyl)oxy)-2-(methylsulfonamido)benzoic acid (69)

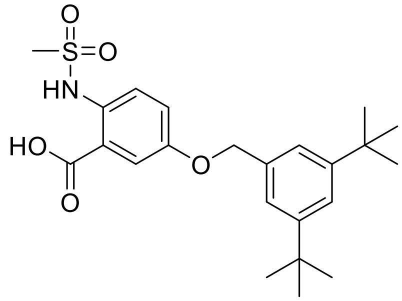

To a stirred solution of **68a** (0.08 g, 0.22 mmol), and pyridine (0.045 mL, 0.56 mmol, 2.5 eq) in DCM (0.75 mL), mesyl chloride (0.02 mL, 0.27 mmol, 1.2 eq) was added at 0°C. The reaction mixture was stirred at rt for 24 h. The resulting suspension was quenched with of 1N HCl (10 mL), and the aqueous phase was extracted with DCM (3x10 mL). The combined organic layers were dried over Na_2SO_4, filtered, concentrated *in vacuo* and purified by preparative HPLC to give **69** as white solid (0.015 g, 16%). **^{1}H NMR** (500 MHz, $CDCl_3$) δ 7.43 (t, J = 1.7, 1H), 7.19 (d, J = 1.7, 2H), 7.15 (d, J = 8.8, 1H), 7.10 (t, J = 4.0, 1H), 6.98 (dd, J = 8.8, 2.9, 1H), 4.97 (dd, 2H), 3.62 (s, 3H), 1.32 (s, 18H). **^{13}C NMR** (126 MHz, $CDCl_3$) δ 167.2, 160.5, 151.4, 135.4, 134.4, 130.8,

124.9, 122.9, 122.4, 118.6, 114.1, 71.6, 42.6, 35.0, 31.5, 29.8. **HR-MS** (ESI) calcd for $C_{23}H_{30}NO_5S$ $[M\text{-}H]^-$: 432.1850, found: 432.1849.

2-Acetamido-5-((3,5-di-*tert*-butylbenzyl)oxy)benzoic acid (70)

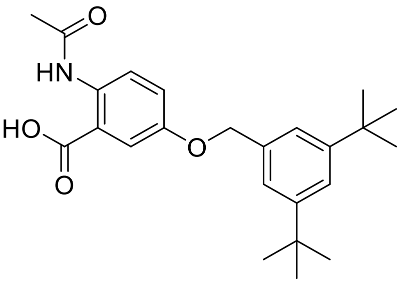

To a stirred solution of **68a** (0.08 g, 0.225 mmol) and pyridine (0.035 ml, 0.45 mmol, 2 eq) in DCM (0.8 ml), acetychloride (0.020 ml, 0.25 mmol, 1.2 eq) was added at 0 °C. The reaction mixture was stirred at rt on. The resulting suspension was quenched with 1N HCl (10 mL), and the aqueous phase extracted with DCM (3x10 mL). The combined organic layers were dried over Na_2SO_4, filtered, concentrated *in vacuo* and purified by preparative HPLC to give **70** as white solid (0.040 g, 44%). **^{1}H NMR** (500 MHz, MeOD-d_4) δ = 8.39 (d, J=9.1, 1H), 7.66 (d, J=3.1, 1H), 7.40 (t, J=1.7, 1H), 7.29 (d, J=1.7, 2H), 7.20 (dd, J=9.2, 3.1, 1H), 5.07 (s, 2H), 2.16 (s, 3H), 1.32 (s, 18H) **^{13}C NMR** (126 MHz, MeOD-d_4) δ 171.1, 170.9, 155.6, 152.2, 137.4, 135.7, 123.3, 123.2, 122.9, 122.2, 119.3, 117.7, 72.2, 35.7, 31.9, 24.8. **HR-MS** (ESI) calcd for $C_{24}H_{30}NO_4$ $[M\text{-}H]^-$: 396.2180, found: 396.2180.

2.2 Table S1. Chemical structures and purity of salicylic acid derivatives provided by BASF (5–24)

All the compounds have more than 95% purity.

Cmpd	R	MS-HPLC
**5**	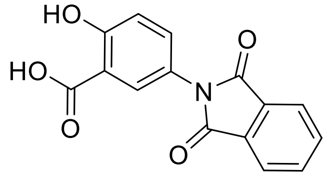	rt: 2.79 min $(M\text{-}H)^-$ = 282<br>168903-55-7<br>Enamine BBV-030341
**6**	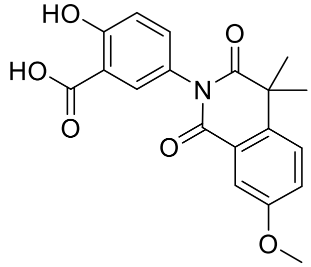	rt: 3.1 min $(M\text{+}H)^+$ = 356
**7**	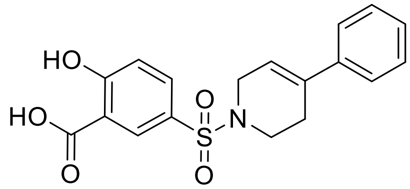	rt: 3.5 min $(M\text{-}H)^-$ = 358<br>863417-63-4<br>Enamine Z45578234

**8**	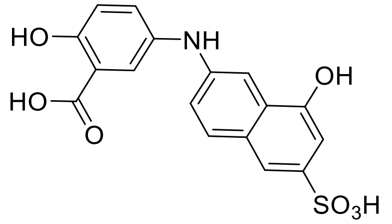	rt: 2.2 min (*M*-H)$^-$ = 374<br>AKOS030557818
**9**	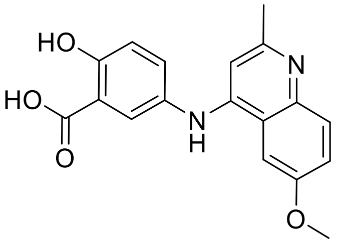	rt: 2.4 min (*M*+H)$^+$ = 325<br>296772-88-8<br>Aurora 174.807.521
**10**	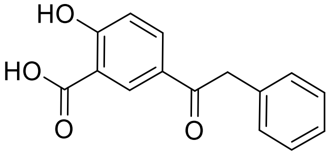	rt: 2.8 min (*M*+H)$^+$ = 257<br>33555-64-5<br>Chemieliva CB0371743
**11**	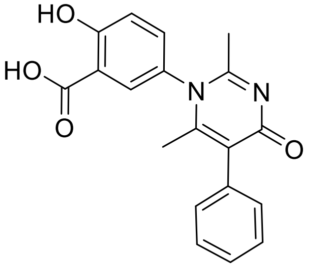	rt: 2.3 min (*M*+H)$^+$ = 337<br>438622-84-5<br>Chemdiv Y510-6398
**12**	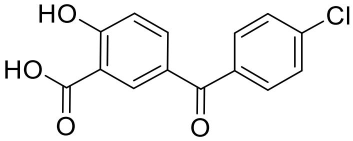	rt: 3.2 min (*M*-H)$^-$ = 275<br>92524-89-5<br>Chemieliva CC0130042
**13**	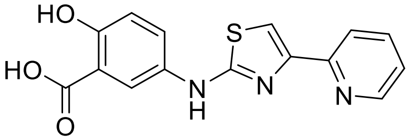	rt: 2.07 min (*M*+H)$^+$ = 314<br>696653-95-9<br>ChemDiv 8015-8805
**14**	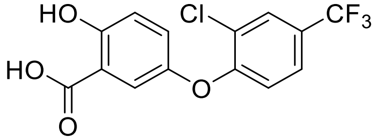	rt: 4.1 min (*M*-H)$^-$ = 331<br>78068-86-7<br>Aurora 209.700.447
**15**	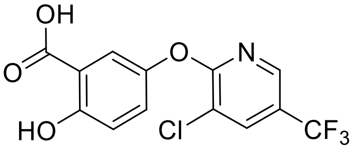	rt: 3.6 min (*M*-H)$^-$ = 332<br>178948-18-0<br>EP716073
**16**	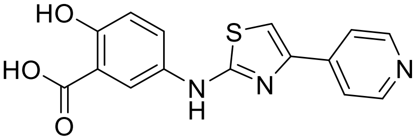	rt: 2.06 min (*M*+H)$^+$ = 314<br>708997-84-6<br>Aurora 174.799.732

**17**	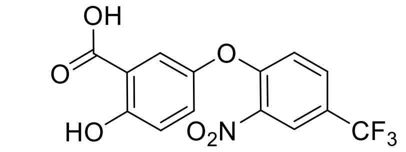	rt: 3.5 min $(M\text{-H})^- = 342$<br>178948-19-1<br>EP716073
**18**	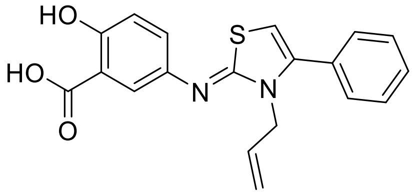	rt: 2.7 min $(M\text{-H})^- = 351$<br>301649-02-5<br>ChemDiv 4052-1387
**19**	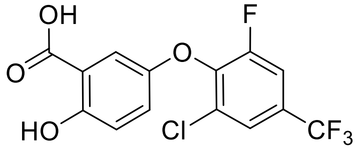	rt: 4.3 min $(M\text{-H})^- = 349$<br>178948-20-4<br>EP716073
**20**	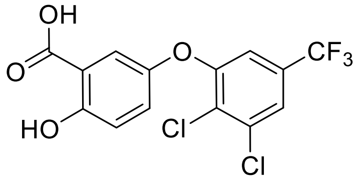	rt: 4.0 min $(M\text{-H})^- = 349$<br>178948-21-5<br>EP716073
**21**	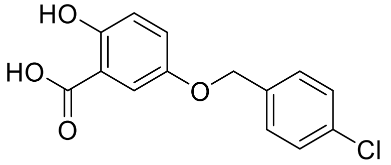	rt: 3.0 min $(M\text{-H})^- = 277$<br>1215858-20-0<br>Aurora 181.983.269
**22**	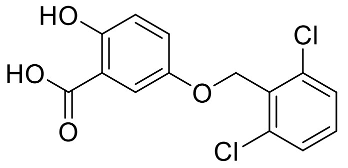	rt: 3.7 min $(M\text{-H})^- = 311$<br>1993176-17-2<br>Aurora 181.988.117
**23**	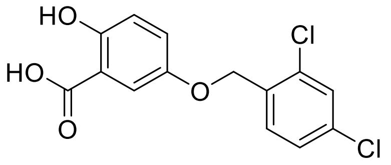	rt: 3.8 min $(M\text{-H})^- = 311$<br>1992955-83-5<br>Aurora 181.983.270
**24**	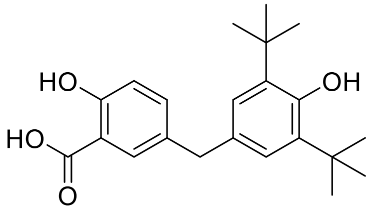	rt: 4.2 min $(M\text{-H})^- = 311$<br>312747-09-4<br>ChemDiv 0471-0001

3.0 Molecular docking

The protein structure used for docking was obtained from the molecular dynamics (MD) trajectory, derived from unbiased coarse-grained MD simulations of compound 1 bound to the ECF-FolT2 transporter, starting from the crystal structure of ECF-FolT2 (PDB ID: 5JSZ). Docking studies were performed in MOE (version 2024.0601). For compounds **67** and **68**, five poses were generated, and the pose selected was the one that best aligned with the binding orientation of compound 1. Protein–ligand interactions of the resulting complexes were subsequently analyzed in MOE.

4.0 Biological results

4.1 ECF-T folate uptake assay

A bacterial uptake assay using *Lactobacillus casei* as a model microorganism and tritium-labeled folic acid (Moravek Biochemicals, Brea, CA, USA) as the substrate to be quantified was conducted as described previously by us.[2] All compounds were tested at 200 μM and for the most active representatives, IC_{50} values were determined.

Tables S2–S4. Inhibitory potency and IC_{50} determination of ECF compounds in the *Lactobacillus casei* whole-cell assay.

Table S2. Compounds 5–24.

Compounds	% Inh. at 200 μM ± S.E.M.[a]	IC_{50} (μM)± S.E.M.[a]
**5**	n. i.	-
**6**	n. i.	-
**7**	45.7 ± 3.9	-
**8**	11 ± 4,3	-
**9**	28.9 ± 19.2	-
**10**	n.i.	-
**11**	n. i.	-
**12**	n. i.	-
**13**	24 ± 19.1	-
**14**	56.0 ± 3.9	-
**15**	32.0 ± 11.5	-
**16**	n.i.	-
**17**	36.3 ± 6.9	-
**18**	23.9 ± 9.3	-
**19**	64 ± 0.4	-
**20**	98.1 ± 1.1	63.6±14.25

**21**	36.6 ± 5.1	-
**22**	61.5 ± 6.0	-
**23**	66.1 ± 3.4	-
**24**	100 ± 1.0	25.52 ± 20.2

[a]S.E.M. derived as mean values of two or more determinations;
n.i. = no inhibition when inhibition < 10%.

Table S3. Compounds 1, 3 and 25–33.

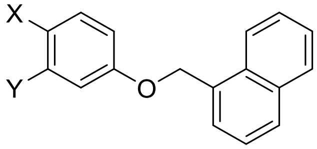

Compounds	X	Y	% Inh. @ 200 μM ± S.E.M.[a]	IC_{50} (μM) ± S.E.M.[a]
**1**	OH	COOH	42.7 ± 8.9	-
**3**	NHBoc	COOH	95.4 ± 1.9	49.2 ± 3.4
**25**	NH_2	COOH	58.5 ± 3.7	-
**26**	H	COOH	37.6 ± 1.4	-
**27**	CH_3	COOH	21.6 ± 3.5	-
**28**	OH	CH_2COOH	n.i.	-
**29**	OH	$(CH_2)_3COOH$	n.i.	-
**30**	OH	$COOCH_3$	n.i.	-
**31**	OH	$CONH_2$	n.i.	-
**32**	OCH_3	COOH	n.i.	-
**33**	OH	CH_2OH	n.i.	-

Table S4. Compounds 44–56.

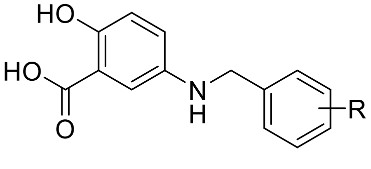

44-51

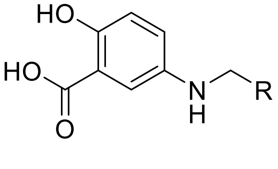

52-56

Compounds	R	% Inh. at 200 μM ± S.E.M.[a]
**44**	H	6.5±1.4
**45**	2,3-dimethyl	41.4± 12.2
**46**	2-methylsulfonyl	n.i.
**47**	2-phenyl	43.1±5.9
**48**	2-benzyl	40.4 ±15

**49**	2-phenoxy	27.6 ± 23.8
**50**	2-phenylthio	59.3 ±1.4
**51**	2-phenlysulfonyl	n.i.
**52**	2,3-*di*hydrobenzo[1,4]dioxin-5yl	n.i.
**53**	1*H*-indolyl-4yl	n.i.
**54**	1*H*-benzimidazol-4yl	n.i.
**55**	1*H*-indazol-4yl	n. i.
**56**	2-naphthayl	29.6 ± 5.1

[a]S.E.M. derived as mean values of at least two replicates;
n.i. = no inhibition when inhibition < 10%.

4.2 Radiolabeled folate and pantothenate transport assay

To determine the inhibition of the transportation of vitamins *in vitro*, uptake assays were performed with ECF-FolT2 and ECF-PanT from *L. delbrueckii* in proteoliposomes.[5] Freshly purified ECF-FolT2 were reconstituted in proteoliposomes, using a protein to lipid ratio of 1:250 (*w/w*). Proteoliposomes were thawed, supplemented with 5 mM Mg^{2+}-ATP (full activity) or 5 mM Mg^{2+}-ADP (negative control) or 5 mM Mg^{2+}-ATP plus compounds. A final concentration of 10% (*v/v*) of DMSO were supplemented to maintain the solubility of the compounds. Subsequently, the proteoliposomes were flash-frozen in liquid nitrogen and thawed under room temperature for three cycles to enclose the nucleotides and the compounds into the proteoliposome lumen, followed by extruding for eleven times through a 400 nm polycarbonate filter. To remove the exterior nucleotides, the proteoliposomes were diluted 32 times, centrifuged at 285.775 g for 45 min under 4 °C, and resuspended with 50 mM KPi (pH 7.5) containing 10% (*v/v*) DMSO. The external presence of the compounds was consistent to the inner side of the proteoliposome lumen. Buffers containing 50 mM *K*Pi (pH 7.5), 10% (*v/v*) DMSO, 100 nM folate (95 nM cold folate and 5 nM radiolabeled [3, 5, 7, 9-^{3}H] folate (American Radiolabeled Chemicals)) were prepared, the presence of compounds were supplemented accordingly. Proteoliposomes prepared as described above were added to the buffer and incubated at 30 °C. The reaction was incubated for 15 s, 1 min, 2 min, 3 min and 4 min, after each time point 2 ml of pre-cold 50 mM KPi (pH 7.5) were added to stop the reaction, then the buffer was rapidly filtered over a BA-85 nitrocellulose filter. The filter was washed with 2 mL of pre-chilled 50 mM KPi (pH 7.5) once more, and was dissolved in 1.8 mL Filter Count scintillation liquid (PerkinElmer). The levels of radioactivity were determined using a PerkinElmer Tri-Carb 280. To obtain percent inhibition values for each sample, their activities were related to those of Mg^{2+}-ATP controls. At least two independent measurements were performed for each compound. For determination of the IC_{50} values, the uptake assays were prepared and performed as described above. However, the proteoliposomes were loaded with concentrations of the compounds ranging from 1×10^{-3} to 1 μM in the presence of 10% (*v/v*) of DMSO. The IC_{50} estimation values were fitted with Inhibitor-Response equation using Prism version 5.01 (GraphPad Inc).

The preparation of proteoliposomes and the uptake assay of ECF-PanT were performed using the same protocol. The substate for ECF-PanT were 100 nM pantothenate (95 nM cold pantothenate and 95 nM radiolabeled D-[2,3-^{3}H] pantothenic sodium salt (American Radiolabeled Chemicals).

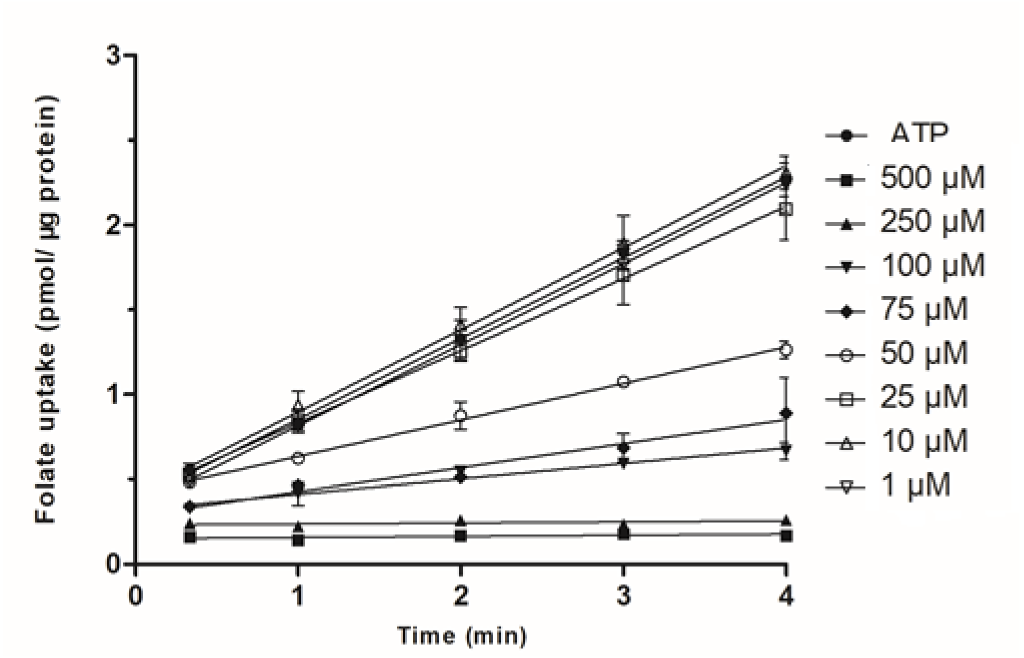

Figure S1. ECF-FolT2 uptake assay for compound **67**.

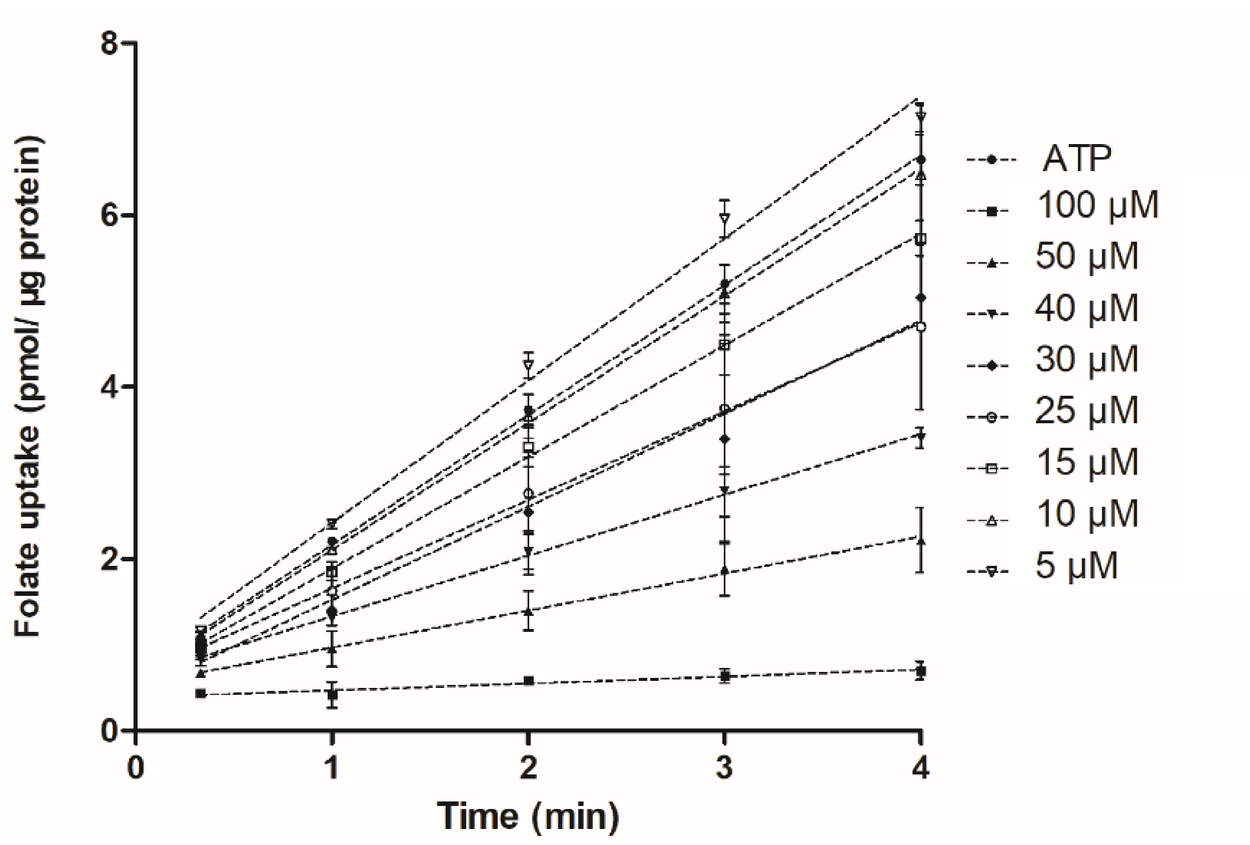

Figure S2. ECF-FolT2 uptake assay for compound **68**.

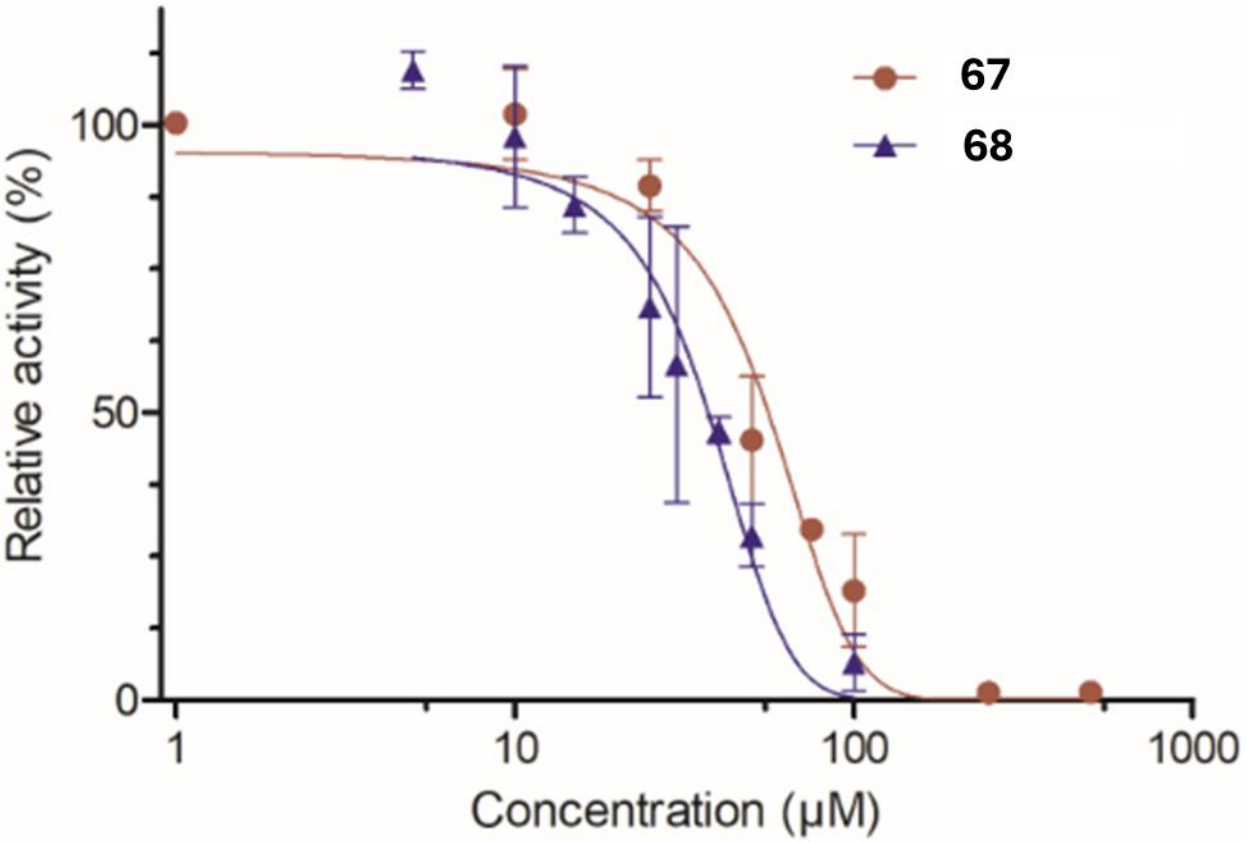
67
68
100
50
0
Relative activity (%)
1
10
100
1000
Concentration (µM)

Table S5 shows the percentage of inhibition for compound **67** at 40 and 100 μM for both proteins ECF-FolT2 and ECF-PanT.

Table S5. Inhibitory profile of compound **67** against ECF-FolT2 and ECF-PanT.

Compound	ECF-FolT2		ECF-PanT	
	@40μM	@100μM	@40μM	@100μM
**67**	65.4%	29.2%	61.8%	65.4%

4.3 Cytotoxicity assay

To obtain information regarding the toxicity of compounds **67** and **68**, their impact on the viability of human cells was investigated. HepG2 cells ($2x10^5$ cells per well) were seeded in 24-well, flat-bottomed culture plates. Twenty-four hours after seeding the cells, the incubation was started by the addition of compounds in a final DMSO concentration of 1%. Duplicates were prepared for each compound concentration. Epirubicin and doxorubicin were used as positive controls (each at 1 μM), and rifampicin was used as a negative control (at 100 μM). The living cell mass was determined 48 h after treatment with compounds by adding 0.1 volumes of 3-(4,5-dimethylthiazol-2-yl)-2,5-diphenyltetrazolium bromide (MTT) solution (5 mg per mL sterile PBS) (Sigma, St. Louis, MO) to the wells. After incubating the cells for 30 min at 37 °C (atmosphere containing 5% CO2), MTT crystals were dissolved in a solution containing 10% SDS and 0.5% acetic acid in DMSO. The optical density (OD) of the samples was determined photometrically at 570 nm in a FLUOstar Omega plate reader (BMG labtech, Ortenberg, Germany). To obtain percent inhibition values for each sample, their ODs were related to those of DMSO controls. At least two independent measurements were performed for each compound. The calculation of IC_{50} values was performed by plotting the percent inhibition vs. the concentration of inhibitor on a semi-log plot. From this the molar concentration causing 50% inhibition was calculated.

4.4 Evaluation of MIC activities

All microorganisms were obtained from the German Collection of Microorganisms and Cell Cultures (DSMZ) or the American Tpye Culture Collection (ATCC) and were handled according to standard procedures. Bacteria were inoculated into tryptic soy broth (TSB) to obtain a final inoculum of 10^5 colony-forming units (CFU)/mL.

The tested compounds were prepared as DMSO stocks (20 mM). Serial dilutions of derivatives in the growth medium (0.06 to 128 μM) were prepared in sterile 96-well plates and the bacterial suspensions were added. Growth inhibition was assessed after static incubation at 37 °C for 24 h.

Streptococcus pneumoniae was grown at 5% CO_2. Minimum inhibitory concentrations (MIC) are defined as the lowest compound concentration where no visible growth is observed.

5.0 ECF conservedness strain list

Isolate ID	GPSC	Serotype	ENA accession number
PBCN0041	124	14	ERS225605
PBCN0085	2705	33A/F	ERS225628
PBCN0120	162	9V	ERS225654
PBCN0133	176	6B	ERS225665
PBCN0150	304	1	ERS225677
PBCN0173	218	12F	ERS225691
PBCN0230	97	10A	ERS225725
PBCN0240	180	3	ERS225731
PBCN0262	36	23F	ERS225747
PBCN0272	433	22F	ERS225756
PBCN0315	60	33F	ERS225782
PBCN0316	306	1	ERS225783
PBCN0322	247	4	ERS225789
PBCN0356	62	11A	ERS225814
PBCN0364	53	8	ERS225822
PBCN0381	162	19F	ERS225837
PBCN0392	1379	6C	ERS225847
PBCN0431	446	35B	ERS225877
PBCN0433	9	14	ERS225879
PBCN0442	230	19A	ERS225886
PBCN0446	191	7F	ERS225889
PBCN0449	667	19A	ERS225891
PBCN0457	42	23A	ERS225899
PBCN0461	66	9N	ERS225902
PBCN0477	989	12A/F	ERS225917
PBCN0488	439	23B	ERS225927
PBCN0490	66	9N	ERS225929

GPSC = Global Pneumococcal Sequence Cluster; ENA = European Nucleotide Archive; PBCN = Pneumococcal Bacteremia Collection Nijmegen.

5.1 MIC determination

Growth curves of wild type *Streptococcus pneumoniae* D39V when treated with ranging doses of **67** and **68** compounds.

Figure S3 (A): **67**; Figure S3 (B): **68**.

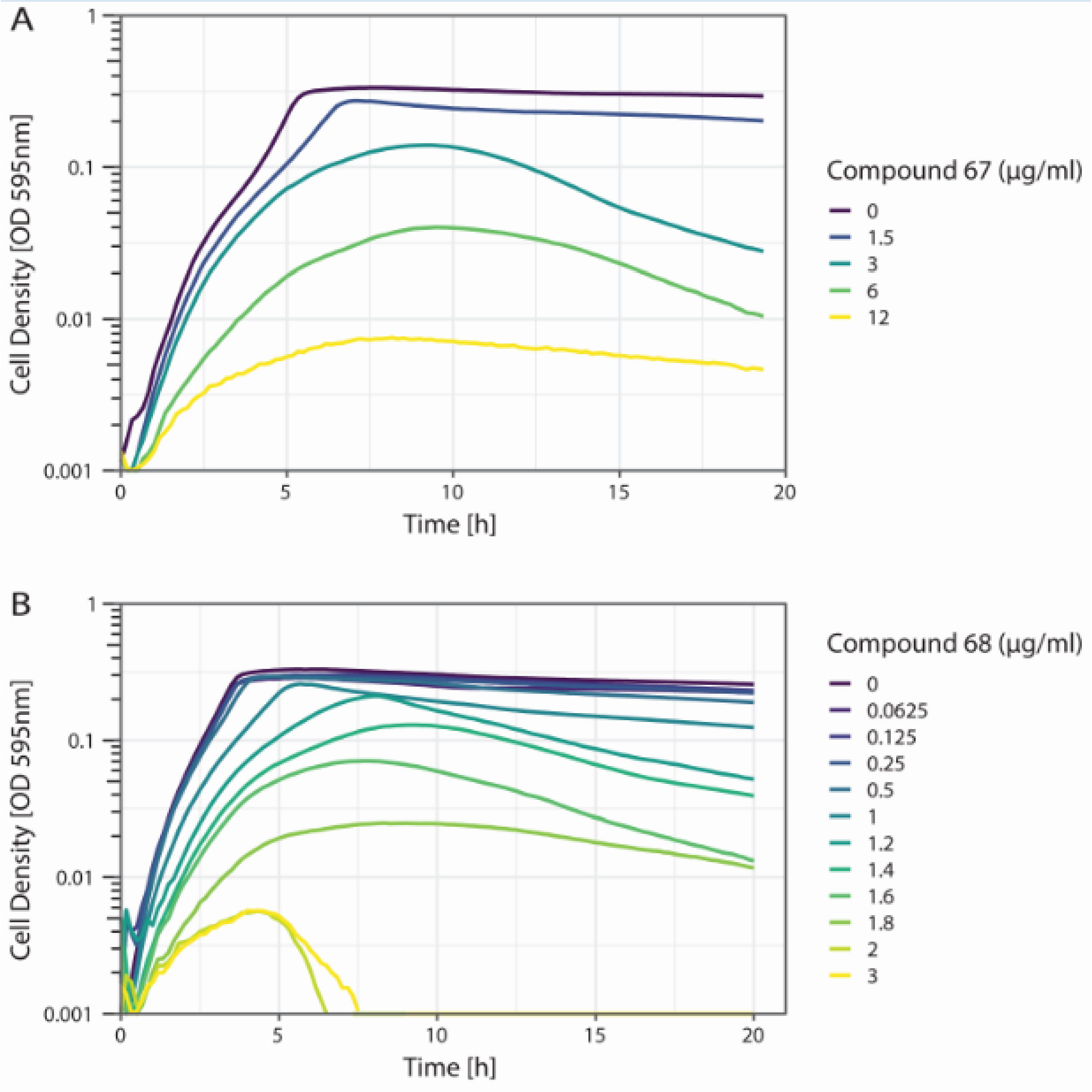

5.2 Untreated culture of *S. pneumoniae*

Shutdown of expression of presumed target (the ECF transporter in *S. pneumoniae*) has less impact on bacterial growth initiation in the absence of an inhibitor compound (Figure S4)

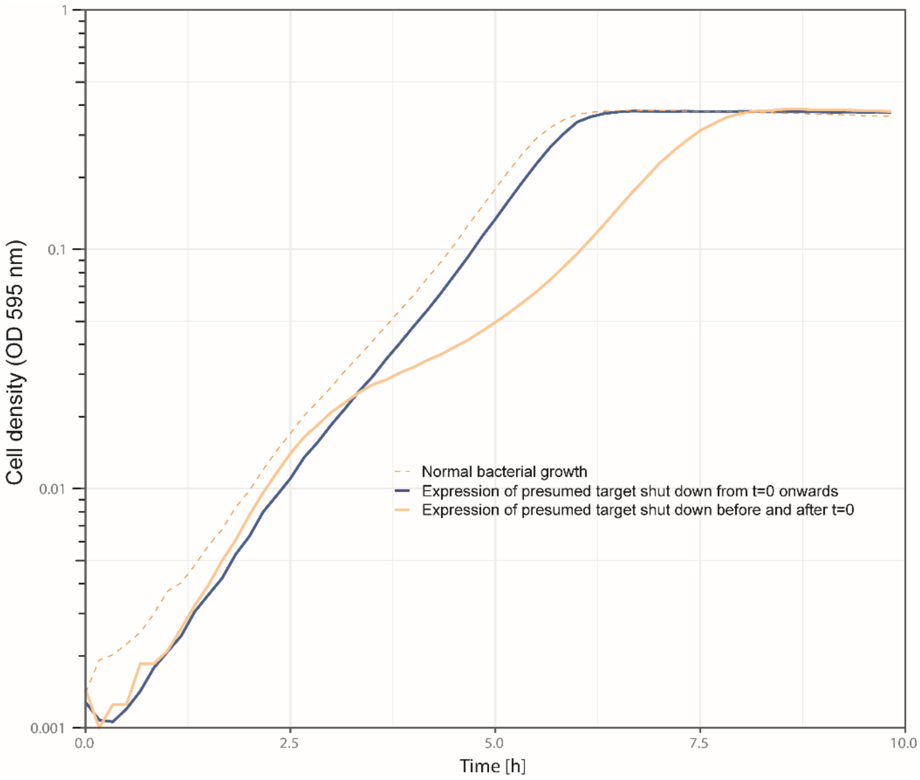

6.0 *In vitro* ADME assays

Lipophilicity Determination

$LogD_{7.4}$ was analyzed using an HPLC−based method. The UV retention time of reference compounds with known $LogD_{7.4}$ was determined and plotted toward their $LogD_{7.4}$. Linear regression was used to determine the $LogD_{7.4}$ of unknown compounds. Analysis was performed using a Vanquish Flex HPLC system with variable wavelength detector (Thermo Fisher, Dreieich, Germany) with the following conditions: EC150/2 NUCLEODUR C18 Pyramid column, 5 μM (Macherey Nagel, Düren, Germany); eluent A: 50 mM NH_4OAc pH 7.4, eluent B: acetonitrile, and flow: 0.6 mL/min. The gradient was set to 0−100% B from 0 to 2.5 min, 100% B from 2.5 to 3.0 min, 100−0% B from 3.0 to 3.2 min, and 0% B from 3.2−5.0.

Metabolic Stability in Liver Microsomes

For the evaluation of phase I metabolic stability, the compound (1 μM) was incubated with 0.5 mg/mL pooled mouse or human liver microsomes (Xenotech, Kansas City, USA), 2 mM NADPH, 10 mM $MgCl_2$ at 37 °C for 120 min on a microplate shaker (Eppendorf, Hamburg, Germany). The metabolic stability of testosterone, verapamil and ketoconazole was determined in parallel to confirm the enzymatic activity of mouse liver microsomes. For human liver microsomes, testosterone, diclofenac and propranolol were used. The incubation was stopped after defined time

points by precipitation of aliquots of enzymes with 2 volumes of cold internal standard solution (15 nM diphenhydramine in 10% methanol/acetonitrile). Samples were stored on ice until the end of the incubation and precipitated protein was removed by centrifugation (15 min, 4 °C, 4,000 g). The remaining test compound at the different time points was analyzed by HPLC-MS/MS (Vanquish Flex coupled to a TSQ Altis Plus, Thermo Fisher, Dreieich, Germany) and used to determine half-life ($t_{1/2}$).

Metabolic Stability in Liver S9 Fractions

For the evaluation of combined phase I and phase II metabolic stability, the compound (1 µM) was incubated with 1 mg/mL pooled mouse liver S9 fraction (Xenotech, Kansas City, USA), 2 mM NADPH, 1 mM UDPGA, 10 mM $MgCl_2$, 5 mM GSH and 0.1 mM PAPS at 37 °C for 240 min. The metabolic stability of testosterone, verapamil and ketoconazole were determined in parallel to confirm the enzymatic activity of mouse S9 fractions. The incubation was stopped after defined time points by precipitation of aliquots of S9 enzymes with 2 volumes of cold internal standard solution (15 nM diphenhydramine in 10% methanol/acetonitrile). Samples were stored on ice until the end of the incubation and precipitated protein was removed by centrifugation (15 min, 4 °C, 4,000 g). The remaining test compound at the different time points was analyzed by HPLC-MS/MS (Vanquish Flex coupled to a TSQ Altis Plus, Thermo Fisher, Dreieich, Germany) and used to determine half-life ($t_{1/2}$).

Stability in Plasma

To determine stability in plasma, the compound (1 µM) was incubated with pooled CD-1 mouse or human plasma (Neo Biotech, Nanterre, France). Samples were taken at defined time points by mixing aliquots with 4 volumes of ice-cold internal standard solution (12.5 nM diphenhydramine in 10% methanol/acetonitrile). Samples were stored on ice until the end of the incubation and precipitated protein was removed by centrifugation (15 min, 4 °C, 4,000 g, 2 centrifugation steps). The remaining test compound at the different time points was analyzed by HPLC-MS/MS (Vanquish Flex coupled to a TSQ Altis Plus, Thermo Fisher, Dreieich, Germany). The plasma stability of procain, propantheline and diltiazem were determined in parallel to confirm the enzymatic activity.

Plasma Protein Binding

Plasma protein binding was determined using the Rapid Equilibrium Dialysis (RED) system (Thermo Fisher Scientific, Waltham MA, USA). Compounds were diluted to 10 µM in 50% murine (CD-1) plasma (Neo Biotech, Nanterre, France) in PBS pH 7.4 and added to the respective chamber according to the manufacturer's protocol, followed by addition of PBS pH 7.4 to the opposite chamber. Diclofenac and propranolol were used as controls. Samples were taken immediately after addition to the plate as well as after 2, 4 and 5 h by mixing 10 µL with 80 µL ice-cold ice-cold internal standard solution (12.5 nM diphenhydramine in 10% methanol/acetonitrile), followed by addition of 10 µL plasma to samples taken from PBS and vice

versa. Samples were stored on ice until the end of the incubation and precipitated protein was removed by centrifugation (15 min, 4 °C, 4,000 g, 2 centrifugation steps). The amount of the remaining test compound at the different time points was analyzed by HPLC-MS/MS (Vanquish Flex coupled to a TSQ Altis Plus, Thermo Fisher, Dreieich, Germany). The amount of compound bound to protein was calculated using the equation PPB [%] = 100 – 100 x (amount in buffer chamber/amount in plasma chamber).

Table S6. *In vitro* ADMET parameters of compounds **4**, **67**, **68**. $LogD_{7.4}$ was determined using a chromatographic method. $t_{1/2}$: half-life, Cl_{int}: intrinsic clearance, n.d.: not determined.

**Parameters**	**Compound**		
	**4**	**67**	**68**
clogP[a]	5.25	5.73	5.95
Chrom. $logD_{7.4}$	n.d.	-0.4	4.3
Liver Microsomes (mouse) $t_{1/2}$ [min] / Cl_{int} [μL/min/mg]	15.5±1.6/ 90±9.4	59±24/ 27±14	67±10/ 21 ± 3
PPB % (mouse)	99.85 ± 0.06	99.81 ± 0.01	99.97 ± 0.02
Liver S9 (mouse) $t_{1/2}$ [min] / Cl_{int} [μL/min/mg]	23.6±2.4/ 29.6±3.0	94±24/ 7.7± 2.1	73±17/ 9.8±2.3
Liver Microsomes (human) $t_{1/2}$ [min] / Cl_{int} [μL/min/mg]	7.0±1.8/ 207±52	7.6±4.3/ 264±183	24.3±3.5/ 57.6 ±8.5
Mouse Plasma $t_{1/2}$ [min]	>240	>240	>240
Human Plasma $t_{1/2}$ [min]	>240	>240	>240

[a]=calculated with Stardrop 7.0.1.29911.

7.0 Mass spectrometric conditions of the pharmacokinetic studies

Table S7: MS/MS transitions for bioanalytics of caffeine (internal standard), **4**, **67** and **68**.

ID	Q1 Mass [Da]	Q3 Mass [Da]	time [msec]	DP [volts]	CE [volts]	CXP [volts]
**Caffeine**	195.024	138.000 (Q)	50.0	60.0	25.0	14.0
		110.000	50.0	60.0	31.0	18.0
**4**	372.848	151.900 (Q)	50.0	-14.0	-22.0	-23.0
		107.900	50.0	-24.0	-52.0	-49.
**67**	355.047	151.900 (Q)	20.0	-30.0	-30.0	-21.0
		107.900	20.0	-30.0	-60.0	-55.0
		310.700	20.0	-30.0	-24.0	-15.0
**68**	453.680	176.900	20.0	-70.0	-28.0	-21.0
		380.100	20.0	-70.0	-18.0	-19.0
		132.900	20.0	-70.0	-46.0	-15.0

8.0 Spectra of representative compounds

All final compounds have a purity of > 95%.

Compound 27

HR-MS, HPLC-MS, ^{1}H-NMR, ^{13}C-NMR

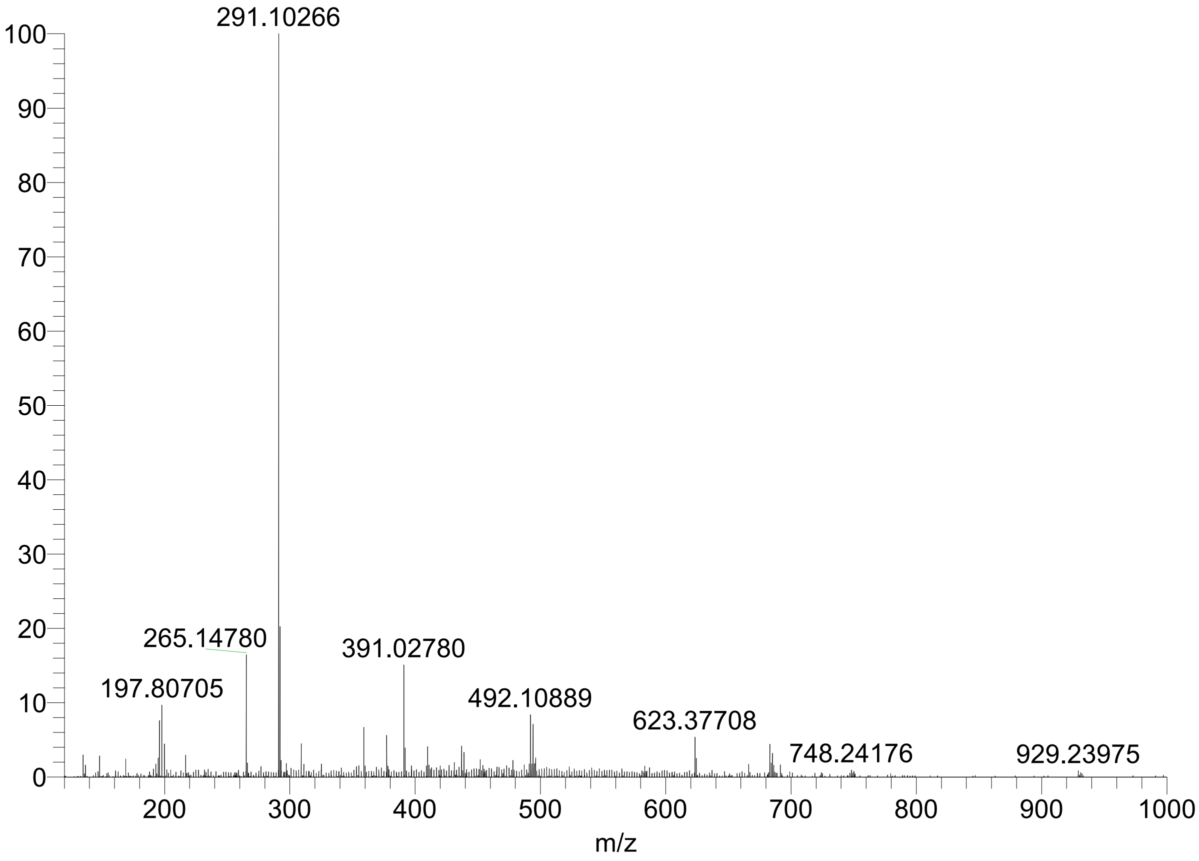
291.10266
265.14780
197.80705
391.02780
492.10889
623.37708
748.24176
929.23975
m/z

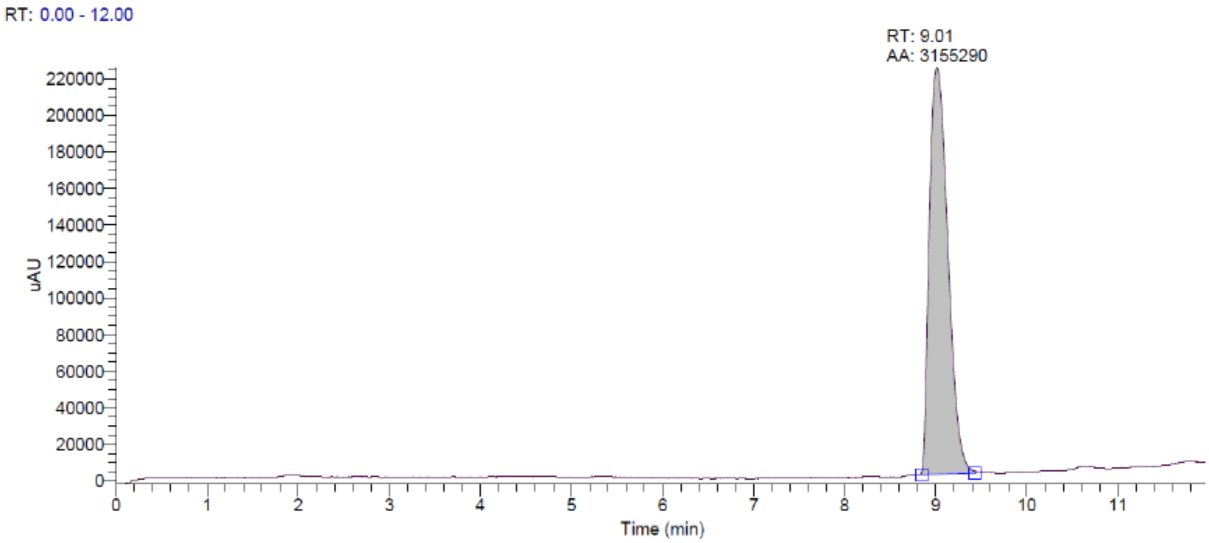
RT: 0.00 - 12.00
RT: 9.01
AA: 3155290
uAU
Time (min)

Purity = 100%

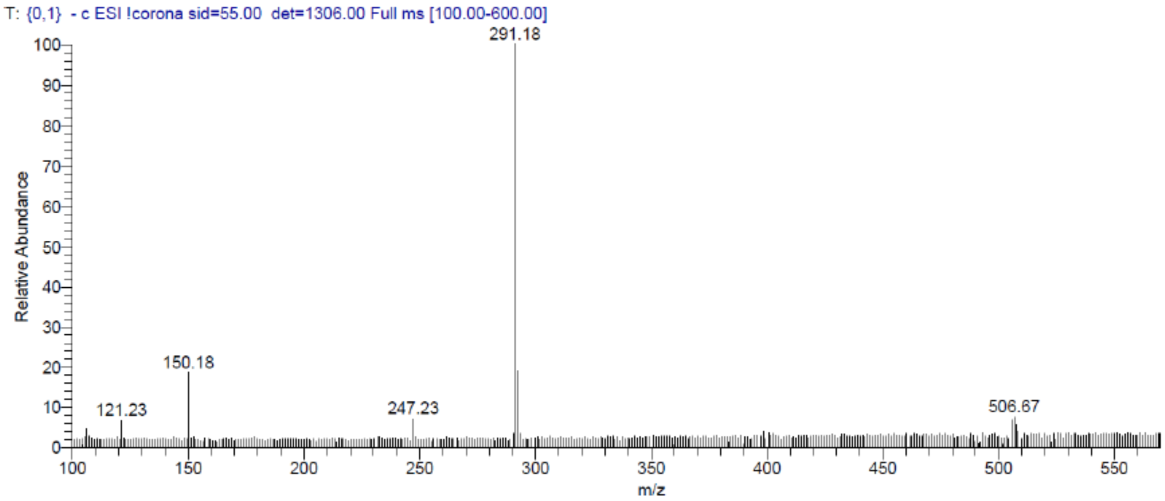

T: {0,1} - c ESI !corona sid=55.00 det=1306.00 Full ms [100.00-600.00]
291.18
150.18
121.23
247.23
506.67
Relative Abundance
m/z

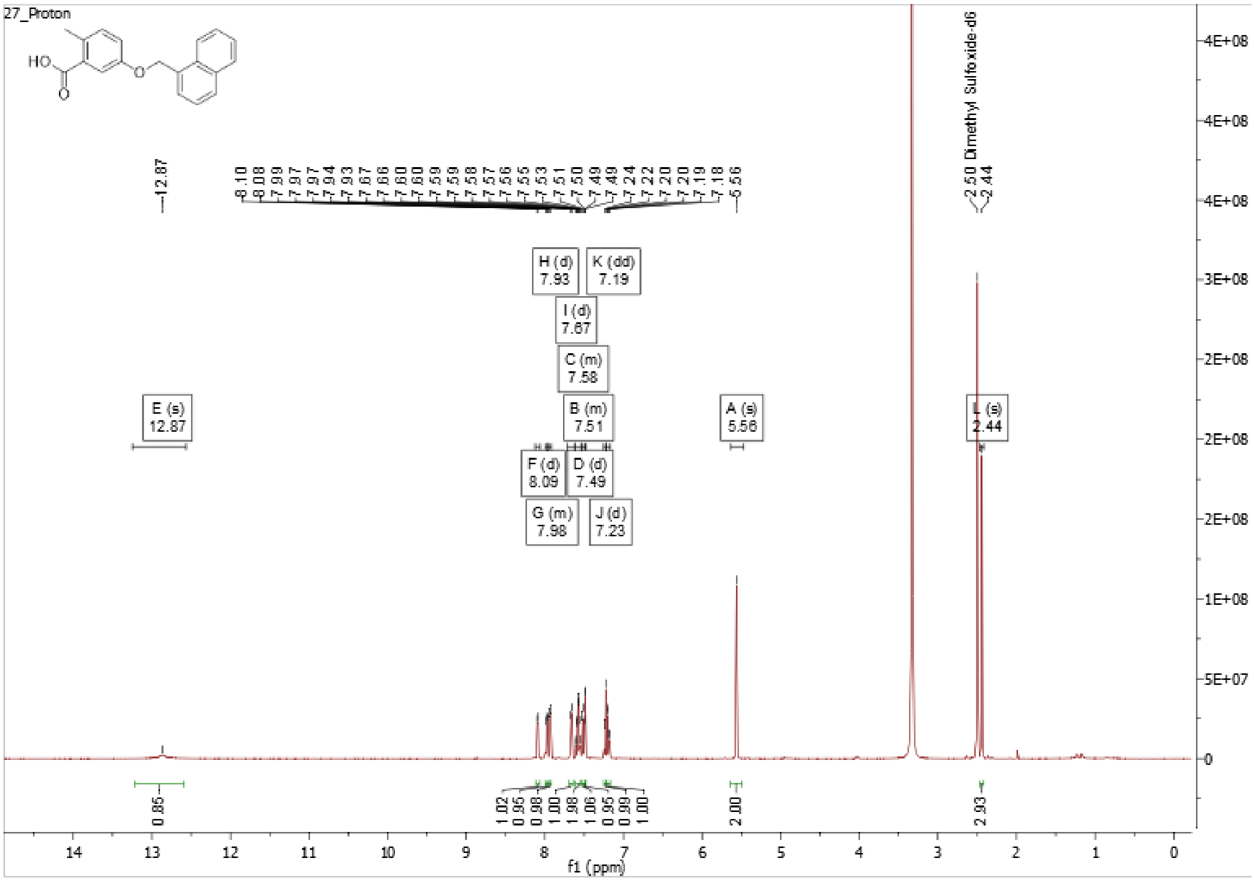

27_Proton
HO
O
O
12.87
8.10 8.08 7.99 7.97 7.94 7.93 7.67 7.66 7.60 7.60 7.59 7.59 7.58 7.57 7.56 7.55 7.53 7.51 7.50 7.49 7.49 7.24 7.22 7.20 7.20 7.19 7.18
5.56
2.50 Dimethyl Sulfoxide-d6
2.44
H (d) 7.93
K (dd) 7.19
I (d) 7.67
C (m) 7.58
E (s) 12.87
B (m) 7.51
A (s) 5.56
L (s) 2.44
F (d) 8.09
D (d) 7.49
G (m) 7.98
J (d) 7.23
0.85
1.02 0.95 0.98 1.00 1.98 1.06 0.95 0.99 1.00
2.00
2.93
f1 (ppm)

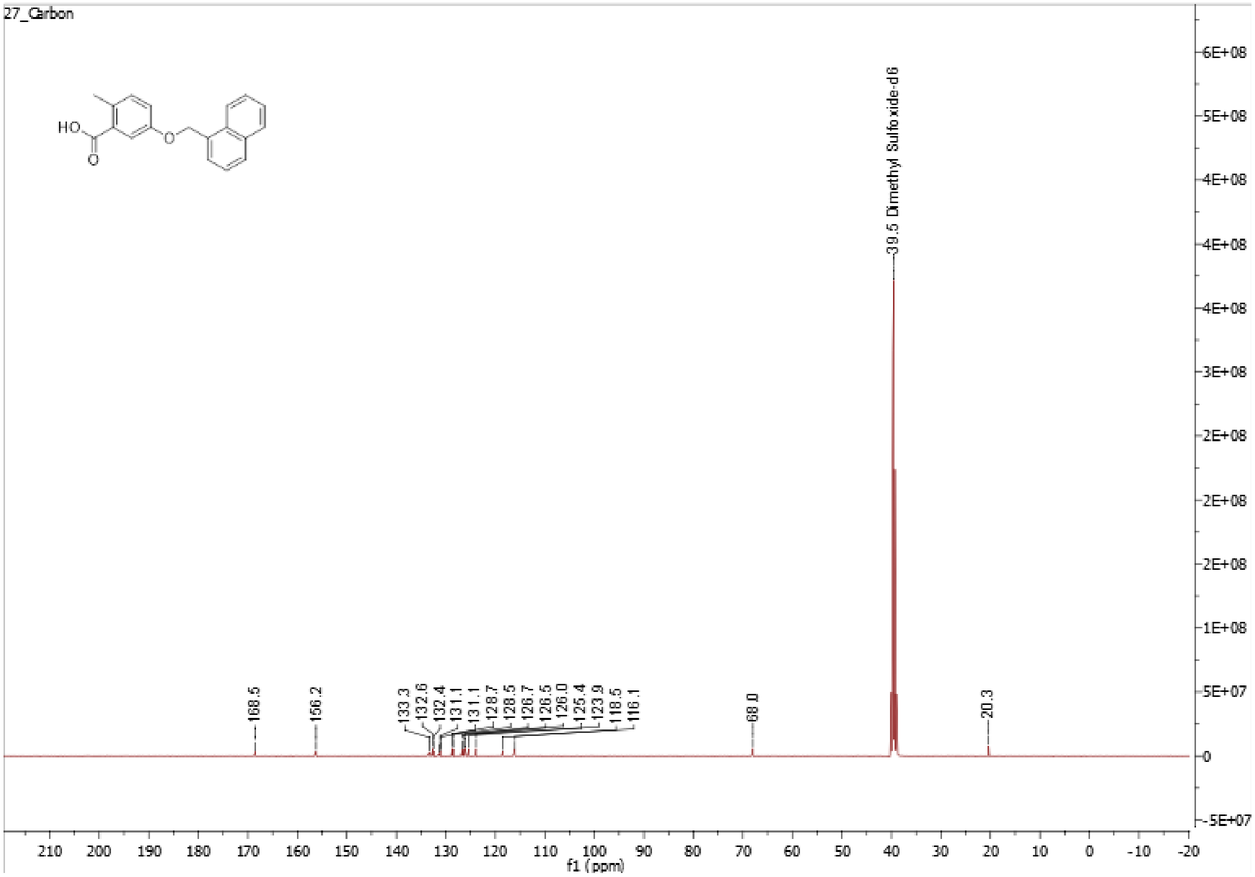

27_Carbon
HO
O
O
168.5
156.2
133.3
132.6
132.4
131.1
131.1
128.7
128.5
126.7
126.5
126.0
125.4
123.9
118.5
116.1
68.0
39.5 Dimethyl Sulfoxide-d6
20.3
6E+08
5E+08
4E+08
4E+08
4E+08
3E+08
2E+08
2E+08
2E+08
1E+08
5E+07
0
-5E+07
210
200
190
180
170
160
150
140
130
120
110
100
90
80
70
60
50
40
30
20
10
0
-10
-20
f1 (ppm)

Compound 28

HR-MS, HPLC-MS, ^{1}H-NMR, ^{13}C-NMR

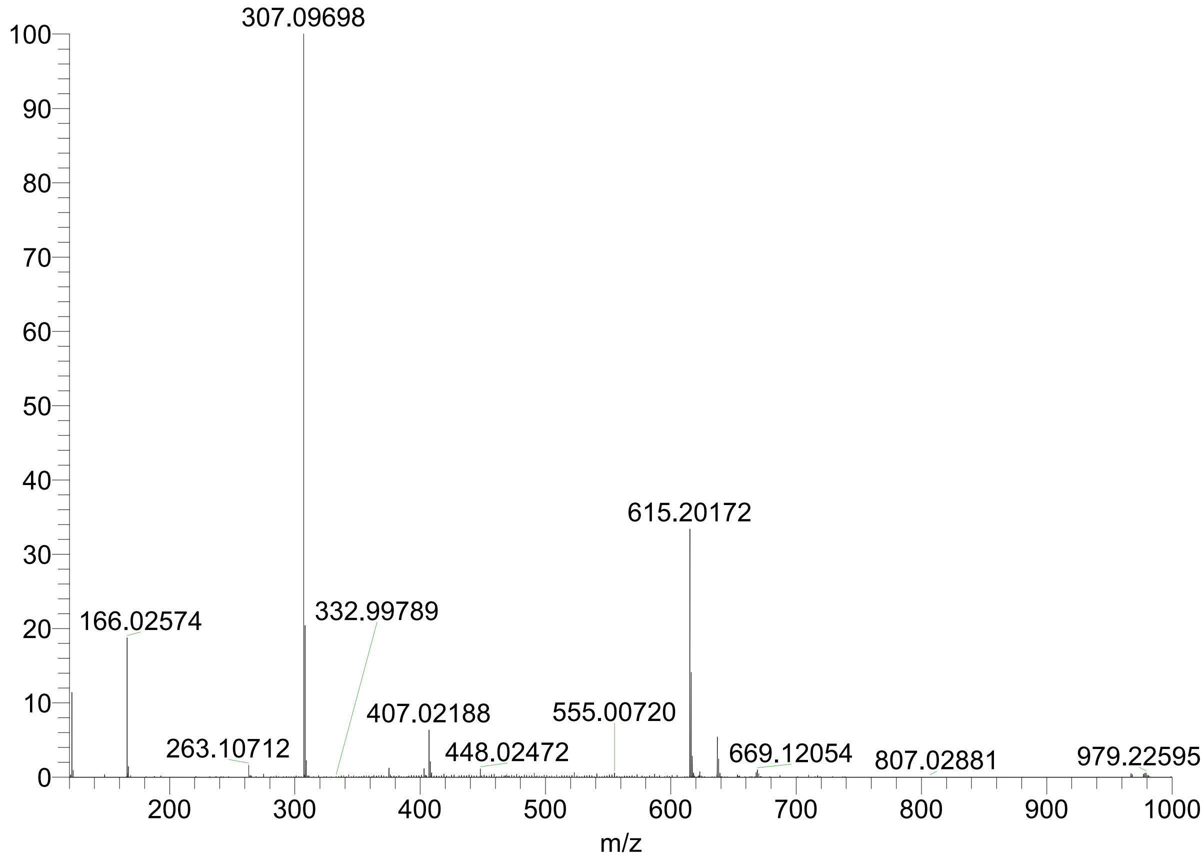

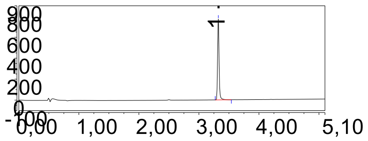

Purity = 100%

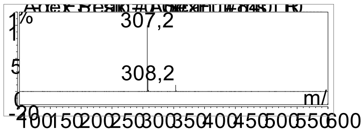
307,2
308,2
m/
-20
100 150 200 250 300 350 400 450 500 550 600

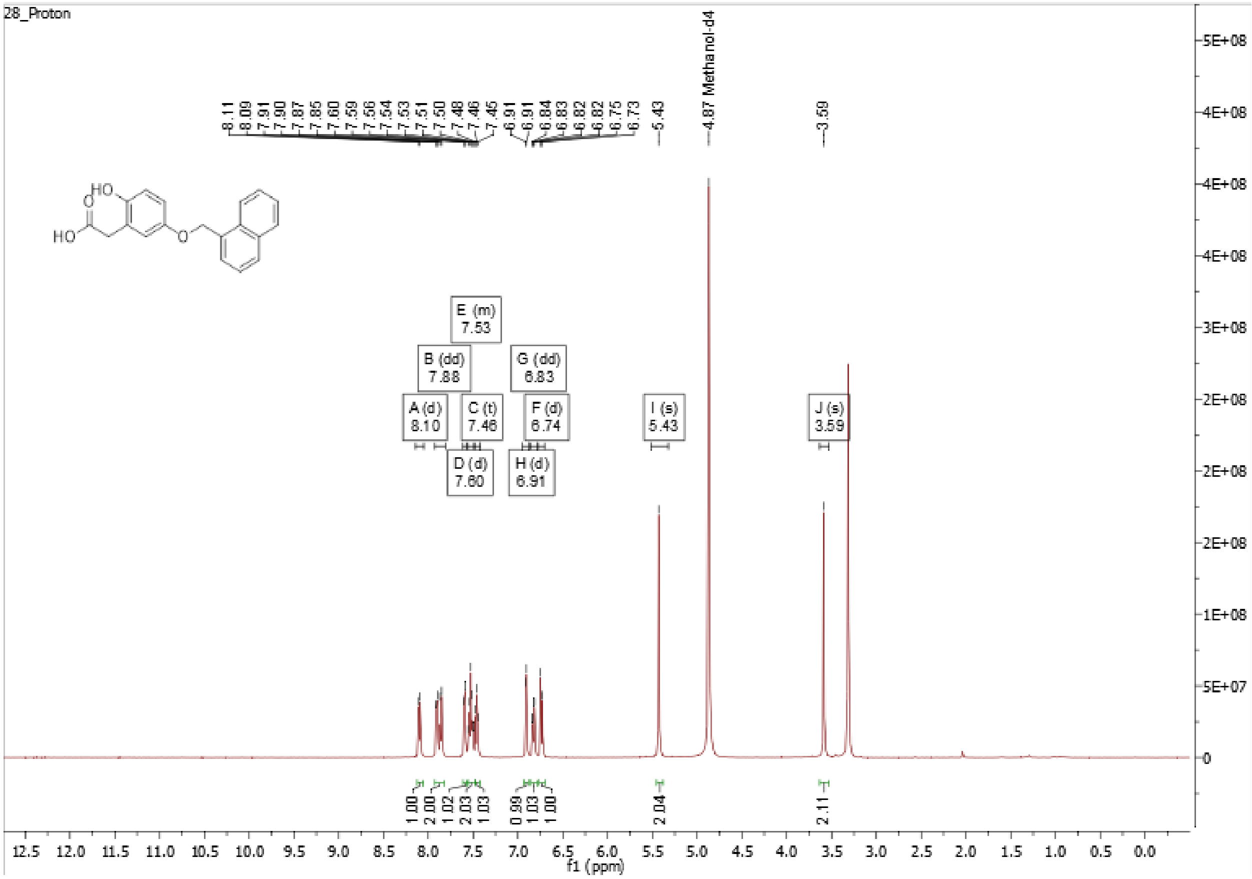
28_Proton
4.87 Methanol-d4
5.43
3.59
A (d) 8.10
B (dd) 7.88
C (t) 7.46
D (d) 7.60
E (m) 7.53
F (d) 6.74
G (dd) 6.83
H (d) 6.91
I (s) 5.43
J (s) 3.59
f1 (ppm)

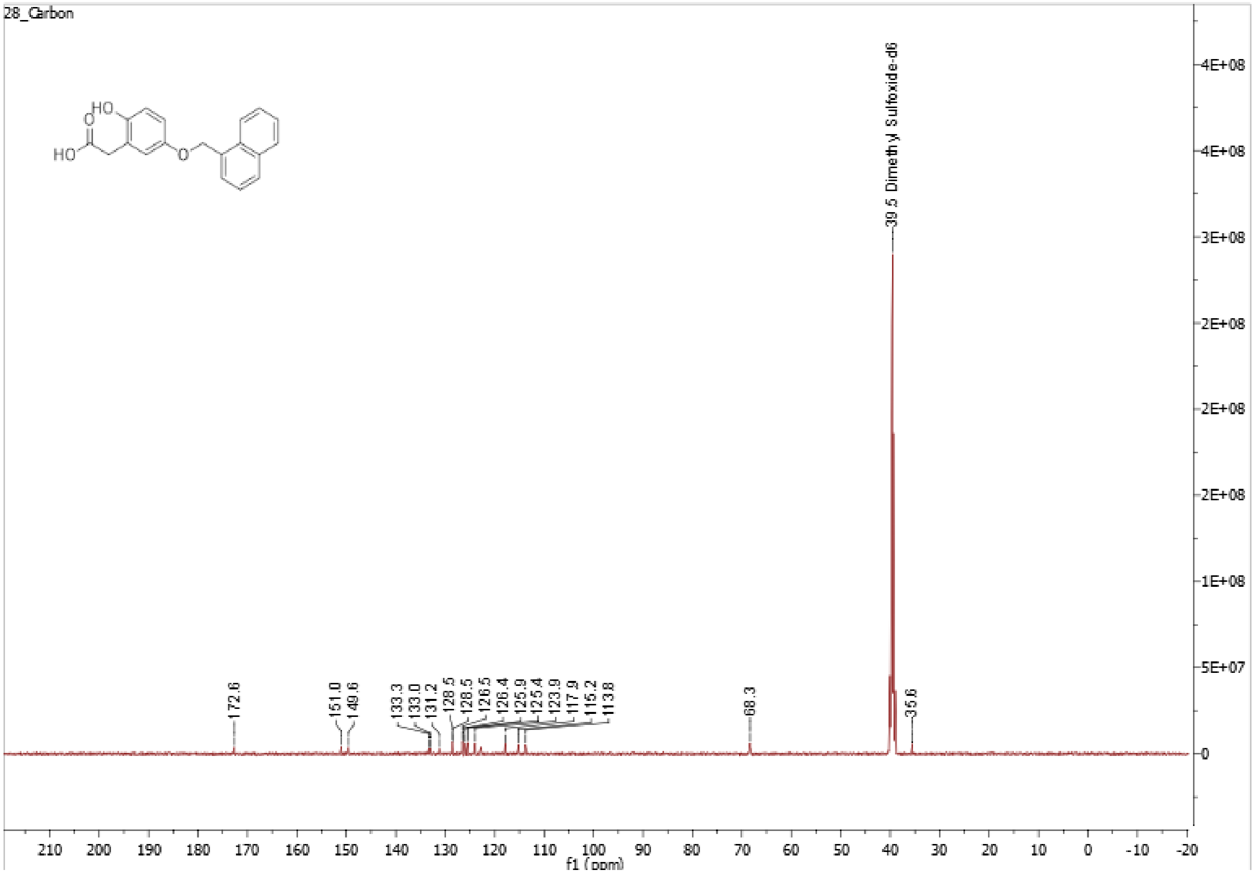

28_Carbon
HO
O
HO
O
39.5 Dimethyl Sulfoxide-d6
172.6
151.0
149.6
133.3
133.0
131.2
128.5
128.5
126.5
126.4
125.9
125.4
123.9
117.9
115.2
113.8
68.3
35.6
4E+08
4E+08
3E+08
2E+08
2E+08
2E+08
1E+08
5E+07
0
210 200 190 180 170 160 150 140 130 120 110 100 90 80 70 60 50 40 30 20 10 0 -10 -20
f1 (ppm)

Compound 29

HPLC-MS, ^{1}H-NMR, ^{13}C-NMR

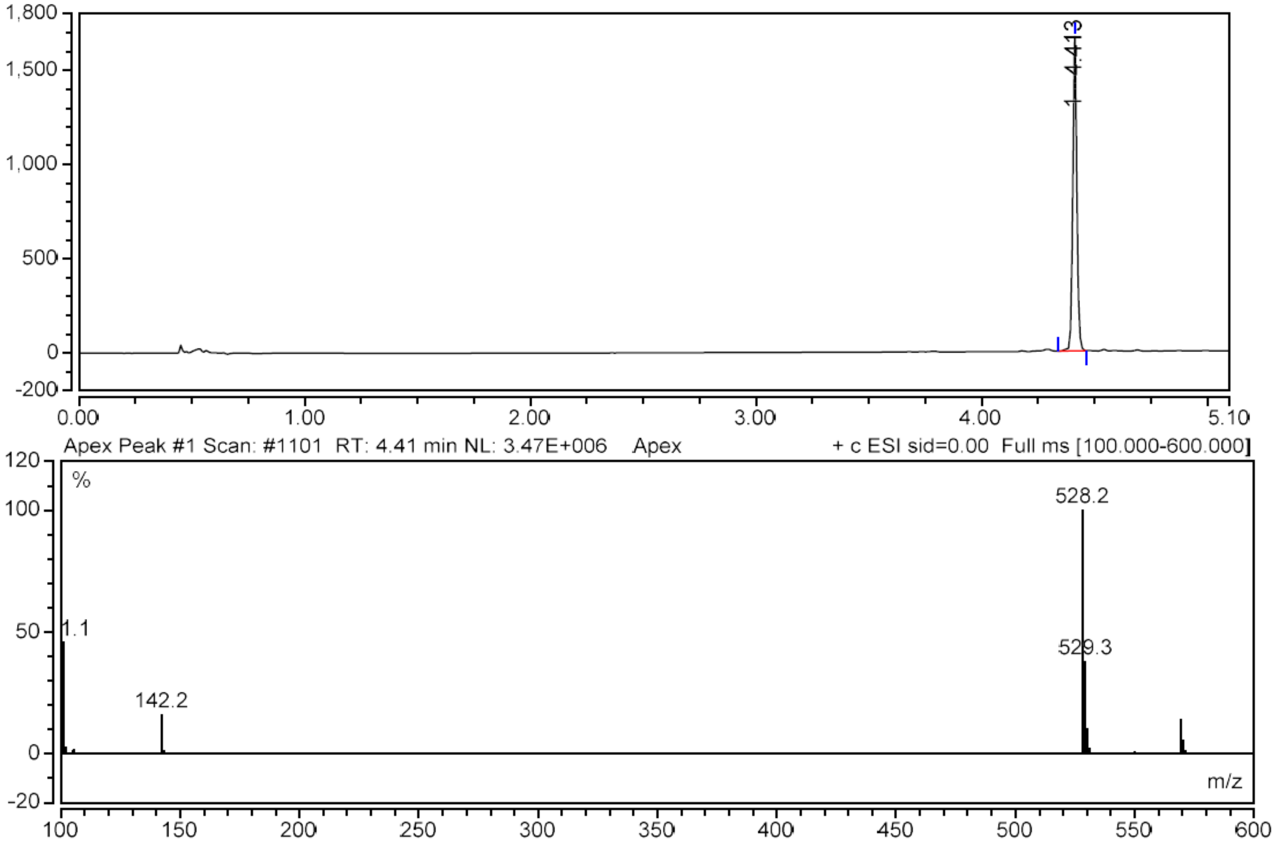

Purity = 100%

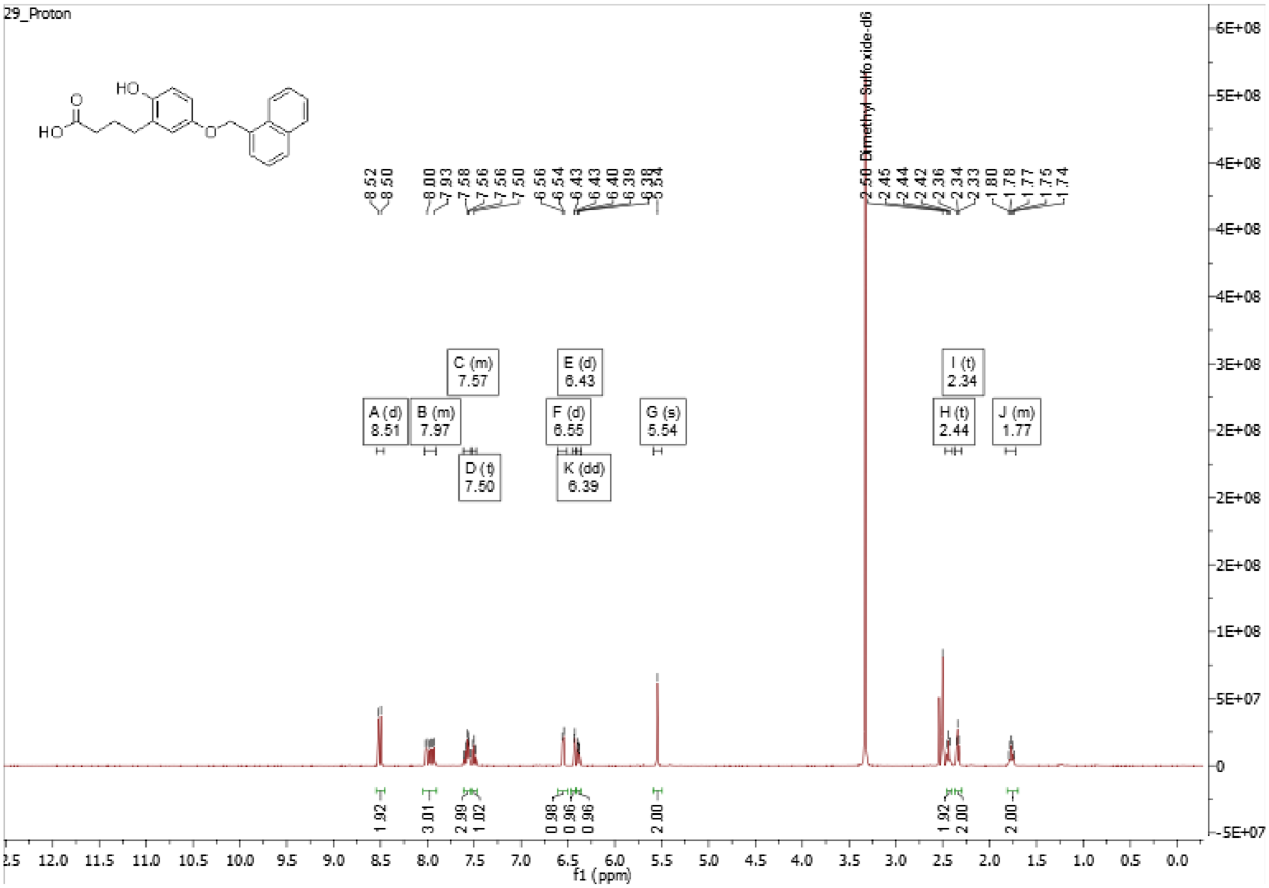
29_Proton
2.50 Dimethyl Sulfoxide-d6
A (d) 8.51
B (m) 7.97
C (m) 7.57
D (t) 7.50
E (d) 6.43
F (d) 6.55
G (s) 5.54
H (t) 2.44
I (t) 2.34
J (m) 1.77
K (dd) 6.39
f1 (ppm)

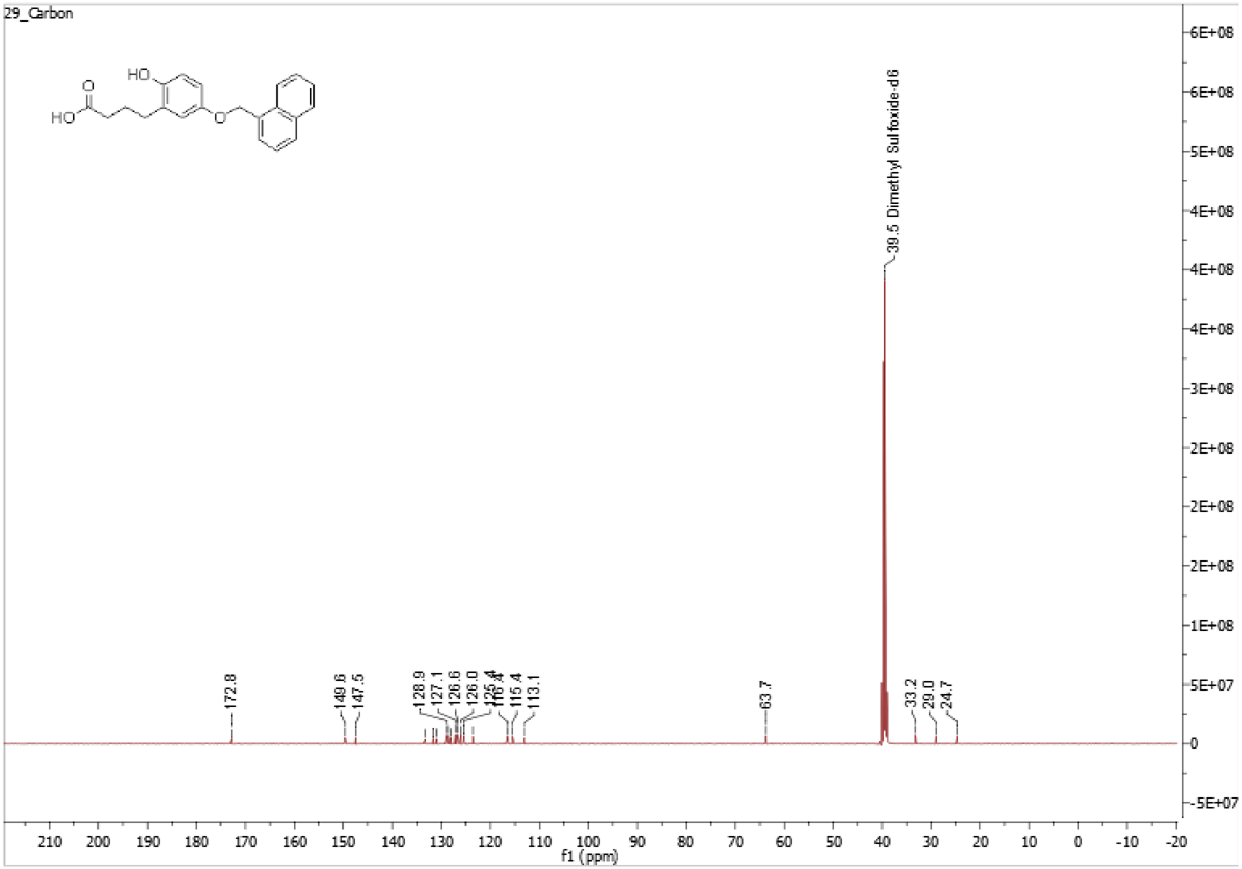
29_Carbon
HO
HO
O
O
172.8
149.6
147.5
128.9
127.1
126.6
126.0
115.4
113.1
63.7
39.5 Dimethyl Sulfoxide-d6
33.2
29.0
24.7
f1 (ppm)

Compound 31

HR-MS, HPLC-MS, ^{1}H-NMR, ^{13}C-NMR

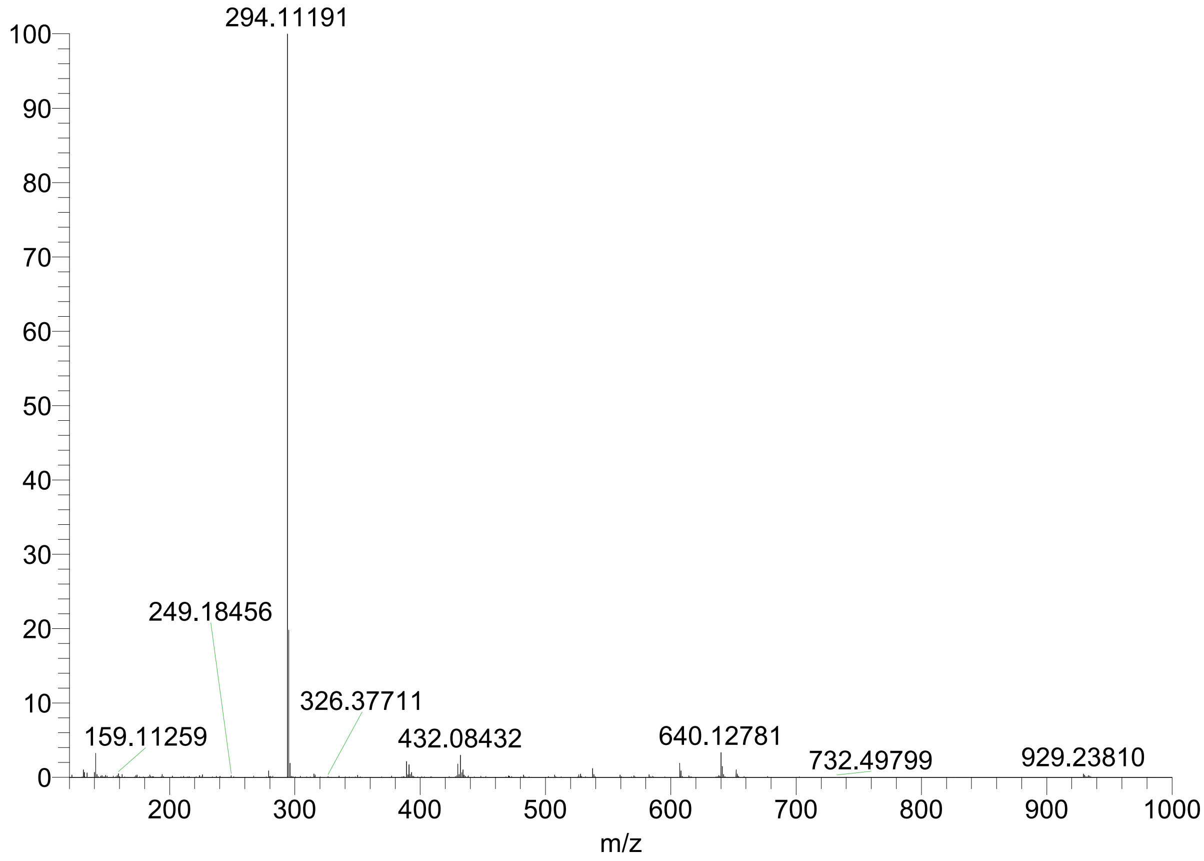
294.11191
249.18456
326.37711
159.11259
432.08432
640.12781
732.49799
929.23810
100
90
80
70
60
50
40
30
20
10
0
200
300
400
500
600
700
800
900
1000
m/z

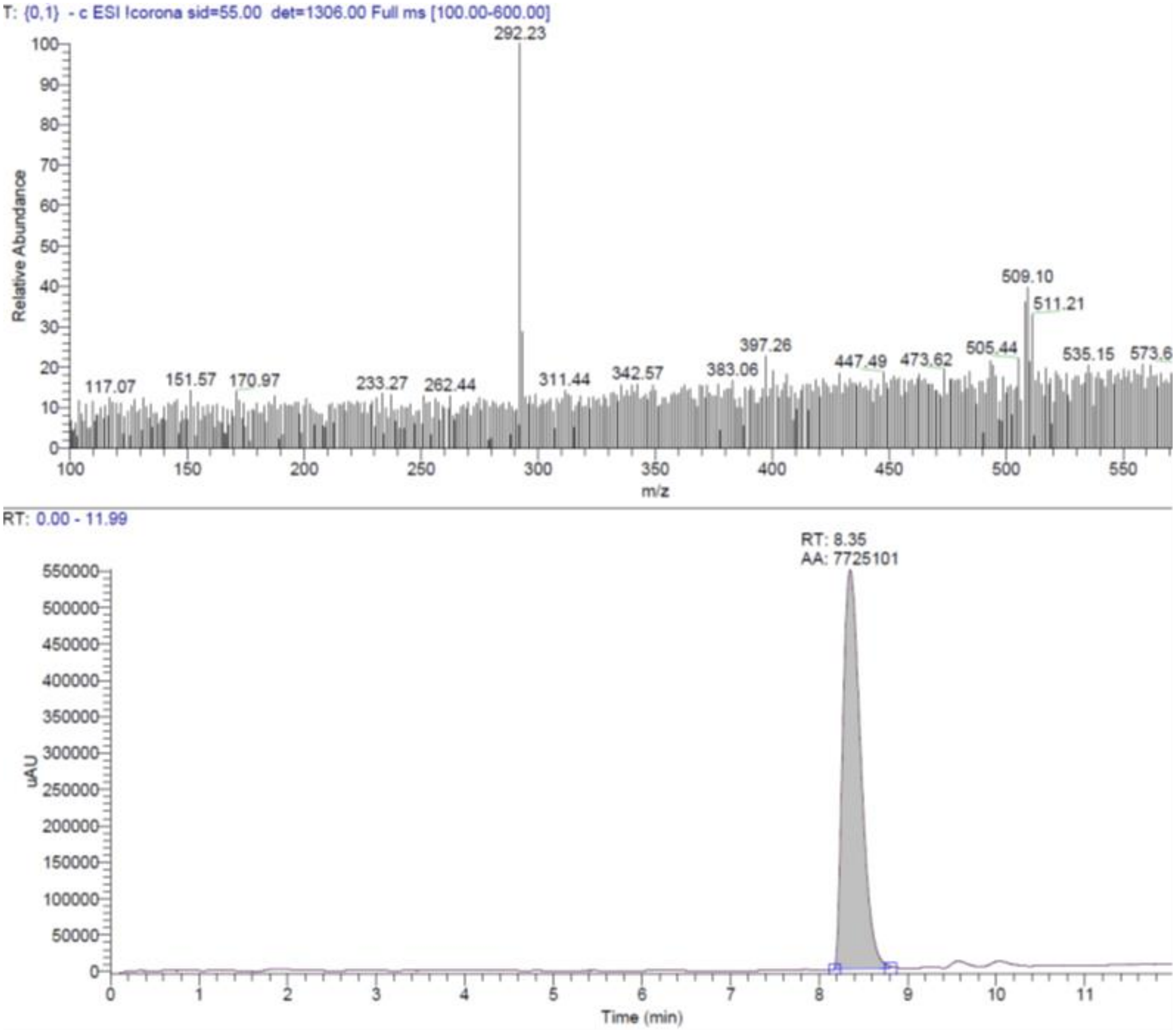
T: {0,1} - c ESI Icorona sid=55.00 det=1306.00 Full ms [100.00-600.00]
292.23
509.10
511.21
117.07
151.57
170.97
233.27
262.44
311.44
342.57
383.06
397.26
447.49
473.62
505.44
535.15
573.6
Relative Abundance
m/z
RT: 0.00 - 11.99
RT: 8.35
AA: 7725101
uAU
Time (min)

Purity = 98%

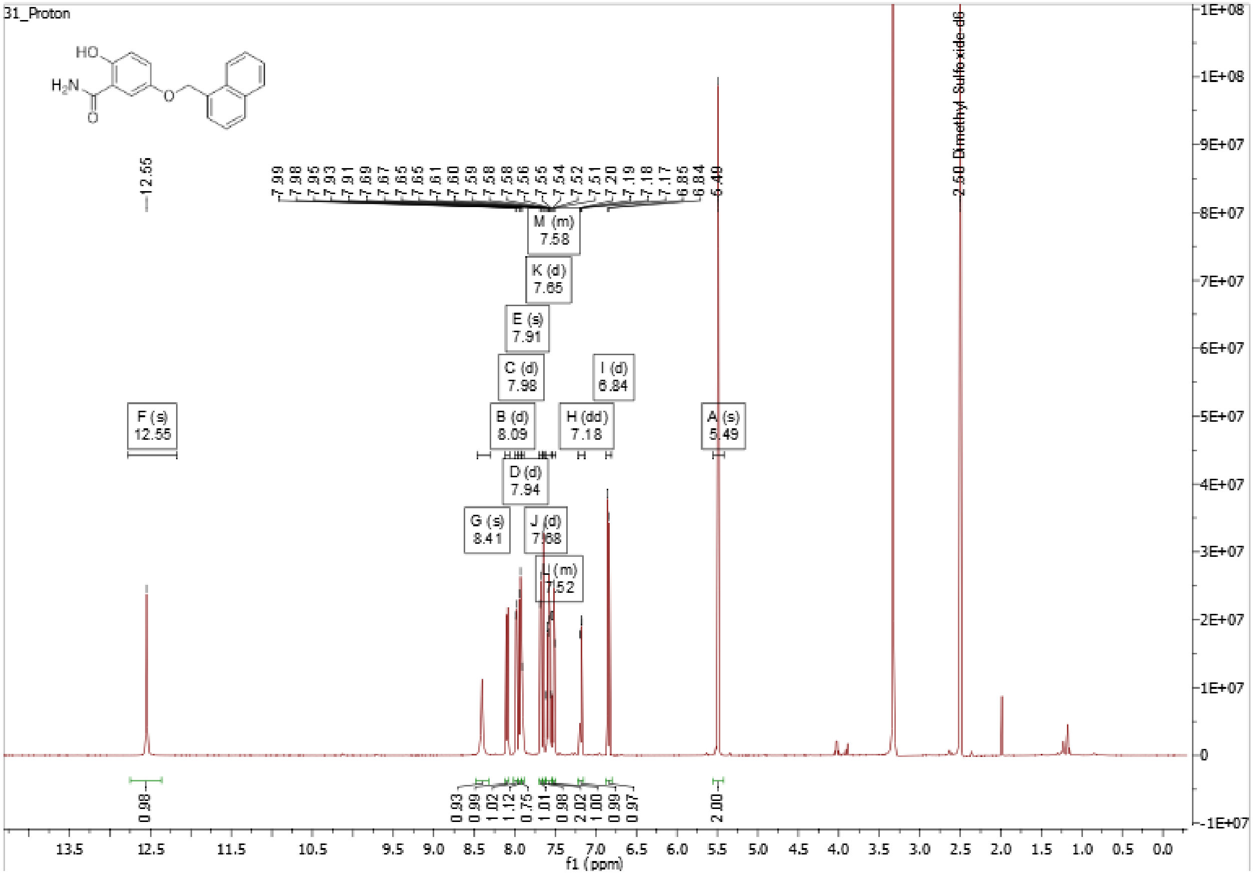
31_Proton
12.55
F (s)
12.55
2.50 Dimethyl Sulfoxide-d6
5.49
A (s)
5.49
f1 (ppm)

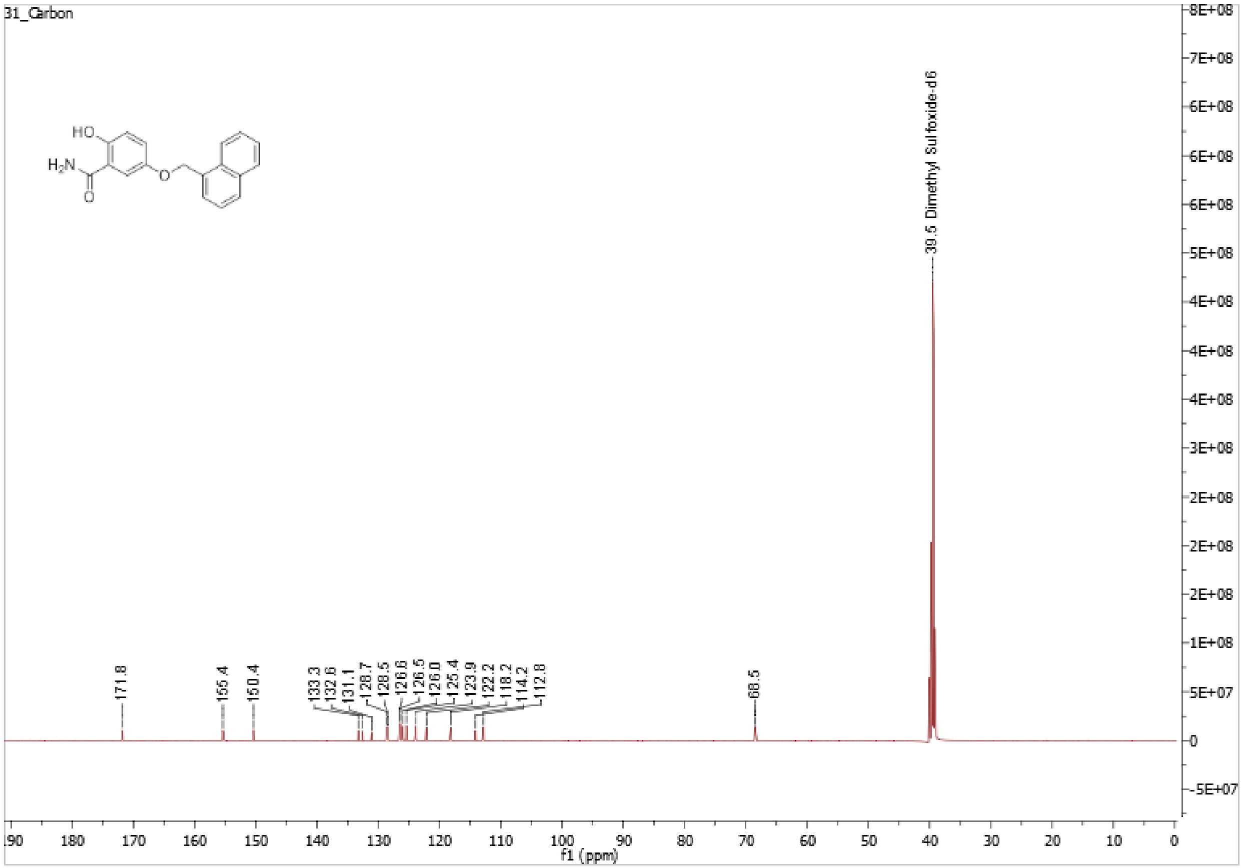

Compound 33

HR-MS, HPLC-MS, ^{1}H-NMR, ^{13}C-NMR

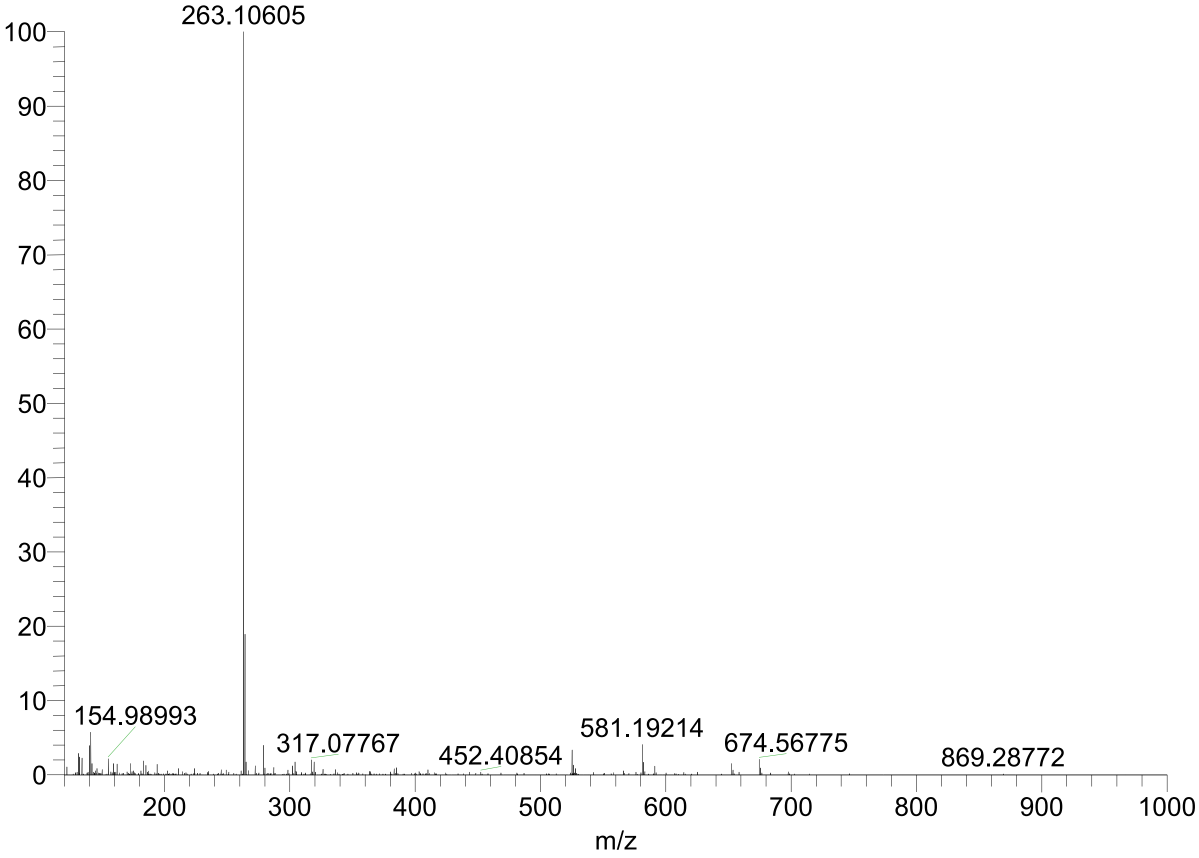
263.10605
154.98993
317.07767
452.40854
581.19214
674.56775
869.28772
m/z

(A)

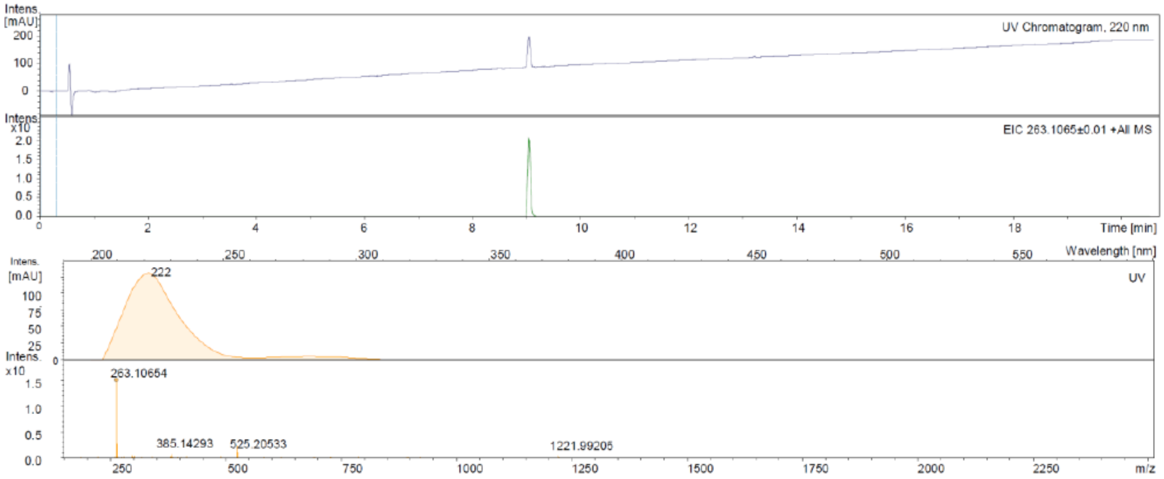
Intens. [mAU]
UV Chromatogram, 220 nm
Intens. x10
EIC 263.1065±0.01 +All MS
Time [min]
Wavelength [nm]
222
UV
263.10654
385.14293
525.20533
1221.99205
m/z

(B)

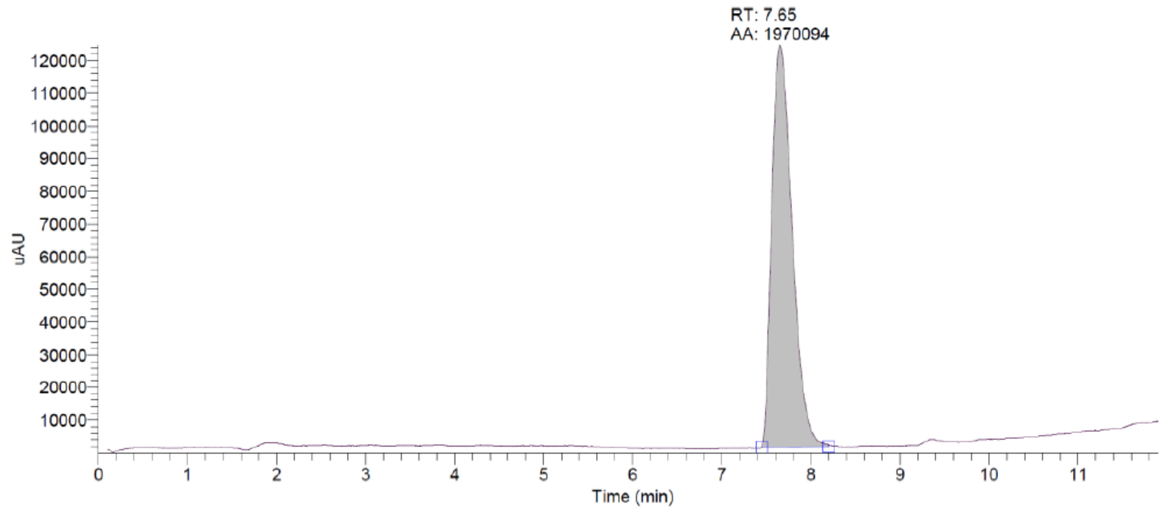
RT: 7.65
AA: 1970094
uAU
Time (min)

Purity = 100%

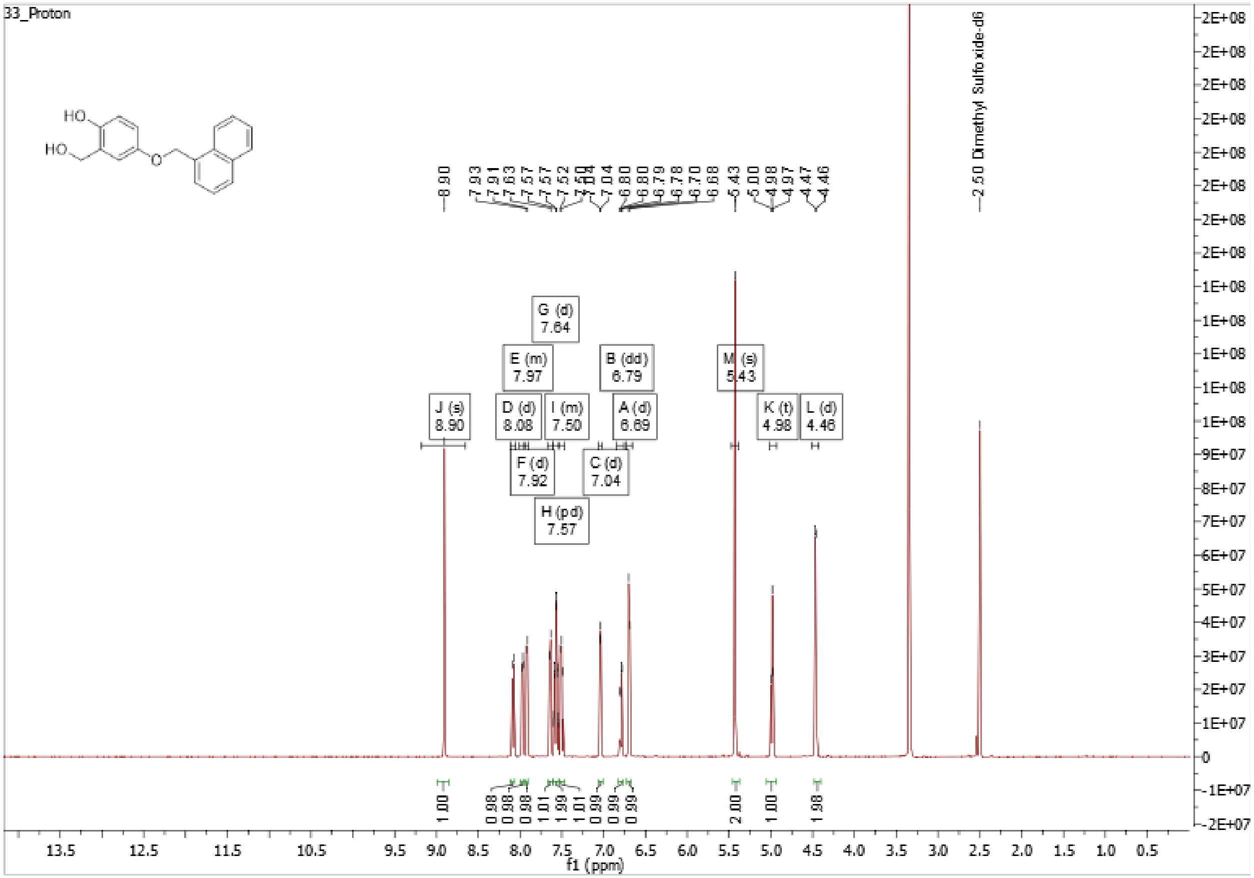
33_Proton
HO
HO
O
2.50 Dimethyl Sulfoxide-d6
J (s) 8.90
D (d) 8.08
E (m) 7.97
F (d) 7.92
G (d) 7.64
H (pd) 7.57
I (m) 7.50
C (d) 7.04
B (dd) 6.79
A (d) 6.69
M (s) 5.43
K (t) 4.98
L (d) 4.46
f1 (ppm)

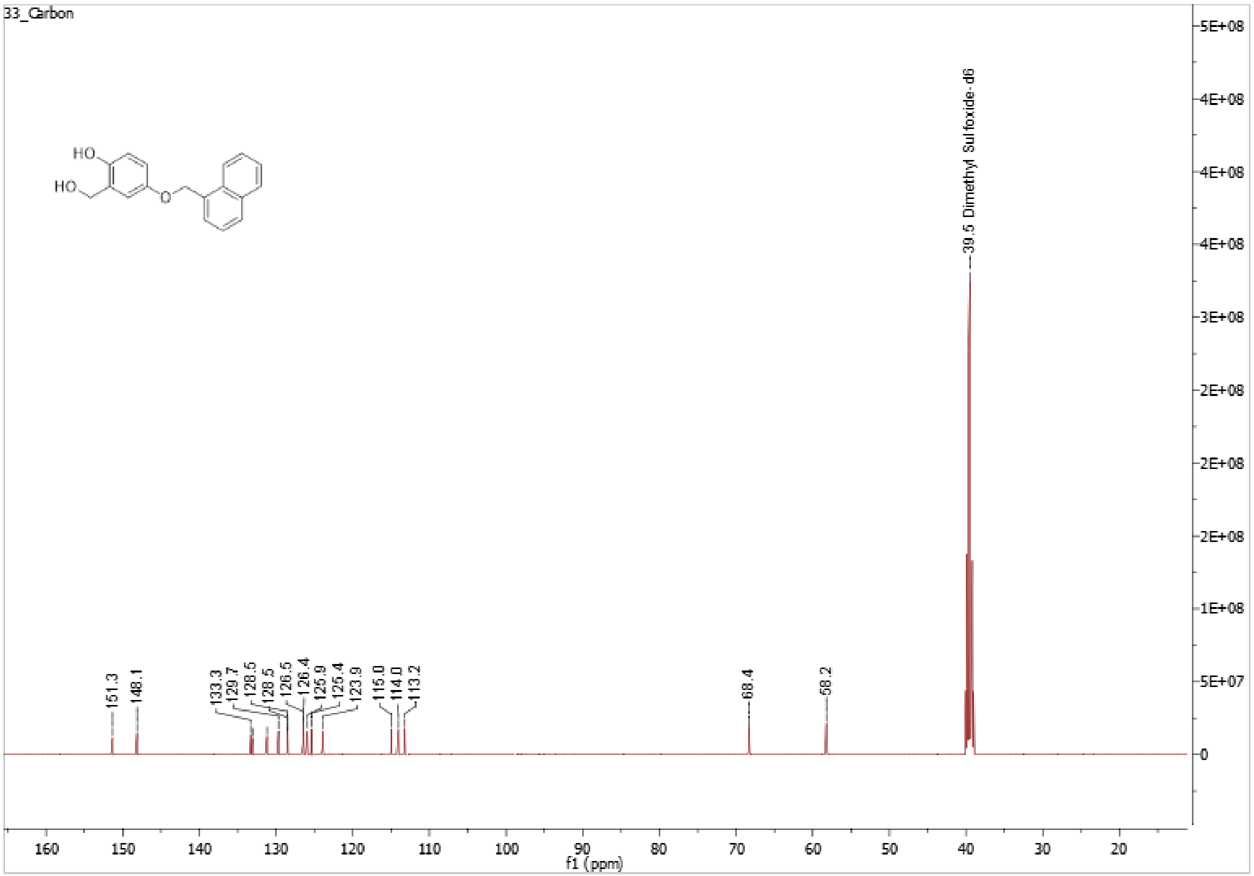

Compound 34

HR-MS, HPLC-MS, ^{1}H-NMR, ^{13}C-NMR

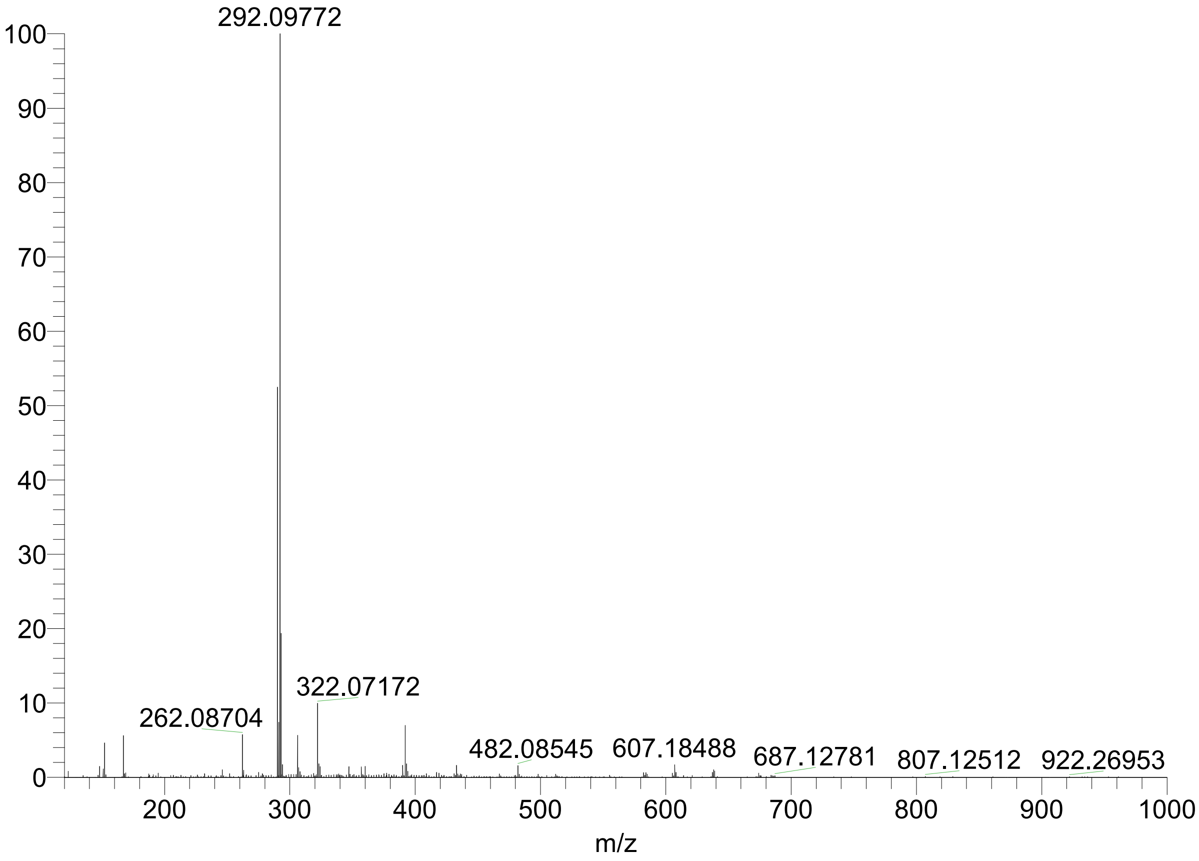

292.09772
262.08704
322.07172
482.08545
607.18488
687.12781
807.12512
922.26953
100
90
80
70
60
50
40
30
20
10
0
200
300
400
500
600
700
800
900
1000
m/z

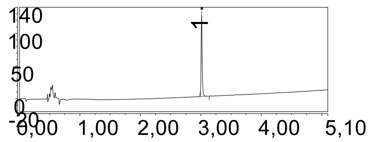
140
100
50
0
-20
0,00
1,00
2,00
3,00
4,00
5,10

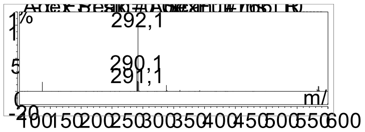
292,1
290,1
291,1
100
150
200
250
300
350
400
450
500
550
600

Purity = 100%

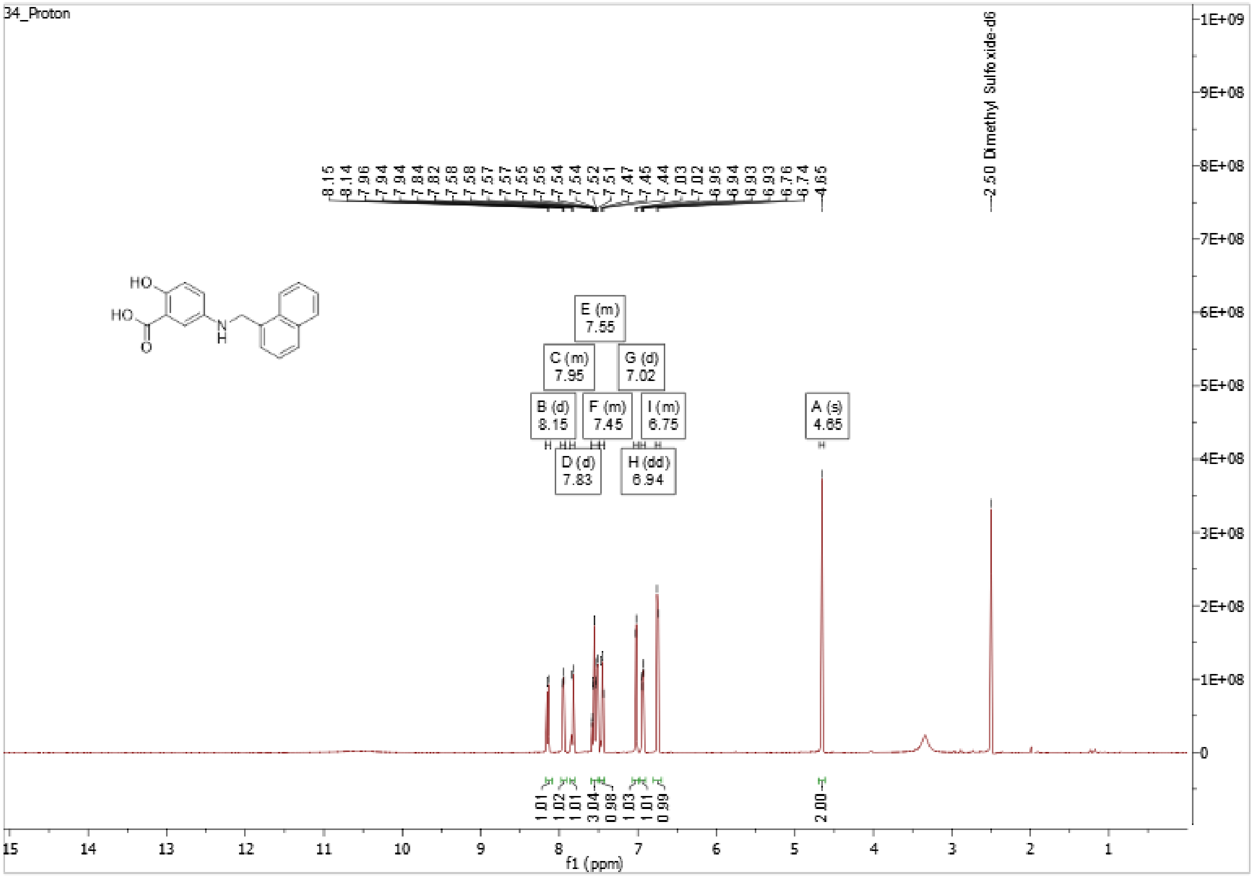
34_Proton
8.15
8.14
7.96
7.94
7.94
7.84
7.82
7.58
7.58
7.57
7.57
7.55
7.55
7.54
7.54
7.52
7.51
7.47
7.45
7.44
7.03
7.02
6.95
6.94
6.93
6.93
6.76
6.74
4.65
2.50 Dimethyl Sulfoxide-d6
HO
HO
O
N
H
E (m)
7.55
C (m)
7.95
G (d)
7.02
B (d)
8.15
F (m)
7.45
I (m)
6.75
A (s)
4.65
D (d)
7.83
H (dd)
6.94
1.01
1.02
1.01
3.04
0.98
1.03
1.01
0.99
2.00
f1 (ppm)

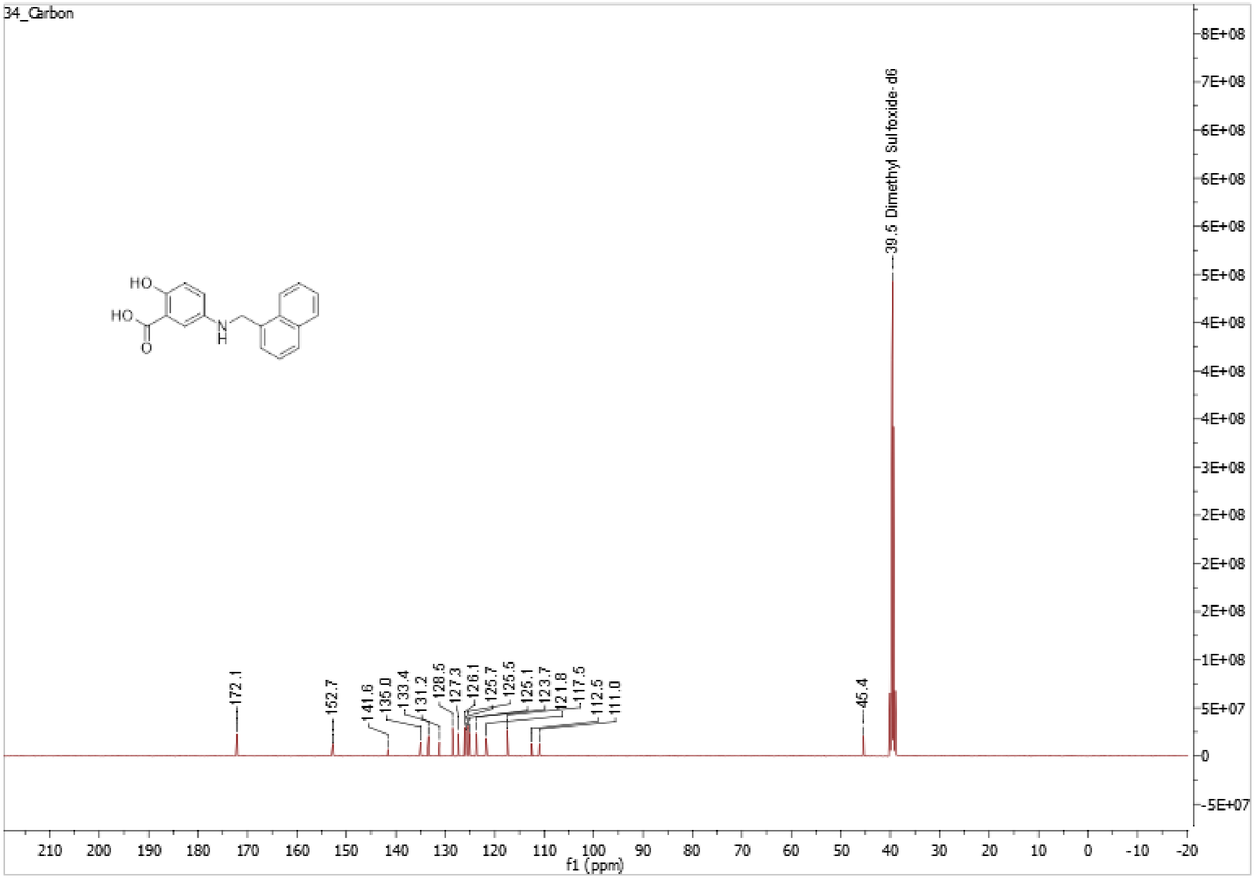
34_Carbon
HO
HO
O
N
H
172.1
152.7
141.6
135.0
133.4
131.2
128.5
127.3
126.1
125.7
125.5
125.1
123.7
121.8
117.5
112.5
111.0
45.4
39.5 Dimethyl Sulfoxide-d6
f1 (ppm)

Compound 35

HR-MS, HPLC-MS, ^{1}H-NMR, ^{13}C-NMR

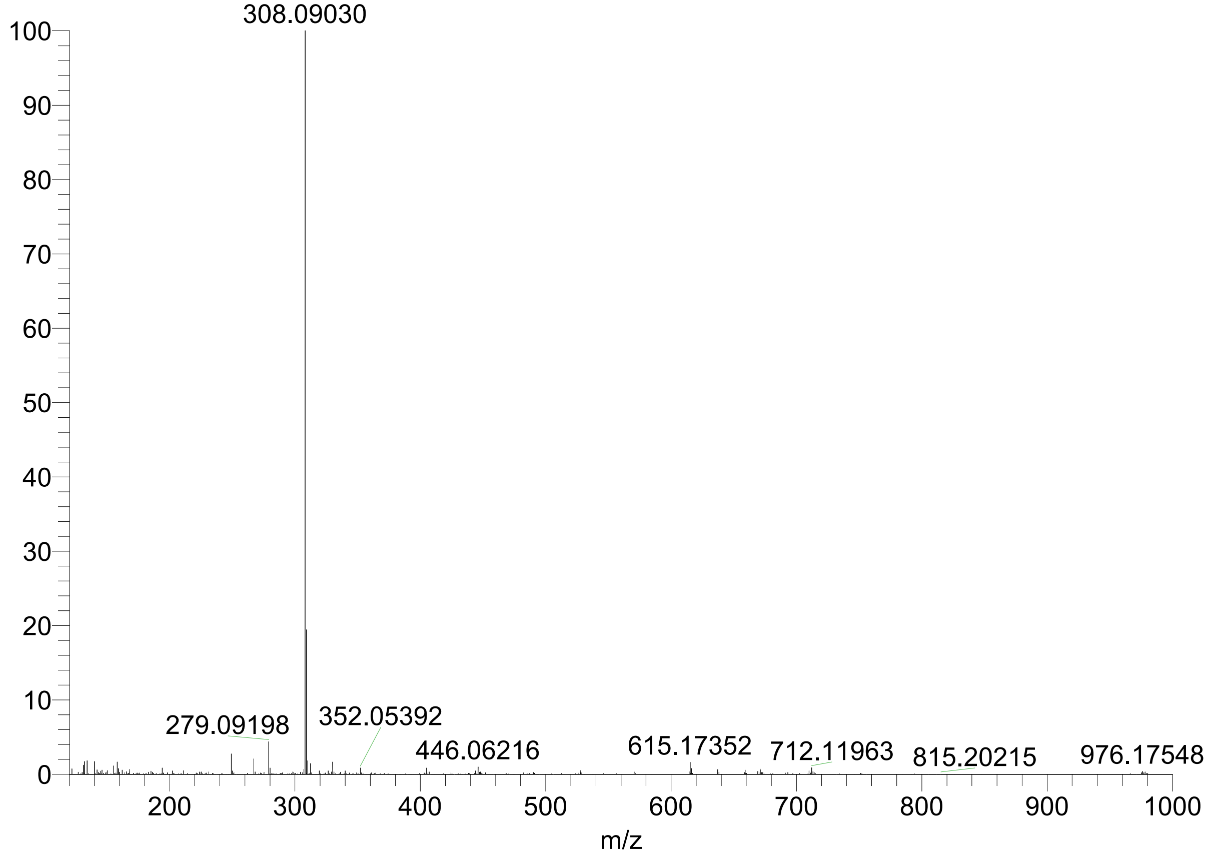

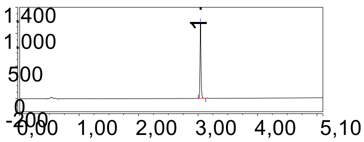

1,400
1,000
500
0
-200
0,00
1,00
2,00
3,00
4,00
5,10

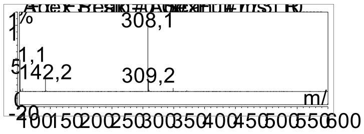

308,1
142,2
309,2
m/
100
150
200
250
300
350
400
450
500
550
600

Purity = 100%

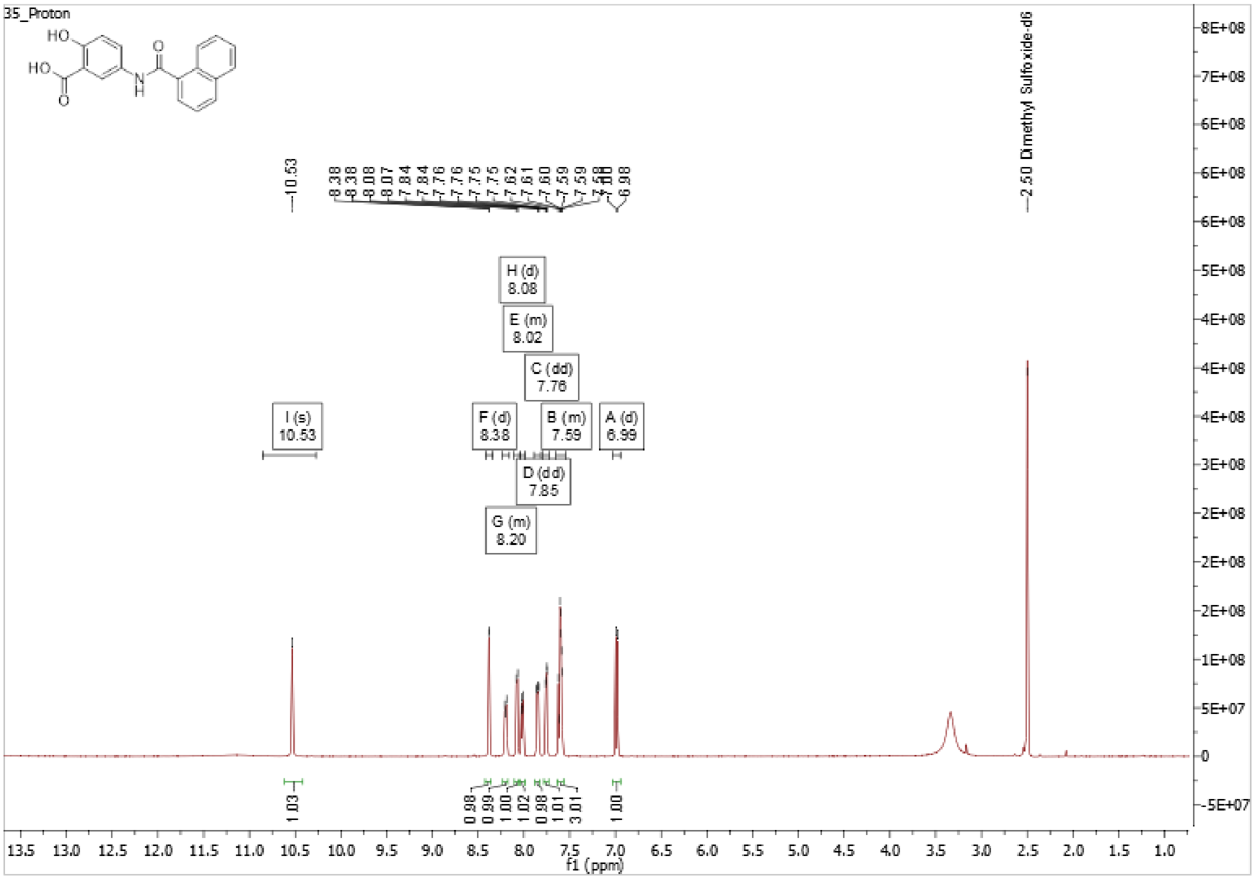

35_Proton
HO
HO
O
N
H
O
10.53
8.38
8.38
8.08
8.07
7.84
7.84
7.76
7.76
7.75
7.75
7.62
7.61
7.60
7.59
7.59
7.58
6.98
2.50 Dimethyl Sulfoxide-d6
H (d)
8.08
E (m)
8.02
C (dd)
7.76
I (s)
10.53
F (d)
8.38
B (m)
7.59
A (d)
6.99
D (dd)
7.85
G (m)
8.20
1.03
0.98
0.99
1.00
1.02
0.98
1.01
3.01
1.00
f1 (ppm)

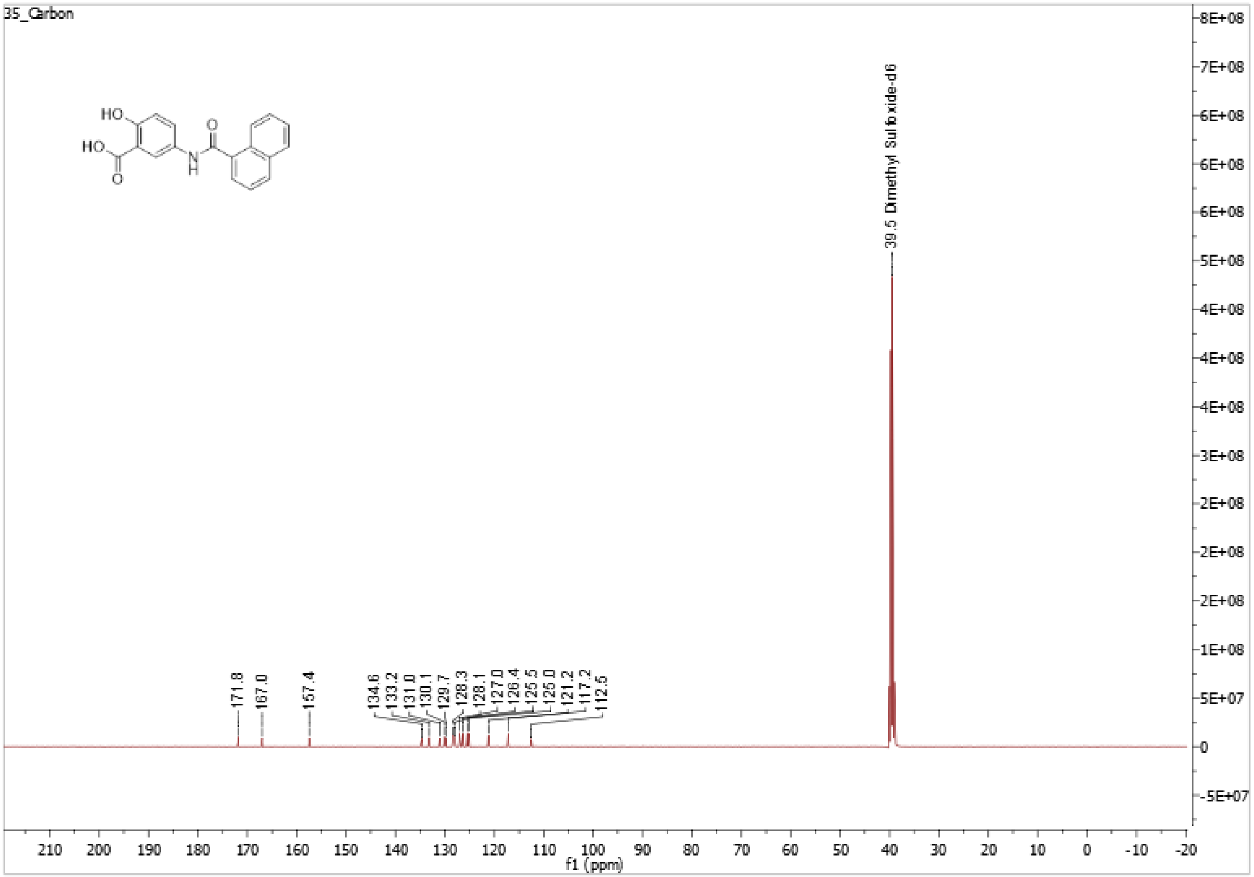
35_Carbon
HO
HO
O
N
H
O
39.5 Dimethyl Sulfoxide-d6
171.8
167.0
157.4
134.6
133.2
131.0
130.1
129.7
128.3
128.1
127.0
126.4
125.5
125.0
121.2
117.2
112.5
f1 (ppm)

Compound 36

HR-MS, ^{1}H-NMR, ^{13}C-NMR

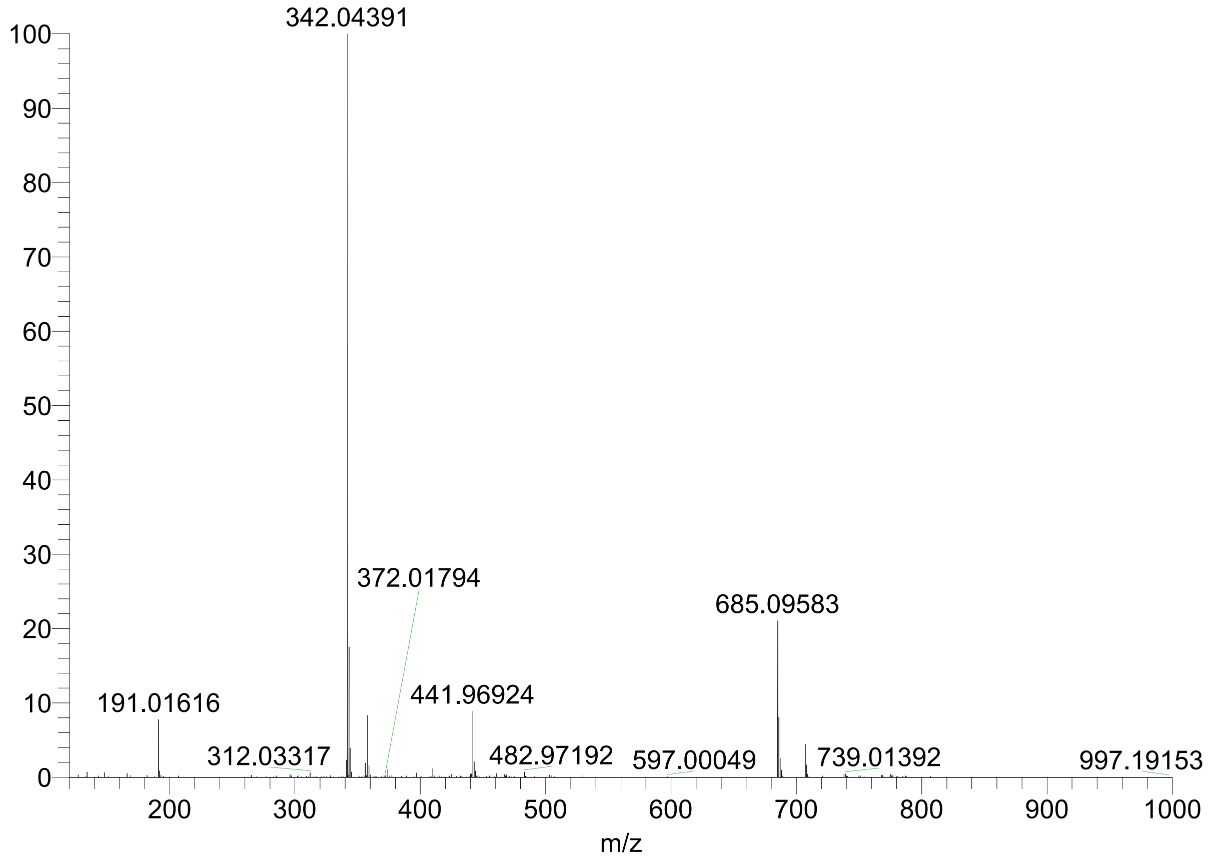
342.04391
100
90
80
70
60
50
40
30
20
10
0
372.01794
685.09583
191.01616
441.96924
312.03317
482.97192
597.00049
739.01392
997.19153
200
300
400
500
600
700
800
900
1000
m/z

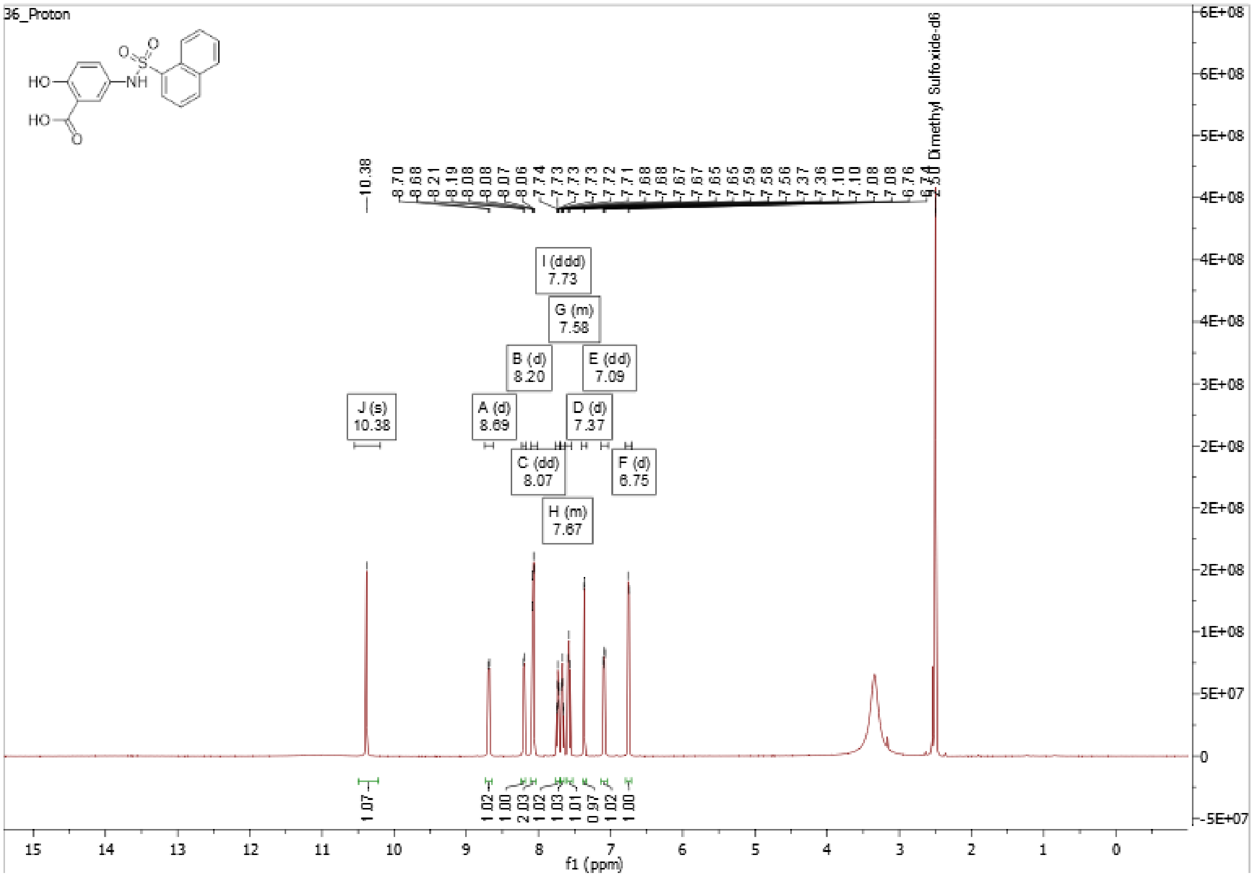
36_Proton
HO
HO
O
O
S
O
NH
10.38
8.70
8.68
8.21
8.19
8.08
8.08
8.07
8.06
7.74
7.73
7.73
7.73
7.72
7.71
7.68
7.68
7.67
7.67
7.65
7.65
7.59
7.58
7.56
7.37
7.36
7.10
7.10
7.08
7.08
6.76
6.74
2.50 Dimethyl Sulfoxide-d6
I (ddd)
7.73
G (m)
7.58
B (d)
8.20
E (dd)
7.09
J (s)
10.38
A (d)
8.69
D (d)
7.37
C (dd)
8.07
F (d)
6.75
H (m)
7.67
1.07
1.02
1.00
2.03
1.02
1.03
1.01
0.97
1.02
1.00
f1 (ppm)

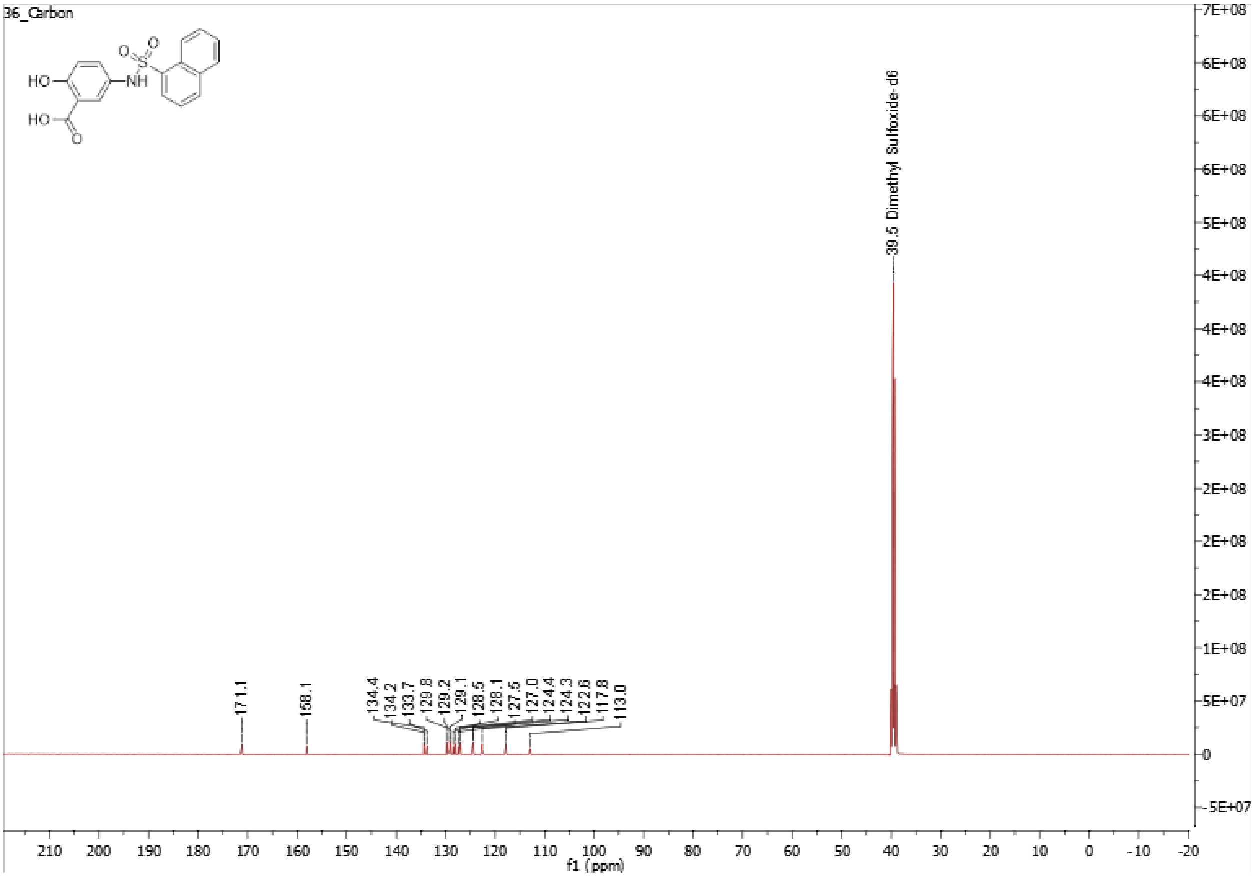

Compound 37

HR-MS, HPLC-MS, ^{1}H-NMR, ^{13}C-NMR

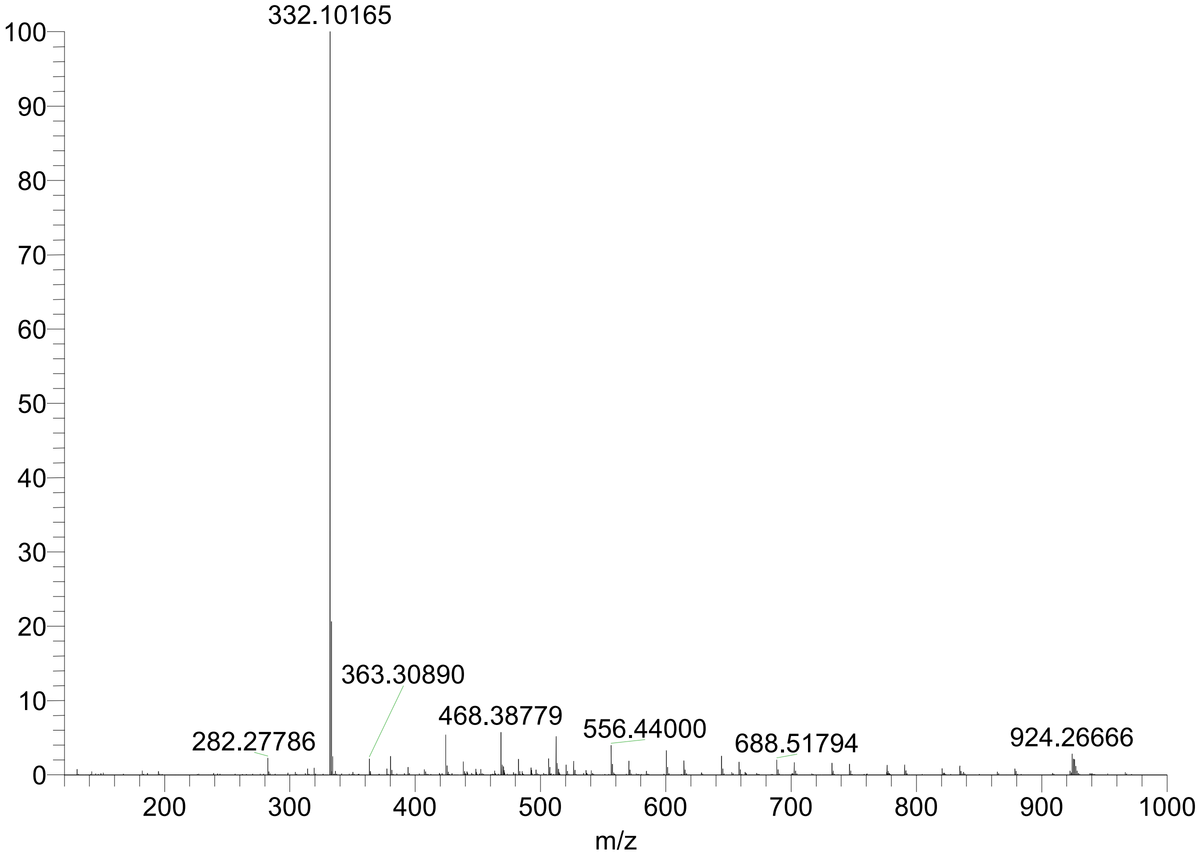

332.10165
363.30890
468.38779
556.44000
282.27786
688.51794
924.26666
m/z

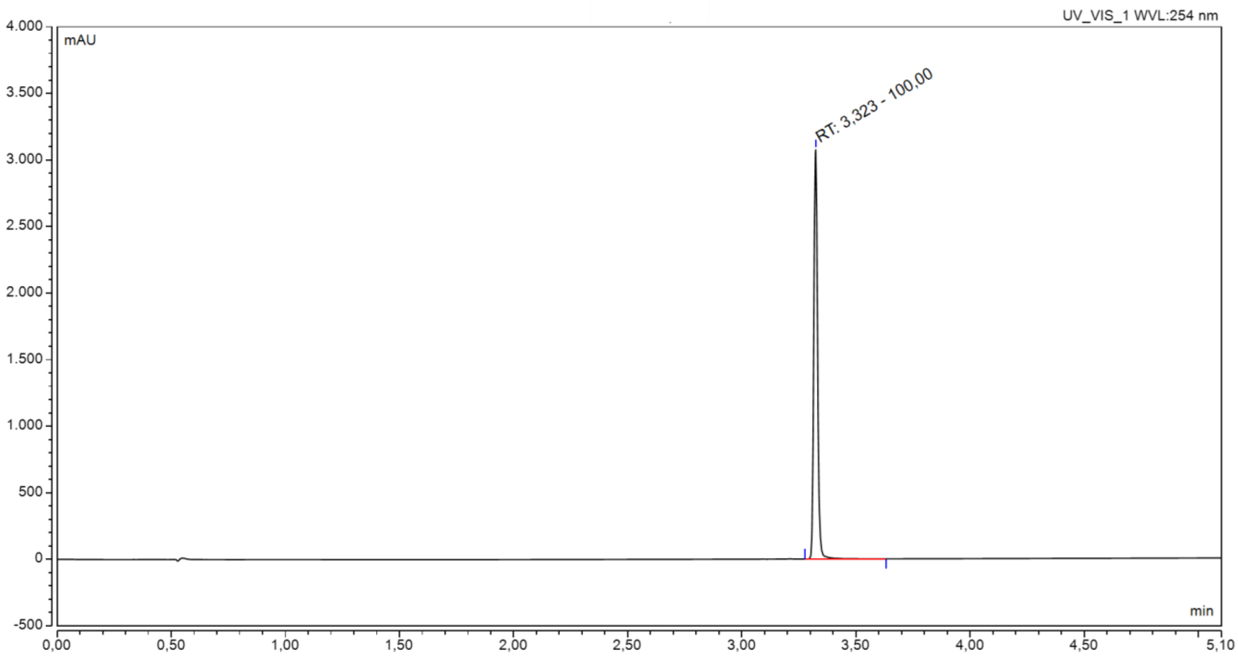

UV_VIS_1 WVL:254 nm
mAU
RT: 3,323 - 100,00
min

Purity = 100%

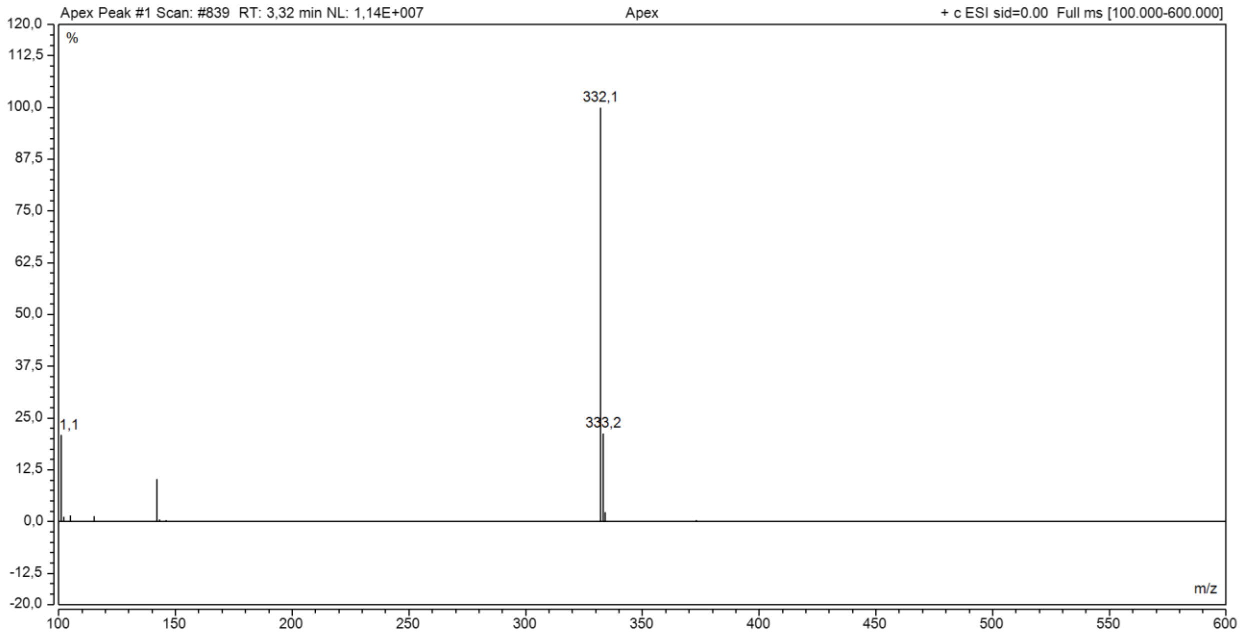

Apex Peak #1 Scan: #839 RT: 3,32 min NL: 1,14E+007
Apex
+ c ESI sid=0.00 Full ms [100.000-600.000]
332,1
333,2
m/z

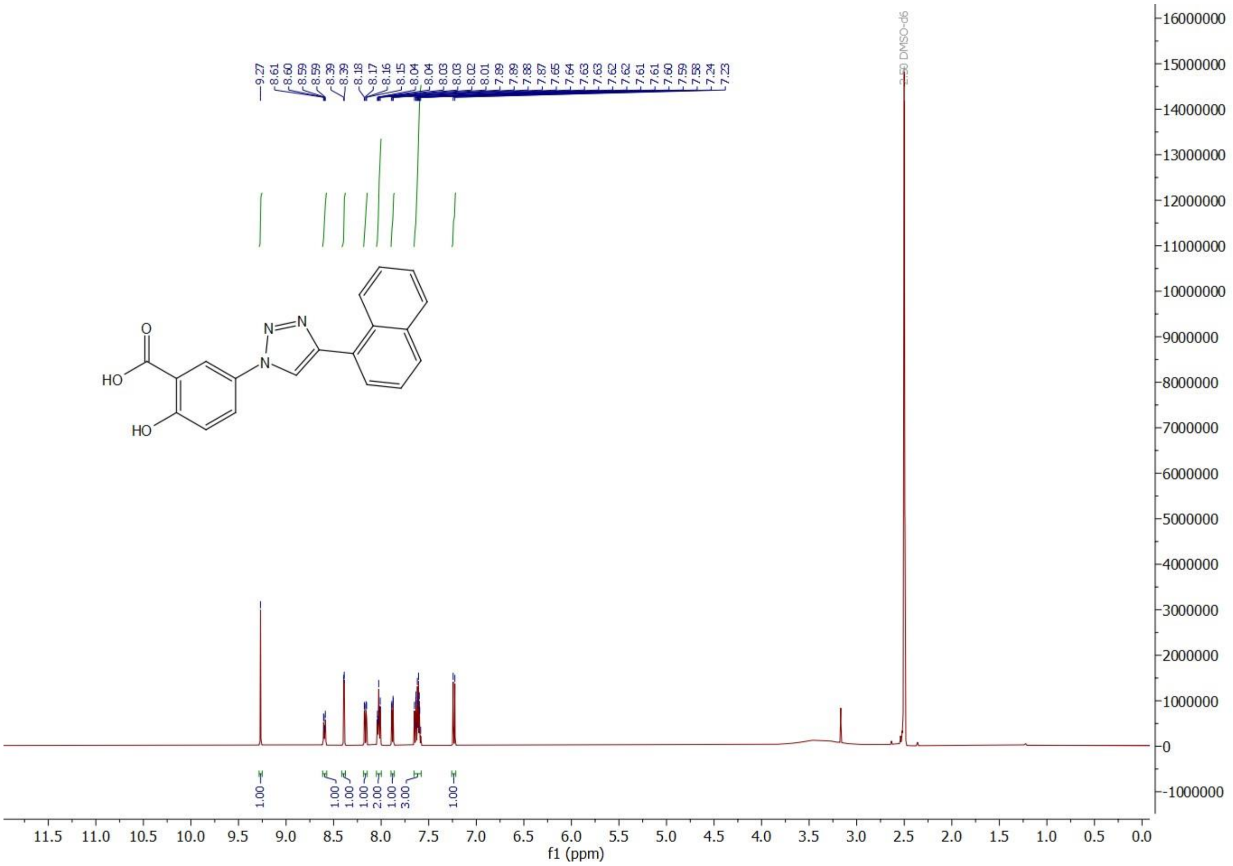

2.50 DMSO-d6
9.27
8.61
8.60
8.59
8.59
8.39
8.39
8.18
8.17
8.16
8.15
8.04
8.04
8.03
8.03
8.02
8.01
7.89
7.89
7.88
7.87
7.65
7.64
7.63
7.63
7.62
7.62
7.61
7.61
7.60
7.59
7.58
7.24
7.23
HO
HO
O
N
N
N
1.00
1.00
1.00
1.00
2.00
1.00
3.00
1.00
f1 (ppm)

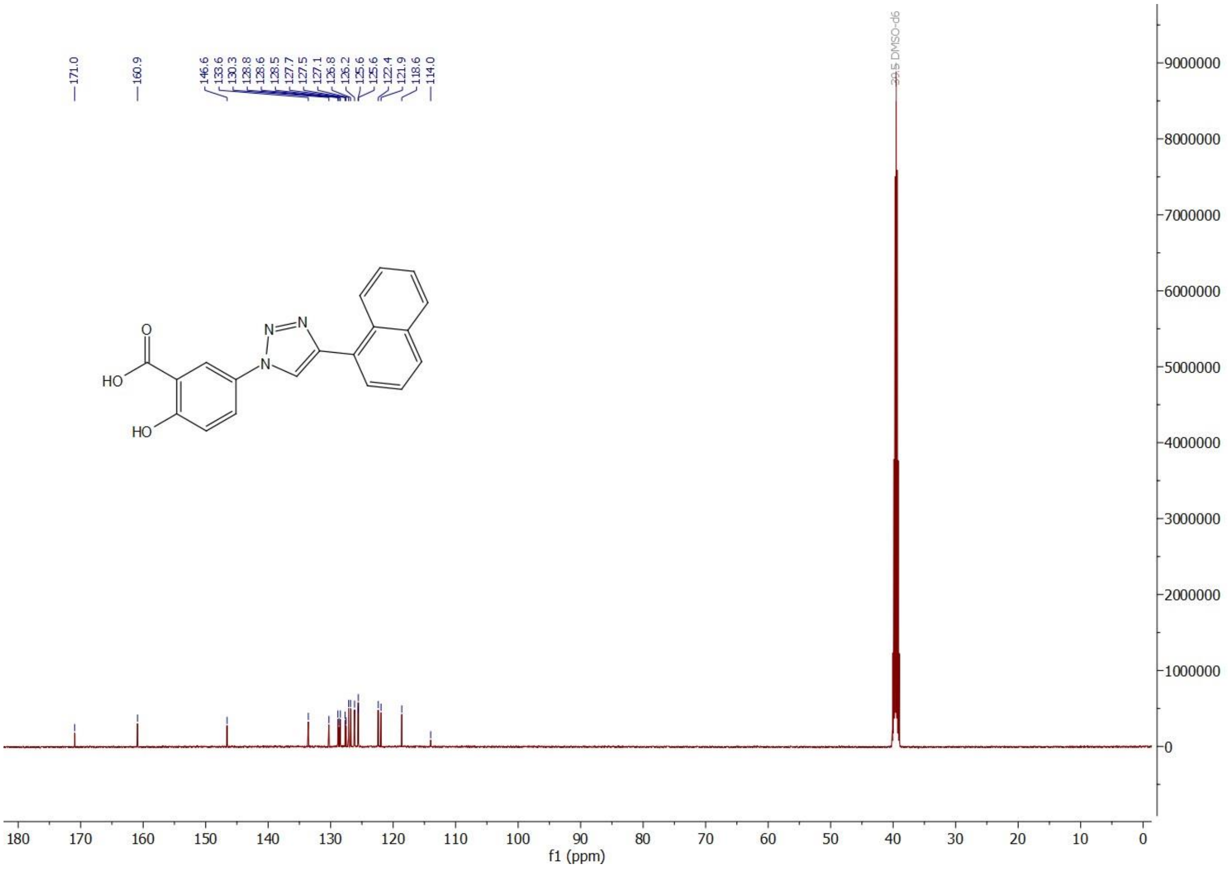

Compound 38

HR-MS, HPLC-MS, ^{1}H-NMR, ^{13}C-NMR

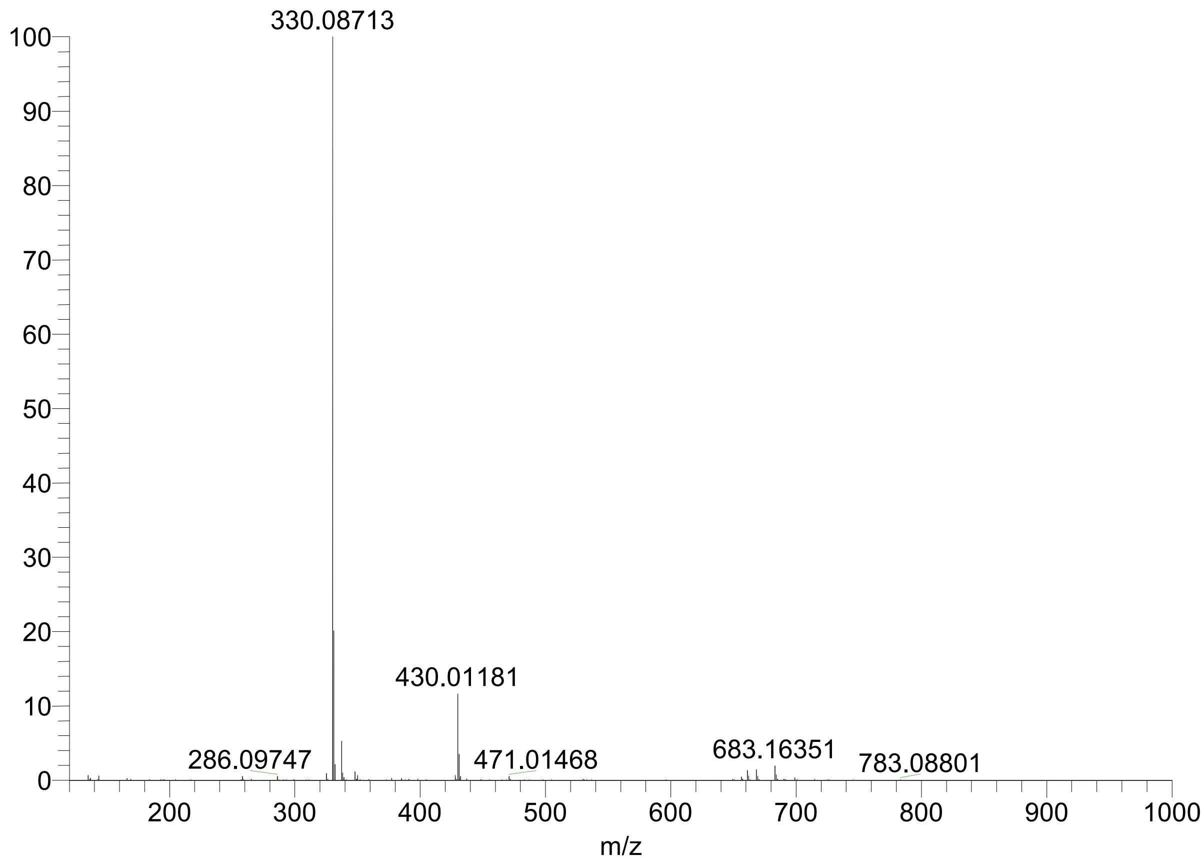

330.08713
430.01181
286.09747
471.01468
683.16351
783.08801
m/z

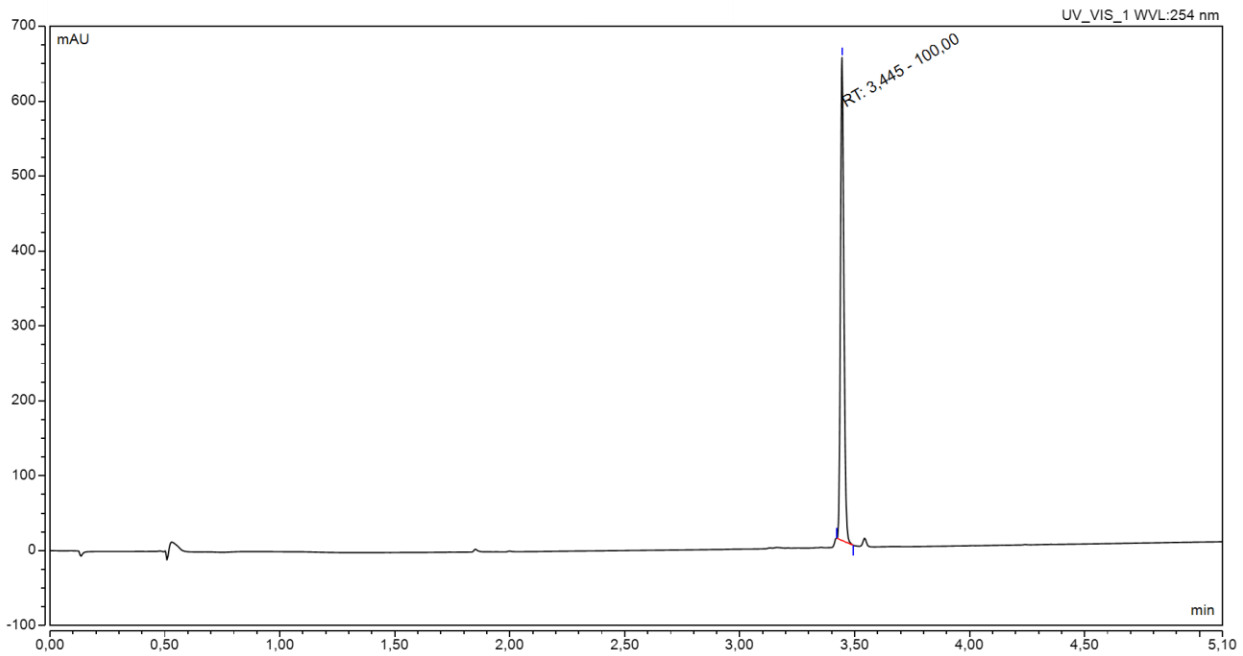

UV_VIS_1 WVL:254 nm
mAU
RT: 3.445 - 100,00
min

Purity = 100%

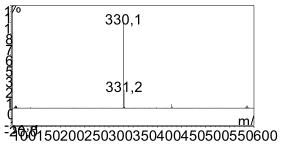

330,1
331,2
m/

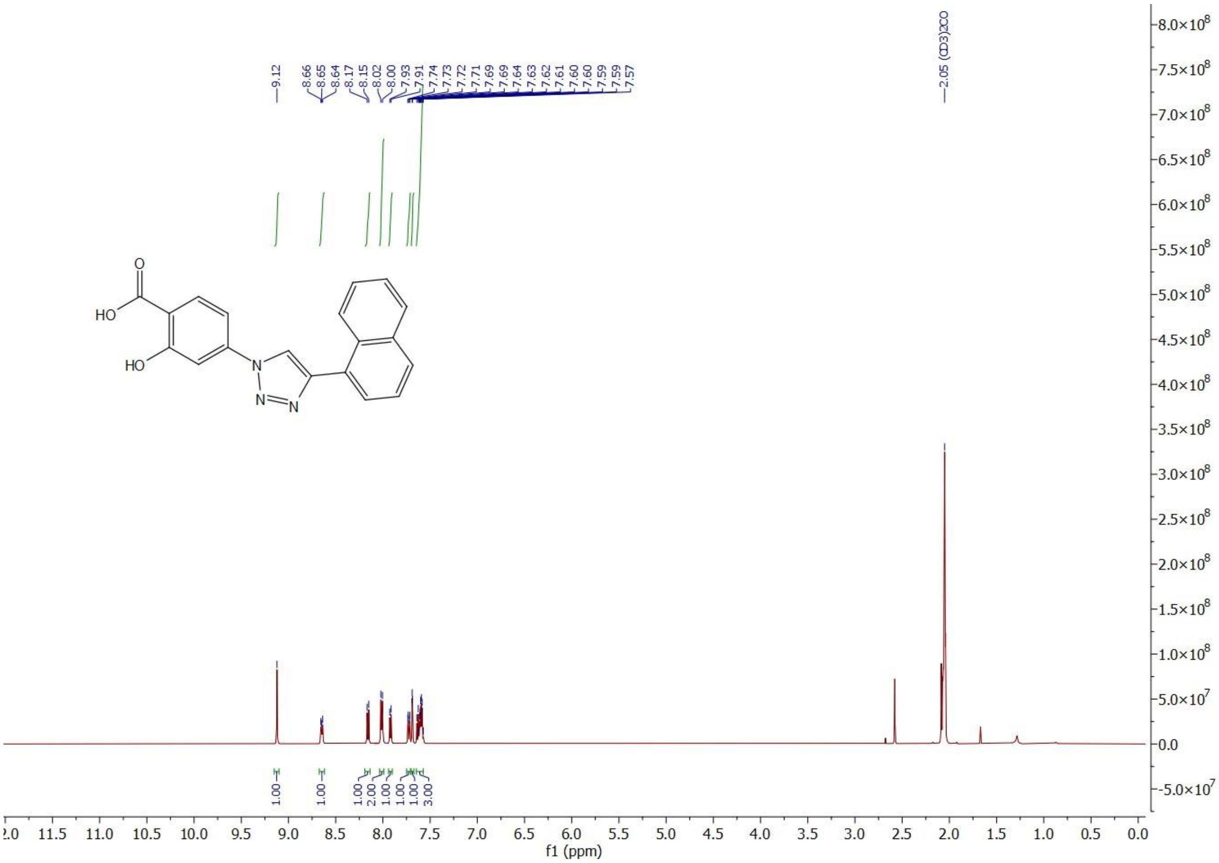

9.12
8.66
8.65
8.64
8.17
8.15
8.02
8.00
7.93
7.91
7.74
7.73
7.72
7.71
7.69
7.69
7.64
7.63
7.62
7.61
7.60
7.59
7.59
7.57
2.05 (CD3)2CO
HO
HO
O
N
N
N
1.00
1.00
1.00
2.00
1.00
1.00
1.00
1.00
3.00
f1 (ppm)

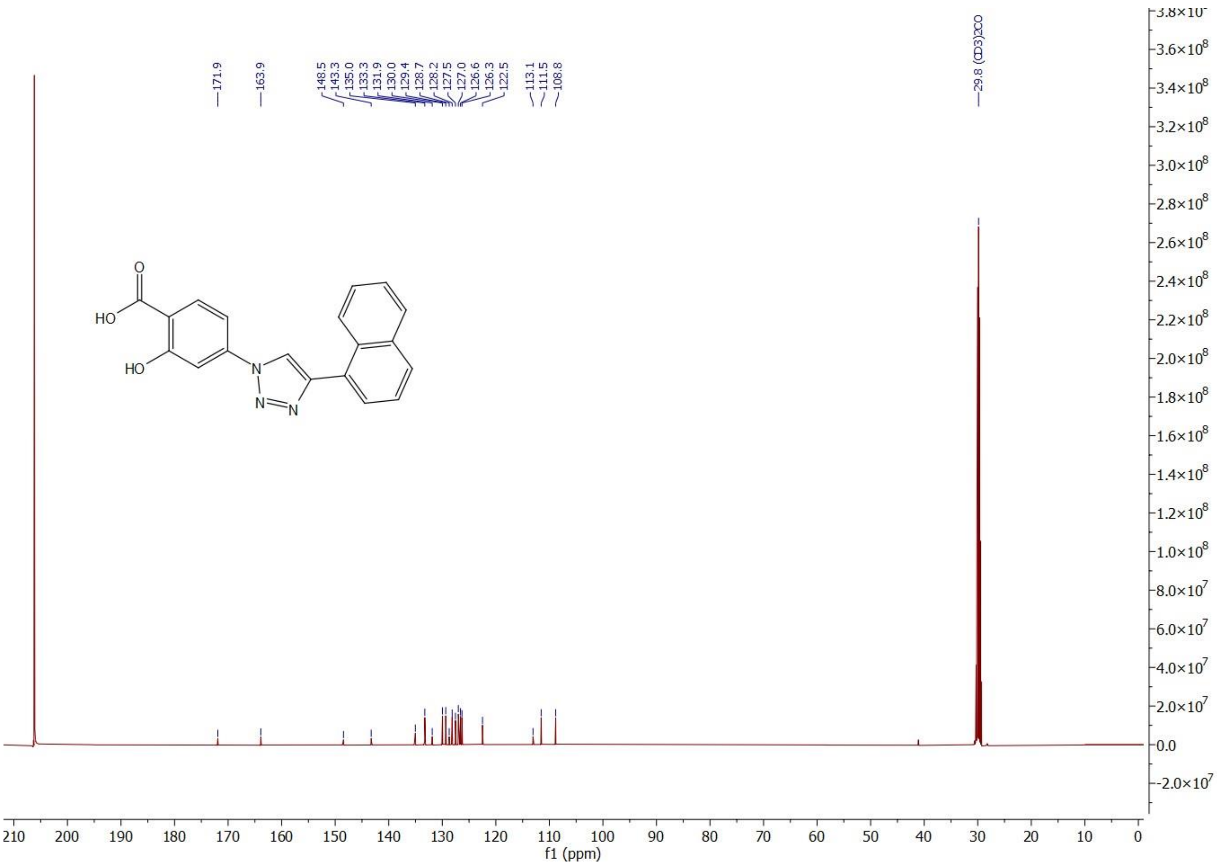

Compound 41

HR-MS, ^{1}H-NMR, ^{13}C-NMR

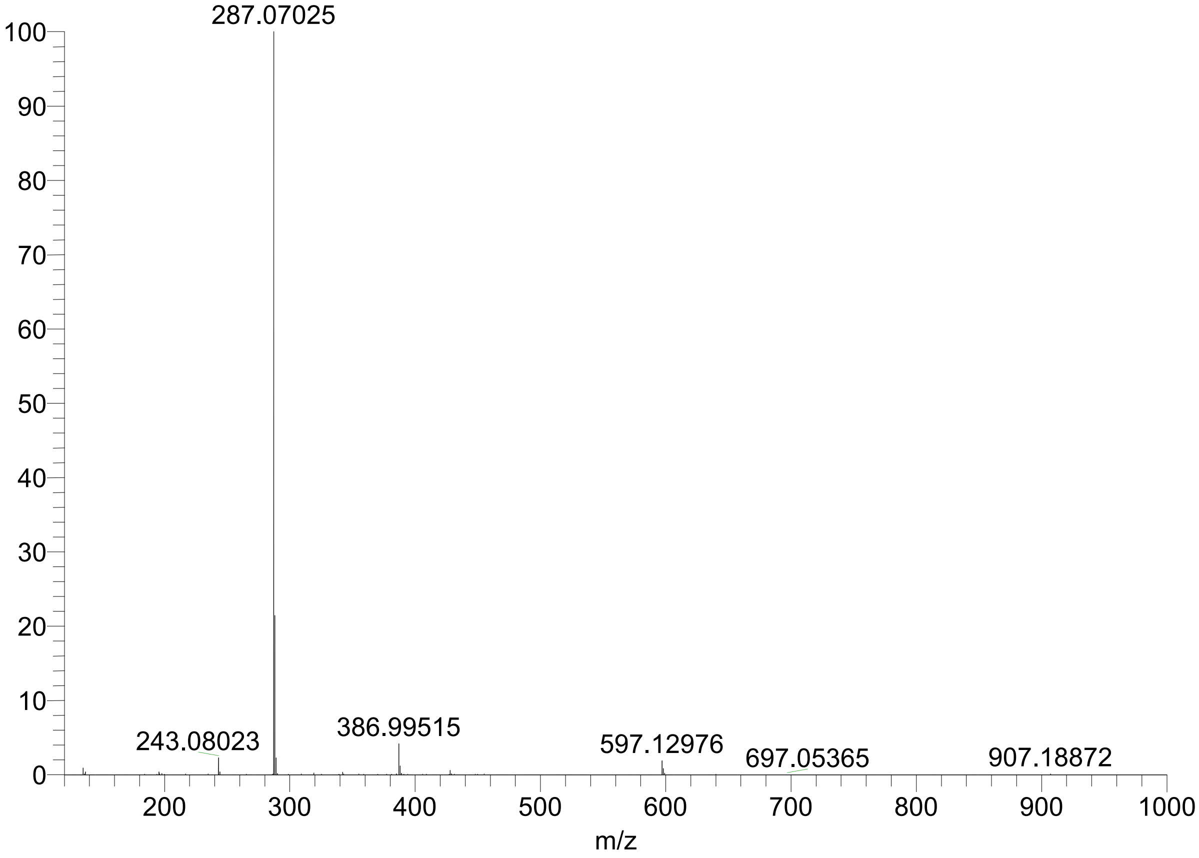
287.07025
243.08023
386.99515
597.12976
697.05365
907.18872
100
90
80
70
60
50
40
30
20
10
0
200
300
400
500
600
700
800
900
1000
m/z

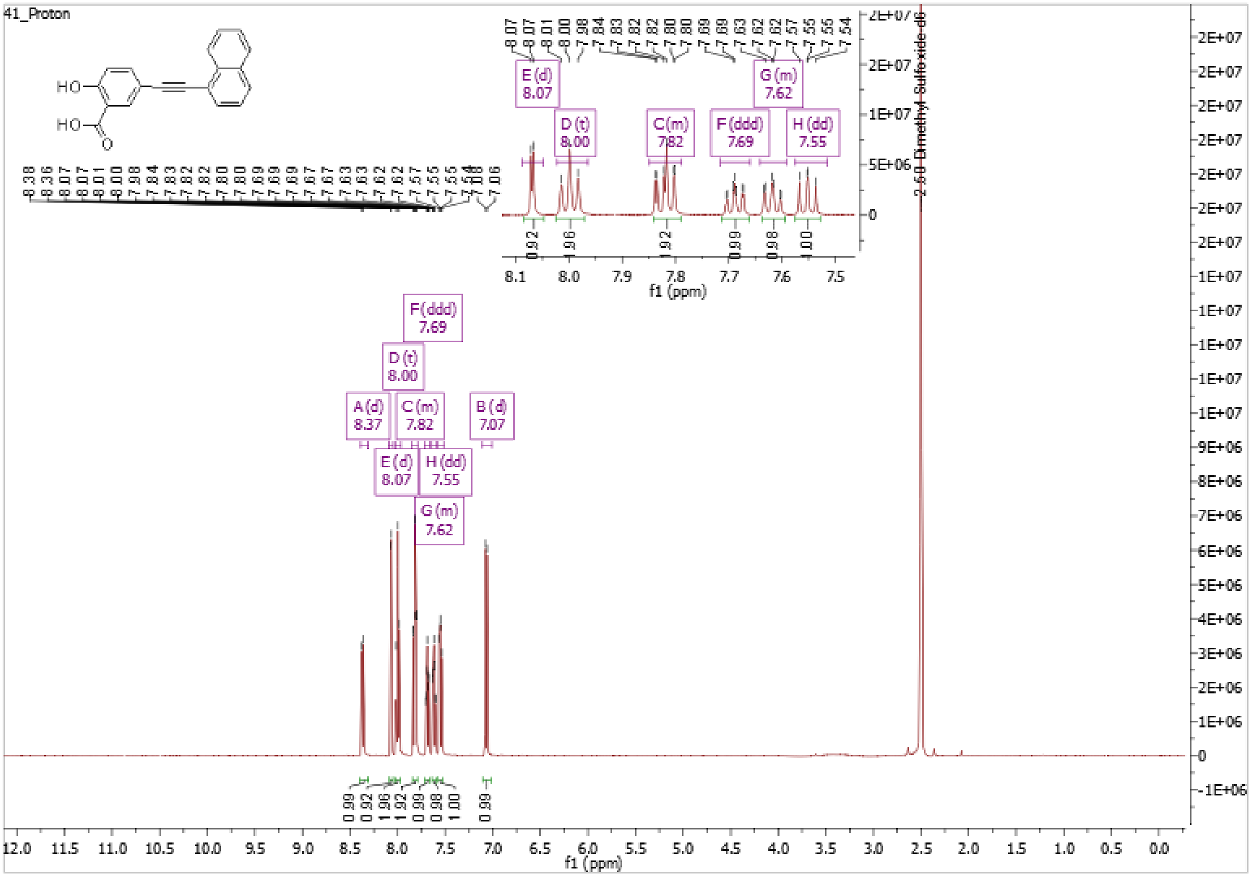
41_Proton
HO
HO
O
A (d)
8.37
B (d)
7.07
C (m)
7.82
D (t)
8.00
E (d)
8.07
F (ddd)
7.69
G (m)
7.62
H (dd)
7.55
2.50 Dimethyl Sulfoxide-d6
f1 (ppm)

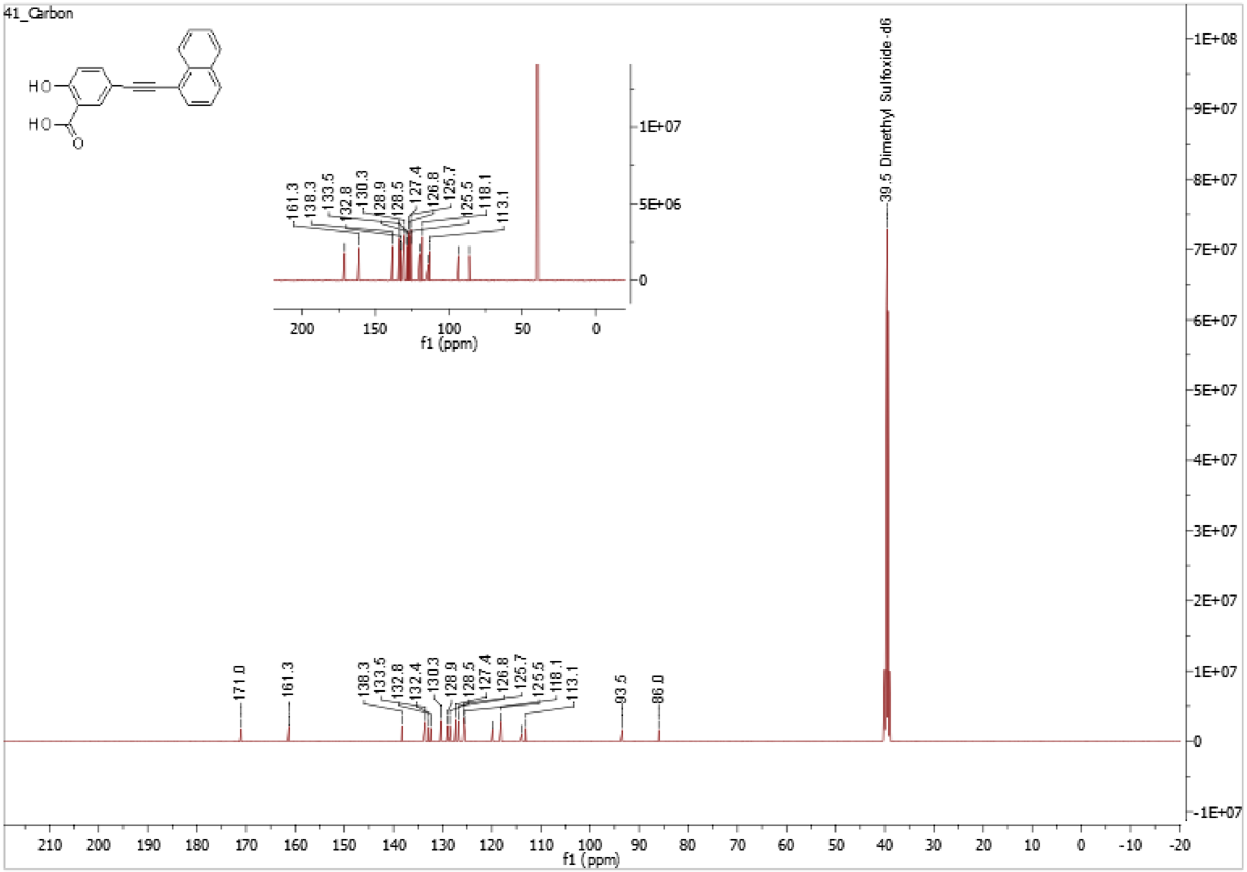

Compound 42

HR-MS, HPLC-MS, ^{1}H-NMR, ^{13}C-NMR

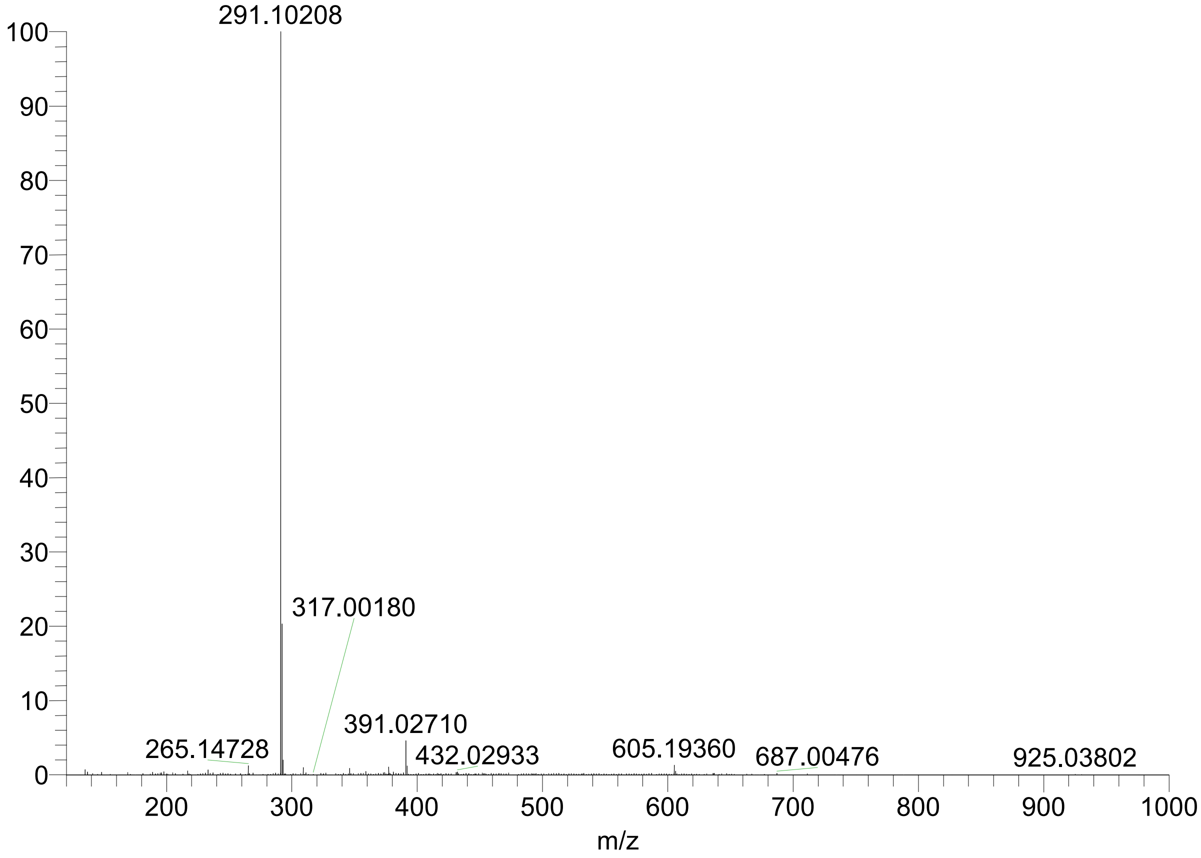
291.10208
317.00180
391.02710
265.14728
432.02933
605.19360
687.00476
925.03802
m/z

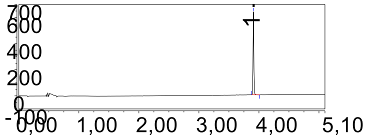

Purity = 100%

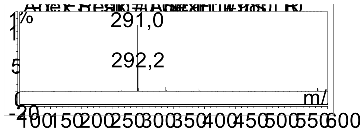
291,0
292,2
m/
100 150 200 250 300 350 400 450 500 550 600

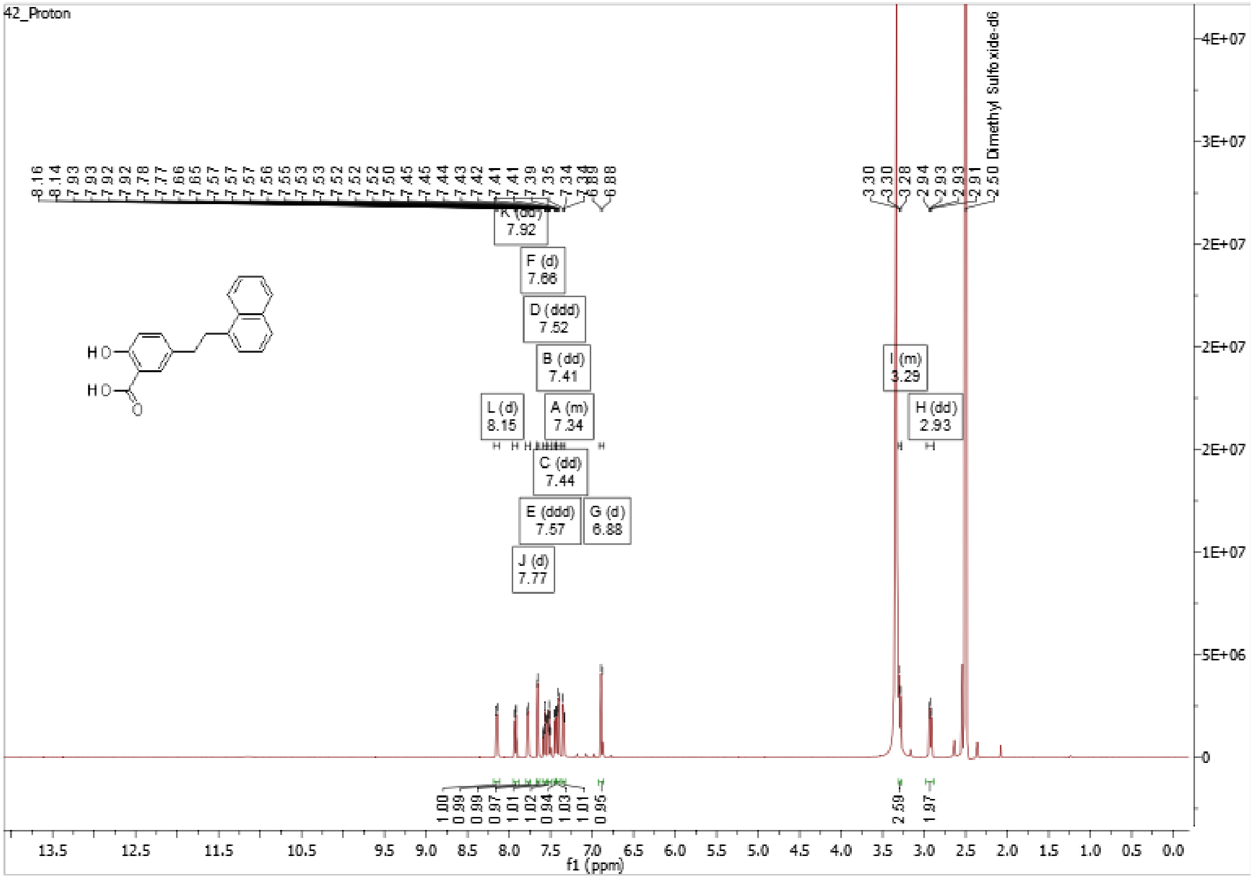
42_Proton
Dimethyl Sulfoxide-d6
K (dd) 7.92
F (d) 7.66
D (ddd) 7.52
B (dd) 7.41
L (d) 8.15
A (m) 7.34
C (dd) 7.44
E (ddd) 7.57
G (d) 6.88
J (d) 7.77
I (m) 3.29
H (dd) 2.93
HO
HO
O
1.00
0.99
0.99
0.97
1.01
1.02
0.94
1.03
1.01
0.95
2.59
1.97
f1 (ppm)

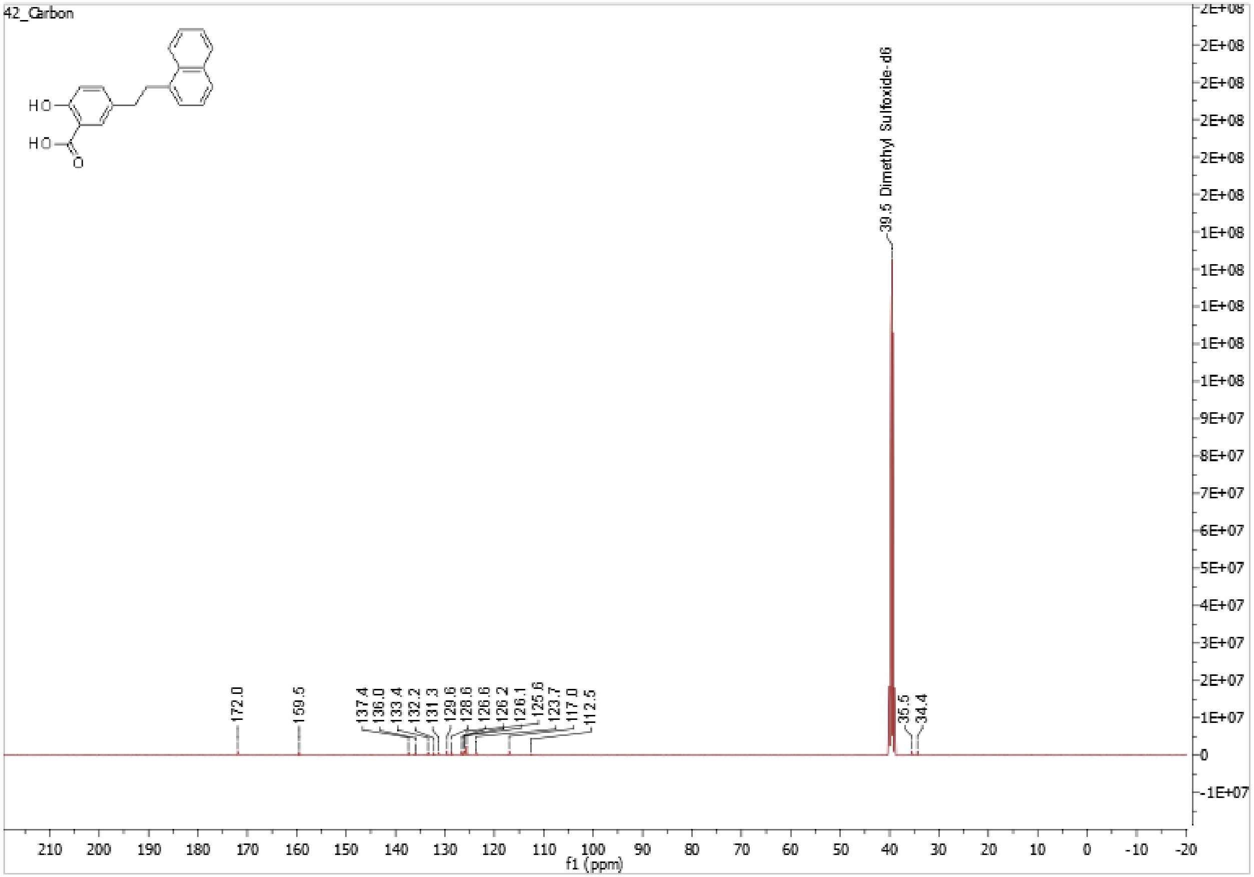

Compound 46

HR-MS, HPLC-MS, ^{1}H-NMR, ^{13}C-NMR

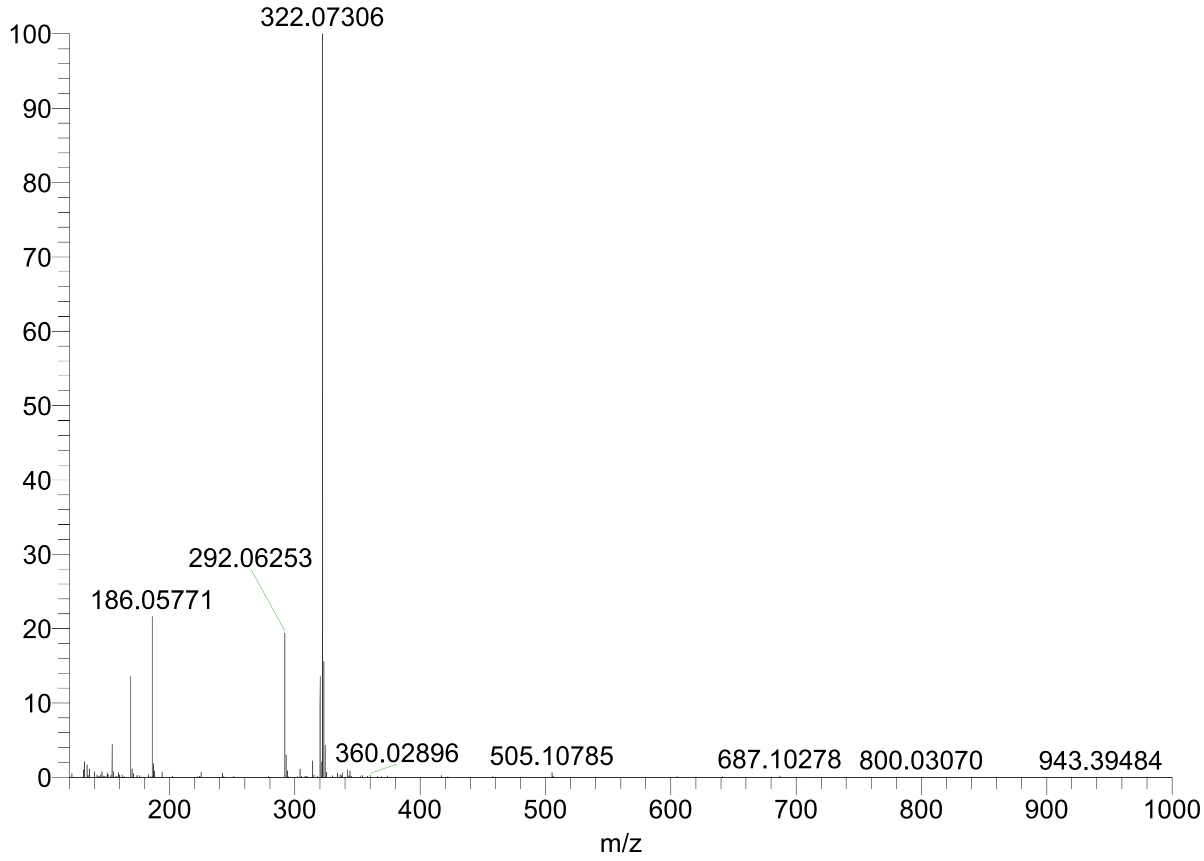
322.07306
292.06253
186.05771
360.02896
505.10785
687.10278
800.03070
943.39484
m/z

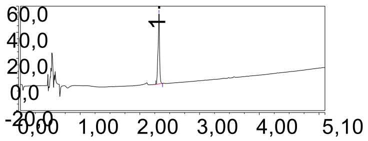

Purity = 100%

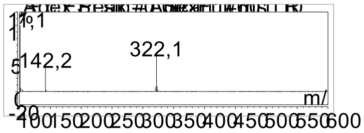
142,2
322,1
m/
100150200250300350400450500550600

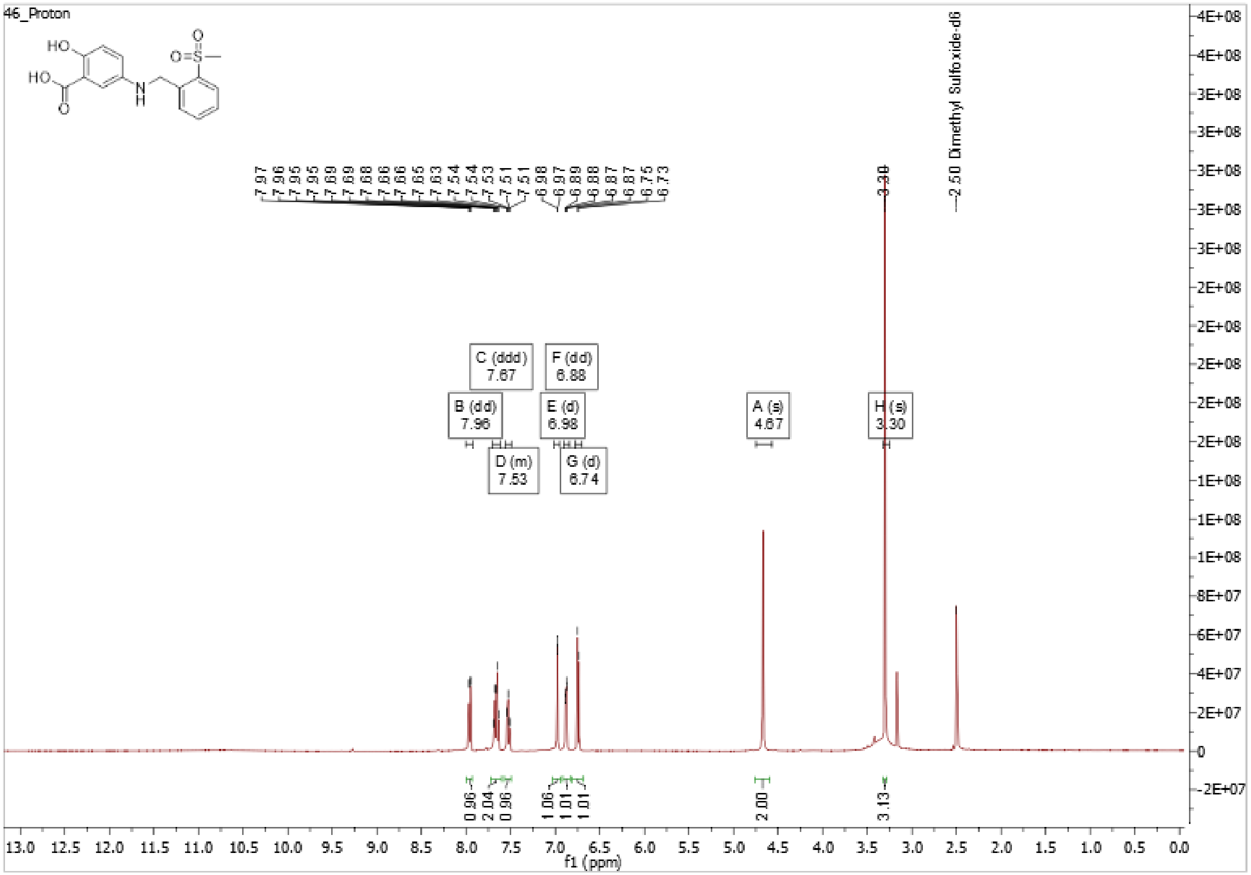
46_Proton
HO
HO
O
N
H
O=S
O
2.50 Dimethyl Sulfoxide-d6
C (ddd) 7.67
F (dd) 6.88
B (dd) 7.96
E (d) 6.98
A (s) 4.67
H (s) 3.30
D (m) 7.53
G (d) 6.74
f1 (ppm)

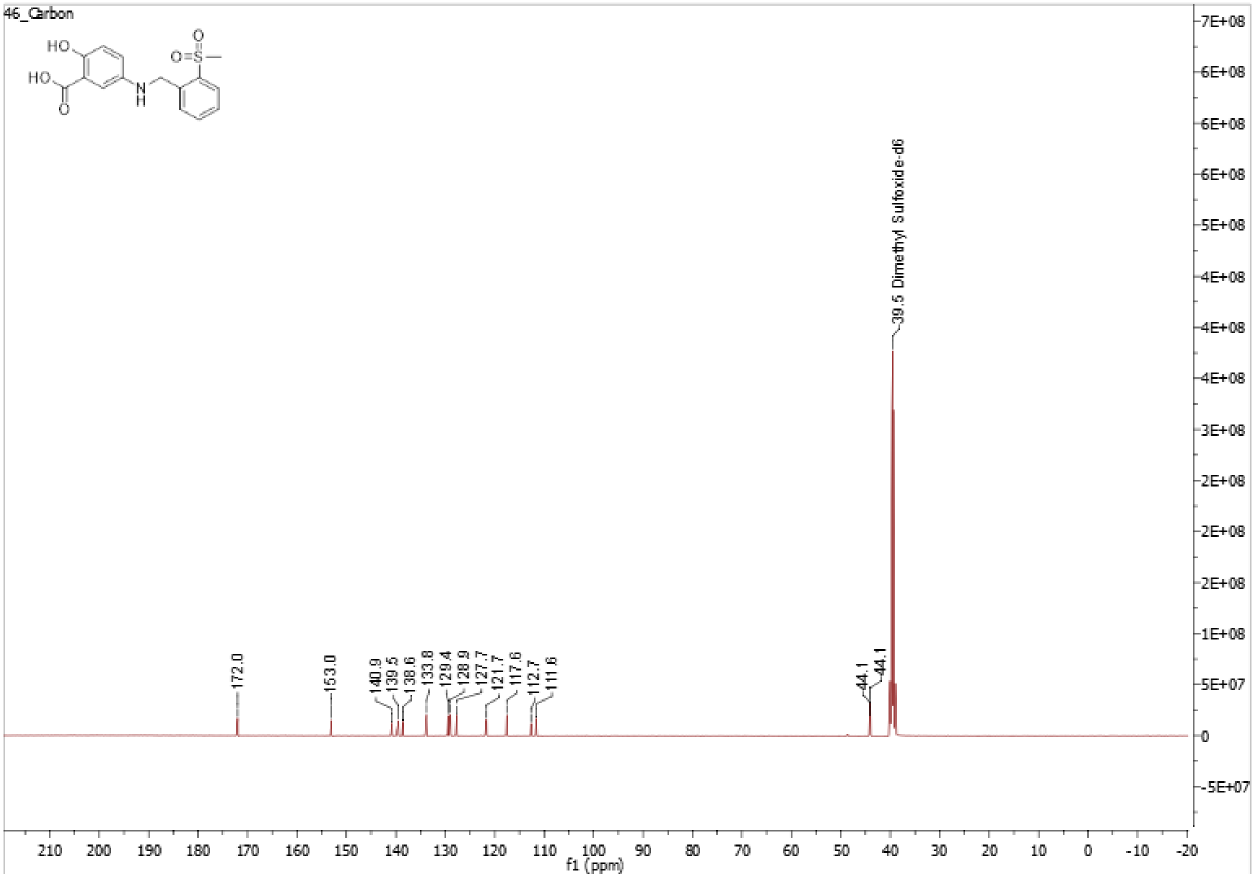

Compound 49

HR-MS, HPLC-MS, ^{1}H-NMR, ^{13}C-NMR

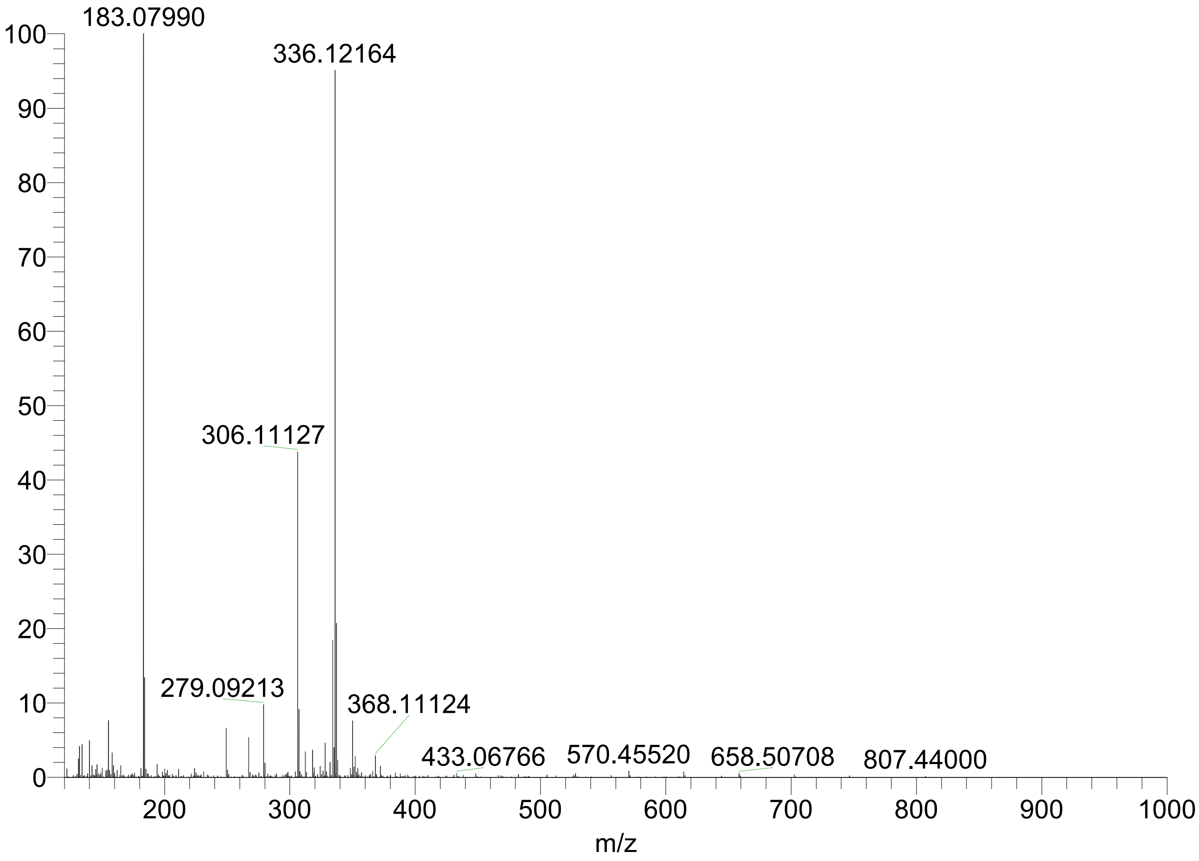
183.07990
336.12164
306.11127
279.09213
368.11124
433.06766
570.45520
658.50708
807.44000
m/z

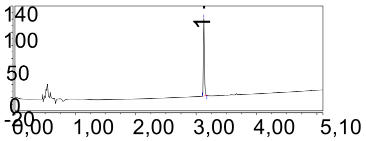

Purity = 100%

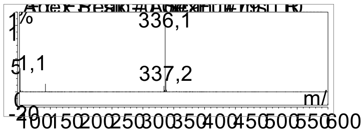

336,1
337,2
m/
100 150 200 250 300 350 400 450 500 550 600

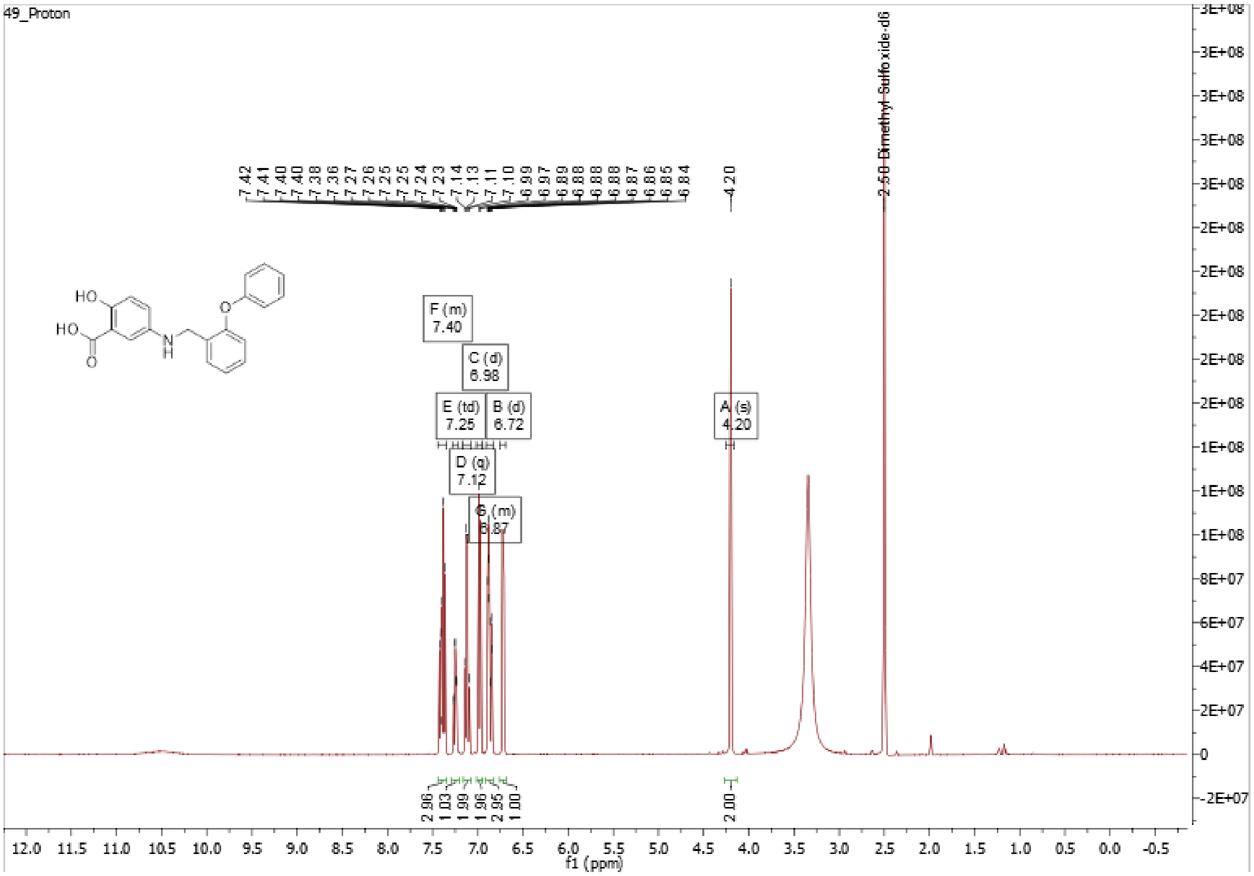

49_Proton
2.50 Dimethyl Sulfoxide-d6
4.20
F (m) 7.40
C (d) 6.98
E (td) 7.25
B (d) 6.72
D (q) 7.12
G (m) 6.87
A (s) 4.20
HO
HO
O
N
H
O
f1 (ppm)

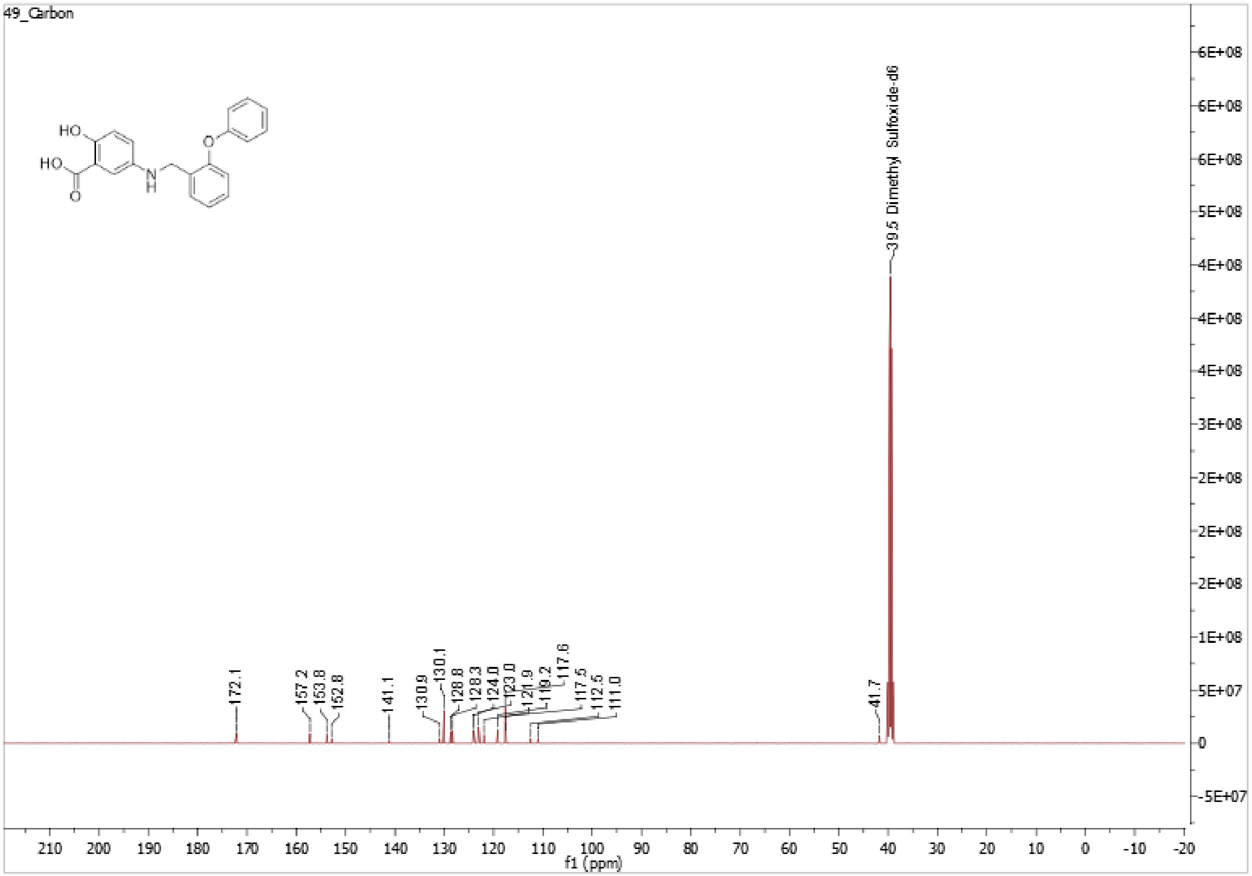

Compound 52

HPLC-MS, ^{1}H-NMR, ^{13}C-NMR

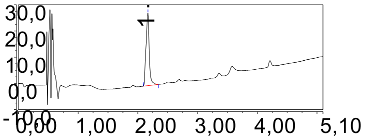
30,0
20,0
10,0
0,0
-10,0
0,00
1,00
2,00
3,00
4,00
5,10

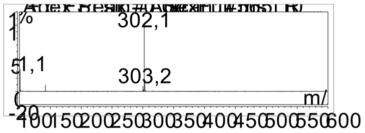
%
302,1
303,2
m/
100 150 200 250 300 350 400 450 500 550 600

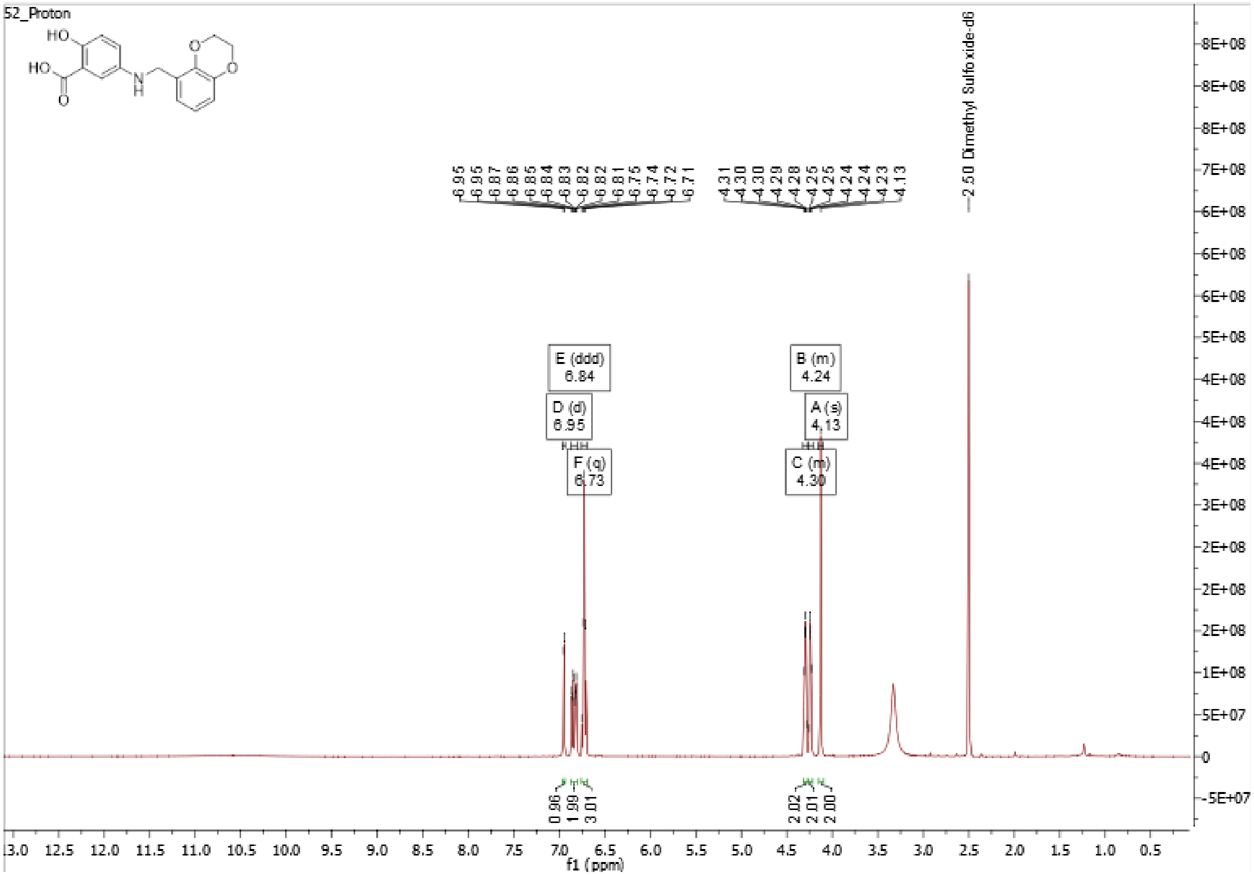
52_Proton
2.50 Dimethyl Sulfoxide-d6
E (ddd) 6.84
D (d) 6.95
F (q) 6.73
B (m) 4.24
A (s) 4.13
C (m) 4.30
f1 (ppm)

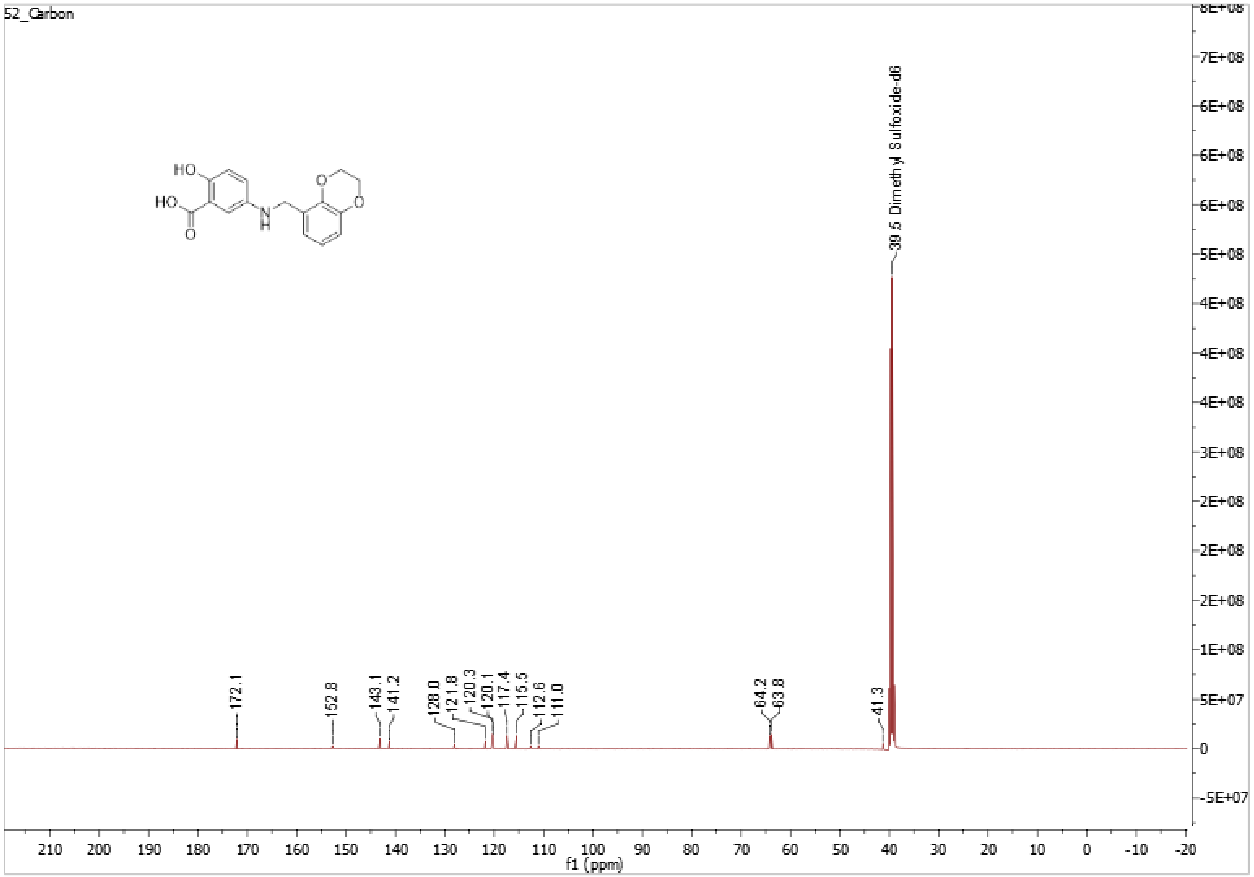

Compound 63

HR-MS, HPLC-MS, ^{1}H-NMR, ^{13}C-NMR

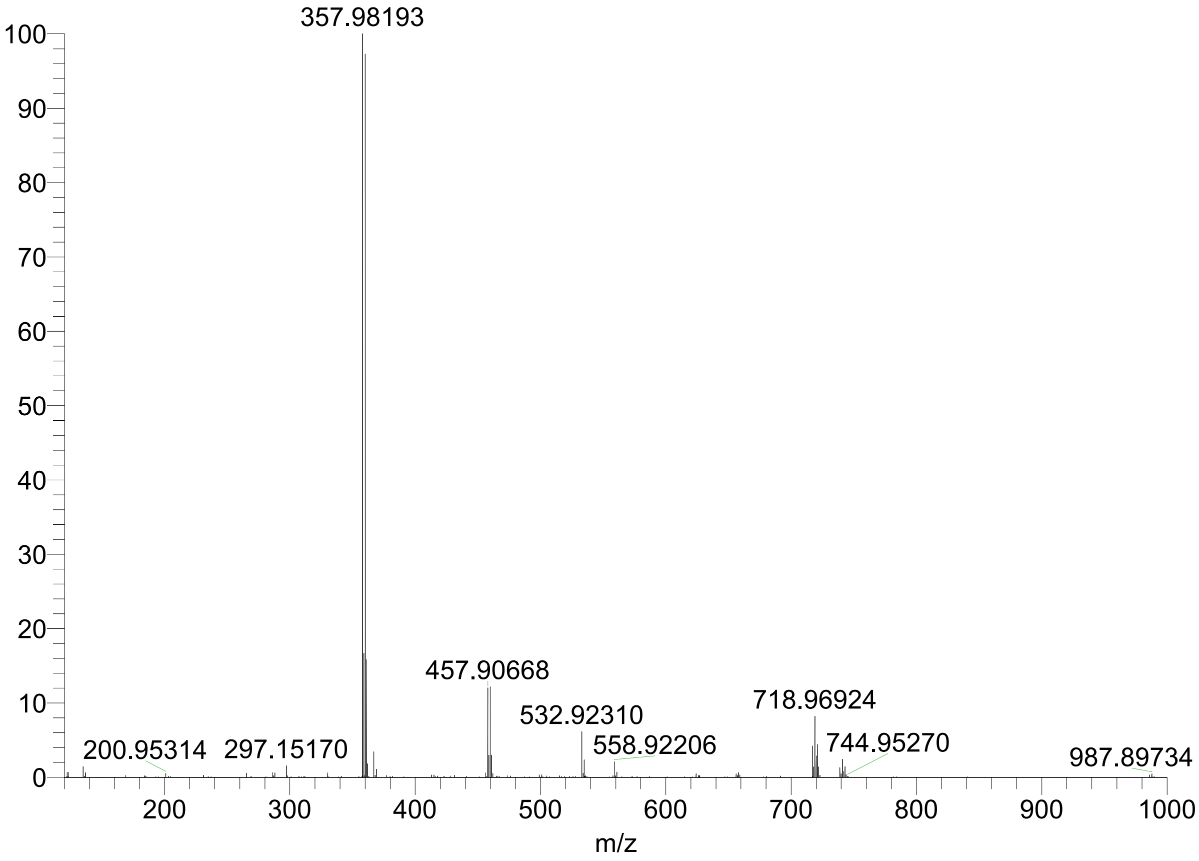
357.98193
457.90668
532.92310
558.92206
718.96924
744.95270
987.89734
200.95314
297.15170
m/z

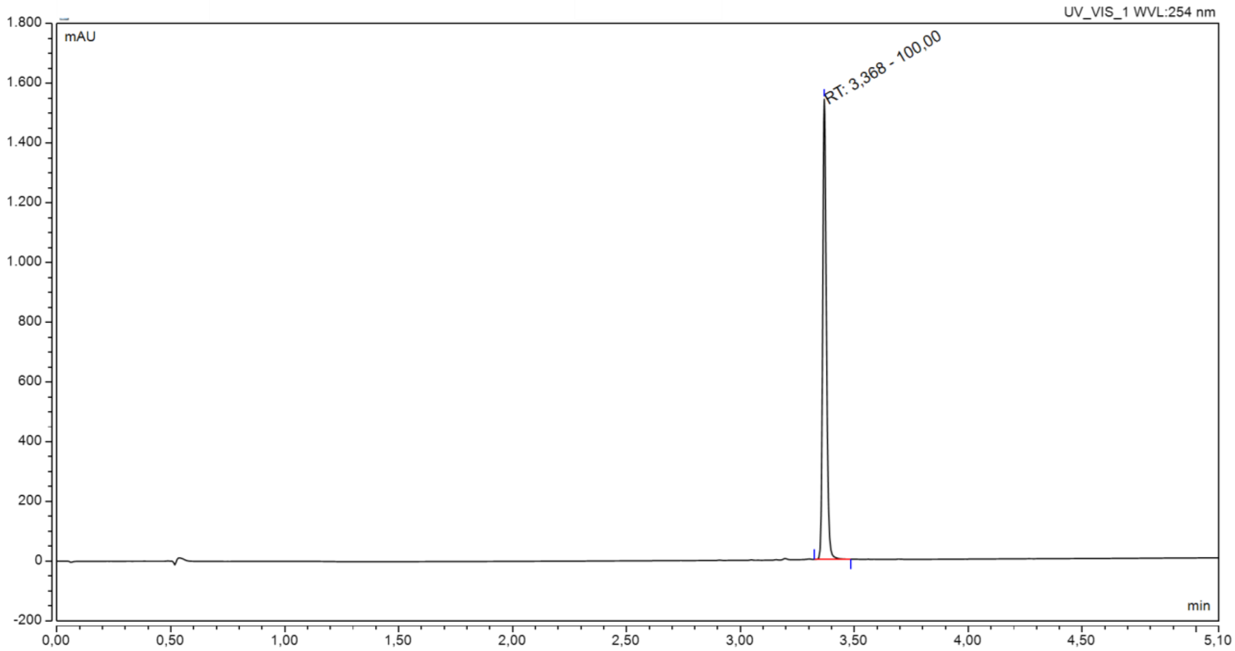
UV_VIS_1 WVL:254 nm
mAU
RT: 3,368 - 100,00
min

Purity = 100%

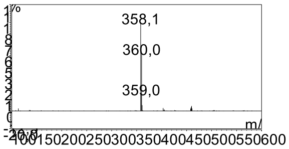
358,1
360,0
359,0
m/

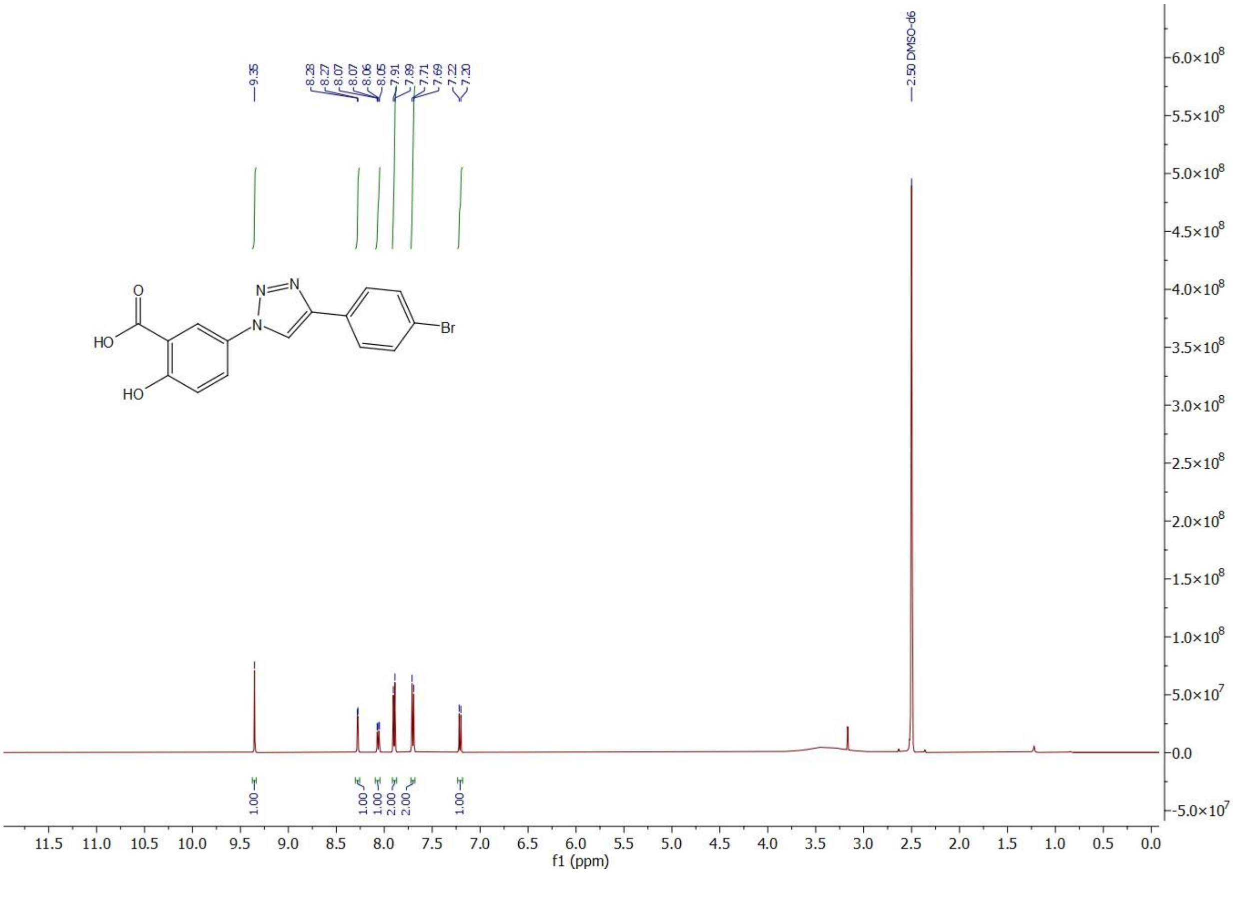
9.35
8.28
8.27
8.07
8.07
8.06
8.05
7.91
7.89
7.71
7.69
7.22
7.20
2.50 DMSO-d6
HO
HO
O
N
N
N
Br
1.00
1.00
1.00
2.00
2.00
1.00
f1 (ppm)

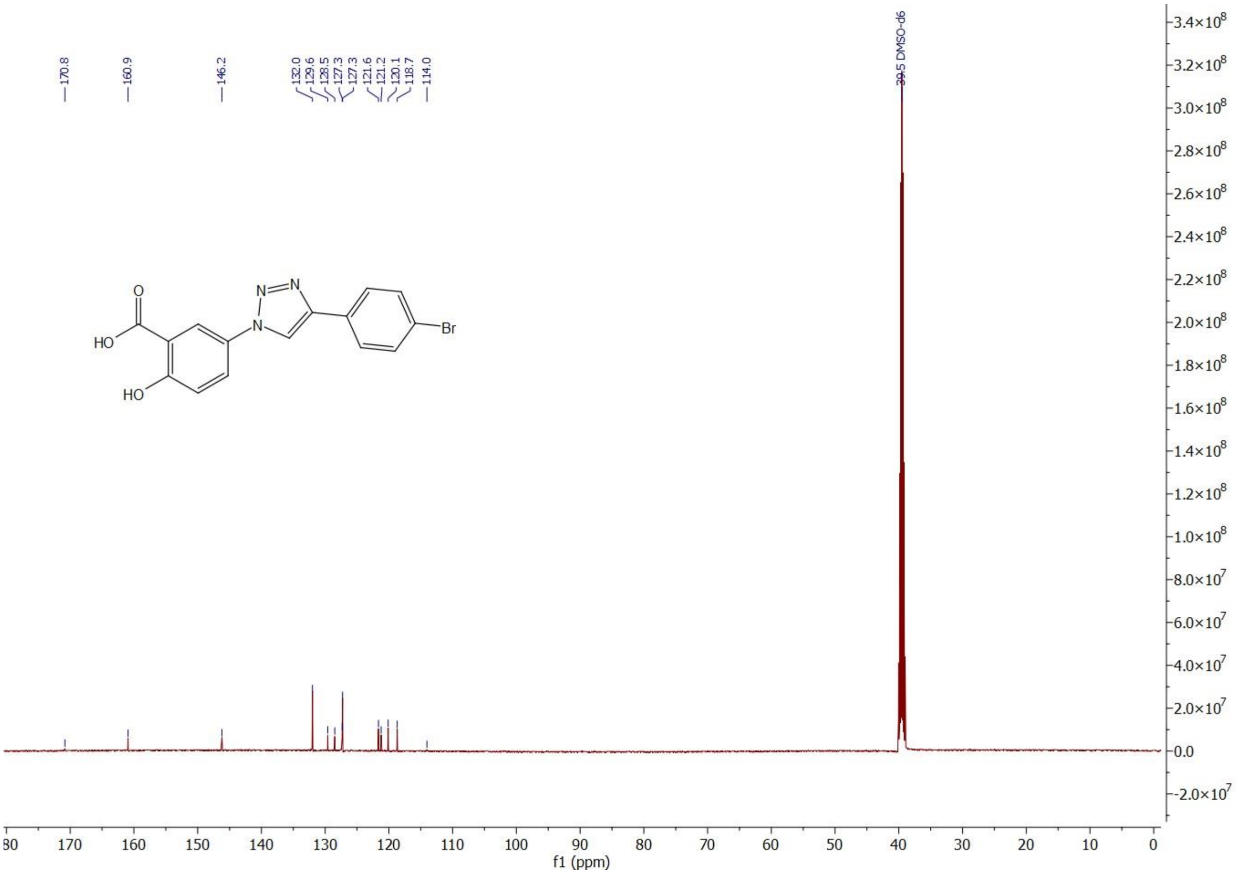

Compound 64

HR-MS, HPLC-MS, ^{1}H-NMR, ^{13}C-NMR

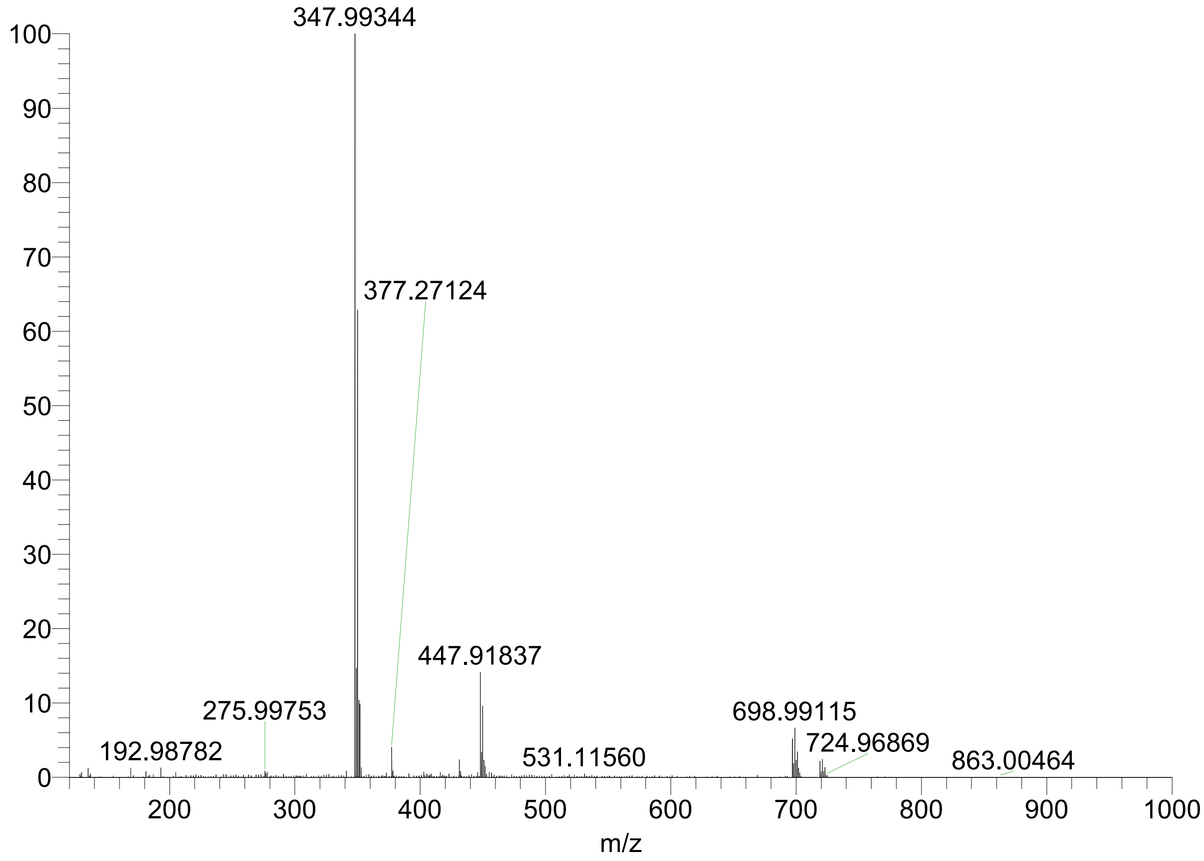
347.99344
377.27124
447.91837
275.99753
192.98782
531.11560
698.99115
724.96869
863.00464
m/z

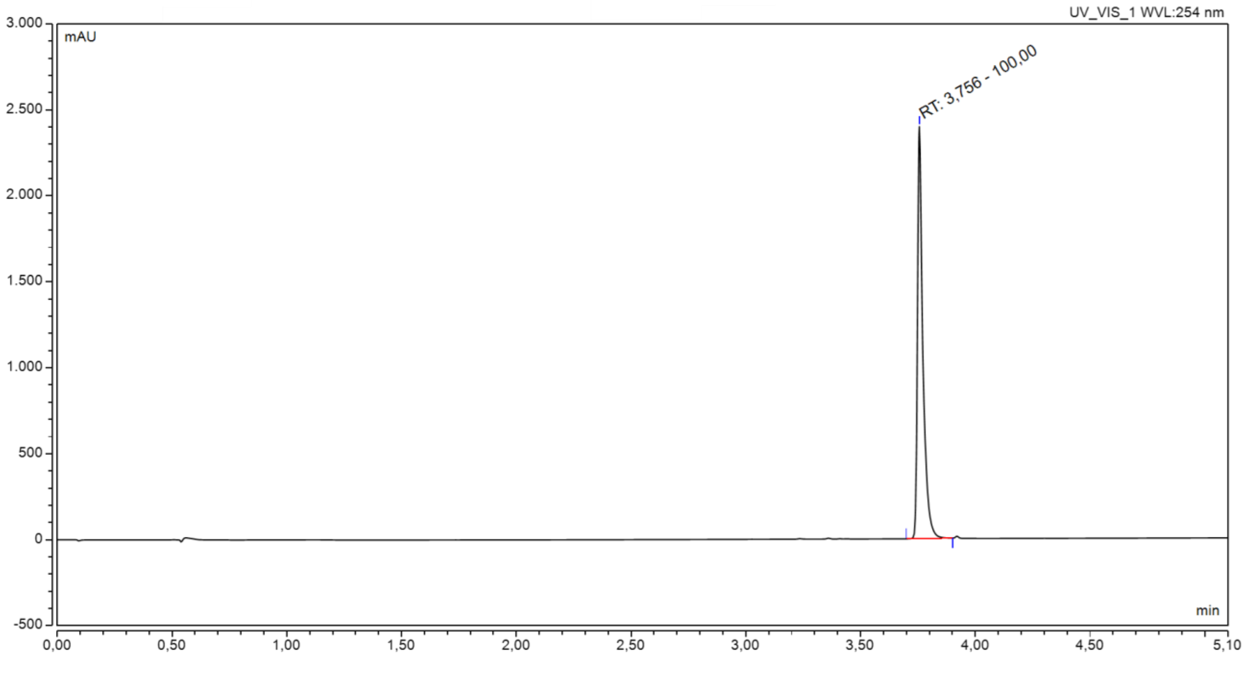
UV_VIS_1 WVL:254 nm
mAU
RT: 3,756 - 100,00
min

Purity = 100%

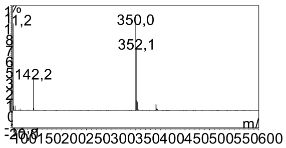

350,0
352,1
142,2
m/

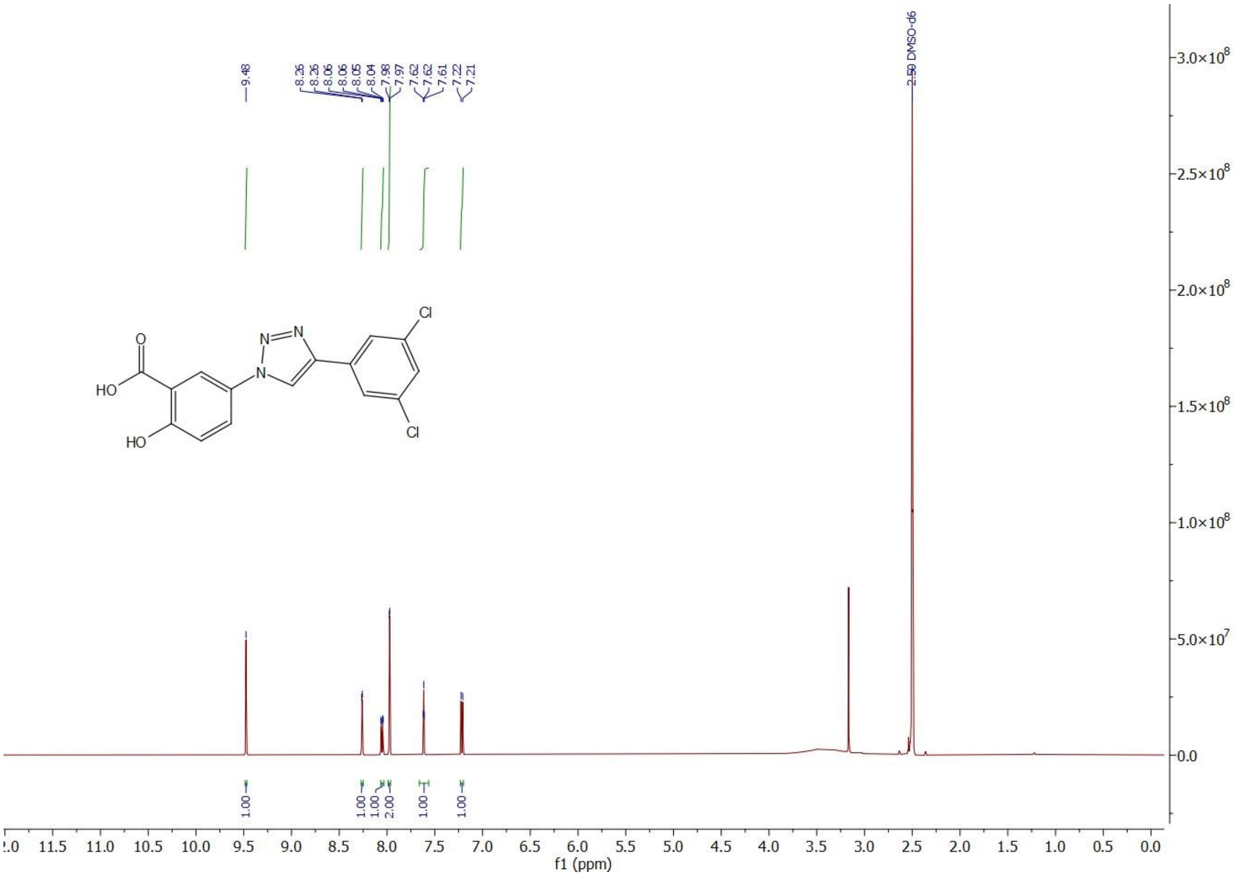

9.48
8.26
8.26
8.06
8.06
8.05
8.04
7.98
7.97
7.62
7.62
7.61
7.22
7.21
2.50 DMSO-d6
HO
O
HO
N
N
N
Cl
Cl
1.00
1.00
1.00
2.00
1.00
1.00
f1 (ppm)

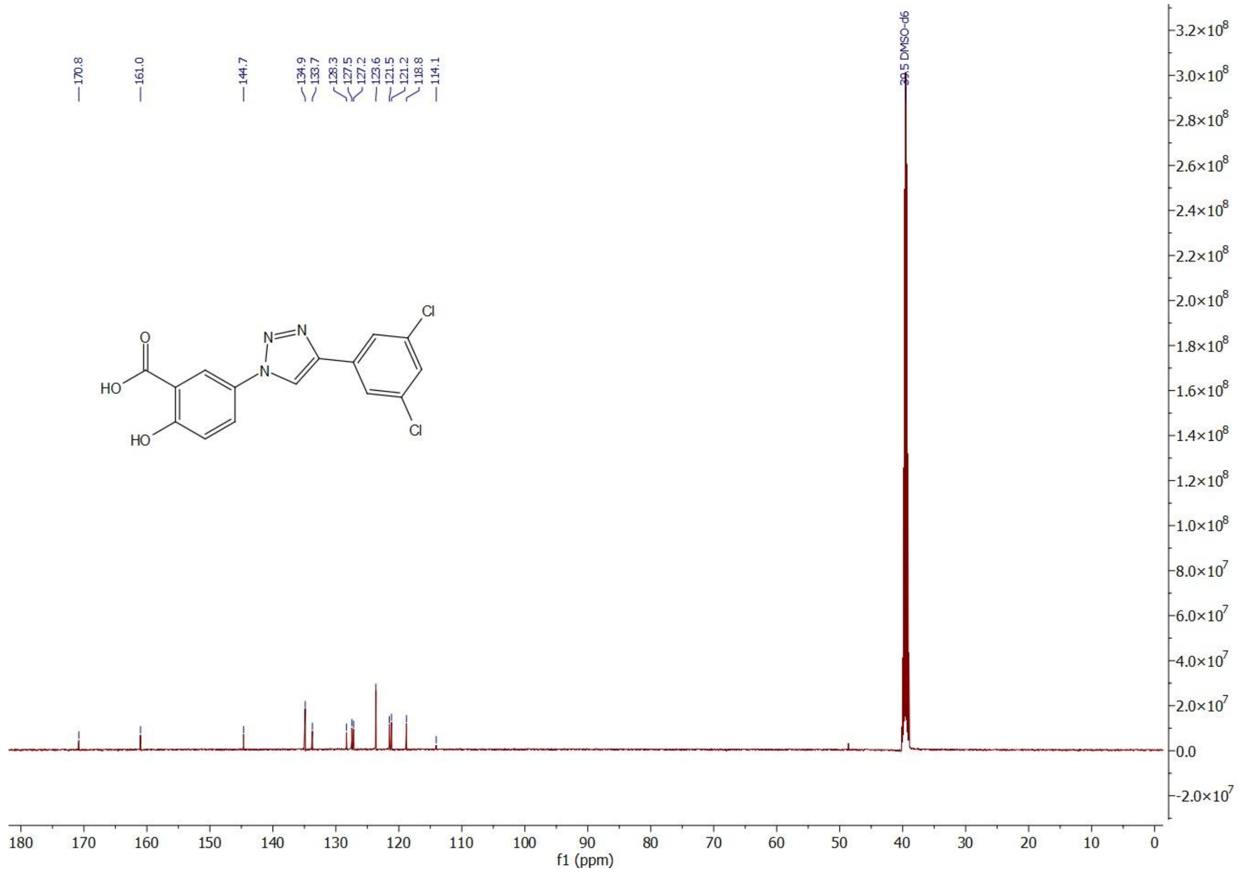

Compound 65

HR-MS, HPLC-MS, ^{1}H-NMR, ^{13}C-NMR

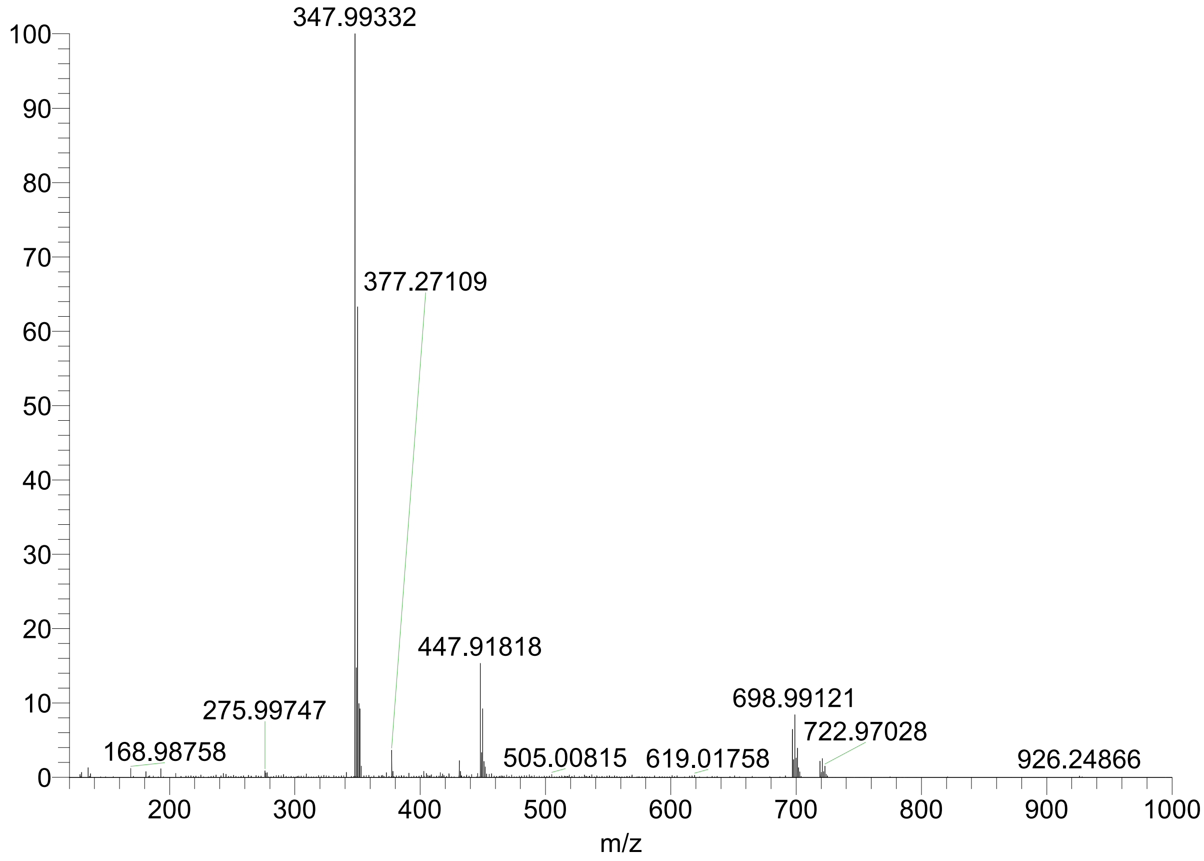
347.99332
377.27109
447.91818
275.99747
168.98758
505.00815
619.01758
698.99121
722.97028
926.24866
m/z

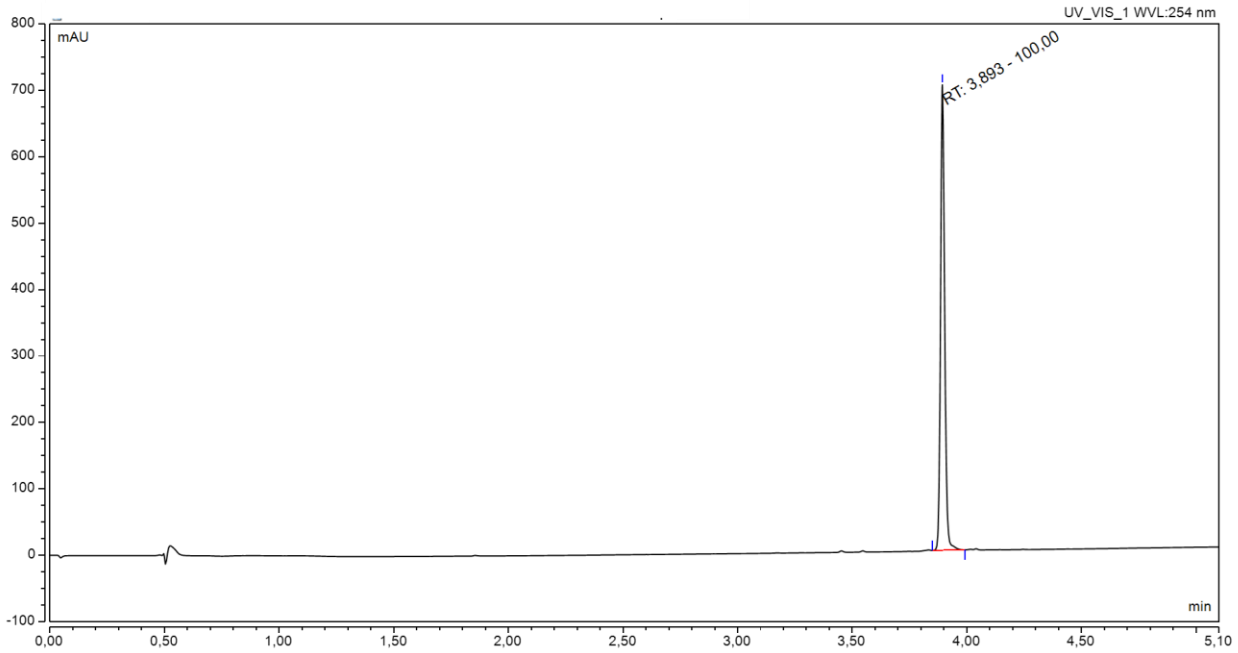
UV_VIS_1 WVL:254 nm
mAU
RT: 3,893 - 100,00
min

Purity = 100%

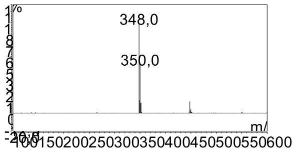

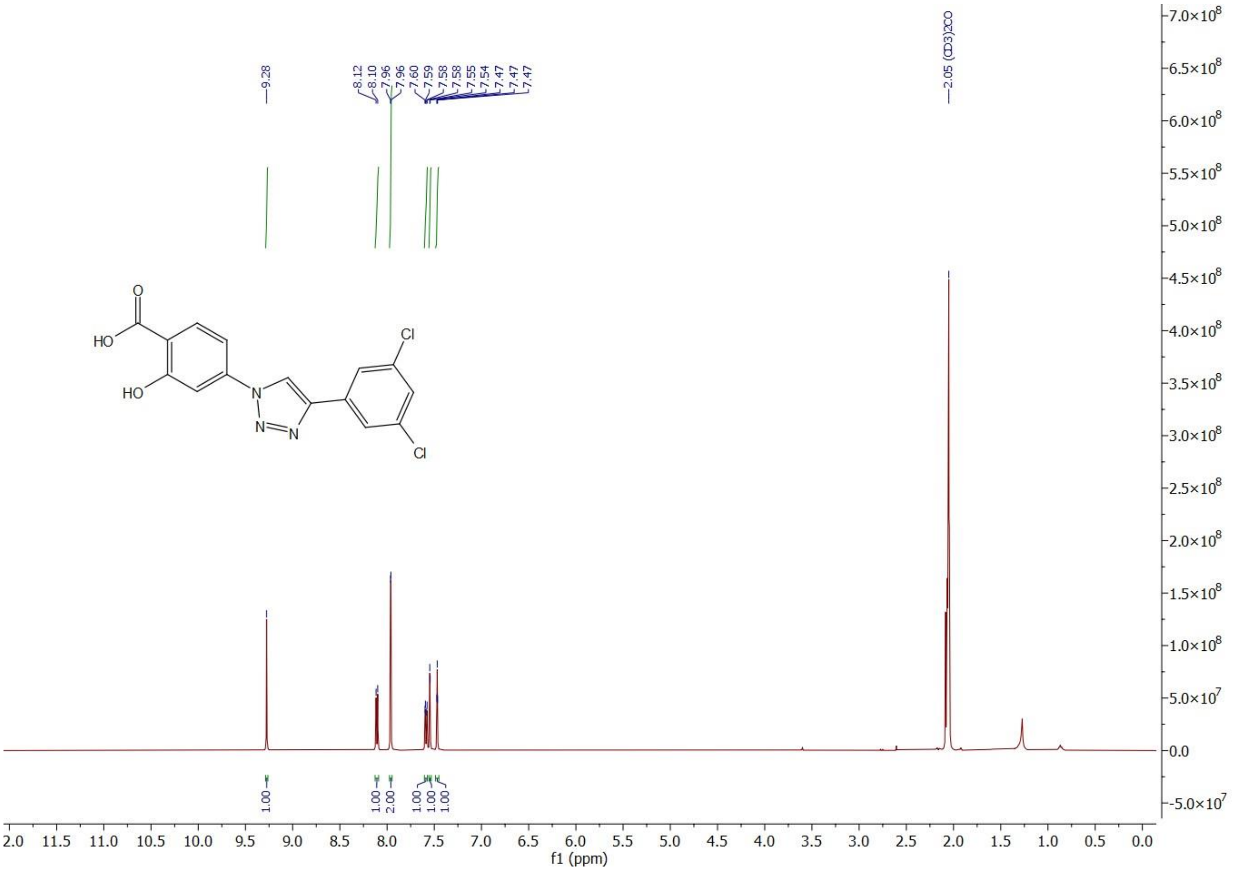

9.28
8.12
8.10
7.96
7.96
7.60
7.59
7.58
7.58
7.55
7.54
7.47
7.47
7.47
2.05 (CD3)2CO
HO
O
HO
N
N
N
Cl
Cl
f1 (ppm)

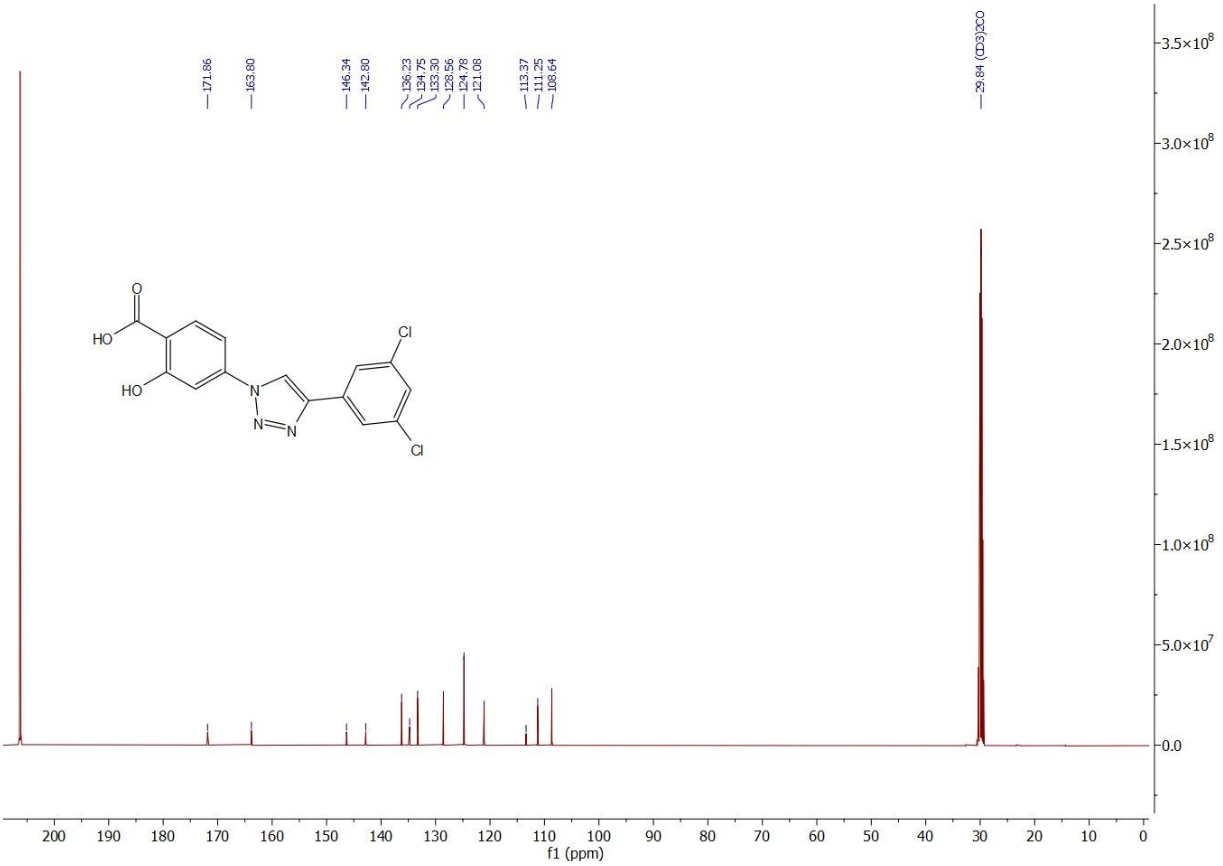

Compound 66

HR-MS, HPLC-MS, ^{1}H-NMR, ^{13}C-NMR

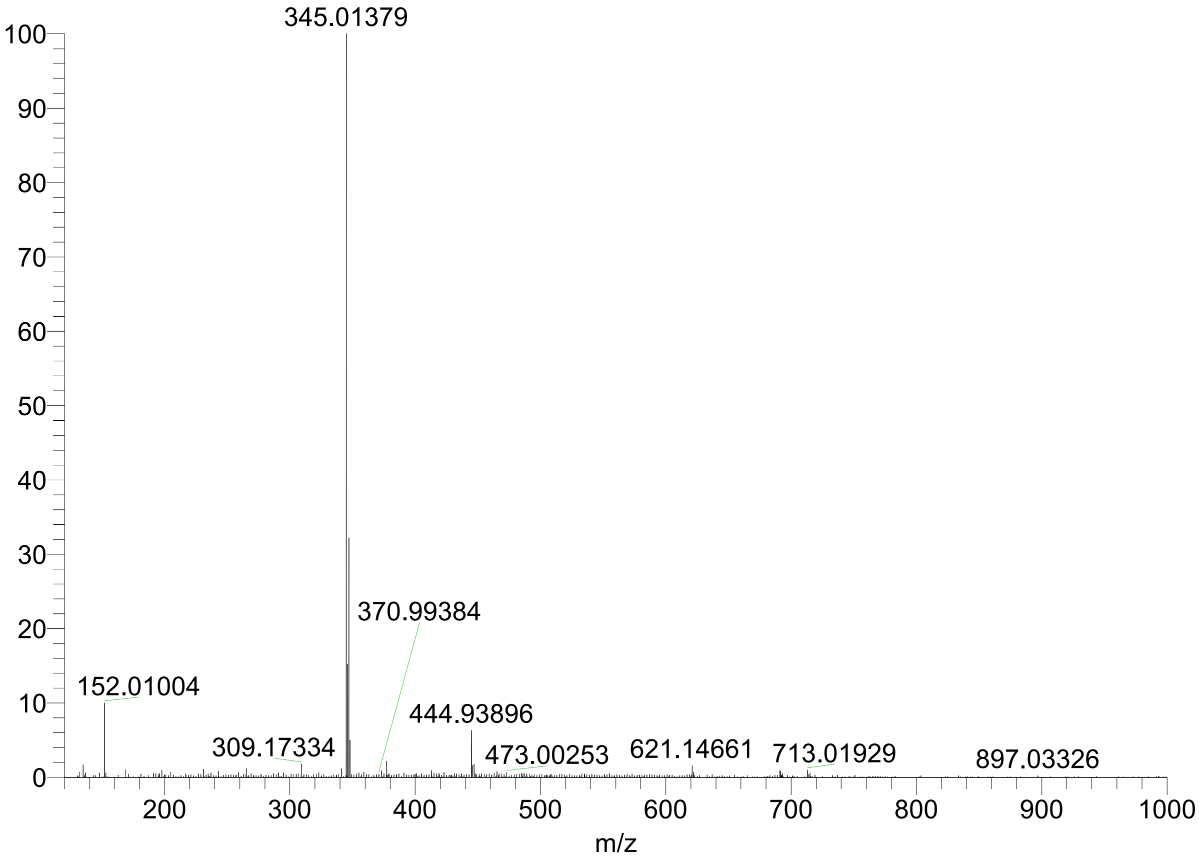
345.01379
370.99384
152.01004
444.93896
309.17334
473.00253
621.14661
713.01929
897.03326
m/z

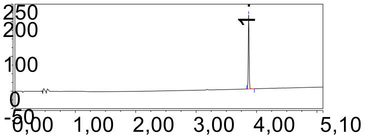

Purity = 100%

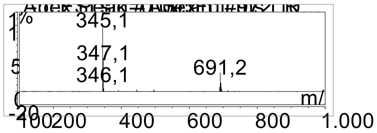
345,1
347,1
346,1
691,2
m/
100 200 400 600 800 1.000

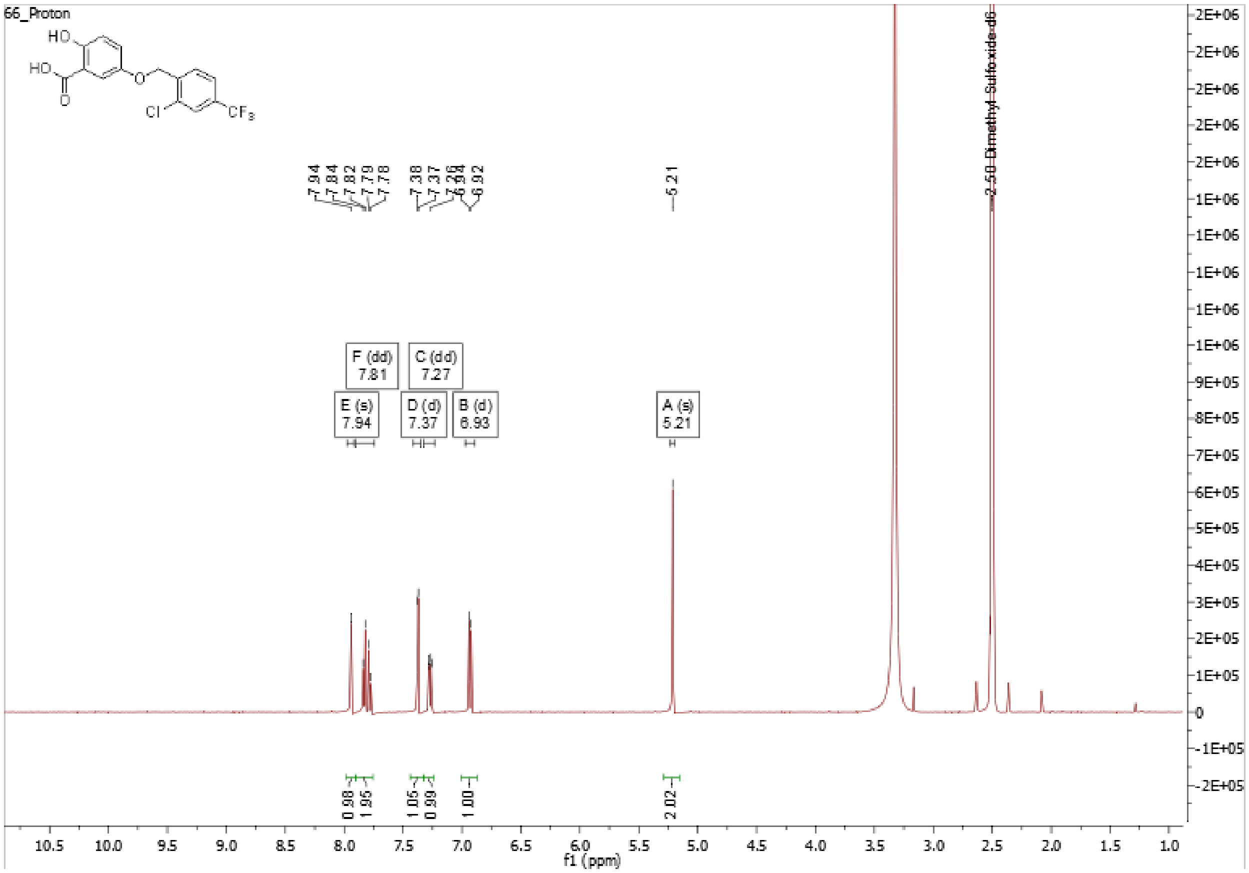
66_Proton
HO
HO
O
O
Cl
CF3
7.94
7.84
7.82
7.79
7.78
7.38
7.37
7.26
6.94
6.92
5.21
2.50 Dimethyl Sulfoxide-d6
F (dd) 7.81
C (dd) 7.27
E (s) 7.94
D (d) 7.37
B (d) 6.93
A (s) 5.21
0.98
1.95
1.05
0.99
1.00
2.02
f1 (ppm)

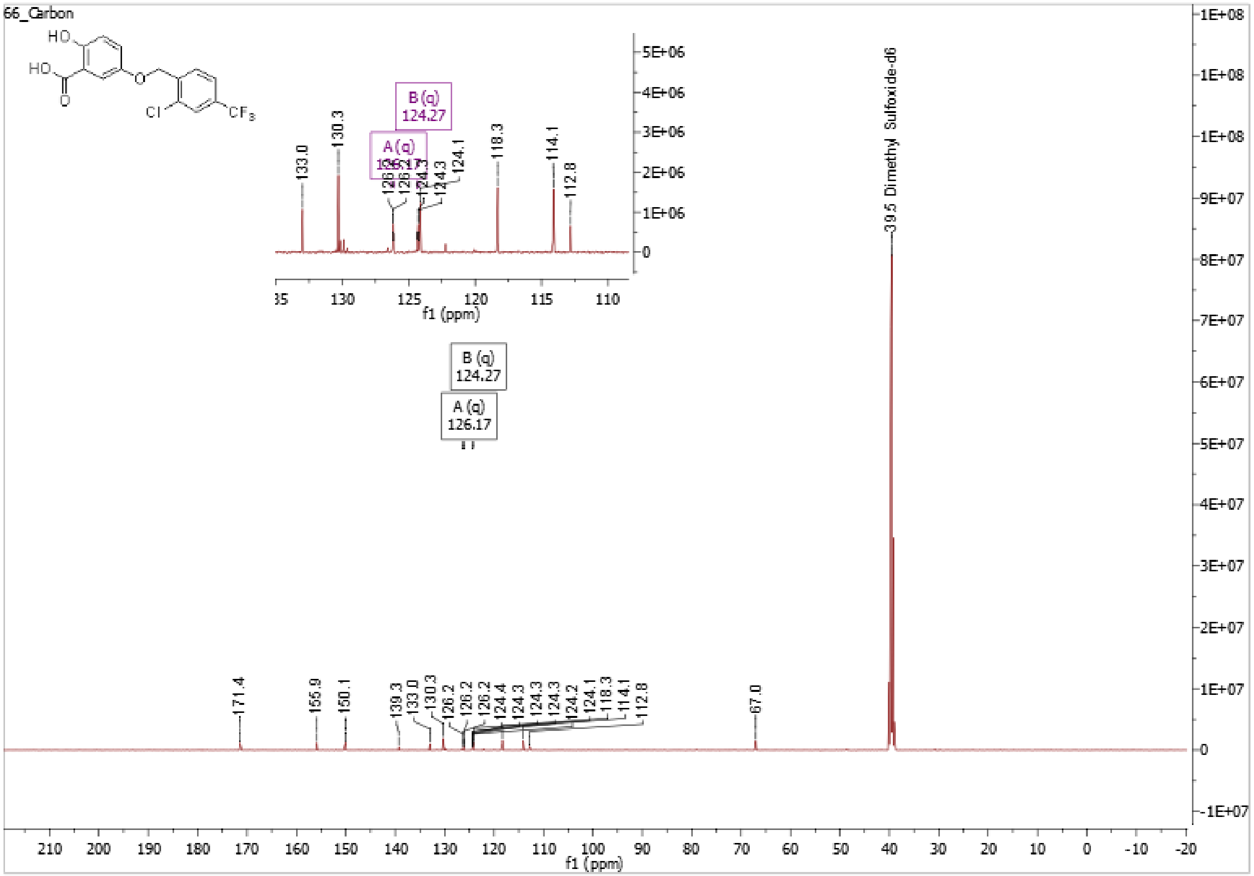
66_Carbon
HO
HO
O
O
Cl
CF3
B (q)
124.27
A (q)
126.17
133.0
130.3
124.1
118.3
114.1
112.8
f1 (ppm)
5E+06
4E+06
3E+06
2E+06
1E+06
0
171.4
155.9
150.1
139.3
133.0
130.3
126.2
126.2
124.4
124.3
124.3
124.2
124.1
118.3
114.1
112.8
67.0
39.5 Dimethyl Sulfoxide-d6
f1 (ppm)

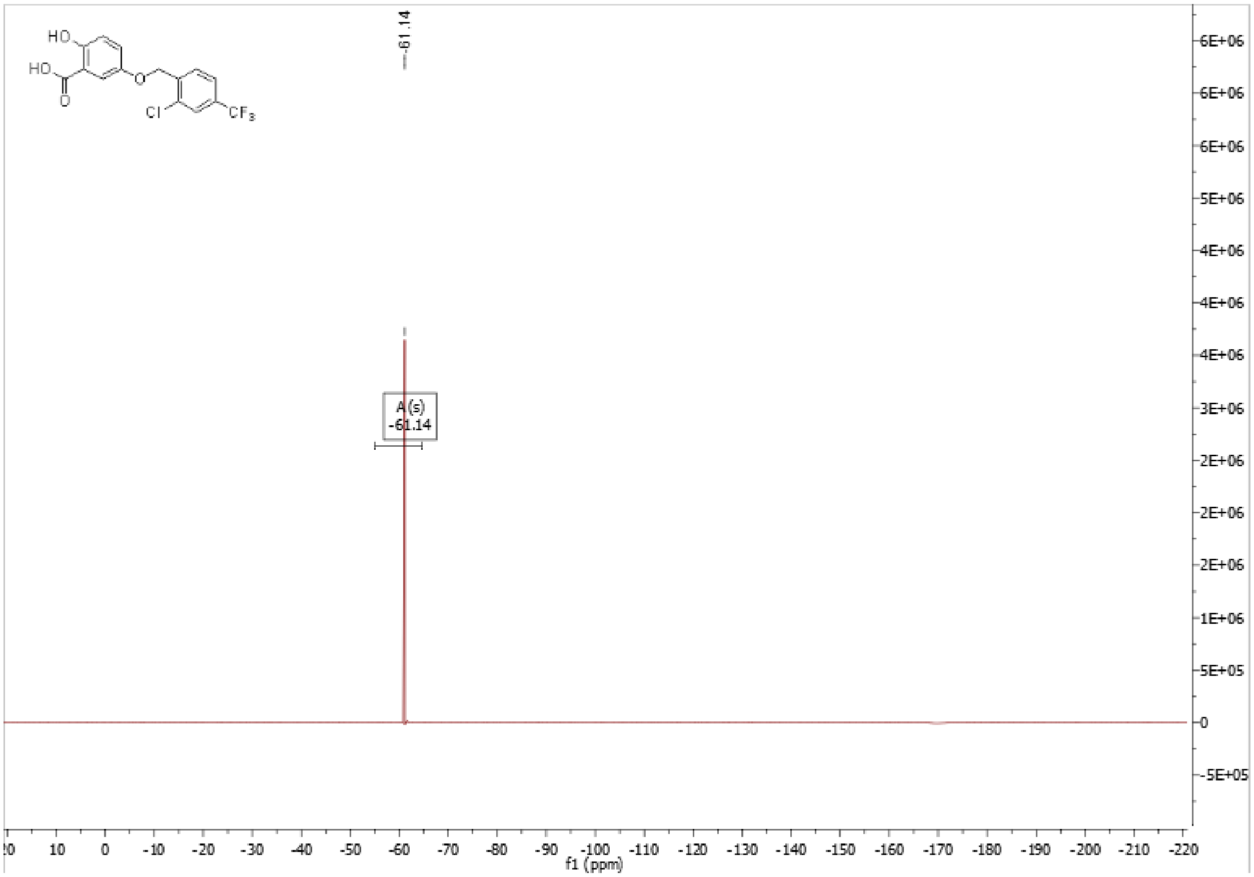

Compound 67

HR-MS, HPLC-MS, ^{1}H-NMR, ^{13}C-NMR

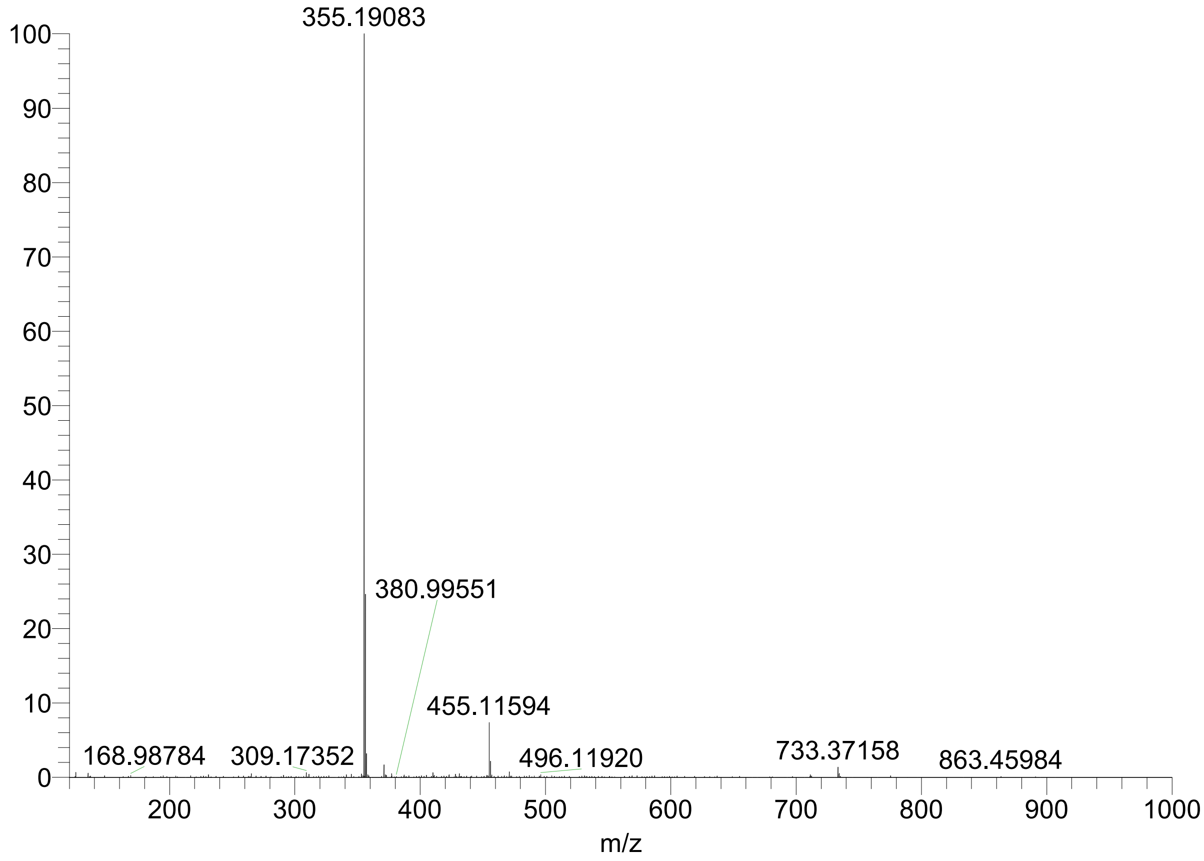
355.19083
380.99551
455.11594
168.98784
309.17352
496.11920
733.37158
863.45984
m/z

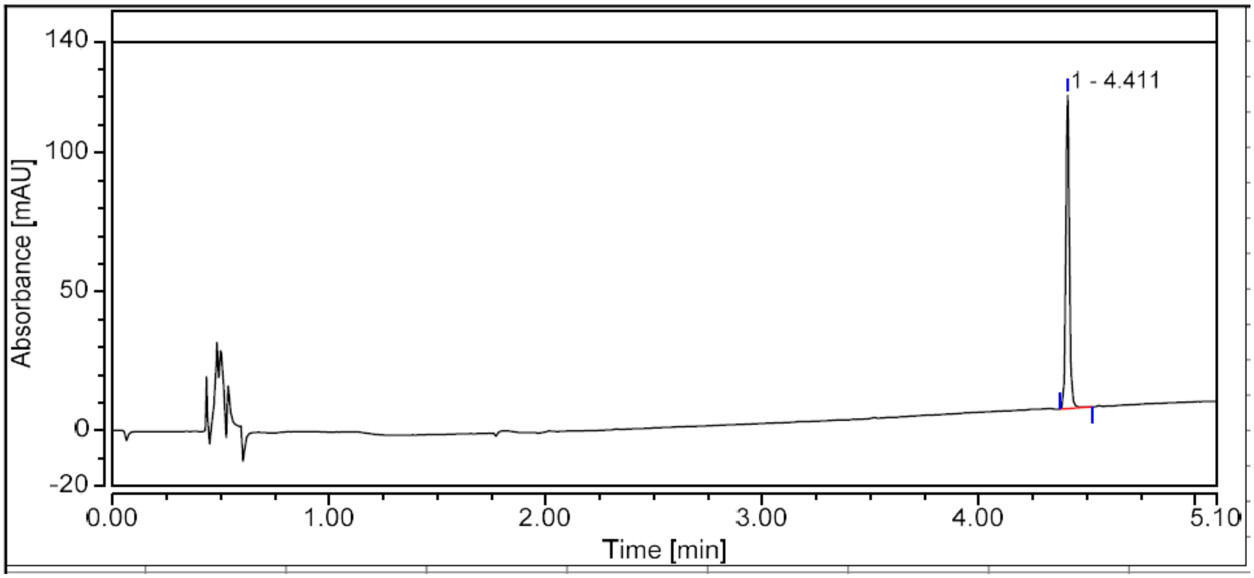
1 - 4.411
Absorbance [mAU]
Time [min]

Purity = 100%

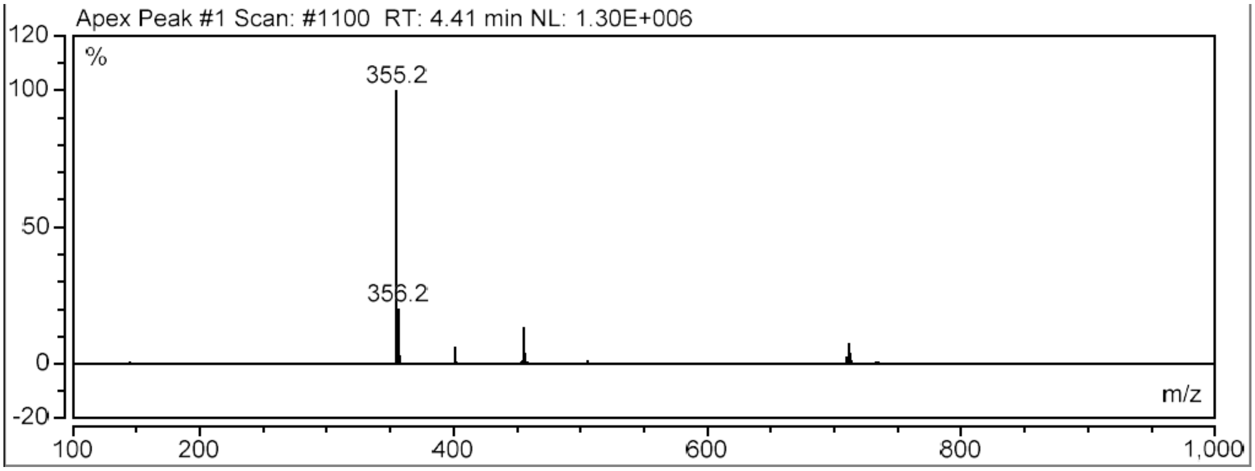
Apex Peak #1 Scan: #1100 RT: 4.41 min NL: 1.30E+006
%
355.2
356.2
m/z

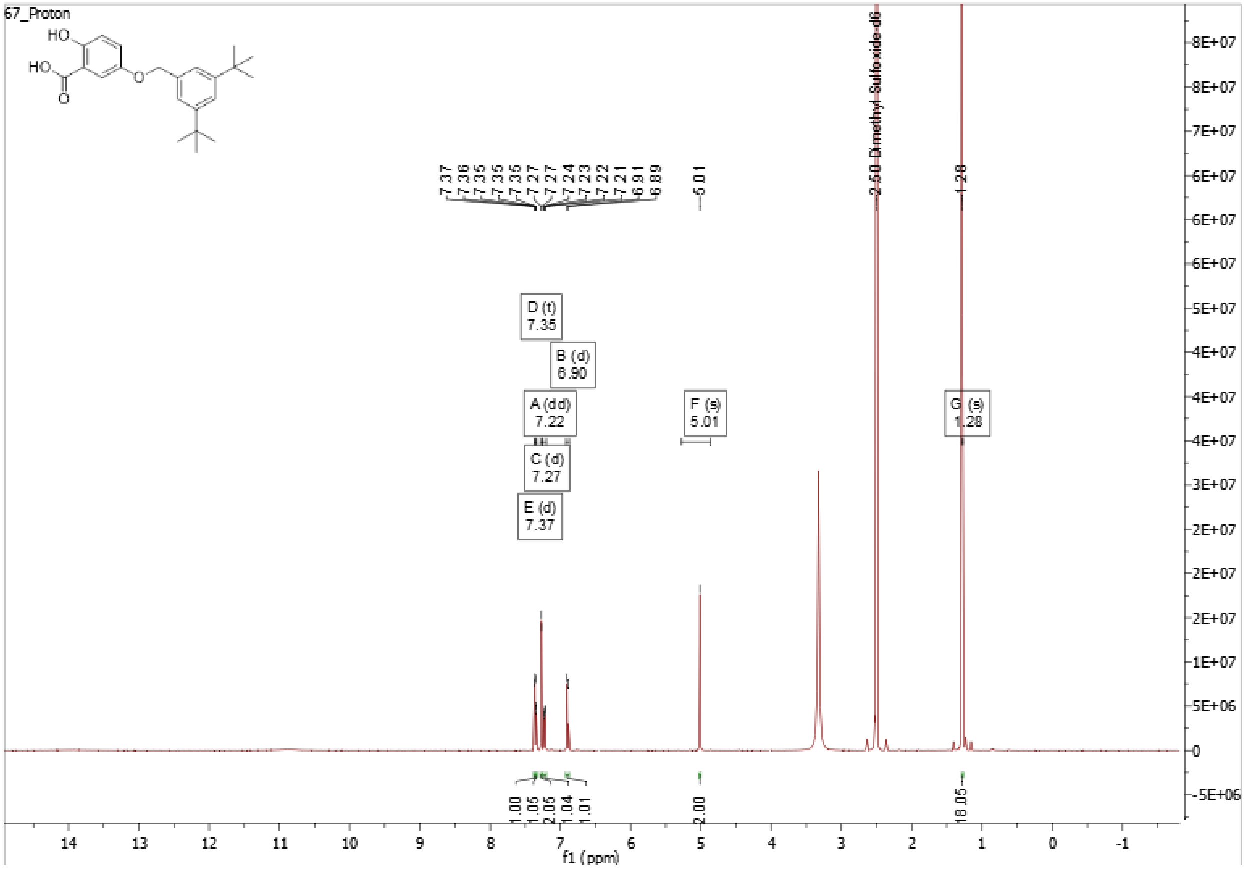
67_Proton
2.50 Dimethyl Sulfoxide-d6
D (t) 7.35
B (d) 6.90
A (dd) 7.22
C (d) 7.27
E (d) 7.37
F (s) 5.01
G (s) 1.28
f1 (ppm)

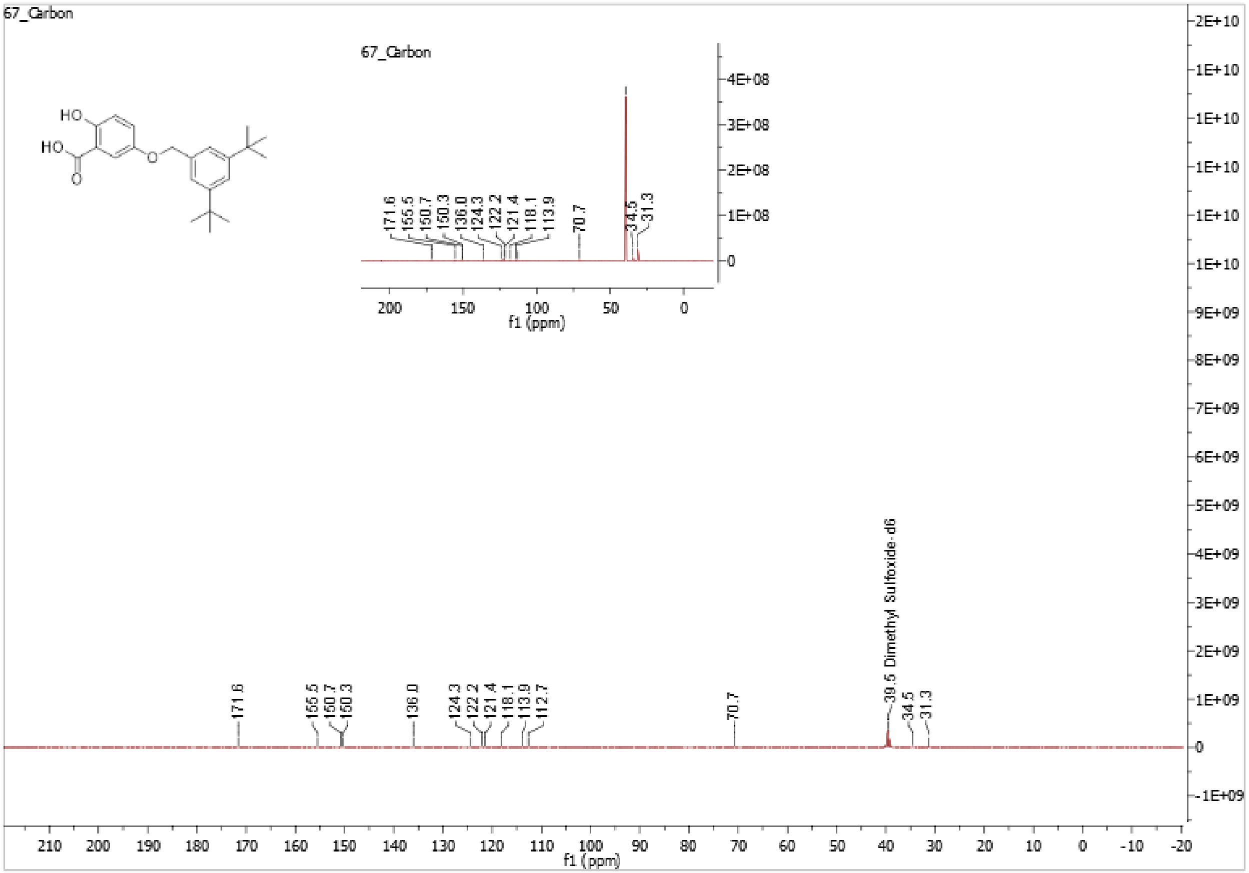

Compound 68

HR-MS, HPLC-MS, ^{1}H-NMR, ^{13}C-NMR

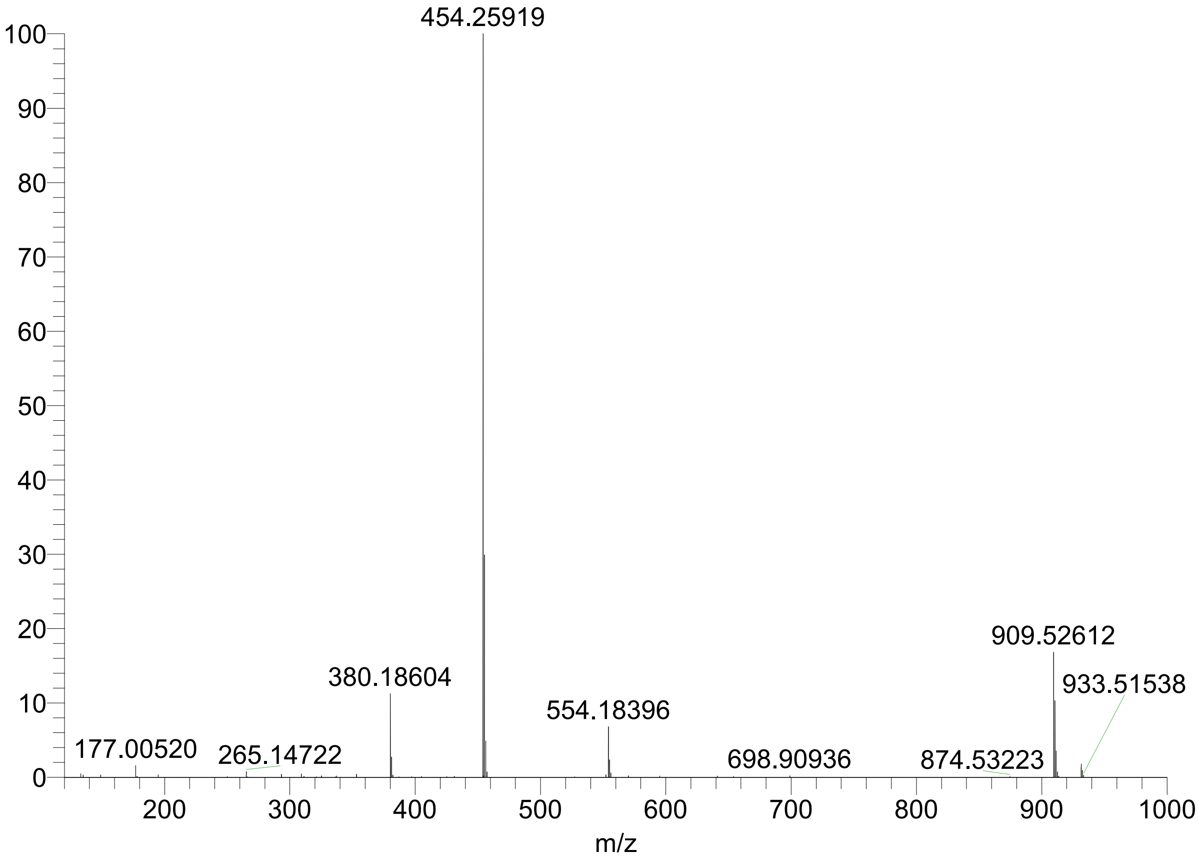
454.25919
380.18604
554.18396
909.52612
933.51538
177.00520
265.14722
698.90936
874.53223
m/z

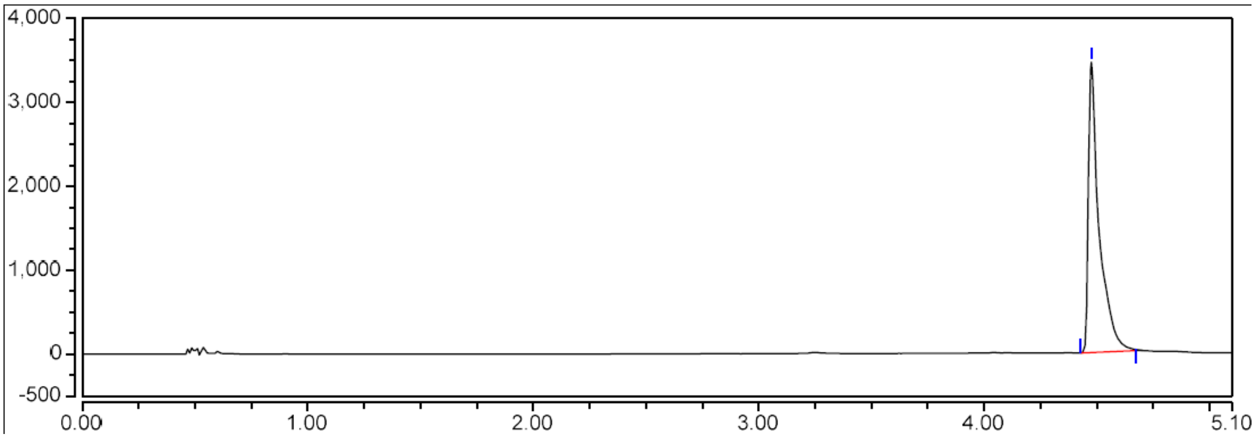

Purity = 100%

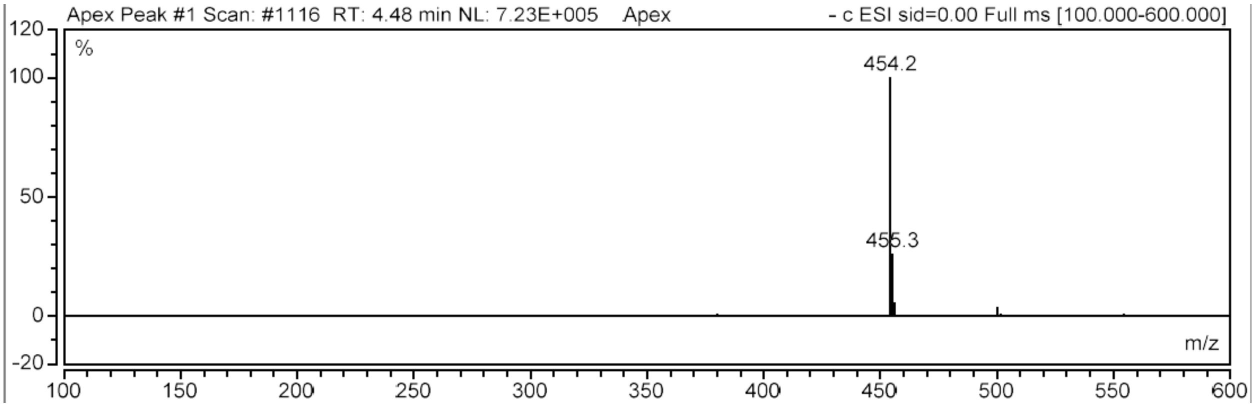

Apex Peak #1 Scan: #1116 RT: 4.48 min NL: 7.23E+005 Apex
- c ESI sid=0.00 Full ms [100.000-600.000]
%
454.2
455.3
m/z

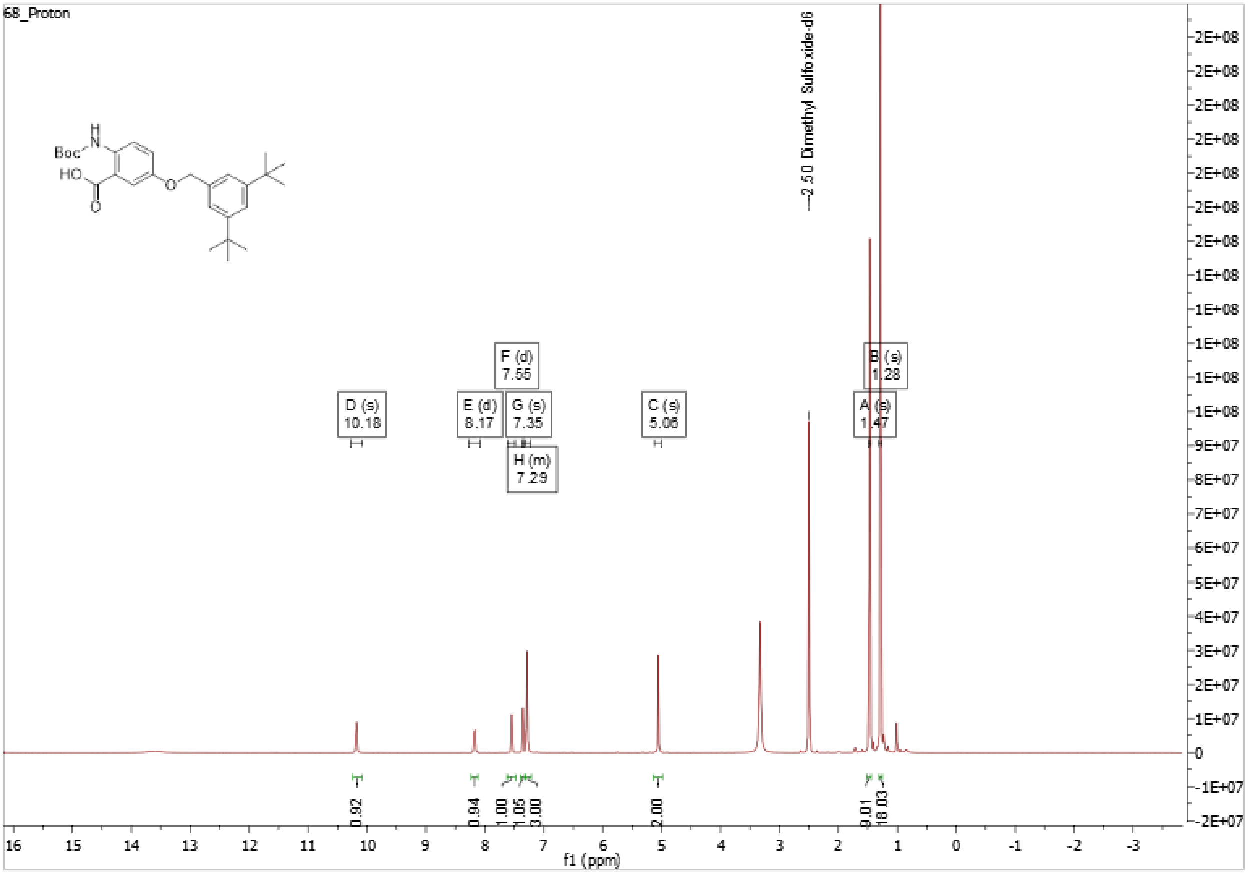

68_Proton
Boc
HO
2.50 Dimethyl Sulfoxide-d6
D (s) 10.18
E (d) 8.17
F (d) 7.55
G (s) 7.35
H (m) 7.29
C (s) 5.06
A (s) 1.47
B (s) 1.28
0.92
0.94
1.00
1.05
3.00
2.00
9.01
18.03
f1 (ppm)

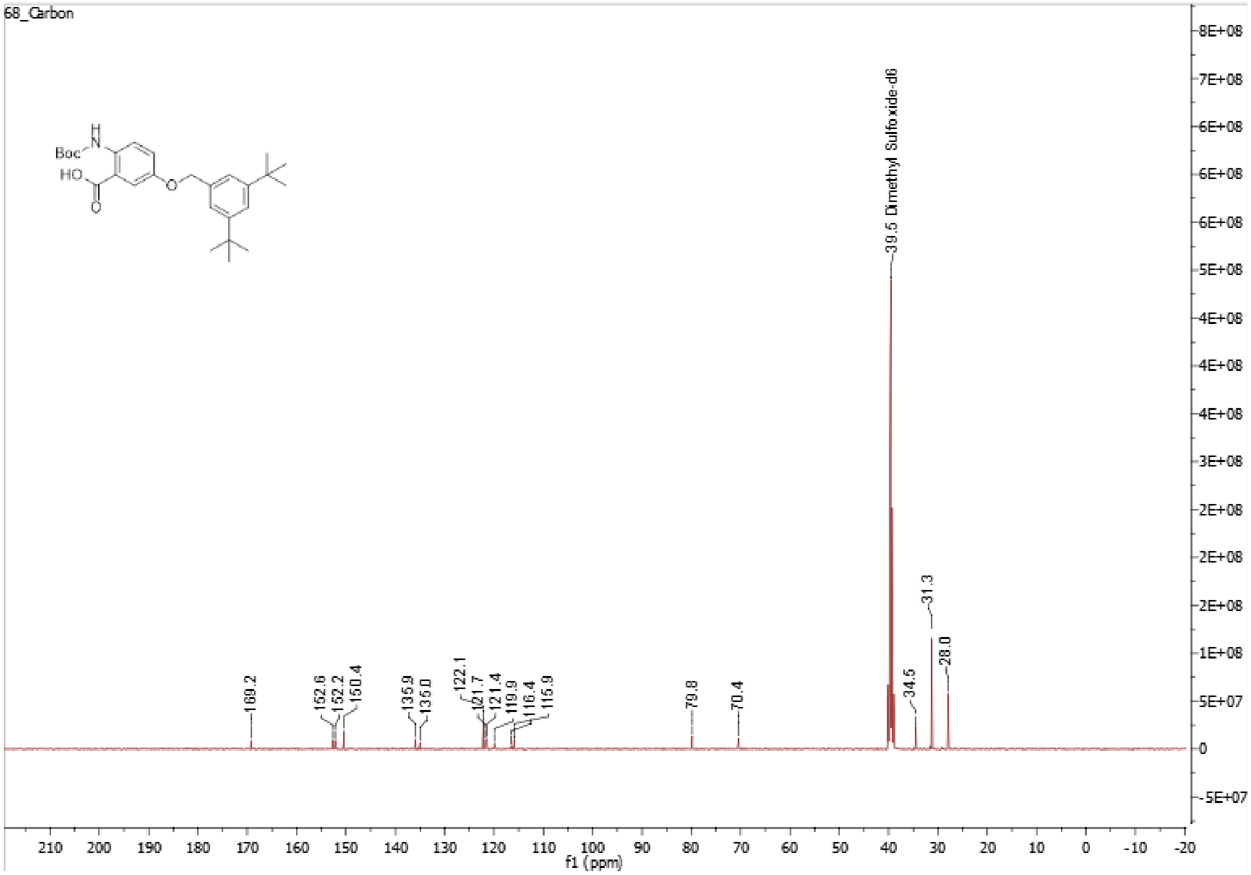

Compound 70

HR-MS, HPLC-MS, ^{1}H-NMR, ^{13}C-NMR

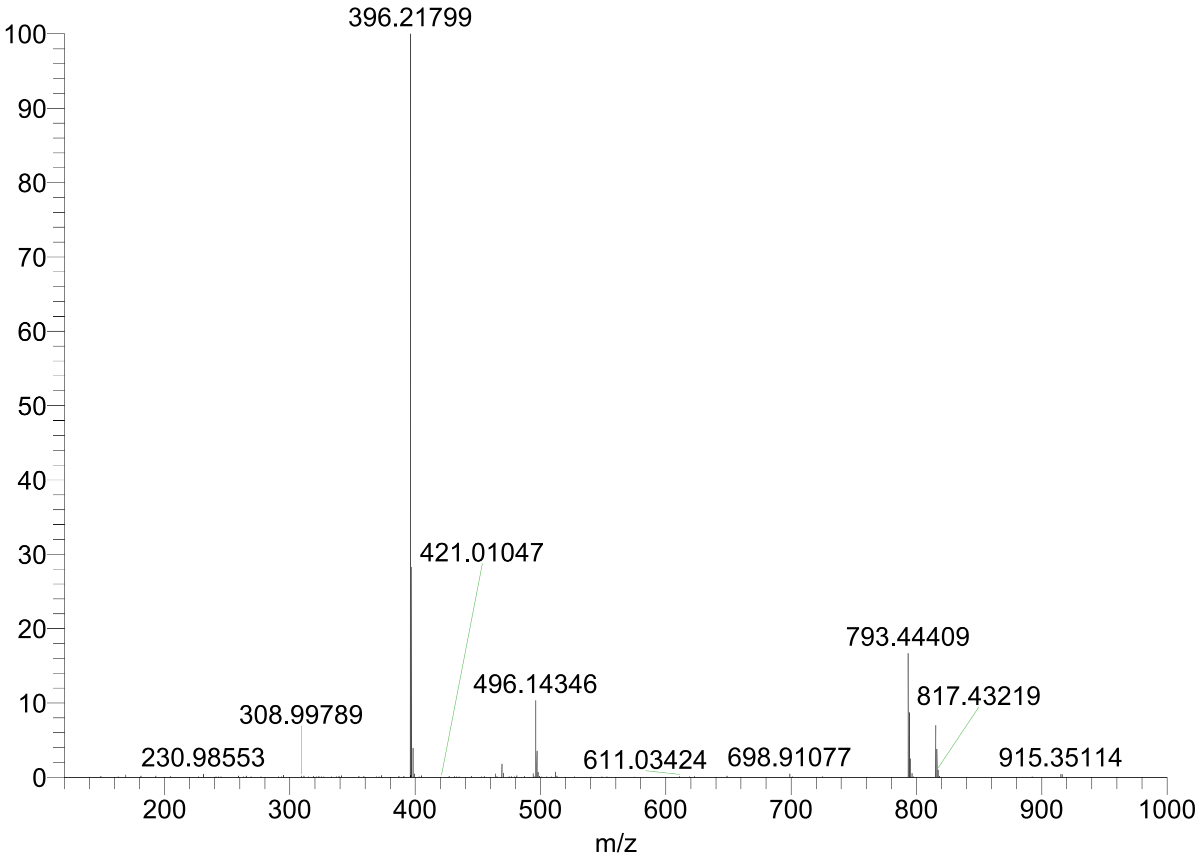
396.21799
421.01047
496.14346
308.99789
230.98553
611.03424
698.91077
793.44409
817.43219
915.35114
100
90
80
70
60
50
40
30
20
10
0
200
300
400
500
600
700
800
900
1000
m/z

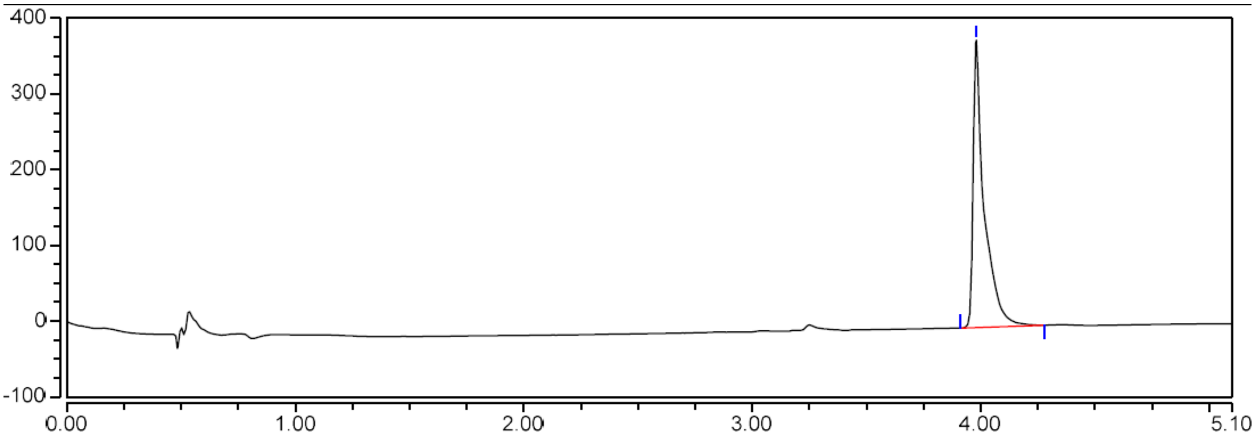
400
300
200
100
0
-100
0.00
1.00
2.00
3.00
4.00
5.10

Purity = 100%

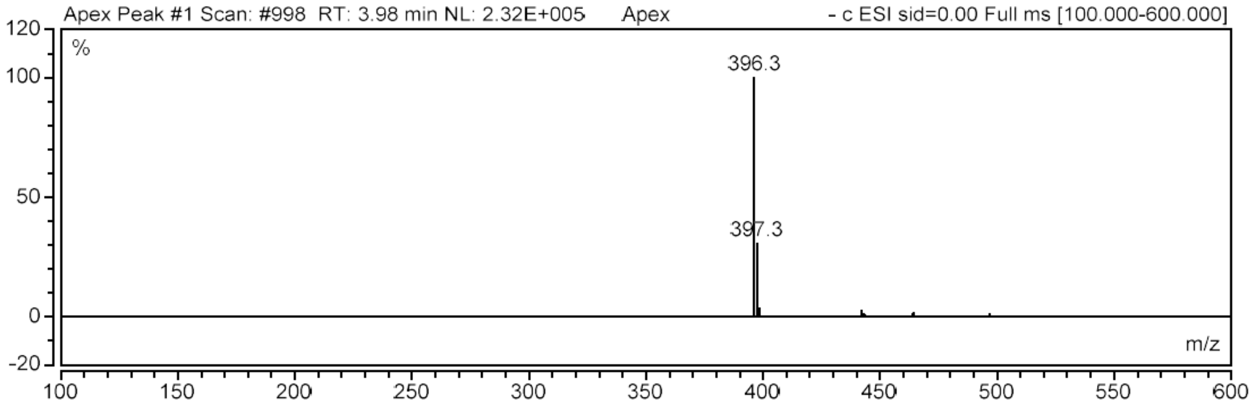
Apex Peak #1 Scan: #998 RT: 3.98 min NL: 2.32E+005 Apex
- c ESI sid=0.00 Full ms [100.000-600.000]
%
396.3
397.3
m/z

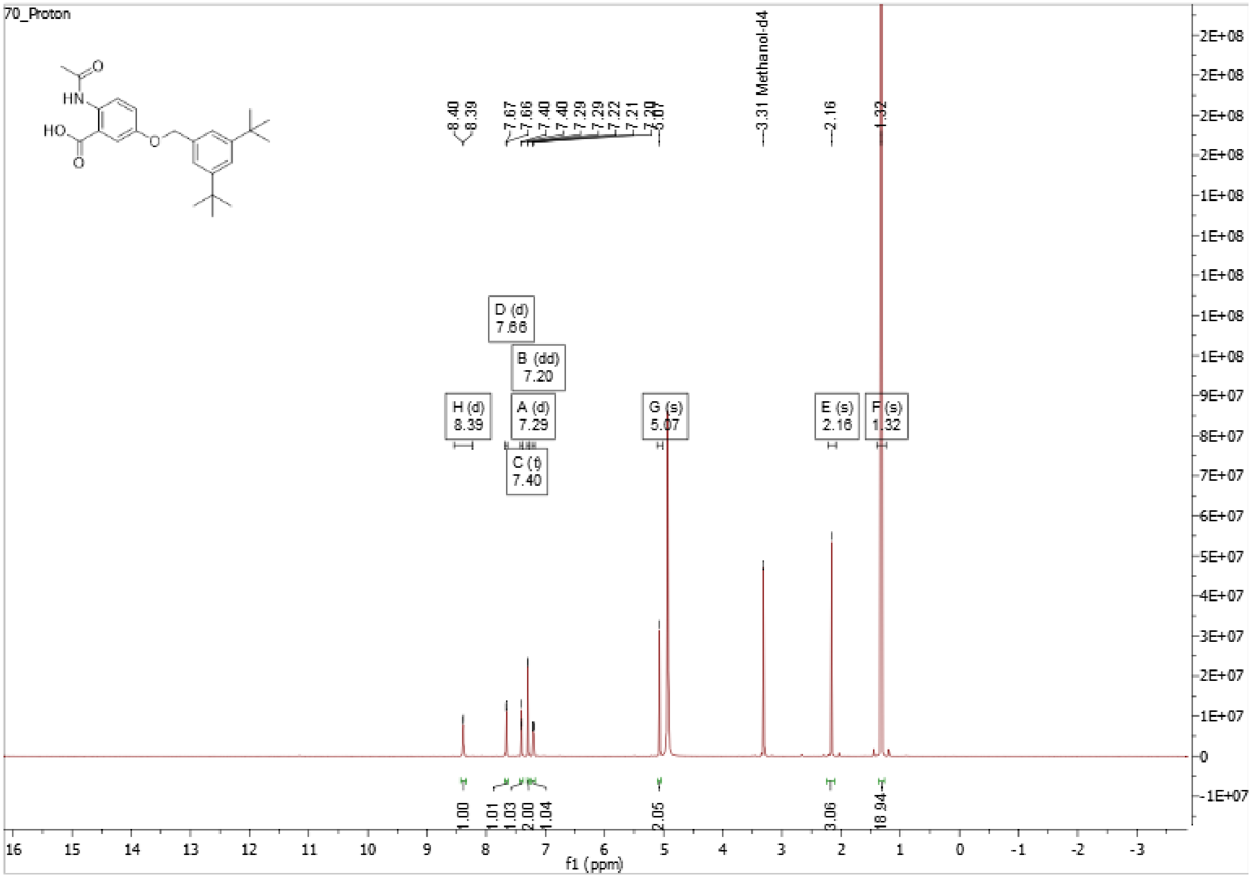
70_Proton
HN
HO
O
3.31 Methanol-d4
D (d) 7.66
B (dd) 7.20
H (d) 8.39
A (d) 7.29
C (t) 7.40
G (s) 5.07
E (s) 2.16
F (s) 1.32
f1 (ppm)

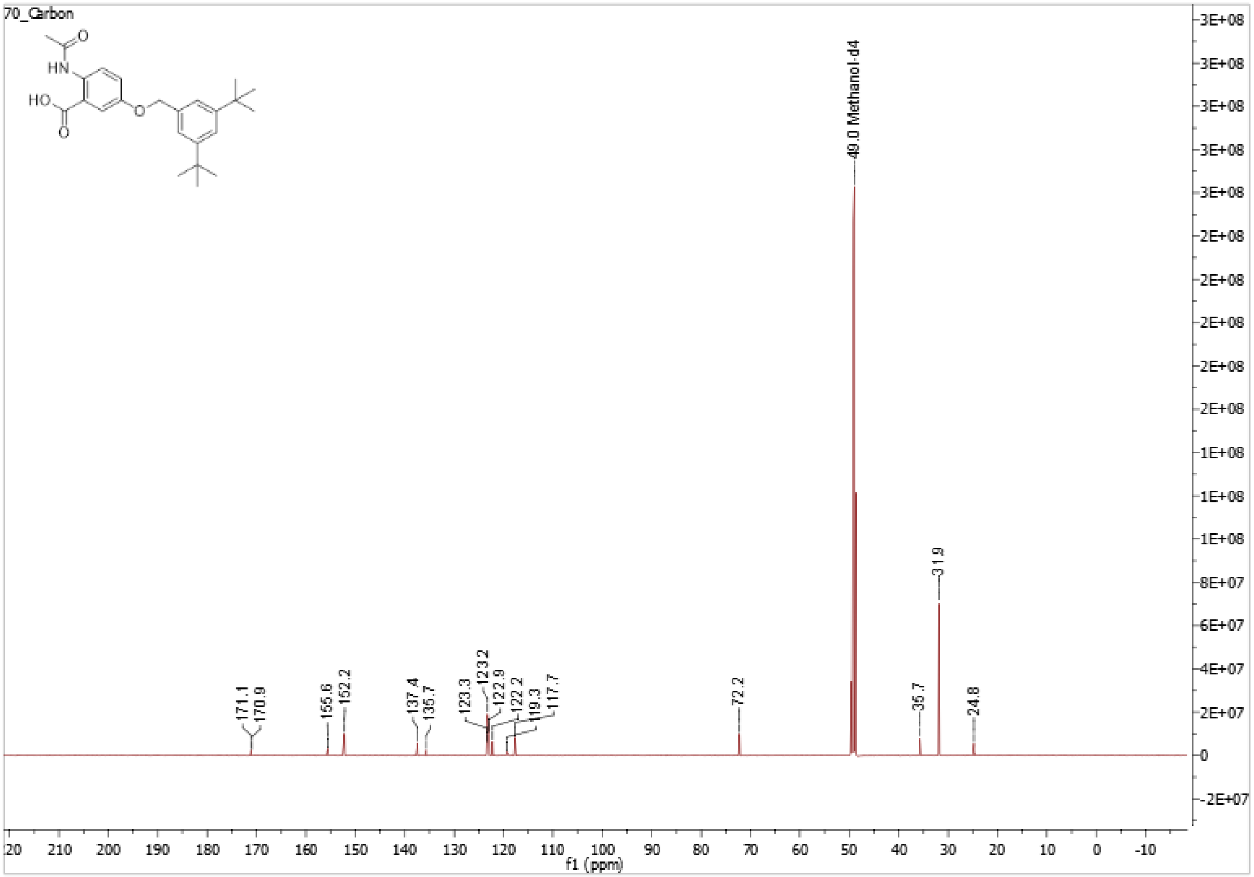

References

(1) Diamanti, E.; Souza, P. C. T.; Setyawati, I.; Bousis, S.; Gomez, L. M.; Swier, L.; Shams, A.; Tsarenko, A.; Stanek, W. K.; Jager, M.; et al. Identification of inhibitors targeting the energy-coupling factor (ECF) transporters. *Commun Biol* **2023**, *6* (1), 1182.

(2) Bousis, S.; Winkler, S.; Haupenthal, J.; Fulco, F.; Diamanti, E.; Hirsch, A. K. H. An Efficient Way to Screen Inhibitors of Energy-Coupling Factor (ECF) Transporters in a Bacterial Uptake Assay. *Int. J. Mol. Sci.* **2022**, *23* (5).

(3) Drost, M.; Diamanti, E.; Fuhrmann, K.; Goes, A.; Shams, A.; Haupenthal, J.; Koch, M.; Hirsch, A. K. H.; Fuhrmann, G. Bacteriomimetic Liposomes Improve Antibiotic Activity of a Novel Energy-Coupling Factor Transporter Inhibitor. *Pharmaceutics* **2021**, *14* (1).

(4) Richmond, E.; Moran, J. Ligand Control of E/Z Selectivity in Nickel-Catalyzed Transfer Hydrogenative Alkyne Semireduction. *J. Org. Chem.* **2015**, *80* (13), 6922-6929.

(5) Swier, L. J.; Guskov, A.; Slotboom, D. J. Structural insight in the toppling mechanism of an energy-coupling factor transporter. *Nat. Commun.* **2016**, *7*, 11072.

(6)Shams, A.; Bousis, S.; Diamanti, E.; Elgaher, WAM; Zeimetz, L.; Haupenthal, J.; Slotboom, D. J.; Hirsch, A. K. H. Expression andcharacterization of pantothenate energy-couplingfactor transporters as an anti-infective drug target. *Protein Sci.* **2024**, 33.
